# Direct Photoexcitation of Ethynylbenziodoxolones: An Alternative to Photocatalysis for Alkynylation Reactions**

**DOI:** 10.1002/anie.202110257

**Published:** 2021-09-21

**Authors:** Stephanie G. E. Amos, Diana Cavalli, Franck Le Vaillant, Jerome Waser

**Affiliations:** ^1^ Laboratory of Catalysis and Organic Synthesis and National Centre of Competence in Research (NCCR) Catalysis Institut des Sciences et Ingénierie Chimique Ecole Polytechnique Fédérale de Lausanne CH-1015 Lausanne Switzerland; ^2^ Max-Planck-Institut für Kohlenforschung Mülheim an der Ruhr 45470 Germany

**Keywords:** alkynes, hypervalent iodine, photochemistry, quaternary centers, synthetic methods

## Abstract

Ethynylbenziodoxolones (EBXs) are commonly used as radical traps in photocatalytic alkynylations. Herein, we report that aryl‐substituted EBX reagents can be directly activated by visible light irradiation. They act as both oxidants and radical traps, alleviating the need for a photocatalyst in several reported EBX‐mediated processes, including decarboxylative and deboronative alkynylations, the oxyalkynylation of enamides and the C−H alkynylation of THF. Furthermore, the method could be applied to the synthesis of alkynylated quaternary centers from tertiary alcohols via stable oxalate salts and from tertiary amines via aryl imines. A photocatalytic process using 4CzIPN as an organic dye was also developed for the deoxyalkynylation of oxalates.

## Graphical abstract

Aryl ethynylbenziodoxolones (ArEBXs) can be directly activated by visible light irradiation. In the excited state, ArEBXs act as strong oxidants, alleviating the need for a photocatalyst in decarboxylative and deboronative alkynylations, the oxyalkynylation of enamides, and the C−H alkynylation of THF.

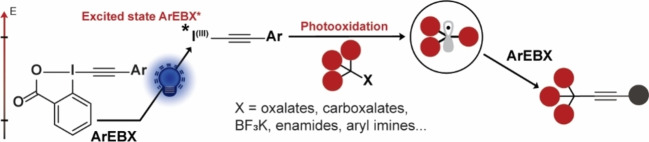

## Introduction

**Scheme 1:**
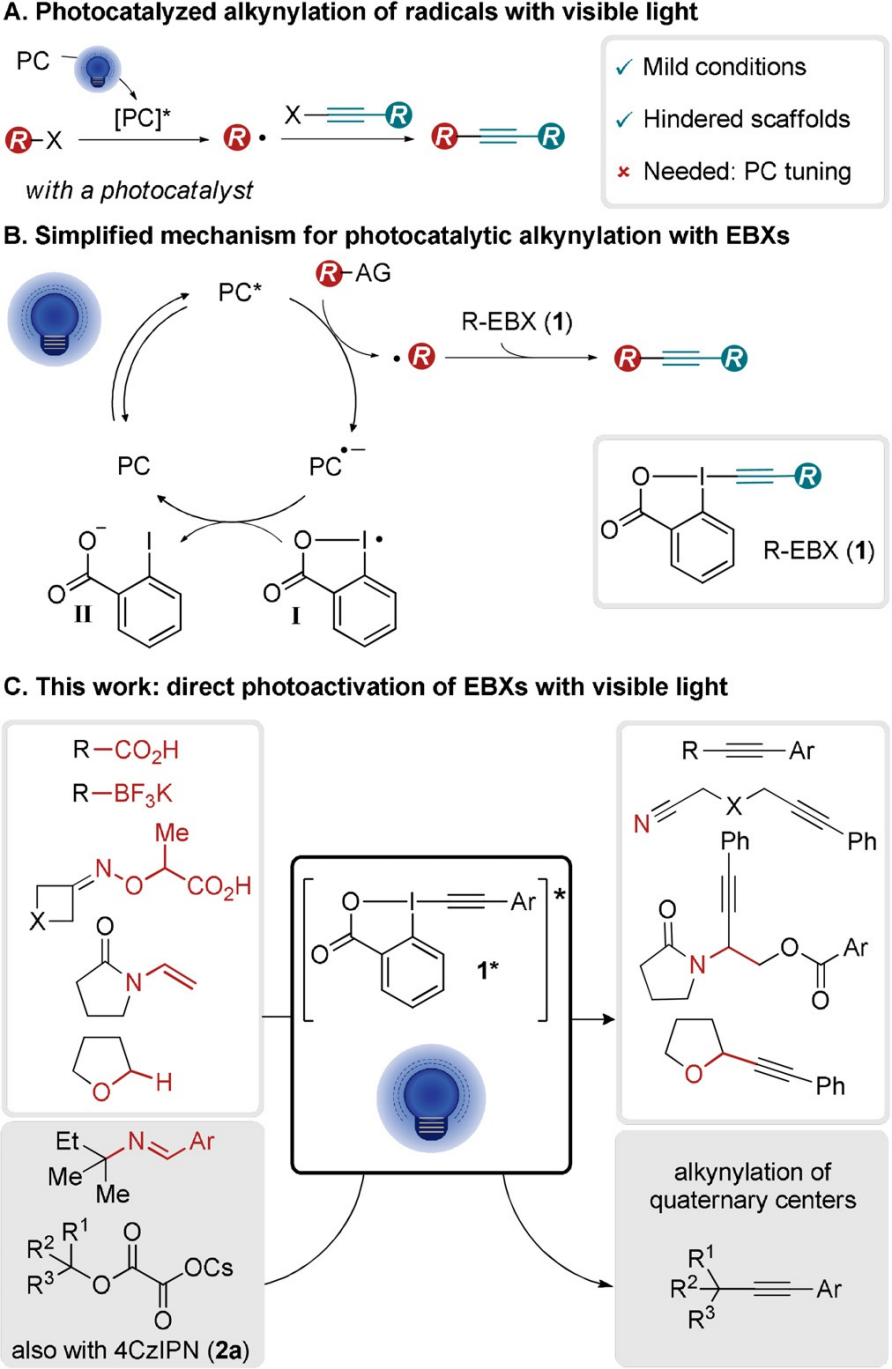
Photomediated alkynylation and use of EBX reagents as radical traps with or without photocatalyst. PC=photocatalyst, AG=activating group.

Alkynes have found broad applications in synthetic and medicinal chemistry, chemical biology, and materials science.^[1]^ They can be used either as an inert and rigid connecting element or as a reactive unit.^[2]^ Therefore, it is not surprising that synthetic methods for accessing alkynes are the focus of intensive research. In addition to alkynylations of nucleophiles and electrophiles, the alkynylation of carbon radicals has emerged as an attractive complementary route for the synthesis of alkynes, enabling in particular the synthesis of highly sterically hindered systems, such as quaternary centers.[^3^, ^4^] These compounds have found numerous applications in the total synthesis of natural products and medicinal chemistry.^[5]^ Recently, particular attention has been placed on visible light mediated alkynylations with a photocatalyst as they enable the generation of highly reactive open‐shell species such as radicals avoiding the use of strong UV irradiation or toxic precursors (Scheme 1 A). However, fine‐tuning of the photocatalyst is required for each new alkynylation process.

Among possible radical traps, Ethynylbenziodoxolones (EBXs, **1**) hypervalent iodine reagents have been especially successful.[^3^, ^6^] Photomediated alkynylations with EBXs (**1**) follow usually a reductive quenching mechanism,^[7]^ in which an excited state photocatalyst (PC*) is required to generate a carbon radical by oxidation of the substrate (Scheme 1 B). The reaction of the radical with EBXs (**1**) gives then the alkynylation product and iodanyl radical **I**. For an efficient catalytic process, the reduced photocatalyst **PC^.−^** needs to be reoxidized by iodanyl radical **I** to give the ground state photocatalyst and benzoate **II**. When considering that several steps in the catalytic cycle involve reactive species present in low concentration, it is not surprising that fine‐tuning of both catalyst structure and reaction conditions is required for success.

Herein, we report the serendipitous discovery of a different alkynylation approach via the visible light photoexcitation of aryl‐EBX reagents, alleviating the need for a fine‐tuned photocatalyst (Scheme 1 C). Visible light irradiation can promote the excitation of a variety of hypervalent iodine reagents through spin forbidden transitions.^[8]^ Nevertheless, to the best of our knowledge, this activation mode has never been reported for EBXs. We now demonstrate that the excited state ArEBX* (1*) of ArEBX (**1**) can be used as a photooxidant to activate a variety of oxidizable functional groups, allowing deboronative^[6b]^ and decarboxylative[^6c^, ^6d^, ^6e^] alkynylations, as well as the decarboxylative fragmentation of oximes,^[6f]^ the difunctionalisation of enamides^[6h]^ and the alkynylation of C−H ether bonds.^[9]^ All these processes were reported only in the presence of a photocatalyst previously.

Furthermore, this direct excitation strategy allowed the deoxygenation/deamination–alkynylation of broadly available tertiary alcohols and amines via cesium oxalate salts^[10]^ or aryl imines^[11]^ respectively, giving access to valuable alkynes connected to quaternary centers. These radical precursors have not yet been used to access alkynes. Only one approach used alcohols as precursors, exploiting a reductive substrate activation strategy with unstable and non‐isolable *N*‐phthalimidoyl oxalates as precursors.^[12]^ We especially focused on easily available oxalate salts and developed in addition for these substrates a photocatalytic method with the organic dye 4CzIPN (2,4,5,6‐tetrakis(9*H*‐carbazol‐9‐yl) isophthalonitrile, **2 a**). Whereas the direct photoactivation method stands out for its operational simplicity, the photocatalytic approach usually proceeded in higher yields and tolerate a broader range of alkynes.

## Results and Discussion

When attempting the photocatalytic deoxyalkynylation of cesium oxalate **3 a** (**3 a^.^**/**3 a^−^** =1.3 V vs. SCE)^[10a]^ with PhEBX (**1 a**) and 4CzIPN (**2 a**) as photocatalyst, we discovered in a control experiment that the desired deoxyalkynylated product **4 a** could be obtained in 50 % yield In DCM with 1.5 equiv of **1 a** in absence of **2 a** (Table 1, entry 1). We also observed the formation of ketones **5 a** and **5 b** in 17 and 12 % yield, respectively (entry 1). For this experiment, we used 2 high‐intensity Kessil lamps (40 W each) with a broad bandwidth irradiation around 440 nm as the light source. When frequently used blue LED strips with a lower intensity (8 W) and an irradiation centered around 460 nm were applied, the formation of **4 a** was not observed (entry 2). This is in agreement with previous reports using blue LED strips in which no background reaction is observed in absence of a photocatalyst.[^6b^, ^6c^, ^6d^, ^6e^, ^6f^, ^6g^, ^6h^] With blue LED strips, we still did see conversion of cesium oxalate **3 a** and PhEBX (**1 a**) to compounds **5 a** and **5 b** (entry 2). When the reaction was performed in the dark at 50 °C, ketones **5 a** and **5 b** were obtained in 45 % and 20 % yield (entry 3). This suggests a thermal pathway for the formation of **5 a** and **5 b**. These ketones originated from the formal hydration of **1 a** and the incorporation of a nucleophile (iodobenzoate or oxalate). Using a larger excess of PhEBX (**1 a**) resulted in an increase of yield to 57 % (entry 4). We then turned to screening the irradiation wavelength using a single lamp.^[13]^ A drop in yield was detected at 440 nm yielding **4 a** in 41 % despite a longer reaction time (entry 5). Irradiation centered at 427 nm led to no significant change (entry 6), whereas a lower conversion of **3 a** and PhEBX (**1 a**) and a lower yield of product **4 a** were observed at 390 nm (entry 7). Finally, 2 lamps at 440 nm and a longer reaction time afforded **4 a** in 63 % yield. However, a more careful monitoring of the reaction over time showed that no further conversion was observed after 8 hours, and the observed small increase is not significant.^[14]^

**Table 1:** Optimization of the direct excitation deoxyalkynylation. 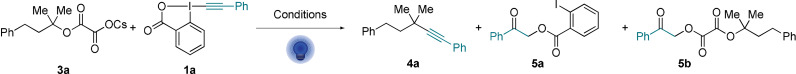

Entry^[a]^	**1 a** (equiv)	reaction time	Light source	λ [nm]	residual **3 a** (equiv)	residual **1 a** (equiv)	Yield **4 a** ^[b]^	Yield **5 a** ^[b]^	Yield **5 b** ^[b]^
1	1.5	18 h	2 Kessil lamps	440	0.25	0.12	50	17	12
2^]^	1.5	24 h	LED strips	460	0.70	0.66	nd	36	23
3^[c]^	1.5	24 h	none	dark	0.75	0.60	nd	45	23
4	2.5	18 h	2 Kessil lamps	440	0.05	0.40	57	50	23
5	2.5	24 h	1 Kessil lamp	440	0.25	0.40	41	43	19
6	2.5	24 h	1 Kessil lamp	427	0.15	0.40	43	25	16
7	2.5	24 h	1 Kessil lamp	390	0.30–0.40	0.60	34	23	17
8	2.5	24 h	2 Kessil lamps	440	0.08	0.40	63	33	18

[a] **3 a** (0.1 mmol) and **1 a** were dissolved in DCM [**3 a**]=0.1 M and irradiated with two lamps (40 W, 440 nm) or LED strips (8 W, 460 nm) at a temperature of 30–35 °C. [b] ^1^H NMR yield was determined using 1 equiv of CH_2_Br_2_ as internal standard. [c] Reaction was run at 50 °C. nd=not detected.

**Scheme 2:**
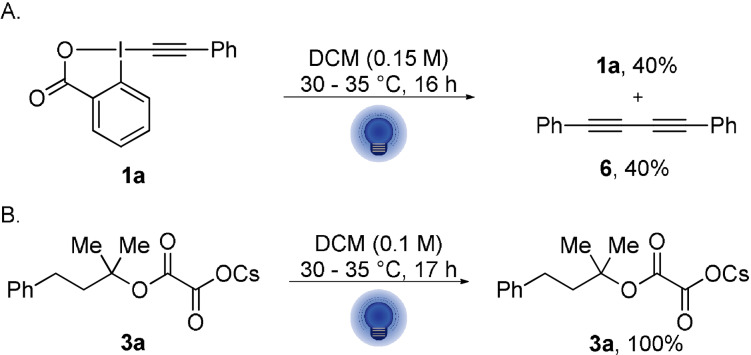
Control experiments supporting the direct photoactivation of PhEBX (**1 a**) and the stability of cesium oxalate **3 a** in absence of **1 a**. The reactions were performed at 0.1 mmol or 0.2 mmol scale and yields were determined by ^1^H NMR by addition of 1 equiv of CH_2_Br_2_ as an internal standard.

The irradiation of a solution of PhEBX (**1 a**) led to non‐negligible degradation (60 % in 16 h) with formation of diyne **6** in 40 % yield (Scheme 2 A), whereas cesium salt **3 a** did not show any degradation when irradiated separately (Scheme 2 B). These experiments confirmed a possible direct light excitation of PhEBX (**1 a**) to PhEBX* (1 a*) independent of the cesium oxalate.

**Scheme 3:**
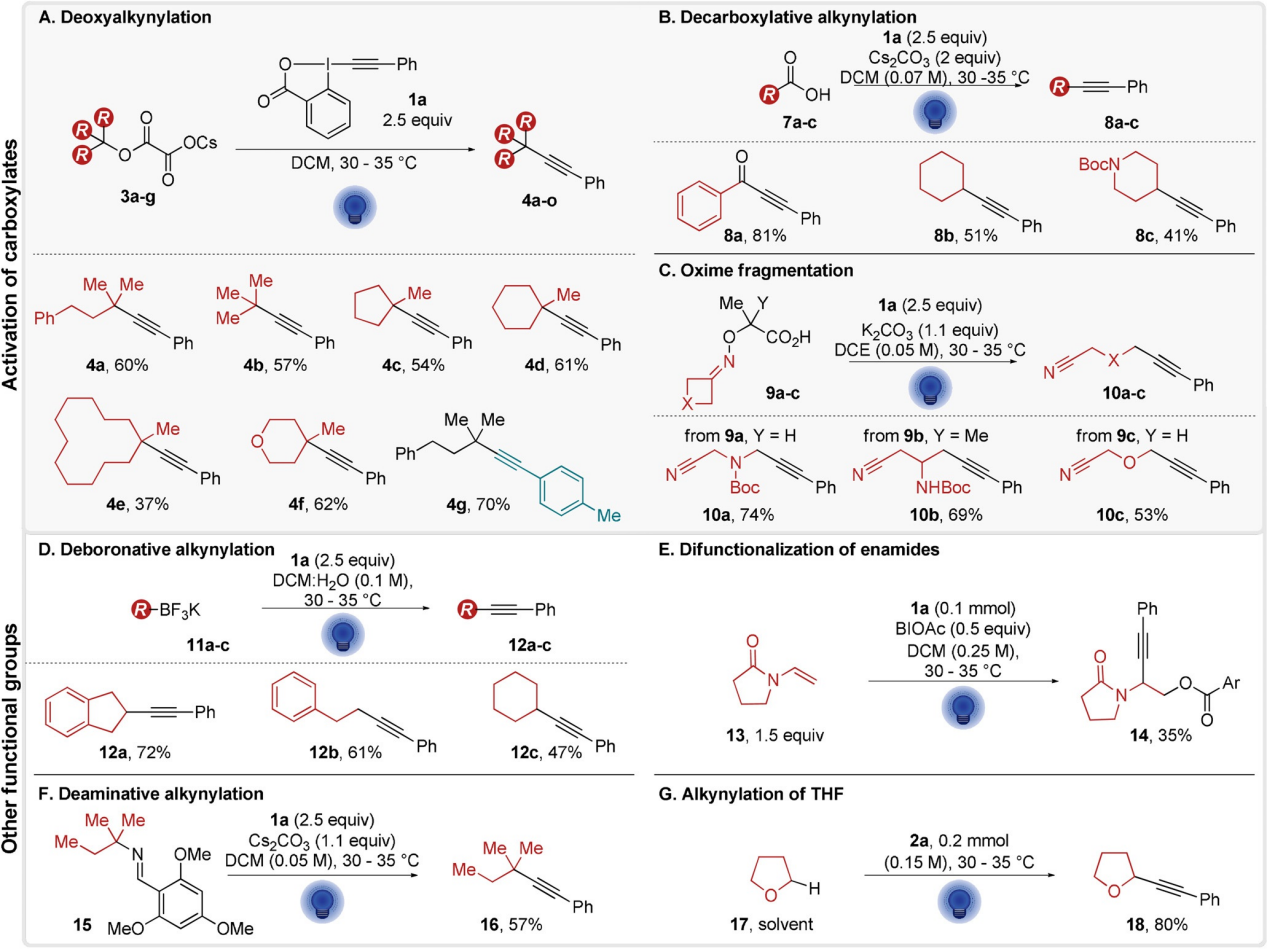
Scope of functional group activation. For A, B, C D, and F: Reactions were performed on 0.3 mmol, E: the reaction was performed on 0.1 mmol scale, the yield was determined by ^1^H NMR using CH_2_Br_2_ (1 equiv) as an internal standard and G: Reaction was performed on 0.2 mmol scale, the yield was determined by ^1^H NMR using CH_2_Br_2_ (1 equiv) as an internal standard.

With the optimized conditions in hand, we first investigated the scope of the alkynylation of cesium oxalates (Scheme 3 A). The model substrate **3 a** could be converted into **4 a** in 60 % isolated yield, while the *tert*‐butanol derivative **3 b** afforded **4 b** with 57 % yield. The same reaction conditions were applied to 5‐, 6‐, and 12‐membered rings **3 c**–**e**, delivering the products **4 c**–**e** in 54, 61 and 37 % yield. Alkynylated heterocyclic **4 f** was obtained in 62 % yield. With *p*TolEBX (**1 b**) as an alkynylating reagent, **4 g** was obtained in 70 % yield. However, the use of halogen‐substituted aryl EBX reagents lead to low yields.^[15]^ We then wondered if this direct excitation strategy could be extended to other decarboxylation processes. We first investigated the decarboxylative alkynylation of carboxylic acids **7** (**7 b^.^**/**7 b^−^** =1.2 V vs. SCE).[^6c^, ^6d^, ^6e^] By simply increasing the reagent and base loading when compared to the photocatalytic procedure, the decarboxylation proceeded in 18 hours affording the desired alkynylation products **8** (Scheme 3 B). This gave access to ynone **8 a** in 81 % yield, as well as aliphatic alkynes **8 b** and **8 c** in 51 % and 41 % yield, respectively.

We then examined the potential of PhEBX* (1 a*) in a decarboxylative oxime fragmentation‐alkynylation (**oxime^.^**/**oxime^−^** ≈1.5 V vs. SCE).^[6f]^ In this case, 2.5 equivalents of PhEBX (**1 a**) instead of the reported 2.0 equivalents gave the desired fragmentation products **10 a**–**c** in 74, 69 and 53 % yield with no other changes to the reaction conditions (Scheme 3 C).

With these results, it appeared that PhEBX* was a potent oxidant activating substrates with potentials up to 1.5 V vs. SCE. It should be therefore able to oxidize other functional groups beside carboxylates. We turned first to the deboronative alkynylation of alkyl trifluoroborate salts **11** (**11 c^.^**/**11 c^−^** =1.5 V vs. SCE).[^6b^, ^16^] In the reported procedure, 0.5 equiv of a hypervalent iodine additive (hydroxybenziodoxolone, BIOH, **19 a**) were required. The direct light activation of PhEBX (**1 a**) in excess alleviated the need for both the photocatalyst and the additive (Scheme 3 D). The alkynylated products **12 a**–**c** were obtained in 72 %, 61 % and 47 % yield, respectively. Furthermore, the direct activation of PhEBX could be used for the difunctionalization of enamide **13** (**enamide^.+^**/**enamide**≈1.3 V vs. SCE) without any change in the reaction conditions, leading to the formation of **14** in 35 % yield (Scheme 3 E).^[6h]^ A new alkynylation reaction was attempted next. Inspired by the deamination‐alkylation of Rovis, we wondered if we could perform a deaminative alkynylation reaction of imine **15** (**imine^.+^**/**imine**≈1.4 vs. SCE).^[11]^ Indeed, the conversion of **15** into **16** occurred in 57 % yield via direct photoexcitation of PhEBX (**1 a**) in the presence of Cs_2_CO_3_ (Scheme 3 F). Finally, the C−H alkynylation of THF (**17**) gave alkyne **18** in 80 % ^1^H NMR yield (Scheme 3 G).

**Scheme 4:**
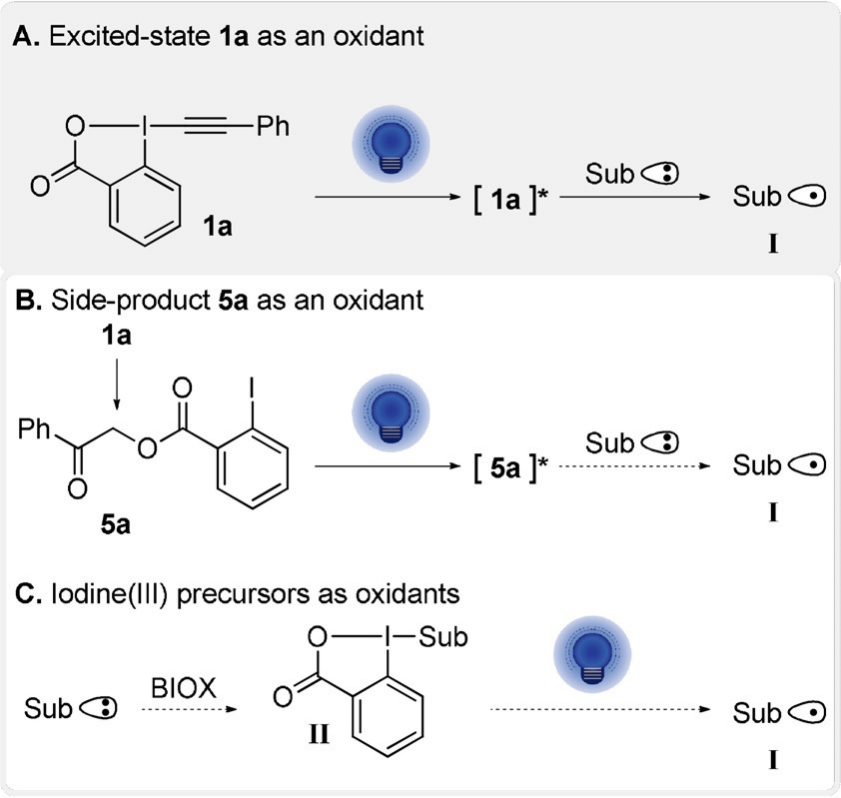
Alternative mechanisms for the oxidative activation of substrates **3**–**8** to give radical **I**.

Having demonstrated that alkynylation was possible for a broad scope of oxidizable substrates, we attempted to gain a better understanding of the transformation. To rationalize our results, we envisaged three main reaction pathways. First, based on the photoinduced degradation of PhEBX (**1 a**, Scheme 2 A) we postulated the direct excitation of PhEBX (**1 a**) to give a strong oxidant 1 a* able to oxidize the substrates to give radical intermediate **I** (Scheme 4 A). Then, based on the broad use of aromatic ketones as photosensitizers,^[17]^ we considered the possibility that the aromatic ketone **5 a** formed during the reaction (Table 1) could act as a photocatalyst and/or a photooxidant (Scheme 4 B). Finally, a residual HIR(III) species BIOX from the synthesis of PhEBX (**1 a**) could be the oxidant (Scheme 4 C). For the special case of decarboxylative procedures, the formation of a covalent adduct **II** between the carboxylate and BIOX, such as BIOH (**19 a**) and BIOAc (**19 b**), could be expected based on literature precedence.[^4^, ^18^] Especially **19 a** could be present in small amounts as impurity in PhEBX (**1 a**), as it is used for its synthesis. Under visible light irradiation, homolytic cleavage of the hypervalent iodine I^III^‐O would form the O‐centered radical, which then undergoes double decarboxylation to give radical **I**.

To gain support for PhEBX* (1 a*) as photooxidant, further experiments were performed (Table 2). Interestingly, when TIPS‐EBX (TIPS=triisopropylsilyl, **1 c**) was used as an alkynylation reagent in absence of photocatalyst, no product was observed and both **1 c** and cesium oxalate **3 a** remained untouched (Table 2, entry 1). **1 c** has been reported to not undergo direct excitation at 400 nm,^[9c]^ but is known to work as a radical trap.^[6d]^ When we performed the reaction with **3 a** and **1 c** with 4CzIPN (**2 a**) as a photocatalyst, we obtained 25 % of alkynylation product **4 h** confirming that **1 c** is able to react with the tertiary radical formed from cesium oxalates, even if the overall reaction is not very efficient (entry 2). This result confirmed that the aryl substituent on PhEBX (**1 a**) was required for the reaction to proceed in absence of photocatalyst, but it still did not allow us to distinguish between our possible reaction pathways.

**Table 2:** Control experiments for the determination of the oxidative species in the deoxyalkynylation. 
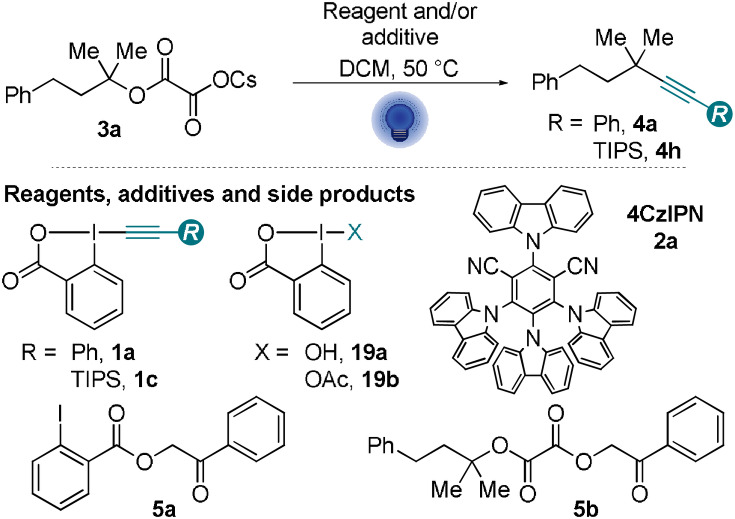

Entry^[a]^	Reagent	Additive (equiv)	Residual **3 a** ^[b]^ [%]	Yield^[b]^ [%]
1	**1 c**	–	100	nd
2	**1 c**	**2 a** (0.05)	30	25
3	**1 c**	**5 a** (0.2)	98	2
4	**–**	**5 a** (1.0)	100	–
5	**1 c**	**19 a** (0.2)	92	5
6	**1 c**	**19 b** (0.2)	92	5
7	**–**	**19 a** (1.5)	90	–
8	**–**	**19 b** (1.5)	100	–
9	**1 c**	**1 a** (0.2)	80	8–13^[c]^

[a] **3 a** (0.1 mmol), **1 c** (1.5 equiv) and the additive were dissolved in DCM [**3 a**]=0.1 M and irradiated with two lamps (40 W, 440 nm) for 18 h. [b] ^1^H NMR yield was determined using 1 equiv of CH_2_Br_2_ as internal standard. [c] Overall yield of deoxy‐alkynylation, **4 a**:**4 h**=1:1.

We then turned to the role of the side product ketone **5 a**. We first explored the possibility of **5 a** acting as photocatalyst and performed the alkynylation with 0.2 equivalents of **5 a** and TIPS‐EBX (**1 c**) (entry 3). Only traces of alkynylation product were observed. In presence of one equivalent of **5 a**, no degradation of cesium oxalate **3 a** or **5 a** was observed under irradiation (entry 4). These results showed that **5 a** was not competent to catalyze the alkynylation process. **5 b** was also subjected to the same control experiments with no alkynylation products detected (see the Supporting Information).^[19]^ We then investigated the potential effect of traces of hypervalent iodine species **19 a** and **19 b**. When the reaction was performed with 0.2 equivalents of either additive, nearly no product formation was obtained with **1 c** (entries 5 and 6). Even with 1.5 equivalents of additive in absence of **1 c**, very little degradation of the starting material was observed upon irradiation (entries 7 and 8). Finally, we performed the alkynylation with 0.2 equivalents of PhEBX (**1 a**) and 1.5 equivalents of TIPS‐EBX (**1 c**). In this case, 20 % conversion of the cesium salt was observed with 8–13 % deoxyalkynylation with phenyl and TIPS alkyne products **4 a** and **4 h** formed in a 1:1 ratio based on ^1^H NMR analysis (entry 9), giving support for **1 a** only acting as photooxidant, whereas both **1 a** and **1 c** can act as radical traps.

**Figure 1:**
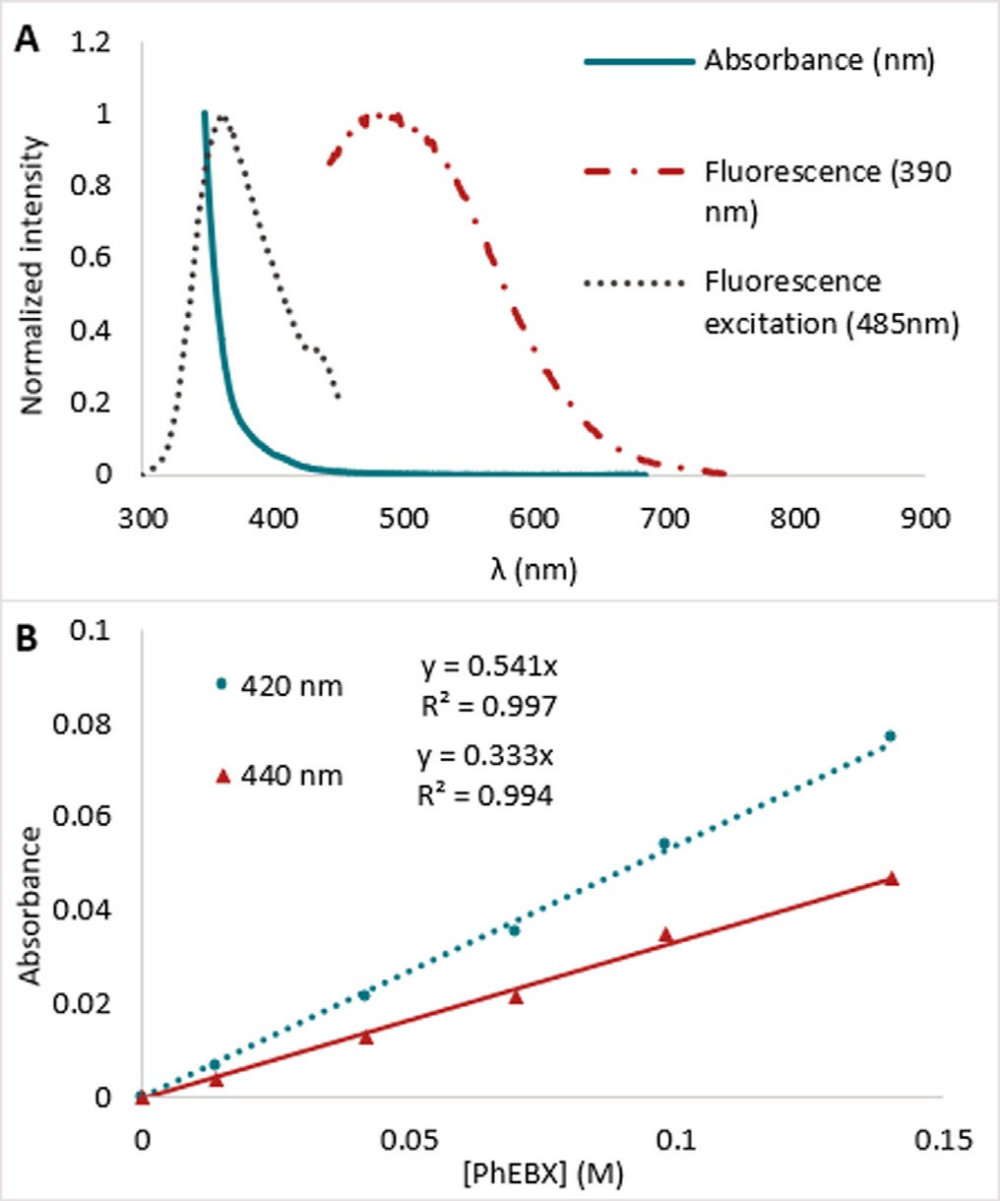
A) Normalized absorption of **1 a** (blue plain line), emission (red dashed line, excitation at *λ*=390 nm, *λ*_max_=485 nm) and fluorescence excitation (gray dotted line, for emission at 485 nm, *λ*_max_=362 nm). B) Beer–Lambert linear regression for 420 nm (blue dotted line) and 440 nm (red plain line). A=*ϵl*[**1 a**], *l*=1 cm), *ϵ*_420 nm_=0.54 L mol^−1^ cm^−1^ and *ϵ*_440 nm_=0.33 L mol^−1^ cm^−1^.

With these results in hand, we turned to UV/Vis absorption and fluorescence spectroscopy to have further support for the photoactivity of PhEBX (**1 a**) under our reaction conditions (Figure 1). We observed absorption until 460 nm (plain blue line) and fluorescence at 485 nm (dashed red line) (Figure 1 A). Fluorescence excitation spectroscopy (dotted grey line) showed that irradiation of **1 a** from 300 nm to 440 nm was responsible for the emission at 485 nm. Specifically, we observed two excitation bands (*λ*_max,1_=380 nm, *λ*_max,2_=430 nm) confirming the possibility of the photoexcitation of **1 a** with a broad band light source with emission centered around 440 nm. To identify the molar extinction coefficient *ϵ* of **1 a**, we performed a Beer–Lambert linear regression at 420 nm and 440 nm providing *ϵ*_420nm_=0.54 L mol^−1^ cm^−1^ and *ϵ*_440nm_=0.33 L mol^−1^ cm^−1^ (Figure 1 B).^[20]^ This is coherent with the weak absorption we observe in the 390 nm–460 nm range, even at high concentrations. These low molar extinction coefficients suggest that the absorption at 420 nm and 440 nm could result from a spin forbidden electronic transition.^[21]^

**Figure 2:**
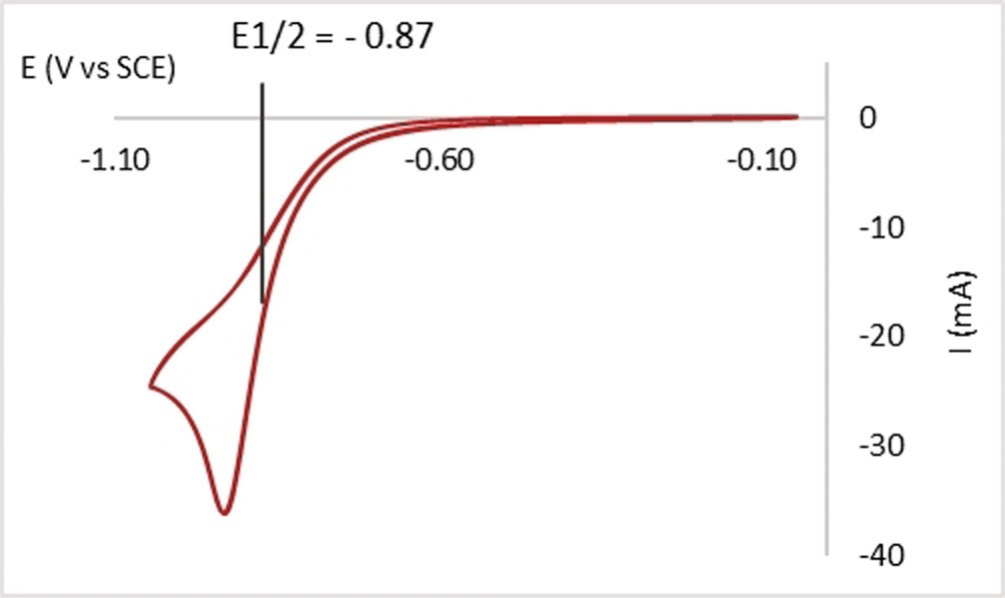
Cyclic voltammogram of **1 a** (50 mV s^−1^, 1.0 μM in MeCN).

Having confirmed that PhEBX (**1 a**) was absorbing under our irradiation conditions, it was important to estimate its strength as an oxidant in the excited state, in particular considering the broad scope of substrates that could be oxidized with 1 a*. First, cyclic voltammetry allowed us to determine the redox potential of the ground state *E*_1/2_(**1 a**/**1 a** ^.−^)=−0.87 V vs. SCE (Figure 2). We could then calculate an estimate of the excited state *E*_1/2_(1 a*/**1 a** ^.−^)=+1.8 V vs. SCE,^[22]^ thus confirming the thermodynamic feasibility of the SET oxidation of the substrates (potentials ranging from +1.3 to +1.5 V, see above).

**Figure 3:**
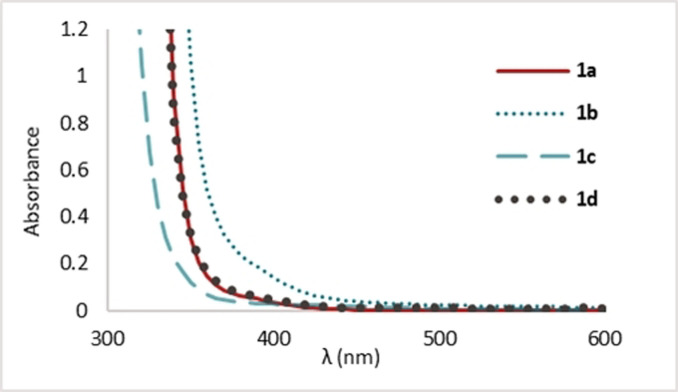
Absorption spectra of **1 a**, **1 b**, **1 c**, and **1 d** at 0.1 M in DMSO.

We then performed UV/Vis of three other EBXs (Figure 3). Interestingly, *p*TolEBX (**1 b**) absorbed more in the region of irradiation than PhEBX (**1 a**). We believe that this could explain the higher yield of deoxyalkynylated **4 g** (70 % instead of 60 % for PhEBX). TIPS‐EBX (**1 c**) absorbed less than the ArEBXs although the absorption band did still tail off into the visible light region. Finally, *m*FPhEBX (**1 d**) absorbed similarly to PhEBX (**1 a**). *m*FPhEBX (**1 d**) did indeed undergo degradation under visible light irradiation. However, the alkynylation was less efficient (30 % by ^1^H NMR).^[15]^

**Scheme 5:**
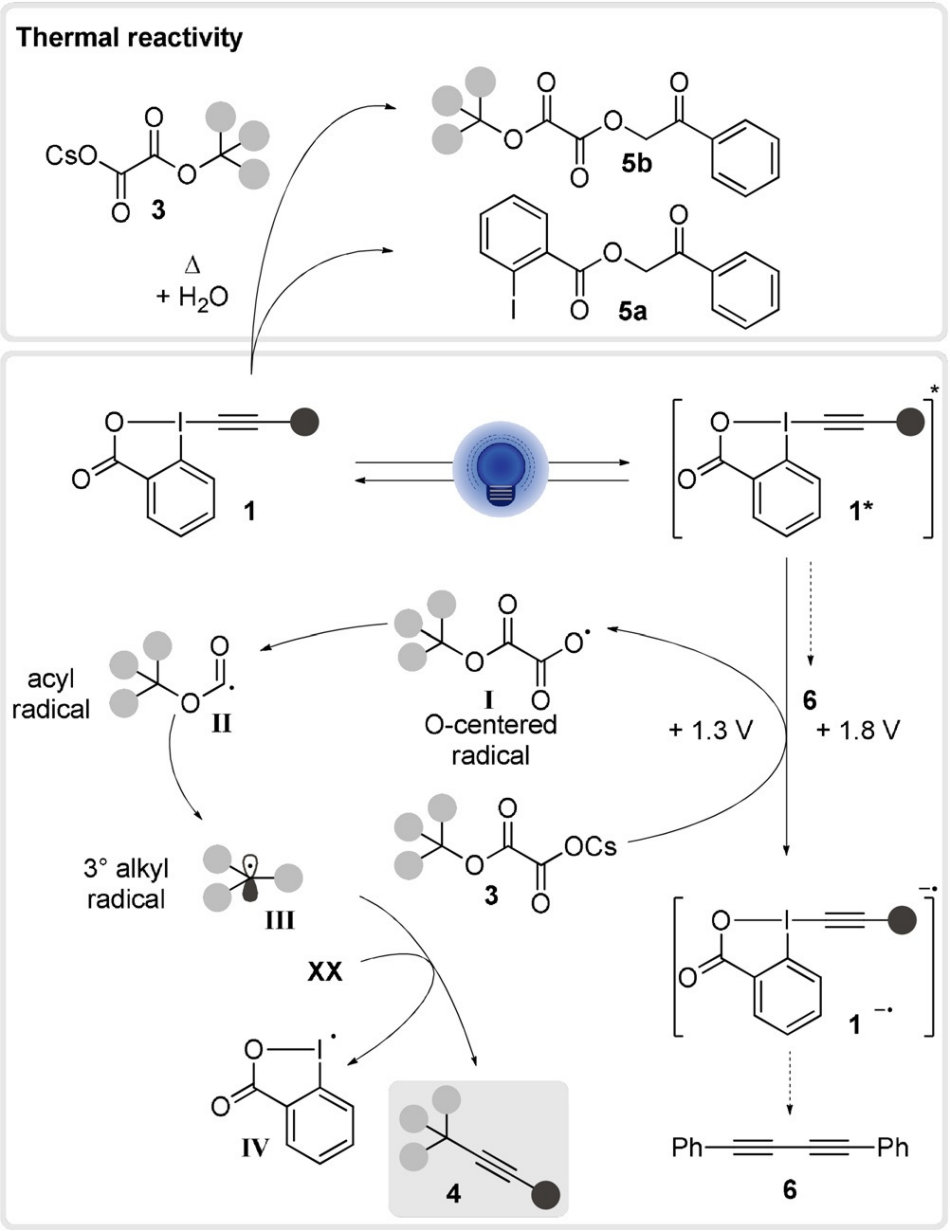
Speculative mechanism.

With the results obtained in our work together with literature precedence,[^4^, ^6^, ^9^, ^16^] we propose the following speculative mechanism for our alkynylation method (Scheme 5). Our experimental data (Figures 1, 2 and 3) suggest that ArEBX (**1**) undergoes direct photoexcitation to generate a highly oxidant excited state 1* (+1.8 V vs. SCE), the latter can then perform a SET oxidation of cesium salt **3** (+1.3 V vs. SCE). The resulting O‐centered radical **I** fragments to the acyl radical **II** and finally the tertiary alkyl radical **III** releasing two molecules of carbon dioxide. The latter can then add to a second EBX reagent **1** affording the final product **4** and the iodanyl radical **IV. IV** (+0.25 V vs. SCE)^[6e]^ would most likely not be capable of performing the oxidation of the cesium salt as it is thermodynamically unfavored. Following the oxidation of oxalate **3**, we suspect that the reduced **1 a^.−^** would be highly unstable and degrade resulting in side products such as the observed 1,4‐diphenylbutadiyne (**6**). Additionally, we cannot exclude the possibility of diyne formation from the excited state 1*. The formation of ketones **5 a** and **5 b** seems to be a background process occurring under thermal conditions. These ketones impact the yield slightly due to the consumption of the starting materials, however, they do not seem to play a role in the reaction mechanism.

**Scheme 6:**
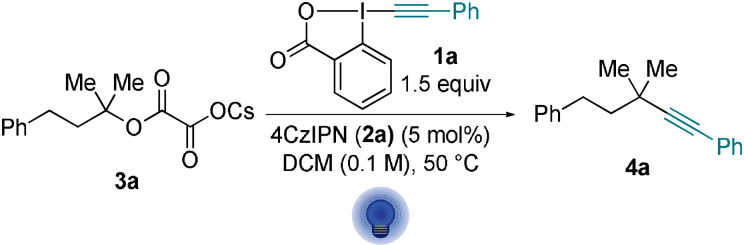
Optimized conditions for the photocatalyzed deoxyakynylation.

Considering the synthetic utility of a photomediated method for accessing alkynylated quaternary centers, we turned back to photocatalysis to improve the yields of our deoxyalkynylation strategy. Using 5 mol % of 4CzIPN (**2 a**) (2 a*/**2 a^−^** =+1.35 V vs. SCE),^[6f]^ under light irradiation at 440 nm with only 1.5 equivalent of PhEBX (**1 a**) in DCM, the desired product (**4 a**) was observed in 75 % NMR yield (94 % based on remaining starting material, Scheme 6).

**Scheme 7:**
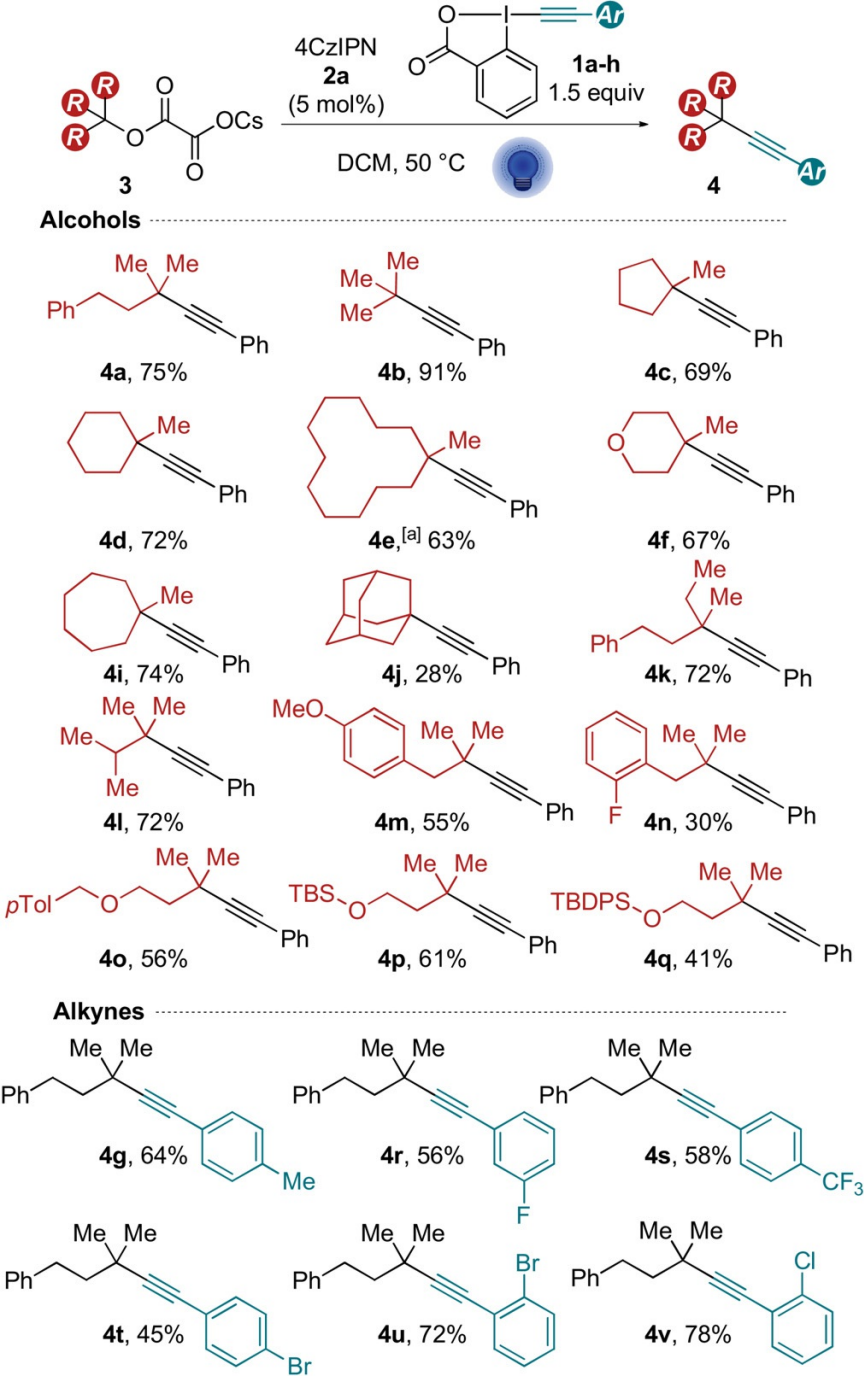
Scope of the photocatalytic deoxyalkynylation. Reactions were performed on 0.3 mmol scale using the corresponding cesium oxalate **3** (1 equiv) and ArEBX **2** (1.5 equiv) with 4CzIPN (**2 a**, 5 mol %) in DCM (0.1 M). [a] Reaction was performed on 0.24 mmol scale with 1.9 equiv of **2 a**.

With the optimized reaction conditions established, we proceeded to explore the scope of cesium oxalates. The model substrate **3 a** afforded the desired alkyne **4 a** in 75 % yield (compared to 60 % for the direct photoexcitation) (Scheme 7). Substrates **3 b**–**f** already examined for the direct photoexcitation gave the products **4 b**–**f** in improved yields (63–91 %). Cycloheptane‐derived alkyne **4 i** was isolated in 74 % yield. The adamantyl alkyne **4 j** was obtained with an expectable drop in yield when considering the unfavored bridged carbon radical.^[23]^ Aliphatic alkynes **4 k** and **4 l** were isolated in 72 % yield. Homobenzylic scaffolds yielded compounds **4 m** and **4 n** in 55 and 30 % yield, respectively. A variety of benzyl and silyl protected alcohols afforded alkynes **4 o**–**q** in up to 61 % yield. Having established the scope of alcohols, we turned to explore different ArEBX reagents. *p*TolEBX afforded the desired product in 64 % yield, which was slightly lower than using the direct excitation approach (70 %). Nevertheless, the catalytic method tolerated a greater panel of reagents than the direct excitation approach: electron‐poor fluorinated reagents afforded the corresponding alkynes **4 r** and **4 s** in 56 % and 58 % yield. Brominated and chlorinated aryl alkynes **4 t**–**v** were obtained in 45 to 78 % yield. Silyl‐ and alkyl‐ EBX reagents however gave the product in only very low yield.

**Scheme 8:**
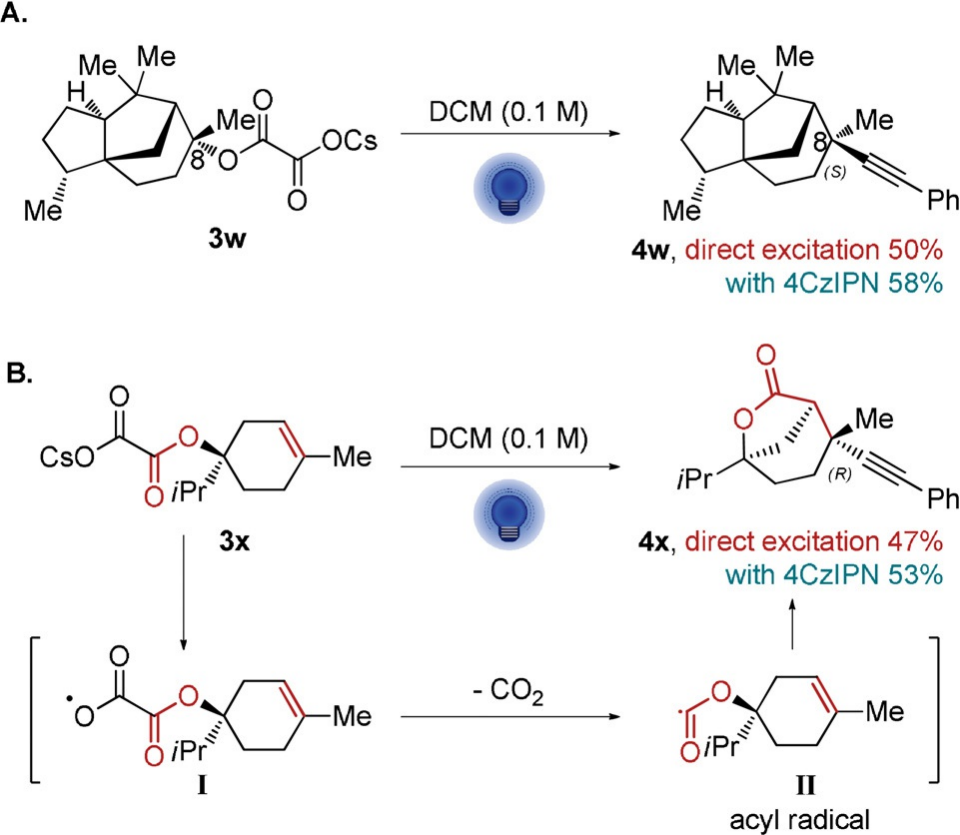
Alkynylation of A. (−)‐Cedrol oxalate **3 w** and B. (−)‐Terpinen‐4‐ol oxalate **3 x**. Reactions were performed on 0.3 mmol scale under blue LED irradiation. Method A: **3 w** or **3 x** (1 equiv), **1 a** (1.5 equiv), **2 a** (5 mol %) in DCM (0.1 M), 50 °C. Method B: **3 w** or **3 x** (1 equiv), **1 a** (2.5 equiv) in DCM (0.1 M).

Finally, we were delighted to see that both the direct excitation and photocatalytic methods could be applied for the diastereoselective deoxyalkynylation of (−)‐cedrol oxalate **3 w** (Scheme 8 A). Both the direct excitation and the photocatalytic methods provided products in over 50 % yield and 20:1 diastereoselectivity based on NMR analysis. NOESY analysis supported that the isomer obtained is of (*S*) configuration at C_8_. Interestingly, when (−)‐terpinen‐4‐ol‐derived oxalate **3 x** was used, a different outcome was observed: the 5‐exo‐trig cyclization of the intermediate acyl radical **II** onto the double bond was observed followed by alkynylation of the formed tertiary carbon radical (Scheme 8 B). Both methods resulted in the formation of product **4 x** in 47 % and 53 % yield, respectively. This indicated that the decarboxylations are stepwise and that the acyl radical **II** formed from the oxalate radical after the release of CO_2_ is long‐lived enough to undergo cyclization before the second decarboxylation. The remotely alkynylated product **4 x** was also obtained in over 20:1 diastereoselectivity.

## Conclusion

We have discovered the direct photoexcitation of aryl EBX reagents in the context of the deoxyalkynylation of cesium oxalates. The broad applicability of the direct excitation of ArEBXs was then exemplified in alkynylation processes requiring a photocatalyst before, including decarboxylative and deboronative alkynylations, the oxyalkynylation of enamides and the C−H alkynylation of THF. The direct excitation of ArEBXs has also enabled a first example of deaminative alkynylation via an aryl imine. In the case of the deoxyalkynylation of oxalates, we also developed a photocatalytic approach using 4CzIPN (**2 a**) as organophotocatalyst and accessed an extended scope of alkynylated quaternary centers.^[24]^ The direct excitation approach discovered in our work results in simplified reaction design and will therefore facilitate the discovery of new alkynylation reactions using ArEBX reagents, as demonstrated in the case of deoxy‐ and deamino‐alkynylation.^[25]^

## Conflict of interest

The authors declare no conflict of interest.

## Supporting information

As a service to our authors and readers, this journal provides supporting information supplied by the authors. Such materials are peer reviewed and may be re‐organized for online delivery, but are not copy‐edited or typeset. Technical support issues arising from supporting information (other than missing files) should be addressed to the authors.

Supporting Information
