# Supplementary material for: Direct Photoexcitation of Ethynylbenziodoxolones: An Alternative to Photocatalysis for Alkynylation Reactions
Source: Angew Chem Int Ed Engl. 2021 Sep 21;60(44):23827–34. doi: 10.1002/anie.202110257 (PMC8596672; doi:10.1002/anie.202110257)

Angewandte Chemie
Eine Zeitschrift der Gesellschaft Deutscher Chemiker

Supporting Information

Direct Photoexcitation of Ethynylbenziodoxolones: An Alternative to Photocatalysis for Alkynylation Reactions**

*Stephanie G. E. Amos[+], Diana Cavalli[+], Franck Le Vaillant, and Jerome Waser**

anie_202110257_sm_miscellaneous_information.pdf

Contents

1. General methods 4

2. Synthesis of starting materials 5

2.1. Synthesis of hypervalent iodine reagents 5

1-Hydroxy-1,2-benziodoxol-3-(1*H*)-one (**19a**) 5

1-Acetoxy-1,2-benziodoxol-3-(1H)-one (**19b**) 5

1-[Phenylethynyl]-1,2-benziodoxol-3(1H)-one (PhEBX, **1a**) 6

1-(p-Tolylethynyl)-1,2-benziodoxol-3(1H)-one (**1b**) 6

Tri*iso*propylsilyl trimethylsilylacetylene (**21c**) 7

1-[(Tri*iso*-propylsilyl)ethynyl]-1,2-benziodoxol-3(1*H*)-one (TIPS-EBX, **1c**) 7

1-[3-Fluorophenylethynyl]-1,2-benziodoxol-3(1H)-one (**1d**) 8

1-[4-Trifluoromethylphenylethynyl]-1,2-benziodoxol-3(1H)-one (**1e**) 9

1-[4-Bromophenylethynyl]-1,2-benziodoxol-3(1H)-one (**1f**) 9

1-[2-Bromophenylethynyl]-1,2-benziodoxol-3(1*H*)-one (**1g**) 10

1-[2-Chlorophenylethynyl]-1,2-benziodoxol-3(1H)-one (**1h**) 11

2.2. Synthesis of the photocatalysts 11

2.3. General procedure A: Synthesis of the photocatalysts 11

2,4,5,6-Tetra(9*H*-carbazol-9-yl)isophthalonitrile (4CzIPN, **2a**) 12

(2r,4s,5r)-2,4,5,6-Tetrakis(3,6-*d*ichloro-9*H*-carbazol-9-yl)isophthalonitrile (4ClCzIPN, **2b**) 12

2.4. Synthesis of tertiary alcohols 13

General procedure B: Synthesis of tertiary alcohols from ketones 13

General procedure C: Synthesis of tertiary alcohols from esters 14

2.5. Synthesis of cesium salts 15

General procedure D: Synthesis of cesium salts from tertiary alcohols 15

Synthetic and characterization data for alkyl ethyl oxalate intermediates **28a-x** and cesium salts **3a-x** 16

2.6. Synthesis of oximes 28

2.7. Synthesis of potassium trifluoroboronates 31

3. Photochemical experimental set-up 32

4. Optimization of the photomediated deoxygenation-alkynylation 33

4.1. Optimization studies method B (Excited state PhEBX **1a**) 33

4.2. Optimization studies of the 4CzIPN photocatalyzed deoxyalkynylation 34

5. Photomediated Alkynyation Reactions: 35

5.1. General Procedures 35

5.1.1. General procedure F: Direct excitation of PhEBX for deoxy-alkynylation 35

5.1.2. General procedure G: Decarboxylative alkynylation 35

5.1.3. General procedure H: Oxime fragmentation-alkynylation 36
5.1.4. General procedure I: Deboronative alkynylation 36
5.1.5. Difunctionalization 36
5.1.6. Deaminative alkynylation 37
5.1.7. HAT 38
5.1.8. General procedure J: 4CzIPN catalyzed deoxyalkynylation 38
5.2. Yields and characterization data 39
5.2.1. Deoxyalkynylated products 39
5.2.2. Decarboxylation alkynylation 53
5.2.3. Oxime fragmentation 54
5.2.4. Deboronative alkynylation 55
6. Mechanistic studies 57
6.1. Monitoring of the reaction by ^{1}H NMR 57
6.2. Side product formation and reaction with TIPS-EBX (1c) monitored by ^{1}H NMR 57
6.3. Synthesis and characterization of **5a**, **5b** and **4h** 59
2-Oxo-2-phenylethyl 2-iodobenzoate (**5a**) 59
2-methyl-4-phenylbutan-2-yl (2-oxo-2-phenylethyl) oxalate (**5b**) 60
(3,3-Dimethyl-5-phenylpent-1-yn-1-yl)triisopropylsilane (**4h**) 61
6.4. Control experiments 61
6.5. UV-Vis absorption and fluorescence studies 62
Absorption and fluorescence studies of PhEBX **1a** and the cesium oxalate **3a** 62
Absorption and Beer-Lambert linear regression at 420 nm and 440 nm of PhEBX (**1a**) 64
6.6. Cyclic voltammetry of PhEBX (**1a**) 65
7. NMR spectra of new compounds 66

1. General methods

All reactions that were carried out in oven dried glassware and under an atmosphere of nitrogen is stated at the start of the reaction conditions. For flash chromatography, distilled technical grade solvents were used. THF, CH_3CN, toluene, Et_2O and CH_2Cl_2 were dried by passage over activated alumina under nitrogen atmosphere (H_2O content < 10 ppm, Karl-Fischer titration). The solvents were degassed by Freeze-Pump-Thaw method when mentioned. All chemicals were purchased from Acros, Aldrich, Fluka, VWR, TCI, Merck and used as such unless stated otherwise. Chromatographic purification was performed as flash chromatography using Macherey-Nagel silica 40-63, 60 Å, using the solvents indicated as eluent with 0.1-0.5 bar pressure. TLC was performed on Merck silica gel 60 F254 TLC glass plates and visualized with UV light and *p*-anisaldehyde stain (EtOH:H_2SO_4:AcOH:*p*-anisaldehyde 135:5:1.5:3.7 V:V:V:V).

^{1}H-NMR spectra were recorded on a Brucker DPX-400 400 MHz spectrometer in CDCl3, acetonitrile-*d*$_3$, DMSO-*d*$_6$ or acetone-*d*$_6$, all signals Are reported in ppm with the internal chloroform signal at 7.26 ppm, the internal acetonitrile signal at 1.94 ppm, the internal methanol signal at 3.30 ppm, the internal DMSO signal at 2.50 ppm or the internal acetone signal at 2.05 ppm as standard. The data is reported as (s = singlet, d = doublet, t= triplet, q = quadruplet, qi = quintet, m = multiplet or unresolved, br = broad signal, app = apparent, coupling constant(s) in Hz, integration, interpretation). ^{13}C-NMR spectra were recorded with ^{1}H-*d*ecoupling on a Brucker DPX-400 100 MHz spectrometer in CDCl3, acetonitrile-*d*$_3$ CD_3OD, DMSO-*d*$_6$ or acetone-*d*$_6$, all signals Are reported in ppm with the internal chloroform signal at 77.0 ppm, the internal acetonitrile signal at 1.3 ppm the internal methanol signal at 49.0 ppm, the internal DMSO signal at 39.5 ppm or the internal acetone signals at 29.84 and 206.26 ppm as standard. Diastereoiomeric ratios has been determined after purification and stereochemistry has been assigned based on ^{1}H NMR analysis.

Infrared spectra were recorded on a JASCO FT-IR B4100 spectrophotometer with an ATR PRO410-S and a ZnSe prisma and is reported in cm^{-1} (w = weak, m = medium, s = strong).

High resolution mass spectrometric measurements were performed by the mass spectrometry service of ISIC at the EPFL on a MICROMASS (ESI) Q-TOF Ultima API.

All photocatalyzed reactions were carried out in oven dried glassware and under inert atmosphere (freeze pump thaw solvent stored on molecular sieves and under argon for maximum one week) unless specified otherwise. They were performed in screw cap dram vials (0.5 – 7,5 mL) which were stuck to a glass plate that was placed on a stirring plate with 2 Kessil lamps (440 nm, 40 W) irradiating from both sides (the hood was free and coated with aluminum foil for personal protection). The distance between the Kessil lamps and the vials was approximatively 10 cm. Long irradiation resulted in temperature increasing up to 50 °C during overnight reactions unless a fan was used in which case the temperature raised to 30-35°C. Photos have been provided.

UV/Vis spectroscopy was performed on an Agilent Cary 60 UV-Vis and steady-state luminescence spectroscopy was recorded on a Varian Cary Eclipse spectrophotometer.

2. Synthesis of starting materials

2.1. Synthesis of hypervalent iodine reagents

The synthesis of reagents **19a-b** and **1a-g** had already been described before.[1,2,3,4,5,6,7,8] Some of the procedures for accessing the ArEBX species have evolved slightly and have been updated with corresponding modifications, the modifications only apply to work-ups and purifications.

1-Hydroxy-1,2-benziodoxol-3-(1*H*)-one (19a)

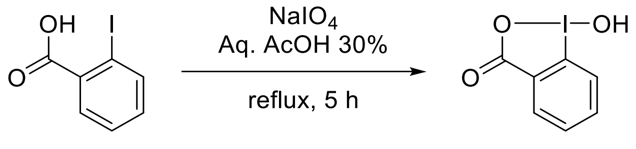

20 **19a**

Following a reported procedure,[1] $NaIO_4$ (40.5 g, 189 mmol, 1.05 equiv) and 2-iodobenzoic acid (**20**, 44.8 g, 180 mmol, 1.0 equiv) were suspended in 30% (v:v) aq. AcOH (350 mL). The mixture was vigorously stirred and refluxed for 5 h. The reaction mixture was then diluted with cold water (250 mL) and allowed to cool to rt, protecting it from light. After 1 h, the crude product was collected by filtration, washed on the filter with ice water (3 x 150 mL) and acetone (3 x 150 mL), and air-dried in the dark overnight to afford 1-Hydroxy-1,2-benziodoxol-3-(1*H*)-one (**19a**, 44.3 g, 168 mmol, 93% yield) as a white solid.

^{1}H NMR (400 MHz, DMSO-d_6) δ 8.02 (dd, J = 7.7, 1.4 Hz, 1H, Ar*H*), 7.97 (m, 1H, Ar*H*), 7.85 (dd, J = 8.2, 0.7 Hz, 1H, Ar*H*), 7.71 (td, J = 7.6, 1.2 Hz, 1H, Ar*H*).

^{13}C NMR (100 MHz, DMSO-d_6) δ 167.7, 134.5, 131.5, 131.1, 130.4, 126.3, 120.4.

Consistent with reported data.[1]

1-Acetoxy-1,2-benziodoxol-3-(1H)-one (19b)

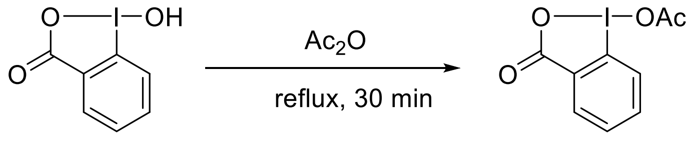

19a **19b**

Following a reported procedure,[9] compound **19a** (3.00 g, 11.3 mmol, 1.00 equiv) was heated in Ac_2O (10 mL) to reflux until the solution turned clear (without suspension, ca. 30 min). The mixture was then left to cool down and white crystals started to form. The crystallization was continued at -18 °C.

[1] Brand, J. P.; Chevalley, C.; Scopelliti, R.; Waser, J. *Chem. – Eur. J.* **2012**, *18*, 5655–5666.
[2] Amos, S. G. E.; Nicolai, S.; Waser, J. *Chem. Sci.* **2020**, *11*, 11274–11279.
[3] Lu, B.; Wu, J.; Yoshikai, N. *J. Am. Chem. Soc.* **2014**, *136*, 11598.
[4] Jia, K.; Zhang, F.; Huang, H.; Chen, Y. *J. Am. Chem. Soc* **2016**, *138*, 1514.
[5] Le Vaillant, F. ; Courant, T. ; Waser, J. *Angew. Chem. Int. Ed.* **2015**, *54*, 11200.
[6] Le Vaillant, F. ; Garreau, M. ; Nicolai, S. ; Gryn'Ova, G. ; Corminboeuf, C. ; Waser, J. *Chem. Sci.* **2018**, *9*, 5883.
[7] Brand, J. P.; Waser, J. *Angew. Chem. Int. Ed.* **2010**, *49*, 7304.
[8] Huang, H.; Zhang, G.; Gong, L.; Zhang, S.; Chen, Y. *J. Am. Chem. Soc.* **2014**, *136*, 2280–2283.
[9] Eisenberger, P.; Gischig, S.; Togni, A. Chem. Eur. J. 2006, 12, 2579

The crystals were then collected and dried overnight under high vacuum to give compound **5a** (3.06 g, 10.0 mmol, 86%).

^{1}H NMR (400 MHz, Chloroform-*d$_3$*) δ 8.25 (dd, 1 H, J = 7.6, 1.4 Hz, Ar*H*), 8.00 (dd, 1 H, J = 8.3, 0.5 Hz, Ar*H*), 7.92 (dt, 1 H, J = 7.0, 1.7 Hz, Ar*H*), 7.71 (td, 1 H, J = 7.6, 0.9 Hz, Ar*H*), 2.25 (s, 3 H, COC*H$_3$*). NMR data correspond to the reported values.[9]

1-[Phenylethynyl]-1,2-benziodoxol-3(1H)-one (PhEBX, 1a)

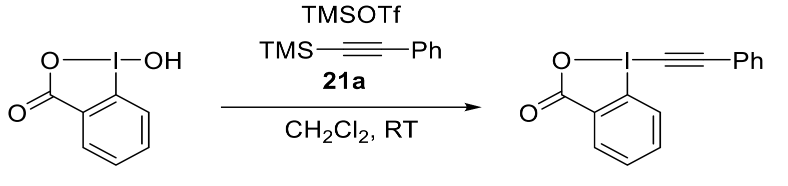

19a **1a**

Following a reported procedure, trimethylsilyltriflate (9.1 mL, 50 mmol, 1.1 equiv) was added dropwise to a suspension of 2-iodosylbenzoic acid (**19a**, 12.1 g, 45.8 mmol, 1.0 equiv) in CH_2Cl_2 (120 mL) at 0 °C. The mixture was stirred for 1 h, followed by the dropwise addition of trimethyl(phenylethynyl)silane (**21a**, 8.8 mL, 50 mmol, 1.1 equiv) (slightly exothermic). The resulting suspension was stirred for 6 h at RT, during this time a white solid was formed. A saturated solution of $NaHCO_3$ (120 mL) was added and the mixture was stirred vigorously for 30 min. The two layers of the mother liquors were separated and the organic layer was washed with sat. $NaHCO_3$ (2x50 mL), dried over $MgSO_4$, filtered and evaporated under reduced pressure. The resulting solid was recrystalized in EtOAc:MeOH (7:3 v:v) (ca. 20 mL). The solution was left to cool to RT then in the freezer ovenight, filtered and dried under high vacuum to afford PhEBX (**1a**, 6.8 g, 25 mmol, 43% yield) as colorless crystals.

Mp (Dec.) 155 – 160 °C.

^{1}H NMR (400 MHz, $CDCl_3$) δ 8.46 (m, 1H, Ar*H*), 8.28 (m, 1H, Ar*H*), 7.80 (m, 2H, Ar*H*), 7.63 (m, 2H, Ar*H*), 7.48 (m, 3H, Ar*H*).

^{13}C NMR (101 MHz, $CDCl_3$) δ 163.9, 134.9, 132.9, 132.5, 131.6, 131.3. 130.8, 128.8, 126.2, 120.5, 116.2, 106.6, 50.2.

Consistent with reported data.[2]

1-(p-Tolylethynyl)-1,2-benziodoxol-3(1H)-one (1b)

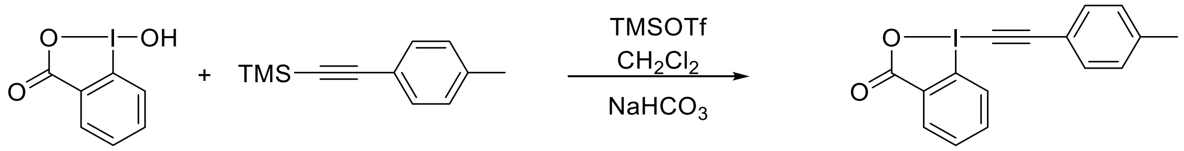

19a **21b** **1b**

Following a reported procedure,[8] trimethylsilyl triflate (1.0 mL, 5.5 mmol, 1.1 equiv) was added to a suspension of 2-iodosylbenzoic acid (**19a**) (1.32 g, 5.00 mmol, 1.00 equiv) in CH_2Cl_2 (15 mL) at room temperature. The resulting suspension was stirred for 3 h, followed by the drop wise addition of trimethyl(p-tolylethynyl)silane (**21b**) (1.04 g, 5.50 mmol, 1.10 equiv). The resulting suspension was stirred for 6 h at room temperature. A saturated solution of $NaHCO_3$ (20 mL) was then added and the mixture was stirred vigorously for 30 minutes, the two layers were separated and the organic layer

was washed with saturated solution of $NaHCO_3$ (20 mL), dried over Na_2SO_4, filtered and evaporated under reduced pressure. The resulting solid was recrystallized from EtOAc:MeOH 7:3 (ca 20 mL). The mixture was cooled down, filtered and dried under high vacuum to afford **1b** (0.620 g, 1.71 mmol, 45%) as a white crystals.

^{1}H NMR (400 MHz, $CDCl_3$) δ 8.43 (dd, *J* = 6.1, 2.9 Hz, 1H, Ar*H*), 8.30– 8.14 (m, 1H, Ar*H*), 7.77 (dd, *J* = 6.9, 3.1 Hz, 2H, Ar*H*), 7.50 (d, *J* = 7.8 Hz, 2H, Ar*H*), 7.25 (d, *J* = 7.6 Hz, 2H, Ar*H*), 2.43 (s, 3H, ArCH_3).

^{13}C NMR (100 MHz, CDCl3): δ 166.6, 141.5, 134.9, 132.8, 132.5, 131.6, 131.3, 129.5, 126.2, 117.4, 116.2, 107.25, 49.1, 21.7. The characterization data corresponded to the reported values.[8]

Tri*iso*propylsilyl trimethylsilylacetylene (**21c**)

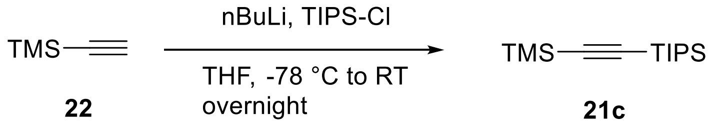

22 **21c**

Following a reported procedure,[10] *n*-butyllithium (2.5 M in hexanes, 28 mL, 70 mmol, 0.98 equiv) was added dropwise to a stirred solution of ethynyltrimethylsilane (**22**, 7.0 g, 71 mmol, 1.0 equiv) in THF (100 mL) at -78 °C. The mixture was warmed to 0 °C and stirred for 5 min. The mixture was then cooled back to -78 °C and chlorotri*iso*propylsilane (15 mL, 71 mmol, 1.0 equiv) was added dropwise. The mixture was then allowed to warm to room temperature and stirred overnight. A saturated solution of ammonium chloride (100 mL) was added, and the reaction mixture was extracted with diethyl ether (2 x 100 mL). The combined organic layers were washed with water and brine, then dried over MgSO4, filtered and concentrated under reduced pressure to obtain a colorless liquid which was further purified by filtration on silica eluting with pentane (500 mL) to yield **21g** (16 g, 64 mmol, 90% yield) as a colorless liquid.

^{1}H NMR (400 MHz, Chloroform-*d*) *δ* 1.08 (m, 21H, TIPS), 0.18 (s, 9H, TMS).

Consistent with reported data.[10]

1-[(Tri*iso*-propylsilyl)ethynyl]-1,2-benziodoxol-3(1*H*)-one (TIPS-EBX, **1c**)

This compound can also be accessed in one pot from commercially available *o*-iodobenzoic acid and the free TIPS alkyne, however in the context of this study it was synthesized in the 2 step fashion.[11]

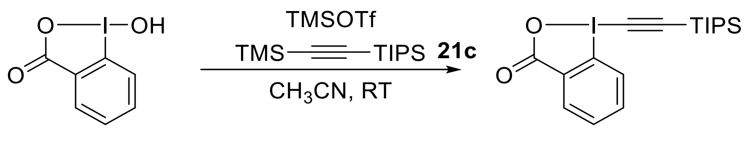

19a **1c**

Following a reported procedure,[7] 2-iodosylbenzoic acid (**19a**, 8.0 g, 30 mmol, 1.0 equiv) was charged in an oven-*d*ried round-bottomed 250 mL flask equipped with a magnetic stirrer. The solid was placed under a nitrogen atmosphere and anhydrous acetonitrile (100 mL) was added. The mixture was cooled to 0 °C. Trimethylsilyltriflate (6.0 mL, 33 mmol, 1.1 equiv) was added dropwise. After 15 min,

---

[10] Helal, C. J.; Magriotis, P. A.; Corey, E. J. *J. Am. Chem. Soc.* **1996**, *118*, 10938.
[11] Hari, D. P.; Caramenti, P.; Schouwey, L.; Chang, M.; Nicolai, S.; Bachert, D.; Wright, T.; Orella, C.; Waser, J. *Org. Process Res. Dev.* **2020**, *24*, 106–110.

(trimethylsilyl)(tri*iso*propylsilyl)acetylene (**21c**, 8.5 g, 33 mmol, 1.1 equiv) was added dropwise. After 30 min, the suspension became an orange solution. Pyridine (2.7 mL, 33 mmol, 1.1 equiv) was added dropwise. After 15 min, the reaction mixture was transferred in a one-neck 500 mL flask and concentrated under vacuum to afford a yellow solid. The solid was dissolved in CH_2Cl_2 (100 mL) and transferred in a 500 mL separatory funnel. The organic layer was washed with a 1 M HCl solution (50 mL) and the aqueous layer was extracted with CH_2Cl_2 (100 mL). The organic layers were combined, washed with a saturated solution of $NaHCO_3$ (2 x 100 mL), dried over $MgSO_4$, filtered and the solvent was evaporated under reduced pressure. Recrystallization from acetonitrile (40 mL) afforded TIPS-EBX (**1c**, 9.2 g, 21.5 mmol, 71% yield) as colorless crystals.

Mp (Dec.) 170-176 °C.

^{1}H NMR (400 MHz, Chloroform-*d*) *δ* 8.44 (m, 1H, Ar*H*), 8.29 (m, 1H, Ar*H*), 7.77 (m, 2H, Ar*H*), 1.16 (m, 21H, TIPS).

^{13}C NMR (100 MHz, Chloroform-*d*) δ 166.4, 134.6, 132.3, 131.4, 131.4, 126.1, 115.6, 114.1, 64.6, 18.4, 11.1.

IR ν 2943 (m), 2865 (m), 1716 (m), 1618 (m), 1604 (s), 1584 (m), 1557 (m), 1465 (m), 1439 (w), 1349 (m), 1291 (m), 1270 (w), 1244 (m), 1140 (m), 1016 (m), 999 (m), 883 (m), 833 (m), 742 (m), 702 (s), 636 (m).

Consistent with reported data.[7]

1-[3-Fluorophenylethynyl]-1,2-benziodoxol-3(1H)-one (**1d**)

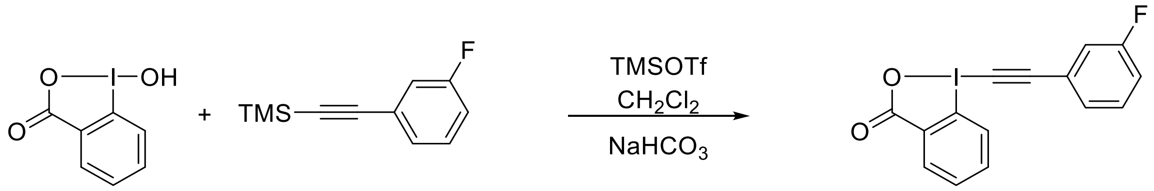

19a **21d** **1d**

Following a slightly modified reported procedure,[6] trimethylsilyl triflate (0.44 mL, 2.5 mmol, 1.1 equiv) was added to a suspension of 2-iodosylbenzoic acid (**19a**, 0.589 g, 2.23 mmol, 1.00 equiv) in CH_2Cl_2 (6.8 mL) at RT The resulting suspension was stirred for 1 h, followed by the dropwise addition of ((3-fluorophenyl)ethynyl)trimethylsilane (**21d**, 0.50 mL, 2.5 mmol, 1.1 equiv). The resulting suspension was stirred for 6 h at RT. A saturated solution of $NaHCO_3$ (10 mL) was then added and the mixture was stirred vigorously for 30 minutes, resulting in a suspension. The mixture was diluted with chloroform (10 mL), water (5 mL) and MeOH (ca. 0.5 mL) resulting in two clear layers. The two layers were separated, and the organic layer was washed with sat. $NaHCO_3$ (7 mL), dried over Na_2SO_4, filtered, and evaporated under reduced pressure. The resulting solid was recrystallized in EtOAc:MeOH (7:3 v:v) (ca. 20 mL). The solution was left to cool to RT then was placed in the freezer (-20 °C) overnight. The crystals were filtered and washed with Et_2O to afford **1d** (787 mg, 2.15 mmol, 43% yield) as colorless crystals.

^{1}H NMR (400 MHz, DMSO-d_6) δ 8.33 (dd, *J* = 8.2, 0.8 Hz, 1H, Ar*H*), 8.13 (dd, *J* = 7.4, 1.7 Hz, 1H, Ar*H*), 7.91 (ddd, *J* = 8.2, 7.2, 1.7 Hz, 1H, Ar*H*), 7.81 (td, *J* = 7.3, 0.9 Hz, 1H, Ar*H*), 7.64 – 7.59 (m, 1H, Ar*H*), 7.58 – 7.53 (m, 2H, Ar*H*), 7.47 – 7.37 (m, 1H, Ar*H*).

^{13}C NMR (101 MHz, DMSO-d_6)[12] 166.3, 161.8 (d, J = 245.6 Hz), 135.3, 131.9, 131.3, 131.2 (d, J = 8.7 Hz), 129.0 (d, J = 2.9 Hz), 127.7, 122.4 (d, J = 9.6 Hz), 119.2 (d, J = 23.4 Hz), 118.1 (d, J = 21.1 Hz), 116.4, 102.5 (d, J = 3.3 Hz), 53.8.

^{19}F NMR (376 MHz, DMSO-d_6) δ -111.7.

Consistent with reported data.[5]

1-[4-Trifluoromethylphenylethynyl]-1,2-benziodoxol-3(1H)-one (1e)

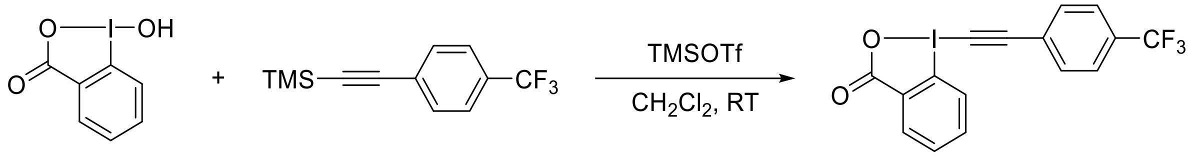

19a **21e** **1e**

Following a reported procedure,[3] trimethylsilyl triflate (1.0 mL, 5.5 mmol, 1.1 equiv) was added to a suspension of 2-iodosylbenzoic acid (**19a**, 1.3 g, 5.0 mmol, 1.0 equiv) in CH_2Cl_2 (15 mL) at RT The resulting suspension was stirred for 1 h, followed by the dropwise addition of trimethyl((4-(trifluoromethyl)phenyl)ethynyl)silane (**21e**, 1.3 mL, 5.5 mmol, 1.1 equiv), which was dissolved in CH_2Cl_2 (1 mL). The resulting suspension was stirred for 6 h at RT A saturated solution of $NaHCO_3$ (20 mL) was then added and the mixture was stirred vigorously for 30 min, the two layers were separated and the organic layer was washed with sat. $NaHCO_3$ (20 mL), dried over $MgSO_4$, filtered and evaporated under reduced pressure. The resulting solid was boiled in CH_3CN (20 mL). The mixture was cooled down, filtered and dried under high vacuum to afford **1e** (1.3 g, 3.2 mmol, 64% yield) as a pale yellow solid.

^{1}H NMR (400 MHz, $CDCl_3$) δ 8.46 – 8.38 (m, 1H, ArH), 8.28 – 8.19 (m, 1H, ArH), 7.84 – 7.74 (m, 2H, ArH), 7.74 – 7.65 (m, 4H, ArH).

^{13}C NMR (101 MHz, $CDCl_3$) δ 166.6, 135.0, 133.0, 132.6, 132.2 (q, J = 33.0 Hz), 131.7, 131.2, 126.3, 125.7 (q, J = 3.6 Hz), 124.4, 123.4 (q, J = 272.6 Hz), 116.1, 104.2, 53.7.

Consistent with reported data.[3]

1-[4-Bromophenylethynyl]-1,2-benziodoxol-3(1H)-one (1f)

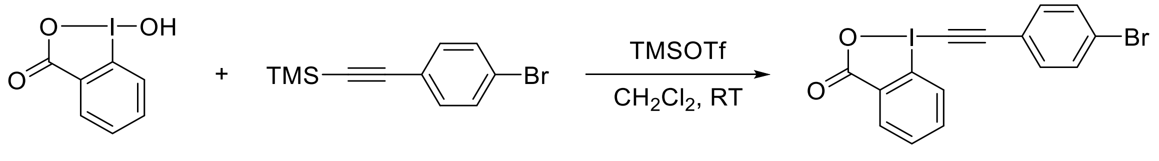

19a **21f** **1f**

Following a reported procedure,[4] trimethylsilyl triflate (1.0 mL, 5.5 mmol, 1.1 equiv) was added to a suspension of 2-iodosylbenzoic acid (**19a**, 1.3 g, 5.0 mmol, 1.0 equiv) in CH_2Cl_2 (15 mL) at RT The resulting suspension was stirred for 1 h, followed by the dropwise addition of ((4-bromophenyl)ethynyl)trimethylsilane (**21f**, 1.2 g, 5.5 mmol, 1.1 equiv), which was dissolved in CH_2Cl_2

[12] One carbon is not resolved.

(1 mL). The resulting suspension was stirred for 6 h at RT A saturated solution of $NaHCO_3$ (20 mL) was then added and the mixture was stirred vigorously for 30 min, the two layers were separated and the organic layer was washed with sat. $NaHCO_3$ (20 mL), dried over $MgSO_4$, filtered and evaporated under reduced pressure. The resulting solid was boiled in CH_3CN (20 mL). The mixture was cooled down, filtered and dried under high vacuum to afford **1f** (1.4 g, 3.3 mmol, 66% yield) as a pale yellow solid.

Mp 158-163 °C (decomposition).

^{1}H NMR (400 MHz, $CDCl_3$) δ 8.51 – 8.30 (m, 1H, Ar*H*), 8.30 – 8.13 (m, 1H, Ar*H*), 7.84 – 7.72 (m, 2H, Ar*H*), 7.58 (d, 2H, *J* = 8.5 Hz, Ar*H*), 7.46 (d, 2 H, *J* = 8.5 Hz, Ar*H*).

^{13}C NMR (101 MHz, $CDCl_3$) δ 166.6, 135.1, 134.3, 132.7, 132.3, 131.9, 131.4, 126.3, 125.7, 119.6, 116.3, 105.4, 52.1.

Consistent with reported data.[4]

1-[2-Bromophenylethynyl]-1,2-benziodoxol-3(1*H*)-one (**1g**)

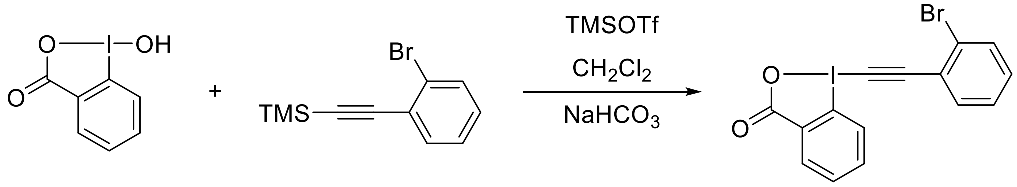

19a **21g** **1g**

Following a slightly modified reported procedure,[5] trimethylsilyl triflate (0.42 mL, 2.4 mmol, 1.1 equiv) was added to a suspension of 2-iodosylbenzoic acid (**19a**, 0.562 g, 2.13 mmol, 1.00 equiv) in CH_2Cl_2 (6 mL) at RT The resulting suspension was stirred for 1 h, followed by the drop wise addition of ((2-bromophenyl)ethynyl)trimethylsilane (**21g**, 0.50 mL, 2.4 mmol, 1.1 equiv). The resulting suspension was stirred for 6 h at RT. A saturated solution of $NaHCO_3$ (10 mL) was then added and the mixture was stirred vigorously for 1 h resulting in a persistent emulsion/suspension. The mixture was diluted with $CHCl_3$ (10 mL), water (5 mL) and MeOH (ca. 2 mL) to afford 2 distinct layers. The two layers were separated, and the organic layer was washed with sat. $NaHCO_3$ (5 mL), dried over Na_2SO_4, filtered, and evaporated under reduced pressure. The resulting solid was recrystallized in EtOAc:MeOH (7:3 v:v) (ca. 20 mL). The solution was left to cool to RT then was placed in the freezer (-20 °C) overnight. The crystals were filtered and washed with Et_2O afford **1g** (1.50 g, 3.51 mmol, 70% yield) as colorless crystals.

^{1}H NMR (400 MHz, $CDCl_3$) δ 8.44 (td, *J* = 7.3, 2.1 Hz, 2 H, Ar*H*), 7.84 – 7.74 (m, 2 H, Ar*H*), 7.68 (d, *J* = 1.1 Hz, 1 H, Ar*H*), 7.61 (dd, *J* = 7.6, 1.7 Hz, 1 H,*ArH*), 7.36 (m, 2 H, Ar*H*).

^{13}C NMR (101 MHz, $CDCl_3$)[7] δ 166.6, 135.2, 134.7, 133.0, 132.7 , 131.8, 131.3, 127.6, 126.8, 126.4, 123.2, 116.5, 104.3, 55.4.

Consistent with reported data.[5]

1-[2-Chlorophenylethynyl]-1,2-benziodoxol-3(1H)-one (**1h**)

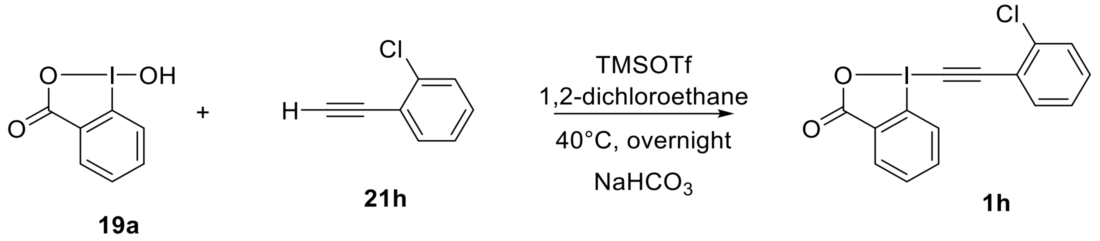

Following a slightly modified reported procedure,[6] trimethylsilyl triflate (0.40 mL, 2.2 mmol, 1.2 equiv) was added to a suspension of 2-iodosylbenzoic acid (**19a**, 0.548 g, 2.08 mmol, 1.1 equiv) in DCE (5.8 mL) at RT The resulting suspension was stirred for 1 h, followed by the drop wise addition of (2-chlorophenyl)acetylene (**21h**, 0.26 mL, 0.19 mmol, 1.0 equiv). The resulting suspension was stirred for 15 h at 40 °C A saturated solution of $NaHCO_3$ (20 mL) was then added and the mixture was stirred vigorously for 30 minutes resulting in a persistent emulsion/suspension. Water (5 mL) was added, followed by chloroform (15 mL) and MeOH (ca. 0.5 mL) resulting in 2 clear layers. The two layers were seperated and the organic layer was washed with sat. $NaHCO_3$ (5 mL), dried over Na_2SO_4, filtered and evaporated under reduced pressure. The resulting solid was recrystallized from EtOAc:MeOH (7:3 v:v, ca. 10 mL). The mixture was cooled down overnight in the freezer (-20 °C), filtered and washed with Et_2O to afford **1h** (0.217 g, 0.567 mmol, 30% yield) as a white crystalline solid.

^{1}H NMR (400 MHz, $CDCl_3$) δ 8.46 – 8.38 (m, 2H, Ar*H*), 7.84 – 7.73 (m, 2H, Ar*H*), 7.62 (dd, *J* = 7.6, 1.7 Hz, 1H, Ar*H*), 7.50 (dt, *J* = 8.2, 1.2 Hz, 1H, Ar*H*), 7.46 – 7.37 (m, 1H, Ar*H*), 7.33 (td, *J* = 7.6, 1.3 Hz, 1H, Ar*H*).

^{13}C NMR (101 MHz, $CDCl_3$) δ 166.6, 137.2, 135.2, 134.5, 132.7, 131.8, 131.7, 131.3, 129.9, 127.0, 126.7, 121.0, 116.4, 102.7, 56.0.

Consistent with reported data[13]

2.2. Synthesis of the photocatalysts

2.3. General procedure A: Synthesis of the photocatalysts

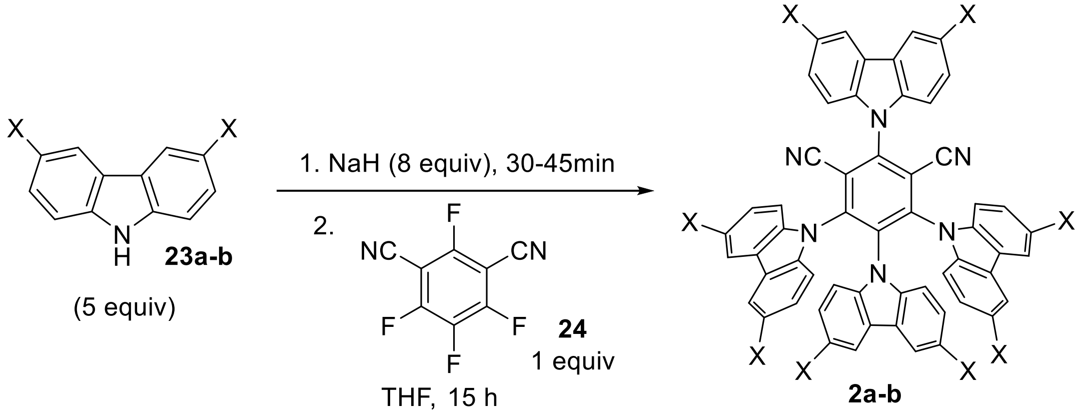

[13] Li, M.; Li, W.; Lin, C.-D.; Wang, J.-H.; Wen, L.-R. *J. Org. Chem.* **2019**, *84* (11), 6904–6915.

Sodium hydride (60% suspension in mineral oil, 8.0 equiv) was added slowly to a stirred solution of substituted-carbazole **23** (5.0 equiv) in dry THF (0.05 M) under a nitrogen atmosphere at RT After 30 min, 2,4,5,6-tetrafluoroisophthalonitrile **24** (1.0 mmol, 1.0 equiv) was added. After stirring at RT for 15 h, 2 mL water was added to the reaction mixture to quench the excess of NaH. The resulting mixture was then concentrated under reduced pressure. The crude product was purified by recrystallization from hexane:CH_2Cl_2 then filtered. The brown liquid filtrate was concentrated and recrystallized as before. The combined solids were then purified by column chromatography on silica gel with CH_2Cl_2:Hexane.

2,4,5,6-Tetra(9*H*-carbazol-9-yl)isophthalonitrile (4CzIPN, 2a)

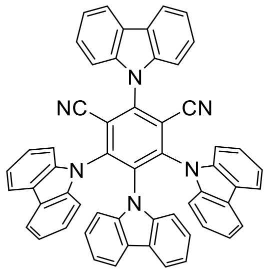

Following *general procedure A* and starting from 9H-carbazole **23a** (X = H, 1.67 g, 10.0 mmol, 5.00 equiv), sodium hydride (0.60 g, 15 mmol, 7.5 equiv) and 2,4,5,6-tetrafluoroisophthalonitrile **24** (0.40 g, 2.0 mmol) in 40 mL of THF. Recrystallization (Hexanes:CH_2Cl_2 (1:1, 90 mL)) afforded the crude product as a yellow powder. Column chromatography afforded 2,4,5,6-tetra(9H-carbazol-9-yl)isophthalonitrile (**2a**) as a bright yellow crystalline solid (1.14 g, 1.45 mmol, 73 % yield).

Rf (Hexane;CH_2Cl_2 1:1) = 0.29. (yellow spot on TLC).

^{1}H NMR (400 MHz, $CDCl_3$) δ 8.2 (d, *J* = 7.7 Hz, 2H, Ar*H*), 7.8 – 7.6 (m, 8H, Ar*H*), 7.5 (ddd, *J* = 8.0, 6.6, 1.6 Hz, 2H, Ar*H*), 7.3 (d, *J* = 7.5 Hz, 2H, Ar*H*), 7.2 (dd, *J* = 8.4, 1.5 Hz, 4H, Ar*H*), 7.2 – 7.0 (m, 8H, Ar*H*), 6.8 (t, *J* = 7.8 Hz, 4H, Ar*H*), 6.6 (td, *J* = 7.6, 1.2 Hz, 2H, Ar*H*).

^{13}C NMR (101 MHz, $CDCl_3$) δ 145.2, 144.6, 140.0, 138.2, 136.9, 134.7, 127.0, 125.8, 124.9, 124.7, 124.5, 123.8, 122.4, 121.9, 121.4, 121.0, 120.4, 119.6, 116.3, 111.6, 109.9, 109.5, 109.4.

^{1}H NMR shift in $CDCl_3$ are consistent with reported data.[14]

(2r,4s,5r)-2,4,5,6-Tetrakis(3,6-*d*ichloro-9*H*-carbazol-9-yl)isophthalonitrile (4ClCzIPN, 2b)

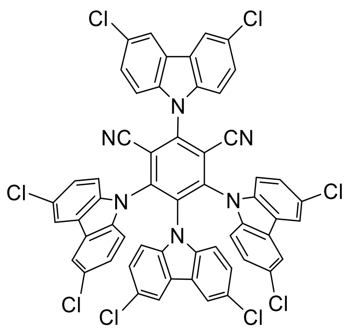

Following *general procedure A* and starting from 3,6-*d*ichloro-9H-carbazole **23b** (1.96 g, 6.00 mmol, 6.0 equiv), sodium hydride (0.320 g, 8.00 mmol, 8.0 equiv) and 2,4,5,6-tetrafluoroisophthalonitrile **24** (200 mg, 1.00 mmol) in 20 mL of THF. Recrystallization (Hexanes:CH_2Cl_2 (1:2, 80 mL)) gave 900 mg of yellow powder, then second recrystallization gave 325 mg of brown powder. Column chromatography of the combined solid afforded (2r,4s,5r)-2,4,5,6-tetrakis(3,6-*d*ichloro-9H-carbazol-9-yl)isophthalonitrile (**2b**) as a bright yellow crystalline solid (830 mg, 0.780 mmol, 87 % yield).

Rf (Hexane:CH_2Cl_2 1:1): 0.25. (yellow spot on TLC).

^{1}H NMR (400 MHz, DMSO-*d*$_6$) δ 8.60 (d, *J* = 2.1 Hz, 2H, Ar*H*), 8.15 (d, *J* = 2.1 Hz, 4H, Ar*H*), 8.08 (d, *J* = 8.8 Hz, 2H, Ar*H*), 7.87 (dd, *J* = 8.8, 2.1 Hz, 2H, Ar*H*), 7.80 (d, *J* = 2.2 Hz, 2H, Ar*H*), 7.69 (d, *J* = 8.8 Hz, 4H, Ar*H*), 7.46 (d, *J* = 8.8 Hz, 2H, Ar*H*), 7.32 (dd, *J* = 8.8, 2.2 Hz, 4H, Ar*H*), 6.93 (dd, *J* = 8.8, 2.2 Hz, 2H, Ar*H*).

^{13}C NMR (101 MHz, DMSO-*d*$_6$) δ 145.0, 144.5, 138.5, 137.4, 136.5, 135.8, 134.5, 127.8, 127.0, 126.4, 125.7, 125.3, 124.2, 123.8, 123.3, 121.6, 120.9, 120.3, 116.8, 112.6, 112.5, 112.3, 111.7.

[14] Uoyama, H.; Goushi, K.; Shizu, K.; Nomura, H.; Adachi, C. *Nature* **2012**, *492*, 234.

^1H NMR shift in $CDCl_3$ are consistent with reported data.[6]

2.4. Synthesis of tertiary alcohols

Alcohols for substrates **3a-d**, **3k-m**, **3p**, **3w** and **3x** were purchased from commercial sources (Sigma-Aldrich, Acros, TCI, abcr) and used directly without prior purification.

General procedure B: Synthesis of tertiary alcohols from ketones

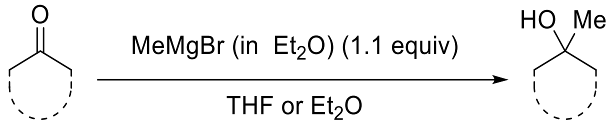

25e-f **26e-f**

An oven dried two-necked flask, equipped with a magnetic stirrer, was charged with the ketone **25e-f** (1.0 equiv) and dissolved in anhydrous THF or Et_2O (0.2 M). The reaction was cooled to 0 °C with an ice bath. The methylmagnesium bromide solution (3.0 M in Et_2O) was diluted to 1 M and added dropwise to the cooled solution *via* a dropping funnel. The reaction was stirred at room temperature overnight (15 to 18 h) at this time the reaction was quenched with sat. aq. NH_4Cl, followed by the addition of water and EtOAc. The layers were separated, the aqueous layer was extracted 3 times with EtOAc then the combined organic layers were washed with sat. aq. NaCl. The organic layer was then dried on $MgSO_4$, filtered and concentrated under reduced pressure. The compound was purified by column chromatography (SiO_2, pentane:EtOAc, *p*-Anisaldehyde stain) affording the desired alcohol.

Methylcyclododecan-1-ol (26e)

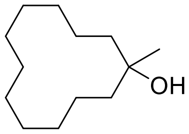

26e was synthesized following the *general procedure B* in Et_2O (25 mL, 0.2 M) from cyclododecanone (**25e**, 1.00 g, 5.49 mmol, 1.0 equiv) using methylmagnesium bromide (3.0 M in Et_2O, 2.0 mL, 6.00 mmol, 1.1 equiv) diluted with THF (4.0 mL).
Column chromatography (SiO_2, 10% EtOAC in Pentane) afforded methylcyclododecan-1-ol **26e** (609 mg, 3.07 mmol, 56 %) as a white amorphous solid. The NMR data was collected and the compound was used in the next step without further analysis.

Rf (pentane:EtOAc 9:1) = 0.4.
^{1}H NMR (400 MHz, $CDCl_3$) δ: 1.59 – 1.52 (m, 2 H, C*H*$_2$), 1.45 – 1.25 (m, 20 H, C*H*$_2$), 1.17 (s, 3 H, C*H*$_3$).
^{13}C NMR (101 MHz, $CDCl_3$) δ: 73.8, 36.3, 29.2, 26.6, 26.2, 22.7, 22.2, 20.1.

4-Methyltetrahydro-2*H*-pyran-4-ol (26f)

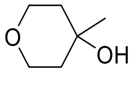

26f was synthesized following the *general procedure B* in THF (50 mL, 0.2 M) from tetrahydro-4*H*-pyran-4-one (**25f**, 0.94 mL, 10 mmol, 1.0 equiv) using methylmagnesium bromide (3.0 M in Et_2O, 3.7 mL, 11 mmol, 1.1 equiv) diluted with THF (7.3 mL). Column chromatography (SiO_2, 25% EtOAc in Pentane) afforded 4-methyltetrahydro-2*H*-pyran-4-ol **26f** (604 mg, 5.20 mmol, 52 %) as a colourless oil.
Rf (pentane:EtOAc 3:1) = 0.3.
^{1}H NMR (400 MHz, $CDCl_3$) δ: 3.81 – 3.75 (m, 2H, OC*H*$_2$), 3.72 – 3.76 (m, 2H, OC*H*$_2$), 1.77 – 1.62 (m, 2H, C*H*$_2$), 1.58 – 1.48 (m, 2H, C*H*$_2$), 1.28 (s, 3H, C*H*$_3$).

^{13}**C NMR** (101 MHz, $CDCl_3$) δ: 67.5, 64.4, 39.6, 30.3.

General procedure C: Synthesis of tertiary alcohols from esters

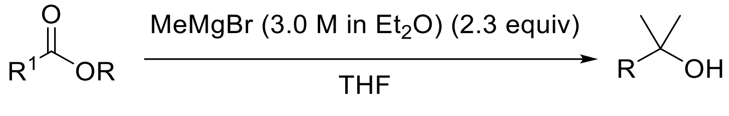

27m-n **26m-n**

An oven dried two necked flask, equipped with a magnetic stirrer, was charged with the ester **27m-n** (1.0 equiv) and dissolved in anhydrous THF (1.0 M). The reaction was cooled to 0 °C with an ice bath. The methyl magnesium bromide solution was diluted to 1 M with THF and added dropwise to the cooled solution *via* syringe. The reaction was left to stir at room temperature overnight (15 to 18 h) at this time the reaction was quenched with sat. aq. NH_4Cl. The aqueous layer was extracted 3 times with EtOAc then the combined organic layers were washed with sat. aq. NaCl. The organic layers were then dried on $MgSO_4$, filtered and concentrated under reduced pressure. The compound was purified by column chromatography (SiO_2, pentane:EtOAc 9:1, 4:1, *p*-Anisaldehyde stain blue to purple and black spots) affording the desired alcohol.

1-(4-Methoxyphenyl)-2-methylpropan-2-ol (26m)

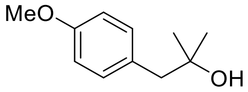

26m was synthesized following the *general procedure B*: in THF (60 mL, 0.1 M) using methyl 2-(4-methoxyphenyl)acetate (**27m** ,1.0 mL, 6.3 mmol, 1.0 equiv) and methyl magnesium bromide (3 M in Et_2O) (4.8 mL, 14 mmol, 2.3 equiv) diluted with 10 mL of THF.
Column chromatography (SiO_2 ca. 40 g, pentane:EtOAc 9:1 to 8:2) afforded 1-(4-methoxyphenyl)-2-methylpropan-2-ol **26m** (0.898 g, 4.98 mmol, 79%).

Rf (pentane:EtOAc 9:1) = 0.3
^{1}**H NMR** (400 MHz, $CDCl_3$) δ 7.16 – 7.11 (m, 2H, Ar*H*), 6.88 – 6.83 (m, 2H, Ar*H*), 3.80 (s, 3H, OMe), 2.71 (s, 2H, ArC*H*$_2$), 1.21 (s, 6H, C(C*H*$_3$)$_2$).
^{13}**C NMR** (101 MHz, $CDCl_3$) δ 158.5, 131.5, 129.9, 113.8, 70.9, 55.4, 48.9, 29.2.
The reported NMR data are consitant with the reported data.[15]

1-(2-Fluorophenyl)-2-methylpropan-2-ol (26n)

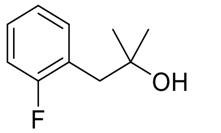

26n was synthesized following the *general procedure B*: in THF (60 mL, 0.1 M) using methyl 2-(2-fluorophenyl)acetate (**27n**, 1.0 mL, 6.8 mmol, 1.0 equiv) and methyl magnesium bromide (3 M in Et_2O) (5.2 mL, 16 mmol, 2.3 equiv) diluted with 10 mL of THF.
Column chromatography (SiO_2 ca. 40g, pentane:EtOAc 9:1 to 8:2) afforded 1-(2-fluorophenyl)-2-methylpropan-2-ol **26n** (0.723 g, 4.30 mmol, 63%).

Rf (pentane:EtOAc 9:1) = 0.3.
^{1}**H NMR** (400 MHz, $CDCl_3$) δ 7.29 – 7.18 (m, 2H, Ar*H*), 7.13 – 7.00 (m, 2H, Ar*H*), 2.83 (d, J = 1.5 Hz, 2H, C*H*$_2$), 1.48 (s, 1H, O*H*), 1.25 (d, J = 0.9 Hz, 6H, C(C*H*$_3$)$_2$).

---

[15] Okamura, T.; Egoshi, S.; Dodo, K.; Sodeoka, M.; Iwabuchi, Y.; Kanoh, N. *Chem. – Eur. J.* **2019**, *25*, 16002–16006.

^{13}C NMR (101 MHz, $CDCl_3$) δ 161.5 (d, *J* = 244.7 Hz), 132.8 (d, *J* = 4.7 Hz), 128.3 (d, *J* = 8.2 Hz), 124.9 (d, *J* = 16.0 Hz), 123.8 (d, *J* = 3.5 Hz), 115.4 (d, *J* = 23.0 Hz), 71.3, 42.3, 29.1.
^{19}F NMR (376 MHz, $CDCl_3$) δ -116.1.
IR (ν_{max}, cm^{-1}) 3420 (m), 3061 (m), 2975 (m), 2963 (m), 2936 (m), 1583 (m), 1493 (s), 1455 (s), 1228 (s), 1184 (s), 1134 (s), 753 (s).
HRMS (APPI/LTQ-Orbitrap) m/z: $[M]^+$ Calcd for $C_{10}H_{12}F^+$ 151.0918; Found 151.0921.

2.5. Synthesis of cesium salts

General procedure D: Synthesis of cesium salts from tertiary alcohols

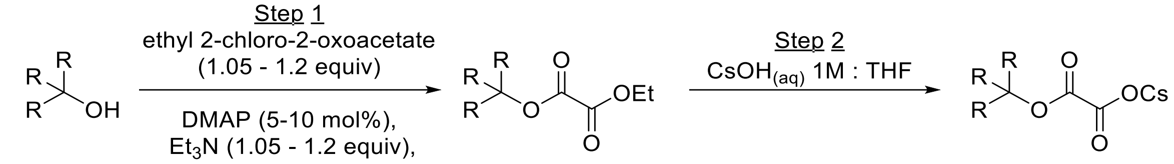

26a, 26c-f, 26i-q 26w, 26x → **28a-f, 28i-q 28w, 26x** → **3a-f, 3i-q 3w, 3x**

Step 1: Following a modified reported procedure,[16] a two necked round bottomed flask, equipped with a magnetic stirrer, was charged with THF or CH_2Cl_2 (0.1 or 0.2 M),[17] DMAP (0.15 mmol, 5 mol%), the tertiary alcohol **26a-x** (3.00 mmol, 1.00 equiv) and triethylamine (1.05 - 1.2 equiv) were then added. Ethyl 2-chloro-2-oxoacetate (1.05 - 1.2 equiv) was then added dropwise and giving a yellowish solution. The reaction was then stirred for 1 h – 2 h at room temperature. Upon full conversion of the alcohol, indicated by TLC analysis, the reactions were quenched with sat. aq. NH_4Cl. The layers were then separated and the organic layer was then washed twice with brine (ca. 10 mL). The organic layer was then dried over Na_2SO_4 and filtered. A solid deposit for flash chromatography was prepared: (ca. 5-7 g SiO_2) concentrated under reduced pressure. The compound was purified by flash chromatography (SiO_2, pentane:EtOAc 9:1, 4:1, *p*-Anisaldehyde stain blue, green or purple spots) affording the desired alkyl ethyl oxalate **28a-x**.

Step 2: Following a modified reported procedure,[13] a round-bottom flask was charged with ethyl oxoacetate **28a-x** (1.75 mmol, 1.00 equiv) followed by the addition of THF (1 M). To this solution, a 1 M stock solution of aq. CsOH (1.7 mmol, 1.00 equiv) was added dropwise (ca. 2 min). The mixture was stirred vigorously for 5 min at room temperature, then concentrated immediately under reduced pressure (T = 55°C - 60 °C: P = 300 mbar to 20 mbar).[18] The resulting solid was then dried under high vacuum for at least 4 hours affording a dry (rarely hygroscopic, some are soap-like) cesium salt **3a-x**.

---

[16] Nawrat, C. C.; Jamison, C. R.; Slutskyy, Y.; MacMillan, D. W. C.; Overman, L. E. *J. Am. Chem. Soc.* **2015**, *137*, 11270–11273.
[17] We have not noticed particular changes of reactivity between THF and CH_2Cl_2 or between 0.1 M or 0.2 M, use of CH_2Cl_2 simplifies extraction.
[18] Other hydrolysis products have been observed when the reaction is left longer or triturated in diethyl ether to attempt purification.

Synthetic and characterization data for alkyl ethyl oxalate intermediates 28a-x and cesium salts 3a-x

*Ethyl 2-(2-methyl-4-phenylbutan-2-yl)oxy-2-oxoacetate (**28a**)*

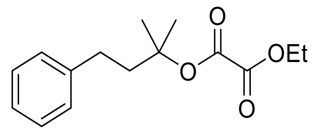

28a was synthesized following step 1 of general *procedure D* in THF (90 mL, 0.1 M) using 2-methyl-4-phenylbutan-2-ol (**22a**, 1.6 mL, 9.1 mmol, 1 equiv), DMAP (0.055 g, 0.46 mmol, 5 mol%), triethylamine (1.3 mL, 9.6 mmol, 1.05 equiv) and ethyl chloro-oxoacetate (1.1 mL, 9.6 mmol, 1.05 equiv).
Column chromatography (SiO_2, pentane:EtOAc 85:15) afforded ethyl (2-methyl-4-phenylbutan-2-yl) oxalate (**28a**, 2.00 g, 7.57 mmol, 83%) as a colorless oil.

Rf (pentane:EtOAc 9:1) = 0.5
^{1}H NMR (400 MHz, $CDCl_3$) δ 7.38 – 7.29 (m, 2H, Ar*H*), 7.25 (d, *J* = 7.1 Hz, 3H, Ar*H*), 4.38 (q, *J* = 7.1 Hz, 2H, OC*H*$_2$-CH$_3$), 2.79 – 2.71 (m, 2H, Ph-C*H*$_2$), 2.25 – 2.16 (m, 2H, C*H*$_2$), 1.66 (s, 6H, d*Me*), 1.43 (t, *J* = 7.1 Hz, 3H, OCH$_2$C*H*$_3$).
^{13}C NMR (101 MHz, $CDCl_3$) δ 158.6, 157.1, 141.6, 128.5, 128.4, 126.0, 86.6, 62.8, 42.5, 30.2, 25.7, 14.0.
IR (vmax, cm-1) 3087 (w), 3062 (w), 3029 (m), 2983 (m), 2949 (m), 2872 (w), 1761 (s), 1737 (s), 1327 (m), 1188 (s), 1163 (s), 1118 (s), 912 (s).
HRMS (ESI/QTOF) m/z: $[M + Na]^+$ Calcd for $C_{15}H_{20}NaO_4^+$ 287.1254; Found 287.1256.

*Cesium 2-(2-methyl-4-phenylbutan-2-yl)oxy-2-oxoacetate (**3a**)*

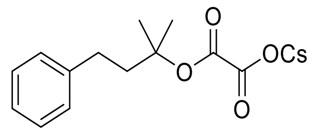

3a was synthesized following step 2 of *general procedure D* in THF (6.5 mL, 0.1 M) using ethyl (2-methyl-4-phenylbutan-2-yl) oxalate (**28a**, 1.70 g, 6.43 mmol, 1.0 equiv) and 1 M aq. CsOH (6.4 mL, 6.4 mmol, 1.0 equiv), affording cesium 2-(2-methyl-4-phenylbutan-2-yl)oxy-2-oxoacetate (**3a**, 2.34 g, 6.36 mmol, 99%) as an off-white amorphous solid.
^{1}H NMR (400 MHz, D_2O) δ 7.31 (m, 5H, Ar*H*), 2.73 – 2.64 (m, 2H, ArC*H*$_2$), 2.20 – 2.11 (m, 2H, C*H*$_2$), 1.55 (s, 6H, C(C*H*$_3$)$_2$).
^{13}C NMR (101 MHz, D_2O) δ 165.2, 164.1, 142.5, 128.7, 128.5, 126.0, 86.0, 41.3, 29.7, 25.4.
HRMS (ESI/QTOF) m/z: $[M - Cs]^-$ Calcd for $C_{13}H_{15}O_4^-$ 235.0976; Found 235.0979.

*Ethyl (tert-butyl)oxy-2-oxoacetate (**28b**)*

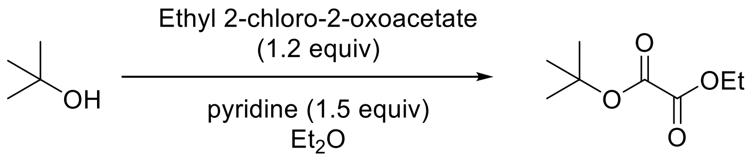

26b **28b**

Following a reported procedure,[19] ethyl 2-chloro-2-oxoacetate (3.6 mL, 32 mmol, 1.2 equiv) was added to a solution of *tert*-butanol (**26b**, 2.0 g, 27 mmol, 1.0 equiv) and pyridine (3.26 mL, 40.5 mmol) in Et_2O (100 mL) and the resulting yellow solution was stirred at room temperature for 4 hours. The organic layer was washed with water (2 x 50 mL) and sat. aq. $NaHCO_3$ solution (50 mL), dried over $MgSO_4$ and

[19] Xu, Y.; McLaughlin, M.; Bolton, E. N.; Reamer, R. A. *J. Org. Chem.* **2010**, *75*, 8666–8669.

concentrated under reduced pressure. The crude material was purified by flash column chromatography on a short column of silica gel (1:20 Et_2O:pentane) to give *tert*-butyl ethyl oxalate (**28b**, 4.4 g, 25 mmol, 98%) as a colorless oil.

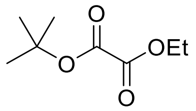

^{1}H NMR (400 MHz, $CDCl_3$) δ 4.31 (q, *J* = 7.1 Hz, 2H, OCH_2), 1.55 (s, 9H, *t*Bu), 1.36 (t, *J* = 7.1 Hz, 3H, CH_2CH_3).
^{13}C NMR (101 MHz, $CDCl_3$) δ 158.8, 157.3, 85.0, 62.9, 27.9, 14.1. The NMR data obtained are consistent with the reported literature data.[16]

*Cesium (tert-butyl)oxy-2-oxoacetate (**3b**)*

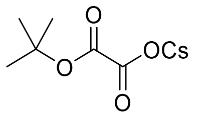

3b was synthesized following <u>step 2</u> of *general procedure D* in THF (2.1 mL, 0.1 M) using *tert*-butyl ethyl oxalate (**28c**, 0.366 g, 2.10 mmol, 1.0 equiv) and 1 M aq. CsOH (2.1 mL, 2.1 mmol, 1.0 equiv), affording cesium (*tert*-butyl)oxy-2-oxoacetate (**3c** 0.505 g, 1.82 mmol, 86%) as a colorless amorphous solid.
^{1}H NMR (400 MHz, DMSO-d_6) δ 1.37 (s, 9H, $C(CH_3)_3$).
^{13}C NMR (101 MHz, DMSO-d_6) δ 167.5, 163.5, 78.0, 27.9.
HRMS (ESI/QTOF) m/z: $[M + Na]^+$ Calcd for $C_6H_9CsNaO_4^+$ 300.9448; Found 300.9451.

*Ethyl 2-(1-methylcyclopent-1-yl)oxy-2-oxoacetate (**28c**)*

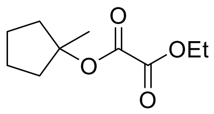

28c was synthesized following <u>step 1</u> of *general procedure D* in THF (16 mL, 0.2 M) using 1-methylcyclopentan-1-ol (**22c**, 337 mg, 3.36 mmol, 1.0 equiv), DMAP (21 mg, 0.17 mmol, 5 mol%), triethylamine (0.56 mL, 11 mmol, 1.2 equiv) and ethyl chloro-oxoacetate (0.45 mL, 11 mmol, 1.2 equiv).
Column chromatography (SiO_2, pentane:EtOAc 9:1 to 8:2) afforded ethyl (1-methylcyclopentan-1-yl) oxalate (**28c**, 596 mg, 2.98 mmol, 89%).

Rf (pentane:EtOAc 9:1) = 0.5.
^{1}H NMR (400 MHz, $CDCl_3$) δ 4.31 (q, *J* = 7.1 Hz, 2H, OCH_2CH_3), 2.21 (ttd, *J* = 10.4, 4.8, 2.4 Hz, 2H, CH_2), 1.83 – 1.71 (m, 4H, CH_2), 1.71 – 1.58 (m, 5H, CH_2 + CH_3), 1.36 (t, *J* = 7.1 Hz, 3H, OCH_2CH_3).
^{13}C NMR (101 MHz, $CDCl_3$) δ 158.8, 157.5, 94.2, 62.9, 39.0, 24.1, 23.8, 14.1.
IR (ν_{max}, cm^{-1}) 2984 (m), 2942 (m), 2910 (w), 1762 (s), 1737 (s), 1370 (m), 1324 (m), 1201 (s), 1139 (s), 1017 (m), 846 (m).
HRMS (ESI/QTOF) m/z: $[M + Na]^+$ Calcd for $C_{10}H_{16}NaO_4^+$ 223.0941; Found 223.0935.

*Cesium 2-(1-methylcyclopent-1-yl)oxy-2-oxoacetate (**3c**)*

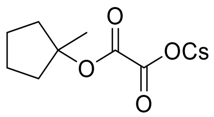

3c was synthesized following <u>step 2</u> of *general procedure D* in THF (1.2 mL, 0.1 M) using ethyl (1-methylcyclopent-1-yl) oxalate (**28c**, 0.37 g, 1.8 mmol, 1.0 equiv) and 1 M aq. CsOH (1.8 mL, 1.8 mmol, 1.0 equiv), affording cesium 2-(1-methylcyclopent-1-yl)oxy-2-oxoacetate (**3c**, 0.541 g, 1.78 mmol, 97%).
^{1}H NMR (400 MHz, DMSO) δ 1.97 (dddt, *J* = 7.1, 5.3, 3.0, 1.8 Hz, 2H, CH_2), 1.72 – 1.49 (m, 6H, CH_2), 1.46 (s, 3H, CH_3).
^{13}C NMR (101 MHz, DMSO) δ 167.5, 163.5, 87.3, 24.3, 23.3, 14.2. Consistent with reported data.[16]

*Ethyl 2-(1-methylcyclohex-1-yl)oxy-2-oxoacetate (**28d**)*

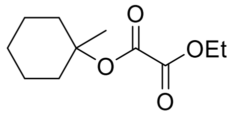

28d was synthesized following <u>step 1</u> of *general procedure D* in THF (90 mL, 0.1 M) using 1-methylcyclohexan-1-ol (**22d**, 1.1 mL, 8.8 mmol, 1.0 equiv), DMAP (107 mg, 0.876 mmol, 0.1 equiv) triethylamine (1.50 mL, 10.5 mmol, 1.2 equiv) and ethyl chloro-oxoacetate (1.20 mL, 10.5 mmol, 1.2 equiv).

Column chromatography (SiO_2, 2% EtOAc in Pentane) afforded ethyl (1-methylcyclohexyl) oxalate (**28d**, 1.18 g, 5.51 mmol, 63%) as a pale yellow oil.

Rf (pentane:EtOAc 98:2) = 0.3.
^{1}H NMR (400 MHz, $CDCl_3$) δ: 4.30 (q, *J* = 7.12 Hz, 2H, CO_2CH_2), 2.21 – 2.18 (m, 2H, CH_2), 1.58 – 1.44 (m, 8 H, CH_2), 1.55 (s, 3H, CH_3), 1.35 (t, *J* = 7.12 Hz, 3 H, $CO_2CH_2CH_3$).
^{13}C NMR (101 MHz, $CDCl_3$) δ: 158.8, 157.2, 86.7, 62.8, 36.4, 25.3, 25.1, 22.1, 14.1.
IR (ν_{max}, cm^{-1}): 2979 (w), 2938 (m), 2864 (w), 1743 (s), 1454 (w), 1326 (m), 1192 (s), 1146 (s).
HRMS (ESI/QTOF) m/z: $[M + Na]^+$ Calcd for $C_{11}H_{18}NaO_4^+$ 237.1097; found 237.1094

*Ethyl 2-(1-methylcyclohex-1-yl)oxy-2-oxoacetate (**3d**)*

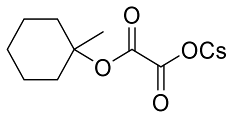

3d was synthesized following <u>step 2</u> of *general procedure D* in THF (5.0 mL, 0.1 M) using ethyl (1-methylcyclohexyl) oxalate (**28d**, 1.07 g, 5.00 mmol, 1.0 equiv) and 1 M aq. CsOH (5.0 mL, 5.0 mmol, 1.0 equiv). Affording cesium 2-((1-methylcyclohexyl)oxy)-2-oxoacetate (**3d**, 1.4 g, 4.4 mmol, 88%) as a colorless amorphous solid.

^{1}H NMR (400 MHz, DMSO-d6) δ: 2.08 – 1.96 (m, 2H, CH_2), 1.56 – 1.43 (m, 3H, CH_2), 1.43 – 1.29 (m, 7H, $CH_2 + CH_3$), 1.27 – 1.18 (m, 1H, CH_2).
^{13}C NMR (101 MHz, DMSO) δ: 167.7, 163.6, 79.2, 36.2, 25.3, 25.0, 21.5.
HRMS (ESI/QTOF) m/z: $[M - Cs]^-$ Calcd for $C_9H_{13}O_4^-$ 185.0819; Found 185.0819.

*Ethyl (1-methylcyclododecyl) oxalate **28e***

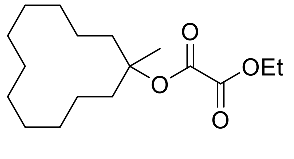

28e was synthesized following <u>step 1</u> of *general procedure D* in THF (25 mL, 0.1 M) using 1-methylcyclododecan-1-ol (**22e**, 500 mg, 2.52 mmol, 1.0 equiv), DMAP (31 mg, 0.25 mmol, 10 mol%), triethylamine (0.42 mL, 3.0 mmol, 1.2 equiv) and ethyl chloro-oxoacetate (0.34 mL, 3.0 mmol, 1.2 equiv).

Column chromatography (SiO_2, 20% EtOAc in Pentane) afforded ethyl (1-methylcyclododecyl) oxalate (**28e**, 1.08 g, 4.25 mmol, 71 %) as an off-white amorphous solid.

Rf (pentane:EtOAc 4:1) = 0.5.
^{1}H NMR (400 MHz, $CDCl_3$) δ 4.31 (q, *J* = 7.2 Hz, 2H, CO_2CH_2), 2.10 – 1.98 (m, 2H, CH_2), 1.74 – 1.61 (m, 2H, CH_2), 1.55 (s, 3H, CH_3), 1.49 – 1.23 (m, 21H, $CH_2 + CH_2CH_3$).
^{13}C NMR (101 MHz, $CDCl_3$) δ 158.9, 157.1, 90.8, 62.9, 32.9, 26.2, 26.2, 24.0, 22.5, 22.0, 19.5, 14.1.
IR (ν_{max}, cm^{-1}): 2939 (s), 2861 (m), 1744 (s), 1467 (m), 1375 (m), 1325 (m), 1190 (s), 1152 (s)
HRMS (ESI/QTOF) m/z: $[M + Na]^+$ Calcd for $C_{17}H_{30}NaO_4^+$ 321.2036; Found 321.2037.

*Cesium 2-((1-methylcyclododecyl)oxy)-2-oxoacetate (**3e**)*

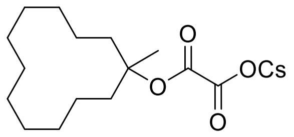

3e was synthesized following <u>step 2</u> of *general procedure D* in THF (1.0 mL, 0.1 M) using ethyl (1-methylcyclododecyl) oxalate (**28e**, 300 mg, 1.00 mmol, 1.0 equiv) and 1 M aq. CsOH (1.0 mL, 1.00 mmol, 1.0 equiv). Cesium 2-((1-methylcyclododecyl)oxy)-2-oxoacetate (**3e**, 300 mg, 0.745 mmol, 74 %) was obtained as an off-white solid.

^{1}H NMR (400 MHz, DMSO) δ: 1.90 – 1.77 (m, 2H, C*H*$_2$), 1.56 – 1.42 (m, 2H, C*H*$_2$), 1.38 (s, 3H, C*H*$_3$), 1.34 – 1.18 (m, 18H, C*H*$_2$).

^{13}C NMR (101 MHz, DMSO) δ: 168.1, 164.0, 83.4, 33.21 26.3, 26.2, 24.4, 22.3, 22.0, 19.2.

HRMS (ESI/QTOF) m/z: [M + Na]$^+$ Calcd for $C_{15}H_{25}CsNaO_4^+$ 425.0700; Found 425.0695.

*Ethyl (4-methyltetrahydro-2H-pyran-4-yl) oxalate (**28f**)*

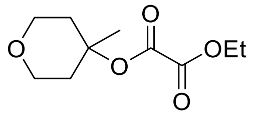

28f was synthesized following <u>step 1</u> of *general procedure D* in THF (45 mL, 0.1 M) using 4-methyloxan-4-ol (**22f**, 500 mg, 4.30 mmol, 1.0 equiv), DMAP (53 mg, 0.43 mmol, 10 mol%), triethylamine (0.72 mL, 5.2 mmol, 1.2 equiv) and ethyl chloro-oxoacetate (0.58 mL, 5.2 mmol, 1.2 equiv).

Column chromatography (SiO_2, 15% EtOAc in Pentane) afforded ethyl (4-methyltetrahydro-2*H*-pyran-4-yl) oxalate (**28f**, 785 mg, 3.63 mmol, 84 %) as a pale yellow oil.

Rf (pentane:EtOAc 85:15) = 0.5.

^{1}H NMR (400 MHz, $CDCl_3$) δ: 4.33 (q, *J* = 7.1 Hz, 2H, $CO_2CH_2CH_3$), 3.83 – 3.59 (m, 4H, OC*H*$_2$), 2.27 – 2.17 (m, 2H, C*H*$_2$), 1.78 (ddd, *J* = 14.6, 10.1, 5.0 Hz, 2H, C*H*$_2$), 1.62 (s, 3H, C*H*$_3$), 1.37 (t, *J* = 7.1 Hz, 3H, $CO_2CH_2CH_3$).

^{13}C NMR (101 MHz, $CDCl_3$) δ: 158.4, 157.1, 83.2, 63.7, 63.1, 36.6, 25.0, 14.1.

IR (ν_{max}, cm^{-1}): 2968 (w), 2864 (w), 1744 (s), 1462 (w), 1324 (m), 1192 (s), 1134 (s), 1023 (m).

HRMS (ESI/QTOF) m/z: [M + Na]$^+$ Calcd for $C_{10}H_{16}NaO_5^+$ 239.0890; Found 239.0894.

*cesium (4-methyltetrahydro-2H-pyran-4-yl) oxalate (**3f**)*

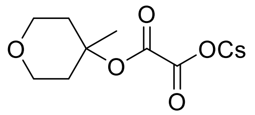

3f was synthesized following <u>step 2</u> of *general procedure D* in THF (2.5 mL, 0.1 M) using ethyl (4-methyltetrahydro-2*H*-pyran-4-yl) oxalate (**28f**, 541 mg, 2.50 mmol, 1.0 equiv) and 1 M aq. CsOH (2.5 mL, 2.50 mmol, 1.0 equiv). Cesium 2-((3-methyl-1-phenylpentan-3-yl)oxy)-2-oxoacetate (**3f**, 725 mg, 2.27 mmol, 91%) was obtained as an off-white amorphous solid.

^{1}H NMR (400 MHz, DMSO) δ: 3.66 – 3.49 (m, 4H, OC*H*$_2$), 2.04 – 1.93 (m, 2H, C*H*$_2$), 1.67 – 1.53 (m, 2H, C*H*$_2$), 1.45 (s, 3H, C*H*$_3$).

^{13}C NMR (101 MHz, DMSO-d$_6$) δ: 167.6, 163.2, 76.5, 62.9, 36.6, 24.9.

HRMS (ESI/QTOF) m/z: [M + Na]$^+$ Calcd for $C_8H_{11}CsNaO_5^+$ 342.9553; Found 342.9553.

*Ethyl 2-(1-methylcycloheptan-1-yl)oxy-2-oxoacetate (**28i**)*

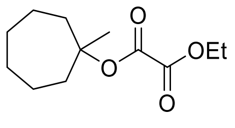

28i was synthesized following <u>step 1</u> of *general procedure D* in DCM (35 mL, 0.1 M) using 1-methylcycloheptan-1-ol (**22i**, 0.30 mL, 3.4 mmol, 1 equiv), DMAP (42 mg, 0.34 mmol, 10 mol%), triethylamine (0.52 mL, 3.7 mmol, 1.1 equiv) and ethyl chloro-oxoacetate (0.42 mL, 3.8 mmol, 1.1 equiv).

Column chromatography (SiO_2, pentane:EtOAc 9:1) afforded ethyl (1-methylcycloheptan-1-yl) oxalate (**28i**, 0.373 g, 1.84 mmol, 54%).

Rf (pentane:EtOAc 9:1) = 0.5.
^{1}H NMR (400 MHz, $CDCl_3$) δ 4.30 (q, *J* = 7.1 Hz, 2H, OCH_2CH_3), 2.20 (ddd, *J* = 14.9, 8.6, 1.7 Hz, 2H, cyclic-CH_2), 1.82 (ddd, *J* = 14.7, 9.8, 1.8 Hz, 2H, cyclic-CH_2), 1.70 – 1.39 (m, 11H, cyclic-$(CH_2))_4$ + CH_3, 1.35 (t, *J* = 7.2 Hz, 3H, OCH_2CH_3).
^{13}C NMR (101 MHz, $CDCl_3$) δ 158.9, 157.3, 91.1, 62.8, 40.0, 29.5, 26.6, 22.6, 14.1.
IR (ν_{max}, cm^{-1}) 3005 (w), 2929 (m), 2858 (m), 1760 (s), 1736 (s), 1459 (m), 1446 (m), 1371 (m), 1323 (m), 1205 (s), 1186 (s), 1159 (s), 1128 (s), 861 (m).
HRMS (ESI/QTOF) m/z: $[M + Na]^+$ Calcd for $C_{12}H_{20}NaO_4^+$ 251.1254; Found 251.1259.

*Cesium 2-(1-methylcycloheptan-1-yl)oxy-2-oxoacetate (**3i**)*

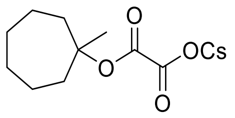

3i was synthesized following <u>step 2</u> of *general procedure D* in THF (1.1 mL, 0.1 M) using ethyl (1-methylcycloheptan-1-yl) oxalate (**28i**, 0.250 g, 1.10 mmol, 1.0 equiv) and 1 M aq. CsOH (1.1 mL, 1.1 mmol, 1.0 equiv), affording cesium 2-(1-methylcycloheptan-1-yl)oxy-2-oxoacetate (**3i**, 0.332 g, 1.00 mmol, 91%). Amorphous solid.
^{1}H NMR (400 MHz, DMSO-d_6) δ 2.02 (ddd, *J* = 14.3, 8.6, 1.6 Hz, 2H, cyclic-CH_2), 1.67 (ddd, *J* = 14.4, 9.9, 1.9 Hz, 2H, cyclic-CH_2), 1.60 – 1.42 (m, 6H, cyclic-CH_2), 1.41 (s, 3H, CH_3), 1.40 – 1.28 (m, 2H, cyclic-CH_2).
^{13}C NMR (101 MHz, DMSO-d_6) δ 168.2, 164.1, 83.9, 29.3, 27.2, 22.5. 1 carbon is unresolved.

*Ethyl 2-(((1S,3S)-adamantan-1-yl)oxy)-2-oxoacetate (**28j**)*

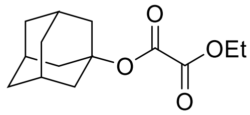

28j was synthesized following <u>step 1</u> of *general procedure D* in DCM (25 mL, 0.1 M) using adamant-1-ol (**22j**, 378 mg, 2.48 mmol, 1.0 equiv), DMAP (30.4 mg, 248 mmol, 10 mol%), triethylamine (0.41 mL, 3.0 mmol, 1.2 equiv) and ethyl chloro-oxoacetate (0.34 mL, 3.0 mmol, 1.2 equiv).
Column chromatography (SiO_2, 15% EtOAc in Pentane) afforded ethyl 2-(((1S,3S)-adamantan-1-yl)oxy)-2-oxoacetate (**28j**, 442 mg, 1.75 mmol, 71 %) as a pale yellow oil.

Rf (pentane:EtOac 9:1) = 0.5.
^{1}H NMR (400 MHz, $CDCl_3$) δ 4.31 (q, *J* = 7.1 Hz, 2H, OCH_2CH_3), 2.19 (d, *J* = 2.7 Hz, 9H, ad-CH_x), 1.76 – 1.55 (m, 6H, ad-CH_x), 1.36 (t, *J* = 7.1 Hz, 3H, OCH_2CH_3).
^{13}C NMR (101 MHz, $CDCl_3$) δ 158.8, 156.8, 85.1, 62.9, 41.0, 36.1, 31.1, 14.1.
IR (ν_{max}, cm^{-1}) 2911 (m), 2854 (w), 1760 (s), 1733 (s), 1176 (s), 1155 (s), 1044 (m).
HRMS (APPI/LTQ-Orbitrap) m/z: $[M + Na]^+$ Calcd for $C_{14}H_{20}NaO_4^+$ 275.1254; Found 275.1256.

*Cesium 2-(((1S,3S)-adamantan-1-yl)oxy)-2-oxoacetate (**3j**)*

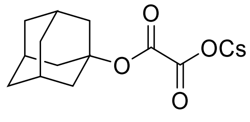

3j was synthesized following <u>step 2</u> of *general procedure D* in THF (2.5 mL, 0.1 M) using ethyl 2-(((1*S*,3*S*)-adamantan-1-yl)oxy)-2-oxalate (**28j**, 252 mg, 1.00 mmol, 1.0 equiv) and 1 M aq. CsOH (2.5 mL, 2.5 mmol, 1.0 equiv). cesium 2-(((1*S*,3*S*)-adamantan-1-yl)oxy)-2-oxoacetate (**3j**, 0.32 g, 0.91 mmol, 91%) was obtained as an off-white amorphous solid.

^{1}H NMR (400 MHz, DMSO) δ: 2.12 – 2.07 (m, 3H, C*H*), 2.06 – 1.99 (m, 6H, CH_2), 1.64 – 1.59 (m, 6H, CH_2).
^{13}C NMR (101 MHz, DMSO) δ: 167.3, 163.4, 78.0, 41.0, 35.8, 30.2.
HRMS (ESI/QTOF) m/z: [M - Cs]$^-$ Calcd for $C_{12}H_{15}O_4^-$ 223.0976; Found 223.0974.

*Ethyl (3-methyl-1-phenylpentan-3-yl) oxalate (**28k**)*

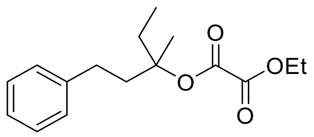

28k was synthesized following <u>step 1</u> of *general procedure D* in THF (60 mL, 0.1 M) using 3-methyl-1-phenylpentan-3-ol (**22k** 1.1 g, 6.0 mmol, 1.0 equiv), DMAP (73 mg, 0.60 mmol, 10 mol%), triethylamine (1.0 mL, 7.2 mmol, 1.2 equiv) and ethyl chloro-oxoacetate (0.80 mL, 7.2 mmol, 1.2 equiv).
Column chromatography (SiO_2, 2% EtOAC in Pentane) afforded ethyl (3-methyl-1-phenylpentan-3-yl) oxalate (**28k**, 1.61 g, 5.78 mmol, 96 %) as a colourless oil.

Rf (pentane:EtOAc 98:2) = 0.4.
^{1}H NMR (400 MHz, $CDCl_3$) δ: 7.33 – 7.24 (m, 2H, Ar*H*), 7.23 – 7.16 (m, 3H, Ar*H*), 4.32 (q, *J* = 7.1 Hz, 2H, $CO_2CH_2CH_3$), 2.72 – 2.59 (m, 2H, ArCH_2), 2.30 – 2.18 (m, 1H, CH_2), 2.17 – 1.99 (m, 2H, CH_2), 1.97 – 1.85 (m, 1H, CH_2), 1.57 (s, 3H, CH_3), 1.37 (t, *J* = 7.1 Hz, 3H, $CO_2CH_2CH_3$), 0.95 (t, *J* = 7.5 Hz, 3H, CH_2CH_3).
^{13}C NMR (101 MHz, $CDCl_3$) δ: 158.7, 157.2, 141.8, 128.6, 128.5, 126.1, 89.6, 62.9, 39.7, 30.9, 30.1, 23.1, 14.1, 8.1.
IR (ν_{max}, cm^{-1}): 2979 (m), 2943 (w), 1739 (s), 1458 (m), 1323 (m), 1185 (s), 1115 (m), 1019 (m).
HRMS (ESI/QTOF) m/z: [M + Na]$^+$ Calcd for $C_{16}H_{22}NaO_4^+$ 301.1410; Found 301.1412.

*Cesium 2-((3-methyl-1-phenylpentan-3-yl)oxy)-2-oxoacetate (**3k**)*

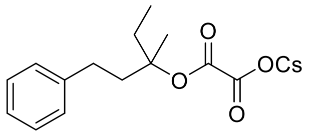

3k was synthesized following <u>step 2</u> of *general procedure D* in THF (3.0 mL, 0.1 M) using ethyl (3-methyl-1-phenylpentan-3-yl) oxalate (**28k**, 835 mg, 3.00 mmol, 1.0 equiv) and 1 M aq. CsOH (3.0 mL, 3.00 mmol, 1.0 equiv). Cesium 2-((3-methyl-1-phenylpentan-3-yl)oxy)-2-oxoacetate (**3k**, 951 mg, 2.49 mmol, 83%) was obtained as an off-white amorphous solid.
^{1}H NMR (400 MHz, DMSO-d_6) δ: 7.29 – 7.24 (m, 2H, Ar*H*), 7.19 – 7.14 (m, 3H, Ar*H*), 2.59 – 2.54 (m, 2H, Ar-CH_2), 2.11 – 2.03 (m, 1H, CH_2), 1.97 – 1.84 (m, 2H, CH_2), 1.76 – 1.67 (m, 1H, CH_2), 1.36 (s, 3H, CH_3), 0.84 (t, *J* = 7.53 Hz, 3H, CH_2CH_3).
^{13}C NMR (101 MHz, DMSO-d_6) δ: 167.7, 163.5, 142.3, 128.3, 128.2, 125.6, 82.3, 64.8, 30.6, 29.3, 23.2, 7.8.
HRMS (ESI/QTOF) m/z: [M + Na]$^+$ Calcd for $C_{14}H_{17}CsNaO_4^+$ 405.0074; Found 405.0075.

*Ethyl (2,3-dimethylbutan-2-yl)oxy-2-oxoacetate (**28l**)*

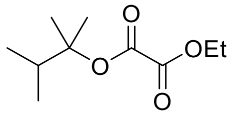

28l was synthesized following <u>step 1</u> of *general procedure D* in DCM (24 mL, 0.1 M) using 2,3-dimethyl-2-butanol (**22l**, 0.30 mL, 2.4 mmol, 1.0 equiv), DMAP (30 mg, 0.24 mmol, 10 mol%), triethylamine (0.35 mL, 2.5 mmol, 1.05 equiv) and ethyl chloro-oxoacetate (0.3 mL, 2.5 mmol, 1.05 equiv).
Column chromatography (SiO_2, pentane:EtOAc 9:1) afforded ethyl (2,3-dimethylbutan-2-yl) oxalate (**28l**, 0.340 g, 1.68 mmol, 70%).

Rf (pentane:EtOAc 9:1) = 0.5.

^{1}**H NMR** (400 MHz, $CDCl_3$) δ 4.30 (q, *J* = 7.2 Hz, 2H, OC*H*$_2$CH$_3$), 2.27 (hept, *J* = 6.9 Hz, 1H, C*H*(CH$_3$)$_2$), 1.49 (s, 6H, OC(C*H*$_3$)$_2$), 1.35 (t, *J* = 7.2 Hz, 3H, OCH$_2$C*H*$_3$), 0.94 (d, *J* = 6.9 Hz, 6H, CH(C*H*$_3$)$_2$).
^{13}**C NMR** (101 MHz, $CDCl_3$) δ 158.9, 157.3, 90.4, 62.8, 36.3, 22.5, 17.4, 14.1.
IR (ν_{max}, cm^{-1}) 2995 (m), 2983 (m), 2962 (w), 2946 (w), 2891 (w), 2878 (w), 2840 (w), 1763 (s), 1737 (s), 1467 (m), 1371 (m), 1324 (s), 1191 (s), 1130 (s), 1094 (s), 1017 (m).
HRMS (ESI/QTOF) m/z: [M + Na]$^+$ Calcd for $C_{10}H_{18}NaO_4^+$ 225.1097; Found 225.1099.

*Cesium (2,3-dimethylbutan-2-yl)oxy-2-oxoacetate (**3l**)*

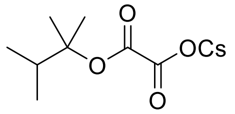

3l was synthesized following step 2 of *general procedure D* in THF (1.0 mL, 0.1 M) using ethyl (2,3-dimethylbutan-2-yl) oxalate (**28l**, 0.200 g, 0.989 mmol, 1.0 equiv) and 1 M aq. CsOH (0.99 mL, 0.99 mmol, 1.0 equiv), affording cesium (2,3-dimethylbutan-2-yl)oxy-2-oxoacetate (**3l**, 137 mg, 0.447 mmol, 45%) as a colorless amorphous solid.
^{1}**H NMR** (400 MHz, DMSO-*d*$_6$) δ 2.22 (hept, *J* = 6.9 Hz, 1H, C*H*(CH$_3$)$_2$), 1.30 (s, 6H, OC(C*H*$_3$)$_2$), 0.84 (d, *J* = 6.9 Hz, 6H, CH(C*H*$_3$)$_2$).
^{13}**C NMR** (101 MHz, DMSO-*d*$_6$) δ 167.6, 163.6, 83.0, 35.3, 22.7, 17.1.
HRMS (ESI/QTOF) m/z: [M + Na]$^+$ Calcd for $C_8H_{13}CsNaO_4^+$ 328.9761; Found 328.9768.

*Ethyl (1-(4-methoxyphenyl)-2-methylpropan-2-yl)oxy-2-oxoacetate (**28m**)*

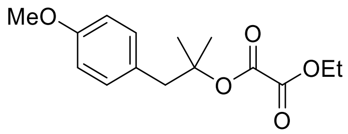

28m was synthesized following step 1 of *general procedure D* in DCM (30 mL, 0.1 M) using 1-(4-methoxyphenyl)-2-methylpropan-2-ol (**22m**, 500 mg, 2.77 mmol, 1.0 equiv), DMAP (33 mg, 0.28 mmol, 10 mol%), triethylamine (0.40 mL, 2.9 mmol, 1.05 equiv) and ethyl chloro-oxoacetate (0.30 mL, 2.9 mmol, 1.05 equiv).
Column chromatography (SiO_2, pentane:EtOAc 4:1) afforded ethyl (1-(4-methoxyphenyl)-2-methylpropan-2-yl) oxalate (**28m**, 270 mg, 0.963 mmol, 35%) as a pale-yellow oil.

Rf (pentane:EtOAc 4:1) = 0.4.
^{1}**H NMR** (400 MHz, $CDCl_3$) δ 7.20 – 7.12 (m, 2H, Ar*H*), 6.87 – 6.79 (m, 2H, Ar*H*), 4.32 (q, *J* = 7.2 Hz, 2H, OC*H*$_2$CH$_3$), 3.79 (s, 3H, OC*H*$_3$), 3.03 (s, 2H, ArC*H*$_2$), 1.53 (s, 6H (C*H*$_3$)$_2$), 1.38 (t, *J* = 7.1 Hz, 3H, OCH$_2$C*H*$_3$).
^{13}**C NMR** (101 MHz, $CDCl_3$) δ 158.6, 158.6, 157.2, 131.8, 128.5, 113.6, 86.8, 62.9, 55.3, 46.1, 25.4, 14.1.
IR (ν_{max}, cm^{-1}) 2995 (m), 2985 (m), 2953 (m), 2937 (m), 2909 (m), 2837 (m), 1761 (s), 1738 (s), 1612 (m), 1513 (s), 1465 (m), 1370 (m), 1321 (s), 1247 (s), 1189 (s), 1177 (s), 1164 (s), 1034 (s), 1019 (s), 851 (s).
HRMS (ESI/QTOF) m/z: [M + Na]$^+$ Calcd for $C_{15}H_{20}NaO_5^+$ 303.1203; Found 303.1206.

*Cesium (1-(4-methoxyphenyl)-2-methylpropan-2-yl)oxy-2-oxoacetate (**3m**)*

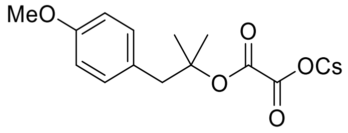

3m was synthesized following step 2 of *general procedure D* in THF (0.7 mL, 0.1 M) using ethyl (1-(4-methoxyphenyl)-2-methylpropan-2-yl) oxalate (**28m**, 0.20 g, 0.71 mmol, 1.0 equiv) and 1 M aq. CsOH (0.7 mL, 0.7 mmol, 1.0 equiv), affording cesium (1-(4-methoxyphenyl)-2-methylpropan-2-yl)oxy-2-oxoacetate (**3m**, 251 mg, 0.653 mmol, 92%) as a coloroless amorphous solid.
^{1}**H NMR** (400 MHz, DMSO-*d*$_6$) δ 7.18 – 7.11 (m, 2H, Ar*H*), 6.85 – 6.78 (m, 2H, Ar*H*), 3.72 (s, 3H, OC*H*$_3$), 2.95 (s, 2H, ArC*H*$_2$), 1.31 (s, 6H, (C*H*$_3$)$_2$).

^{13}C NMR (101 MHz, DMSO-d_6) δ 167.7, 163.3, 157.7, 131.5, 129.3, 113.2, 80.2, 54.9, 44.5, 25.8.
HRMS (ESI/QTOF) m/z: [M - Cs]$^-$ Calcd for $C_{13}H_{15}O_5^-$ 251.0925; Found 251.0936.

*Ethyl (1-(2-fluorophenyl)-2-methylpropan-2-yl)oxy-2-oxoacetate (**28n**)*

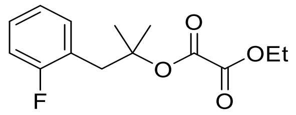

28n was synthesized following step 1 of *general procedure D* in DCM (30 mL, 0.1 M) using 1-(2-fluorophenyl)-2-methylpropan-2-ol (**26n**, 500 mg, 2.97 mmol, 1.0 equiv), DMAP (36 mg, 0.30 mmol, 10 mol%), triethylamine (0.44 mL, 3.1 mmol, 1.05 equiv) and ethyl chloro-oxoacetate (0.35 mL, 3.1 mmol, 1.05 equiv).
Column chromatography (SiO_2, pentane:EtOAc 9:1 to 8:2) afforded ethyl (1-(4-fluorophenyl)-2-methylpropan-2-yl) oxalate (**28n**, 467 mg, 1.74 mmol, 59%) as a colorless oil.

Rf (pentane:EtOAc 9:1) = 0.35.
^{1}H NMR (400 MHz, $CDCl_3$) δ 7.30 (td, *J* = 7.6, 1.8 Hz, 1H, Ar*H*), 7.26 – 7.20 (m, 1H, Ar*H*), 7.12 – 6.99 (m, 2H, Ar*H*), 4.32 (q, *J* = 7.2 Hz, 2H, OCH_2CH_3), 3.16 (d, *J* = 1.4 Hz, 2H, $ArCH_2$), 1.57 (d, *J* = 1.0 Hz, 6H, $(CH_3)_2$), 1.38 (t, *J* = 7.2 Hz, 3H, OCH_2CH_3).
^{13}C NMR (101 MHz, $CDCl_3$) δ 162.8, 160.4, 157.8 (d, *J* = 141.6 Hz), 133.2 (d, *J* = 4.4 Hz), 128.8 (d, *J* = 8.2 Hz), 123.9 (d, *J* = 3.5 Hz), 123.5 (d, *J* = 15.7 Hz), 115.4 (d, *J* = 23.0 Hz), 86.6, 62.9, 39.3, 25.4, 14.1.
^{19}F NMR (376 MHz, $CDCl_3$) δ -115.9.
IR ($ν_{max}$, cm^{-1}) 3004 (m), 2989 (m), 2965 (w), 2938 (m), 2899 (w), 1764 (s), 1737 (s), 1495 (m), 1456 (m), 1372 (m), 1319 (m), 1233 (s), 1190 (s), 1172 (s), 1120 (s), 759 (s).
HRMS (ESI/QTOF) m/z: [M + Na]$^+$ Calcd for $C_{14}H_{17}FNaO_4^+$ 291.1003; Found 291.1002.

*Cesium (1-(2-fluorophenyl)-2-methylpropan-2-yl)oxy-2-oxoacetate (**3n**)*

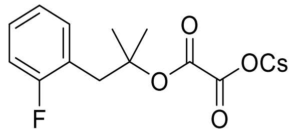

3n was synthesized following step 2 of *general procedure D* in THF (1.5 mL, 0.1 M) using ethyl (1-(2-fluorophenyl)-2-methylpropan-2-yl) oxalate (**28n**, 0.40 g, 1.5 mmol, 1.0 equiv) and 1 M aq. CsOH (1.5 mL, 1.5 mmol, 1.0 equiv), affording cesium (1-(2-fluorophenyl)-2-methylpropan-2-yl)oxy-2-oxoacetate (**3n**, 469 mg, 1.26 mmol, 84%) as a coloroless amorphous solid.
^{1}H NMR (400 MHz, DMSO-d_6) δ 7.36 (td, *J* = 7.7, 1.9 Hz, 1H, Ar*H*), 7.33 – 7.23 (m, 1H, Ar*H*), 7.19 – 7.06 (m, 2H, Ar*H*), 3.08 (s, 2H, $ArCH_2$), 1.34 (d, *J* = 1.0 Hz, 6H, $(CH_3)_2$).
^{13}C NMR (101 MHz, DMSO-d_6) δ 167.6, 163.1, 160.9 (d, *J* = 243.4 Hz), 133.1 (d, *J* = 4.4 Hz), 128.5 (d, *J* = 8.2 Hz), 124.0 (d, *J* = 4.0 Hz), 124.0, 114.9 (d, *J* = 22.9 Hz), 80.0, 37.5, 25.7.
^{19}F NMR (376 MHz, DMSO-d_6) δ -116.6.
HRMS (ESI/QTOF) m/z: [M - Cs]$^-$ Calcd for $C_{12}H_{12}FO_4^-$ 239.0725; Found 239.0719.

*4-Methylbenzylation of 3-methylbutane-1,3-diol (**26o**)*

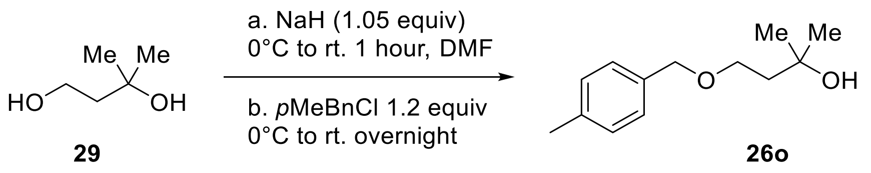

An oven dried 25 mL flask, equipped with a magnetic stirring bar, was flushed with nitrogen then charged with 3-methylbutane-1,3-diol (**29**, 0.26 mL, 2.4 mmol, 1.0 equiv) and anhydrous DMF (12.5 mL, 0.2 M). The solution was cooled to 0 °C and NaH (60% oil dispersion, 102 mg, 2.56 mmol, 1.05 equiv) was added portion-wise under nitrogen. The latter solution was stirred for 1 h at room

temperature. The solution was cooled back down to 0 °C and 1-(chloromethyl)-4-methylbenzene (411 mg, 2.92 mmol, 1.2 equiv) was added under nitrogen. The reaction was left to warm up to RT slowly and was stirred overnight. The reaction was quenched with sat. aq. NH_4Cl (5 mL) then diluted with water (10 mL) and CH_2Cl_2 (15 mL). The layers were separated, and the aqueous layer was washed with CH_2Cl_2 (15 mL). The organic layers were combined and washed with a (sat. aq. NaCl):water (1:1) solution (15 mL) three times. The organic layers were then dried over Na_2SO_4, filtered, and concentrated under reduced pressure. The resulting crude oil was purified by column chromatography (SiO_2, pentane:EtOAc 9:1 to 8:2) affording 2-methyl-4-((4-methylbenzyl)oxy)butan-2-ol (**26h**, 297 mg, 1.43 mmol, 59%) as a colorless oil with some trace impurities. After ^{1}H NMR and HRMS confirmation, the compound was used directly in next step with no further purification or analyses.

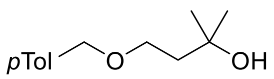

^{1}H NMR (400 MHz, $CDCl_3$) δ 7.21 (d, *J* = 8.0 Hz, 2H, Ar*H*), 7.15 (d, *J* = 7.8 Hz, 2H, Ar*H*), 4.48 (s, 2H, ArC*H*$_2$), 3.70 (t, *J* = 5.9 Hz, 2H, C*H*$_2$), 3.14 (bs, 1H, OH), 2.34 (s, 3H, ArC*H*$_3$), 1.79 (t, *J* = 5.9 Hz, 2H, C*H*$_2$), 1.23 (s, 6H, (C*H*$_3$)$_2$).

HRMS (ESI/QTOF) m/z: $[M + Na]^+$ Calcd for $C_{13}H_{20}NaO_2^+$ 231.1356; Found 231.1358.

*Ethyl (2-methyl-4-((4-methylbenzyl)oxy)butan-2-yl)oxy-2-oxoacetate (**28o**)*

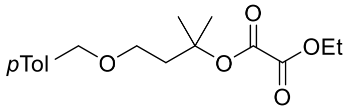

28o was synthesized following <u>step 1</u> of *general procedure D* in THF (11 mL, 0.1 M) using 2-methyl-4-((4-methylbenzyl)oxy)butan-2-ol (**22o**, 220 mg, 1.06 mmol, 1.0 equiv), DMAP (13 mg, 0.11 mmol, 10 mol%), triethylamine (0.16 mL, 1.2 mmol, 1.1 equiv) and ethyl chloro-oxoacetate (0.16 mL, 1.2 mmol, 1.1 equiv).

Column chromatography (SiO_2, pentane:EtOAc 9:1 to 8:2) afforded ethyl (2-methyl-4-((4-methylbenzyl)oxy)butan-2-yl) oxalate (**28o**, 224 mg, 0.726 mmol, 69%).

Rf (pentane:EtOAc 9:1) = 0.3.

^{1}H NMR (400 MHz, $CDCl_3$) δ 7.21 (d, *J* = 8.1 Hz, 2H, Ar*H*), 7.18 – 7.11 (m, 2H, Ar*H*), 4.44 (s, 2H, ArC*H*$_2$), 4.28 (q, *J* = 7.1 Hz, 2H, OC*H*$_2$CH$_3$), 3.59 (t, *J* = 6.6 Hz, 2H, C*H*$_2$), 2.34 (s, 3H, ArC*H*$_3$), 2.19 (t, *J* = 6.6 Hz, 2H, C*H*$_2$), 1.57 (s, 6H, (C*H*$_3$)$_2$), 1.34 (t, *J* = 7.1 Hz, 3H, OCH$_2$C*H*$_3$).

^{13}C NMR (101 MHz, $CDCl_3$) δ 158.6, 157.2, 137.4, 135.3, 129.2, 127.8, 86.1, 73.0, 66.0, 62.9, 39.9, 26.3, 21.3, 14.1.

IR (ν_{max}, cm^{-1}) 3048 (m), 3016 (m), 2991 (m), 2929 (m), 2876 (m), 2860 (m), 1760 (m), 1737 (s), 1370 (m), 1325 (m), 1187 (s), 1134 (s), 1112 (s), 1096 (s), 1018 (m), 802 (s).

HRMS (ESI/QTOF) m/z: $[M + Na]^+$ Calcd for $C_{17}H_{24}NaO_5^+$ 331.1516; Found 331.1518.

*Cesium (2-methyl-4-((4-methylbenzyl)oxy)butan-2-yl)oxy-2-oxoacetate (**3o**)*

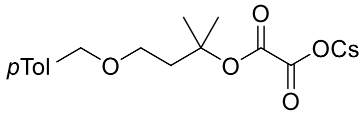

3o was synthesized following <u>step 2</u> of *general procedure D* in THF (0.6 mL, 0.1 M) using ethyl (2-methyl-4-((4-methylbenzyl)oxy)butan-2-yl) oxalate (**28o**, 0.19 g, 0.60 mmol, 1.0 equiv) and 1 M aq. CsOH (0.6 mL, 0.6 mmol, 1.0 equiv), affording cesium (2-methyl-4-((4-methylbenzyl)oxy)butan-2-yl)oxy-2-oxoacetate (**3o**, 233 mg, 0.565 mmol, 94%) as a coloroless amorphous solid.

^{1}H NMR (400 MHz, DMSO-*d*$_6$) δ 7.23 – 7.17 (m, 2H, ArH), 7.14 (d, *J* = 7.9 Hz, 2H, Ar*H*), 4.38 (s, 2H, ArC*H*$_2$), 3.54 – 3.45 (m, 2H, C*H*$_2$), 2.28 (s, 3H, ArC*H*$_3$), 2.01 (t, *J* = 7.1 Hz, 2H, C*H*$_2$), 1.37 (s, 6H, (C*H*$_3$)$_2$).

^{13}C NMR (101 MHz, DMSO-*d*$_6$) δ 168.0, 163.7, 136.9, 136.0, 129.3, 128.0, 79.6, 72.3, 66.3, 30.2 26.9, 21.2.

HRMS (ESI/QTOF) m/z: $[M + Na]^+$ Calcd for $C_{15}H_{19}CsNaO_5^+$ 435.0179; Found 435.0183.

*4-((tert-butyldimethylsilyl)oxy)-2-methylbutan-2-ol (**26p**)*

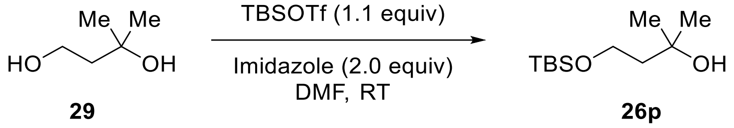

To a solution of 3-methylbutane-1,3-diol (**29**, 500 mg, 4.80 mmol, 1.00 equiv) and 1H-imidazole (654 mg, 9.60 mmol, 2.00 equiv) in *N,N*-dimethylformamide (25 mL), TBSOTf (1.4 g, 1.2 mL, 5.3 mmol, 1.1 equiv) was added dropwise. The reaction mixture was stirred at room temperature until TLC showed full conversion of the starting material. DCM and a 1:1 solution of brine and water were added, the layers were separated and the organic layer was washed with half brine (2x), dried over MgSO4 and solvent removed *in vacuo*. The crude was purified by flash chromatography (SiO_2, 5% EtOAc in pentane) affording 4-((*tert*-butyldimethylsilyl)oxy)-2-methylbutan-2-ol (**26p**, 950 mg, 4.35 mmol, 91% yield) as a pale yellow oil. The NMR data was collected and the compound was used in the next step without further analyses.

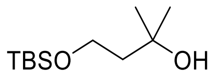

Rf (pentane:EtOAc 95:5) = 0.4.
^{1}H NMR (400 MHz, $CDCl_3$) δ: 3.91 (t, *J* = 5.8 Hz, 2H, OCH_2), 3.83 (bs, 1H, O*H*), 1.70 (t, *J* = 5.8 Hz , 2H, CH_2), 1.24 (s, 6H, $C(CH_3)_2$), 0.90 (s, 9H, $C(CH_3)_3$), 0.09 (s, 6H, $Si(CH_3)_2$).
^{13}C NMR (101 MHz, $CDCl_3$) δ 71.0, 61.1, 43.0, 29.4, 26.0, 18.2, -5.5.

*4-((tert-Butyldimethylsilyl)oxy)-2-methylbutan-2-yl ethyl oxalate (**28p**)*

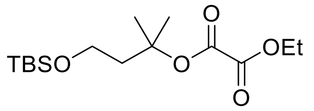

28p was synthesized following step 1 of *general procedure D* in THF (25 mL, 0.1 M) using 4-((*tert*-butyldimethylsilyl)oxy)-2-methylbutan-2-ol (**26p**, 500 mg, 2.30 mmol, 1.0 equiv), DMAP (28 mg, 0.23 mmol, 10 mol%), triethylamine (0.40 mL, 2.8 mmol, 1.2 equiv) and ethyl chloro-oxoacetate (0.30 mL, 2.8 mmol, 1.2 equiv). Column chromatography (SiO_2, 2% EtOAc in Pentane) afforded 4-((*tert*-butyldimethylsilyl)oxy)-2-methylbutan-2-yl ethyl oxalate (**28p**, 517 mg, 1.62 mmol, 71 %) as a yellow oil.

Rf (pentane:EtOAc 98:2) = 0.2.
^{1}H NMR (400 MHz, $CDCl_3$) δ 4.31 (q, *J* = 7.2 Hz, 2H, $COOCH_2$), 3.75 (t, *J* = 6.7 Hz, 2H, OCH_2), 2.09 (t, *J* = 6.7 Hz, 2H, CH_2), 1.57 (s, 6H, $C(CH_3)_2$), 1.36 (t, *J* = 7.2 Hz, 3H, $COOCH_3$), 0.88 (s, 9H, $C(CH_3)_3$), 0.05 (s, 6H, $Si(CH_3)_2$).
^{13}C NMR (101 MHz, $CDCl_3$) δ 158.7, 157.1, 86.4, 62.9, 59.0, 43.0, 26.3, 26.0, 18.4, 14.1, -5.3.
IR (ν_{max}, cm^{-1}): 2944 (m), 2891 (m), 2863 (m), 1744 (s), 1468 (m), 1323 (m), 1256 (m), 1190 (s), 1133 (s), 1098 (s).
HRMS (ESI/QTOF) m/z: $[M + Na]^+$ Calcd for $C_{15}H_{30}NaO_5Si^+$ 341.1755; Found 341.1752.

*Cesium 2-((4-((tert-butyldimethylsilyl)oxy)-2-methylbutan-2-yl)oxy)-2-oxoacetate (**3p**)*

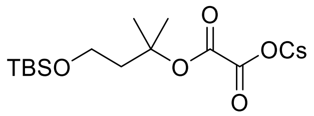

3p was synthesized following step 2 of *general procedure D* in THF (1.1 mL, 0.1 M) using 4-((*tert*-butyldimethylsilyl)oxy)-2-methylbutan-2-yl ethyl oxalate (**28p**, 350 mg, 1.10 mmol, 1.0 equiv) and 1 M aq. CsOH (1.1 mL, 1.1 mmol, 1.0 equiv). Cesium 2-((4-((*tert*-butyldimethylsilyl)oxy)-2-methylbutan-2-yl)oxy)-2-

oxoacetate (**3p**, 450 mg, 1.07 mmol, 97 %) was obtained as an off-white amorphous solid.

^{1}H NMR (400 MHz, DMSO) δ 3.65 (t, *J* = 7.2 Hz, 2H, OC*H*$_2$), 1.94 (t, *J* = 7.2 Hz, 2H, C*H*$_2$), 1.37 (s, 6H, C(C*H*$_3$)$_2$), 0.85 (s, 9H, C(C*H*$_3$)$_3$), 0.03 (s, 6H, Si(C*H*$_3$)$_2$).
^{13}C NMR (101 MHz, DMSO) δ 167.5, 163.3, 79.1, 58.7, 42.7, 26.5, 25.8, 17.8, -5.3.
HRMS (ESI/QTOF) m/z: [M + Na]$^+$ Calcd for $C_{13}H_{25}CsNaO_5Si^+$ 445.0418; Found 445.0418.

*4-((tert-butyldiphenylsilyl)oxy)-2-methylbutan-2-ol (**26q**)*

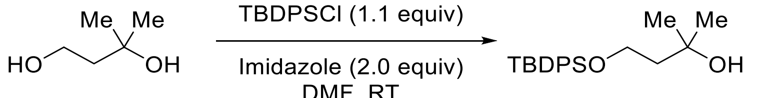

29 **26q**

To a solution of 3-methylbutane-1,3-diol (**29**, 500 mg, 4.80 mmol, 1.00 equiv) and 1H-imidazole (654 mg, 9.60 mmol, 2.00 equiv) in *N,N*-dimethylformamide (25.0 mL), TBDPSCl (1.45 g, 1.37 mL, 5.28 mmol, 1.10 equiv) was added dropwise. The reaction mixture was stirred at room temperature until TLC showed full conversion of the starting material. DCM and half brine were added, the layers were separated and the organic layer was washed with half brine (2x), dried over MgSO4 and solvent removed under vacuo. The crude product was purified by flash chromatography (SiO_2, 5% EtOAc in pentane) affording 4-((*tert*-butyldiphenylsilyl)oxy)-2-methylbutan-2-ol (**26q**, 1.64 g, 4.80 mmol, 100% yield) as a fain yellow oil. The NMR data was collected and the compound was used in the next step without further analyses.

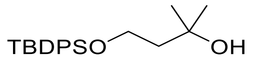

Rf (pentane:EtOAc 95:5) = 0.4.
^{1}H NMR (400 MHz, $CDCl_3$) δ: 7.72 – 7.65 (m, 4H, Ar*H*), 7.47 – 7.36 (m, 6H, Ar*H*), 3.90 (t, *J* = 5.8 Hz, 2H, OC*H*$_2$), 3.74 (bs, 1H, O*H*), 1.75 (t, *J* = 5.7 Hz, 2H, C*H*$_2$), 1.27 (s, 6H, C(C*H*$_3$)$_2$OH), 1.05 (s, 9H, C(C*H*$_3$)$_3$).
^{13}C NMR (101 MHz, $CDCl_3$) δ 135.7, 132.9, 130.03, 128.0, 71.1, 62.2, 43.2, 29.5, 26.9, 19.1.

*4-((tert-Butyldiphenylsilyl)oxy)-2-methylbutan-2-yl ethyl oxalate (**28q**)*

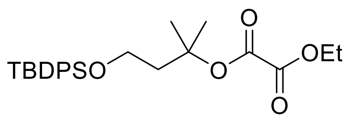

28q was synthesized following <u>step 1</u> of *general procedure D* in THF (30 mL, 0.1 M) using 4-((*tert*-butyldiphenylsilyl)oxy)-2-methylbutan-2-ol (**26q**, 1.00 g, 2.92 mmol, 1.0 equiv), DMAP (36 mg, 0.29 mmol, 10 mol%), triethylamine (0.50 mL, 3.5 mmol, 1.2 equiv) and ethyl chloro-oxoacetate (0.40 mL, 3.5 mmol, 1.2 equiv). Column chromatography (SiO_2, 2% EtOAc in Pentane) afforded 4-((*tert*-butyldiphenylsilyl)oxy)-2-methylbutan-2-yl ethyl oxalate (**28q**, 1.06g, 2.40 mmol, 82 %) as a pale yellow oil.

Rf (pentane:EtOAc 98:2) = 0.15.
^{1}H NMR (400 MHz, $CDCl_3$) δ: 7.69 – 7.64 (m, 4H, Ar*H*), 7.43 – 7.35 (m, 6H, Ar*H*), 4.27 (q, *J* = 7.1 Hz, 2H, CO$_2$C*H*$_2$CH$_3$), 3.78 (t, *J* = 6.7 Hz, 2H, OC*H*$_2$), 2.16 (t, *J* = 6.7 Hz, 2H, C*H*$_2$), 1.55 (s, 6H, C(C*H*$_3$)$_2$), 1.32 (t, *J* = 7.1 Hz, 3H, CO$_2$CH$_2$C*H*$_3$), 1.04 (s, 9H, C(C*H*$_3$)$_3$).
^{13}C NMR (101 MHz, $CDCl_3$) δ: 158.5, 157.0, 135.6, 133.6, 129.7, 127.7, 86.2, 62.8, 59.8, 42.6, 26.8, 26.2, 19.1, 13.9.
IR (ν_{max}, cm^{-1}): 3064 (w), 2939 (m), 2862 (m), 1743 (s), 1323 (m), 1190 (s), 1104 (s), 823 (m).
HRMS (ESI/QTOF) m/z: [M + Na]$^+$ Calcd for $C_{25}H_{34}NaO_5Si^+$ 465.2068; Found 465.2076.

*Cesium 2-((4-((tert-butyldiphenylsilyl)oxy)-2-methylbutan-2-yl)oxy)-2-oxoacetate (**3q**)*

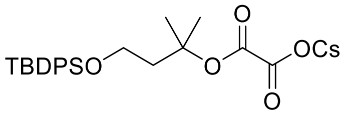

3q was synthesized following <u>step 2</u> of *general procedure D* in THF (1.1 mL, 0.1 M) using 4-((*tert*-butyldiphenylsilyl)oxy)-2-methylbutan-2-yl ethyl oxalate (**28q**, 500 mg, 1.13 mmol, 1.0 equiv) and 1 M aq. CsOH (1.1 mL, 1.1 mmol, 1.0 equiv). Cesium 2-((4-((*tert*-butyldiphenylsilyl)oxy)-2-methylbutan-2-yl)oxy)-2-oxoacetate (**3q**, 600 mg, 1.10 mmol, 97 %) was obtained as an off-white amorphous solid.

^{1}H NMR (400 MHz, DMSO) δ 7.65 – 7.58 (m, 4H, Ar*H*), 7.48 – 7.41 (m, 6H, Ar*H*), 3.74 (t, *J* = 7.1 Hz, 2H, OC*H*$_2$), 2.03 (t, *J* = 7.1 Hz, 2H, C*H*$_2$), 1.34 (s, 6H, C*H*$_3$), 0.99 (s, 9H, C(C*H*$_3$)$_3$).
^{13}C NMR (101 MHz, DMSO) δ 167.5, 163.2, 135.0, 133.2, 129.8, 127.9, 79.1, 59.9, 42.7, 26.7, 26.4, 18.7.
HRMS (ESI/QTOF) m/z: [M + H]$^+$ Calcd for $C_{23}H_{30}CsO_5Si^+$ 547.0912; Found 547.0908.

*Ethyl (2-methyl-4-((4-methylbenzyl)oxy)butan-2-yl) oxalate (**28w**)*

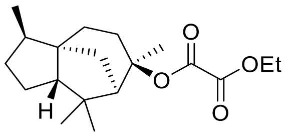

28w was synthesized following <u>step 1</u> of *general procedure D* in DCM (50 mL, 0.1 M) using Cedrol (**22w**, 1.00 g, 4.46 mmol, 1.0 equiv), DMAP (0.054 g, 0.45 mmol, 10 mol%), triethylamine (0.68 mL, 4.9 mmol, 1.1 equiv) and ethyl chloro-oxoacetate (0.55 mL, 4.9 mmol, 1.1 equiv).

Column chromatography (SiO_2, pentane:EtOAc 9:1 to 8:2) afforded ethyl (2-methyl-4-((4-methylbenzyl)oxy)butan-2-yl) oxalate (**28w**, 0.343 g, 0.1.06 mmol, 24%).

Rf (pentane:EtOAc 9:1) = 0.45.
^{1}H NMR (400 MHz, CDCl$_3$) δ 4.30 (q, *J* = 7.1 Hz, 2H, OC*H*$_2$CH$_3$), 2.46 – 2.40 (m, 1H, aliphatic-C*H* or C*H*$_2$), 2.17 (ddt, *J* = 13.6, 5.8, 1.7 Hz, 1H, aliphatic-C*H* or C*H*$_2$), 2.13 – 2.00 (m, 1H, aliphatic-C*H* or C*H*$_2$), 1.94 – 1.78 (m, 2H, aliphatic-C*H* or C*H*$_2$), 1.74 – 1.64 (m, 2H, aliphatic-C*H* or C*H*$_2$), 1.62 (d, *J* = 1.0 Hz, 3H, C*H*$_3$), 1.59 – 1.47 (m, 2H, aliphatic-C*H* or C*H*$_2$), 1.46 – 1.33 (m, 6H, aliphatic-C*H* or C*H*$_2$ + OCH$_2$C*H*$_3$), 1.32 – 1.23 (m, 1H, aliphatic-C*H* or C*H*$_2$), 1.18 (s, 3H, C*H*$_3$), 0.99 (s, 3H, C*H*$_3$), 0.84 (d, *J* = 7.1 Hz, 3H, CH$_2$C*H*$_3$).
^{13}C NMR (101 MHz, CDCl$_3$) δ 158.8, 157.1, 91.2, 62.8, 57.0, 56.8, 54.0, 43.7, 41.4, 41.2, 37.1, 33.0, 31.4, 28.5, 27.1, 25.5, 25.4, 15.6, 14.1.
IR (ν_{max}, cm^{-1}) 2990 (w), 2939 (w), 2876 (w), 1738 (s), 1373 (s), 1236 (s), 1186 (m), 1044 (s).
HRMS (ESI/QTOF) m/z: [M + Na]$^+$ Calcd for $C_{19}H_{30}NaO_4^+$ 345.2036; Found 345.2029.

*(-)-Cedrol derived cesium oxalate: cesium (2-methyl-4-((4-methylbenzyl)oxy)butan-2-yl)oxy-2-oxoacetate (**3w**)*

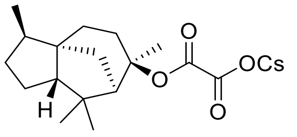

3w was synthesized following <u>step 2</u> of *general procedure D* in THF (0.78 mL, 0.1 M) using ethyl (2-methyl-4-((4-methylbenzyl)oxy)butan-2-yl) oxalate (**28w**, 0.250 g, 0.775 mmol, 1.0 equiv) and 1 M aq. CsOH (0.78 mL, 0.78 mmol, 1.0 equiv), affording cesium (2-methyl-4-((4-methylbenzyl)oxy)butan-2-yl)oxy-2-oxoacetate (**3w**, 0.330 g, 0.774 mmol, 100%). Amorphous white amorphous solid.

^{1}H NMR (400 MHz, DMSO-*d*$_6$) δ 2.34 (d, *J* = 5.1 Hz, 1H, aliphatic-C*H*), 1.90 – 1.71 (m, 4H, aliphatic-C*H*), 1.68 – 1.54 (m, 2H, aliphatic-CH or CH2), 1.45 (s, 4H, aliphatic-CH or CH2 + C*H*$_3$), 1.42 – 1.19 (m, 5H, aliphatic-CH or CH2), 1.16 (s, 3H, C*H*$_3$), 0.91 (s, 3H, C*H*$_3$), 0.81 (d, *J* = 7.1 Hz, 3H, CHC*H*$_3$).

^{13}**C NMR** (101 MHz, DMSO-d_6) δ 167.3, 163.5, 83.8, 56.4, 56.2, 53.6, 43.0, 40.7, 40.3, 36.4, 33.0, 30.6, 28.4, 27.3, 25.7, 24.9, 15.5.
HRMS (ESI/QTOF) m/z: $[M - Cs]^-$ Calcd for $C_{17}H_{25}O_4^-$ 293.1758; Found 293.1751.

*(R)-Ethyl (1-isopropyl-4-methylcyclohex-3-en-1-yl) oxalate (**28x**)*

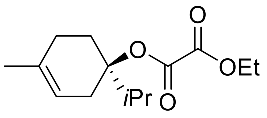

28x was synthesized following step 1 of *general procedure D* in THF (60 mL, 0.1 M) using (-)-terpinen-4-ol (**22x**, 1.00 mL, 6.00 mmol, 1.0 equiv), DMAP (73 mg, 0.60 mmol, 10 mol%), triethylamine (1.00 mL, 7.20 mmol, 1.2 equiv) and ethyl chloro-oxoacetate (0.80 mL, 7.2 mmol, 1.2 equiv).
Column chromatography (SiO_2, 2% EtOAc in Pentane) afforded (*R*)-ethyl (1-isopropyl-4-methylcyclohex-3-en-1-yl) oxalate (**28x**, 1.08 g, 4.25 mmol, 71 %) as a pale yellow oil.

Rf (pentane:EtOAc 98:2) = 0.4.
^{1}**H NMR** (400 MHz, $CDCl_3$) δ: 5.29 – 5.21 (m, 1H, C=C*H*), 4.29 (q, *J* = 7.1 Hz, 2H, CO_2CH_2), 2.71 (hept, *J* = 6.9 Hz, 1H, C*H*($CH_3)_2$), 2.54 – 2.43 (m, 2H, C*H*$_2$), 2.29 – 2.19 (m, 1H, C*H*$_2$), 2.11 – 1.98 (m, 1H, C*H*$_2$), 1.97 – 1.87 (m, 1H, C*H*$_2$), 1.78 – 1.68 (m, 1H, C*H*$_2$), 1.73 – 1.62 (m, 3H, C*H*$_3$), 1.34 (t, *J* = 7.1 Hz, 3H, $CO_2CH_2CH_3$), 0.95 (d, *J* = 6.9 Hz, 3H, $CH(CH_3)_2$), 0.94 (d, *J* = 6.9 Hz, 3H, $CH(CH_3)_2$).
^{13}**C NMR** (101 MHz, $CDCl_3$) δ: 158.9, 157.6, 133.8, 117.2, 91.1, 62.7, 32.7, 29.9, 27.9, 27.3, 23.3, 17.7, 17.2, 14.1.
IR (ν_{max}, cm^{-1}): 2973 (m), 2933 (m), 1738 (s), 1444 (m), 1380 (m), 1324 (m), 1180 (s), 1014 (m).
HRMS (ESI/QTOF) m/z: $[M + Na]^+$ Calcd for $C_{14}H_{22}NaO_4^+$ 277.1410; Found 277.1415.

*(-)Terpinen-4-ol derived cesium oxalate: cesium (R)-2-((1-isopropyl-4-methylcyclohex-3-en-1-yl)oxy)-2-oxoacetate (**3x**)*

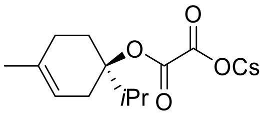

3x was synthesized following step 2 of *general procedure D* in THF (2.0 mL, 0.1 M) using (*R*)-ethyl (1-isopropyl-4-methylcyclohex-3-en-1-yl) oxalate (**28x**, 509 mg, 2.00 mmol, 1.0 equiv) and 1 M aq. CsOH (2.0 mL, 2.0 mmol, 1.0 equiv). Cesium (*R*)-2-((1-isopropyl-4-methylcyclohex-3-en-1-yl)oxy)-2-oxoacetate (**3x**, 661 mg, 1.85 mmol, 92 %) was obtained as an off-white amorphous solid.
^{1}**H NMR** (400 MHz, DMSO) δ: 5.20 – 5.16 (m, 1H, C=C*H*), 2.66 (p, *J* = 7.0 Hz, 1H, C*H*($CH_3)_2$), 2.40 – 2.31 (m, 1H, C*H*$_2$), 2.23 – 2.04 (m, 2H, C*H*$_2$), 2.04 – 1.90 (m, 1H, C*H*$_2$), 1.86 – 1.71 (m, 1H, C*H*$_2$), 1.60 (s, 3H, C*H*$_3$), 1.59 – 1.50 (m, 1H, C*H*$_2$), 0.86 (d, *J* = 7.0 Hz, 3H, $CH(CH_3)_2$), 0.84 (d, *J* = 7.0 Hz, 3H), $CH(CH_3)_2$).
^{13}**C NMR** (101 MHz, DMSO) δ: 168.1, 163.5, 132.8, 117.9, 83.3, 32.0, 29.5, 27.7, 26.7, 23.2, 17.4, 16.7.
HRMS (ESI/QTOF) m/z: $[M + H]^+$ Calcd for $C_{12}H_{18}CsO_4^+$ 359.0254; Found 359.0260.

2.6. Synthesis of oximes

2-(Aminooxy)-2-methylpropanoic acid hydrochloride was purchased from commercial sources (ABCR)

*2-(Aminooxy)propanoic acid hydrochloride (**33a**)*

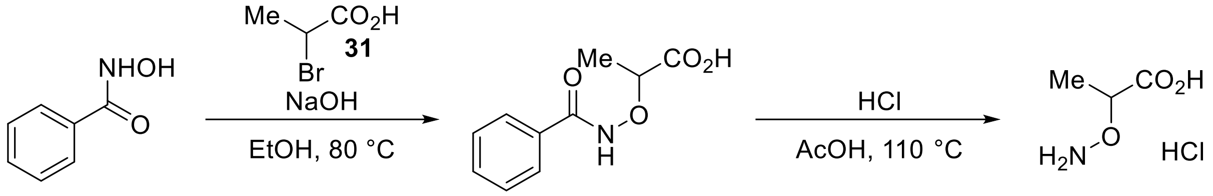

30 **32** **33**

Following a reported procedure,[20] N-hydroxybenzamide (**30**) (6.08 g, 44.3 mmol, 1.0 equiv) and finely ground NaOH (5.32 g, 133 mmol, 3.0 equiv) were suspended in absolute EtOH (66 mL). To the resulting thick, off-white suspension, 2-bromopropanoic acid (**31**) (4.1 mL, 44 mmol, 1.0 equiv) was added slowly via syringe under stirring. This resulted in the conversion of the homogeneous suspension into a pale brown solution, which was then heated to 80 °C. Once this temperature was reached, the mixture looked again as a homogeneous, off-white suspension, which was stirred overnight. The mixture was then concentrated under reduced pressure to provide a solid residue, which was dissolved in water (90 mL). The resulting aqueous solution was washed once with diethyl ether (100 mL) and then acidified by careful addition of aq. HCl (37 % w/w) until pH = 1. It was then extracted with EtOAc (3 x 100 mL) and the combined organic layers were dried over $MgSO_4$, filtered and concentrated under vacuum to provide an off-white solid. Recrystallization from hexane (50 mL) and EtOAc (100 mL) afforded 2-(benzamidooxy)propanoic acid (**32**) (7.08 g, 33.9 mmol, 76% yield) as a colorless solid. The compound was used directly in next step with no further analyses.

2-(Benzamidooxy)propanoic acid (**32**) (7.08 g, 33.8 mmol, 1.0 equiv) was suspended in acetic acid (20.5 mL). Aq. HCl (5.0 M; 68 mL, 34 mmol, 10 equiv) was then added and the mixture was heated to reflux (110 °C), which resulted in the formation of a pale yellow, clear solution. The latter was refluxed for 18 hours. It was then allowed to cool down to room temperature. This led to the precipitation of a crystalline solid (benzoic acid), which was filtered off. The resulting solution was stored at 4 °C overnight, which permitted the precipitation of a further amount of benzoic acid. Upon removal of the latter (4.13 g, 33.8 mmol, 100% yield) through filtration, the so-obtained clear solution was concentrated under vacuum. The resulting wet solid was further dried under vacuum at 60 °C for 3 hours. It was then refluxed in a mixture of EtOAc (30 mL) and EtOH (1.5 mL) for 20 minutes, filtered, washed with pentane, and dried in the air. 2-(Aminooxy)propanoic acid hydrochloride (**33a**) was obtained as a colorless solid (4.15 g, 29.3 mmol, 87% yield). The compound was used directly in next step with no further analyses.

2-(Aminooxy)-2-methylpropanoic acid hydrochloride (**33b**) and cyclobutanones were commercially available and purchased.

[20] H. Jiang, A. Studer, *Angew. Chem. Int. Ed.* **2017**, *56*, 12273–12276.

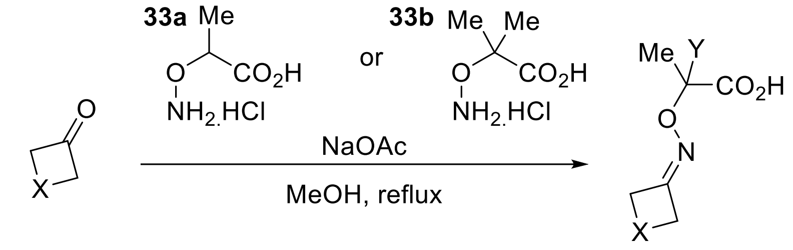

34a-c

9a, X = NBoc, Y = H
9b, X = CH(NHBOc) Y = Me
9c, X = O, Y = H

General procedure E:

Following a reported procedure,[21] a solution of cyclobutanone (**34**) (1.0 equiv) in MeOH (0.20 M) was treated with hydroxylamine **33a** or **33b** (1.2 equiv), sodium acetate (2.4 equiv) and heated to reflux until complete by TLC analysis (4.5 – 6.0 hours). The mixture was then allowed to cool to room temperature and aq. Na_2CO_3 (2.0 M) was added. In some cases, the addition of a small volume of water was necessary to achieve the complete dissolution of the solids. The resulting aqueous solution was extracted once with Et_2O and the organic layer was washed with aq. Na_2CO_3 (2.0 M; 2 x). The combined aqueous extracts were then acidified by careful addition of aq. HCl solution (30% v/v) until pH < 2, and extracted with DCM (3 x). The combined organic layers were dried over $MgSO_4$, filtered, and concentrated under vacuum to provide the pure product.

*2-(((1-(Tert-butoxycarbonyl)azetidin-3-ylidene)amino)oxy)propanoic acid (**9a**)*

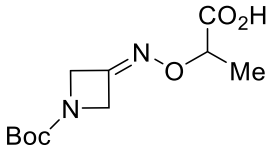

9a was synthesized following *general procedure E* using tert-butyl 3-oxoazetidine-1-carboxylate (**34a**, 342 mg, 2.00 mmol, 1.0 equiv) and 2-(aminooxy)propanoic acid hydrochloride (**33a**, 340 mg, 2.40 mmol, 1.2 equiv) and NaOAc (394 mg, 4.80 mmol, 2.4 equiv). 2-(((1-(tert-butoxycarbonyl)azetidin-3-ylidene)amino)oxy)propanoic acid (**9a**, 517 mg, 2.00 mmol, 100%) was obtained as an off-white amorphous solid.

^{1}H NMR (400 MHz, $CDCl_3$) δ 8.70 (bs, 1H, CO_2H) 4.72 – 4.58 (m, 5H, C*H*$_2$-N + C*H*-O), 1.50 – 1.47 (m, 3H, Me), 1.45 (bs, 9H, *t*Bu)..

^{13}C NMR (101 MHz, $CDCl_3$) δ 177.3, 156.3, 149.9, 80.9, 77.3, 58.3, 28.3, 16.6.

IR (ν_{max}, cm^{-1}) 3700 – 2800 (broad), 2981 (m), 2939 (m), 1705 (s), 1396 (s), 1134 (s), 1250 (m), 1828 (w), 960 (m)

HRMS (ESI/QTOF) m/z: $[M + Na]^+$ Calcd for $C_{11}H_{18}N_2NaO_5^+$ 281.1108; Found 281.1112.

*2-(((3-((tert-Butoxycarbonyl)amino)cyclobutylidene)amino)oxy)-2-methylpropanoic acid (**9b**)*

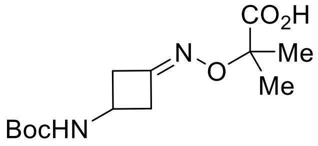

9b was synthesized following *general procedure E* using tert-butyl (3-oxocyclobutyl)carbamate (**34b**) (250 g, 1.28 mmol, 1.0 equiv) and 2-(aminooxy)-2-methylpropanoic acid hydrochloride (**33b**, 252 mg, 1.62 mmol, 1.2 equiv) and NaOAc (266 mg, 3.24 mmol, 2.4 equiv). 2--(((3-((*tert*-Butoxycarbonyl)amino)cyclobutylidene)amino)oxy)-2-methylpropanoic acid (**9b**, 360 mg, 1.23 mmol, 98%) was obtained as an white amorphous solid.

[21] E. M. Dauncey, S. P. Morcillo, J. J. Douglas, N. S. Sheikh, D. Leonori, *Angew. Chem. Int. Ed.* **2018**, *57*, 744–748.

^{1}H NMR (400 MHz, DMSO-d6) δ 12.40 (s, 1H, CO_2H), 7.38 (d, J = 7.4 Hz, 1H, NH), 4.03 (q, J = 7.4 Hz, 1H, CHNHBoc), 3.19 – 2.98 (m, 2H, CH_2), 2.83 – 2.66 (m, 2H, CH_2), 1.39 (s, 9H, *t*Bu), 1.36 (s, 3H, CMe_2), 1.35 (s, 3H, CMe_2).

^{13}C NMR (101 MHz, DMSO-d6) δ 175.0, 154.7, 153.3, 80.1, 78.0, 38.8, 28.2, 24.0. 1 carbon is not resolved.

Corresponds to literature data.[6]

*2-(((Oxetan-3-ylidene)amino)oxy)propanoic acid (**9c**)*

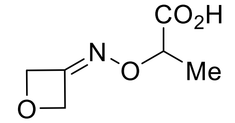

9c was synthesized following *general procedure E* using 3-oxetanone (**34c**, 72 mg, 1.0 mmol, 1.0 equiv) and 2-(aminooxy)propanoic acid hydrochloride (**33a**, 170 mg, 1.20 mmol, 1.2 equiv) and NaOAc (197 mg, 2.40 mmol, 2.4 equiv). 2-(((oxetan-3-ylidene)amino)oxy)propanoic acid (**9c**, 66 mg, purety 90%, 0.37 mmol, 37%) was obtained as an off-white amorphous solid.

^{1}H NMR (400 MHz, $CDCl_3$) δ 5.53 – 5.15 (m, 4H, OCH_2), 4.70 (q, J = 7.1 Hz, 1H, OCHMe), 1.50 (d, J = 7.1 Hz, 3H, Me). CO_2H is not detected.

^{13}C NMR (101 MHz, $CDCl_3$) δ 176.9, 155.1, 79.2, 78.8, 29.9, 16.8, 0.1.

IR (ν_{max}, cm^{-1}) 3556 – 2573 (broad), 3066 (w), 2939 (w), 2858 (w), 1720 (s), 1643 (w), 1442 (w), 1250 (m), 1300 (m), 1203 (m), 1138 (m), 1095 (m), 1041 (m), 976 (s), 864 (s).

HRMS (ESI/QTOF) m/z: $[M + H_{-1}]^-$ Calcd for $C_6H_8NO_4^-$ 158.0459; Found 158.0456.

2.7. Synthesis of potassium trifluoroboronates

*potassium 2,3-dihydro-1H-inden-2-yl-trifluoroborate (**11a**)*

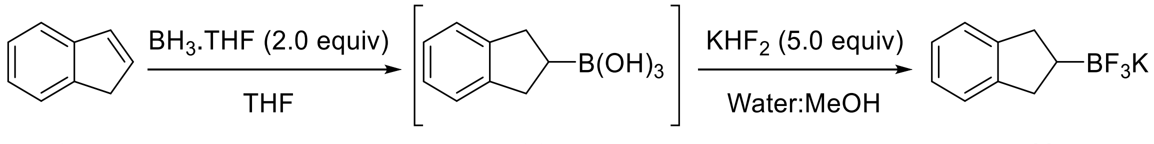

35 **36a** **11a**

Following a reported procedure,[22] a flame dried round bottom flask containing a solution of BH_3.THF (34.0 mL, 34.0 mmol, 1.00M, 2.00 equiv) in THF was cooled to 0 °C. A solution of 1H-indene (**35**) (1.98 mL, 17.0 mmol, 1.00 equiv) in tetrahydrofuran (3.40 mL) was added and the mixture was warm to rt and stirred for 2 h. Water (3.40 mL) was added dropwise and the mixture was stirred for 3 h at rt. The mixture was concentrated in vacuo to remove the solvents except water. Ethyl acetate (50 mL) was added to the suspension and the mixture was washed with a sat. sol. of $NaHCO_3$ (50 mL) and brine (50 mL). The organic layers were combined, dried over $MgSO_4.(H_2O)_2$ and concentrated in vacuo. The crude oil was used directly in next step. To a round bottom flask (PFA) containing a solution of potassium hydrogen fluoride (6.64 g, 85.0 mmol, 5.00 equiv) in water (25.0 mL) were added the crude boronic acid (**36a**) and methanol (34.0 mL). The mixture was stirred at rt open to air for 2 h. The

[22] Weng, W.-Z.; Liang, H.; Zhang, B. *Org. Lett.* **2018**, *20* (16), 4979–4983.

mixture was concentrated in vacuo, the wet solid obtained was further dried by co-evaporation with acetone (3 times). The resulting solid was diluted with acetone (30 mL) and was put on the rotavap at P_{atm} with the bath at 45 °C for 10 minutes. The solution was filtered with care to leave the remaining insoluble solid in the flask. This process was repeated 2 more times, the solution of acetone was concentrated in vacuo to 1/3 of the initial volume. The solution was left to cool to rt then Et_2O was added to induce precipitation (~40 mL). The solution was cooled to 0 °C and left for 15 min standing at this temperature. The solid was filtered, washed with Et2O and dried in vacuo to afford potassium 2,3-dihydro-1*H*-inden-2-yl-trifluoroborate (**11a**) (1.38 g, 6.14 mmol, 36% yield) as a white solid.

^{1}H NMR (400 MHz, Acetone) δ 7.07 (dd, *J* = 5.3, 3.3 Hz, 2H, Ar*H*), 6.95 (dd, *J* = 5.5, 3.1 Hz, 2H, Ar*H*), 2.75 (dd, *J* = 9.9, 3.6 Hz, 4H, C*H*$_2$), 1.29 (m, 1H, C*H*B).

^{13}C NMR (101 MHz, Acetone) δ 148.3, 125.6, 124.6, 36.8. One carbon is not resolved.

^{19}F NMR (376 MHz, Acetone) δ -146.34 (d, *J* = 95.0 Hz). Corresponds to the reported literature data.[23]

*potassium 2,3-dihydro-1H-inden-2-yl-trifluoroborate (**11c**)*

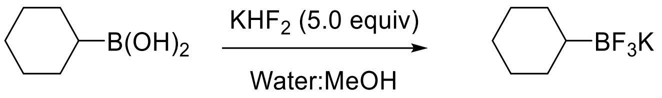

36c **11c**

Following a reported procedure,[24] in a round bottom flask (PFA), cyclohexyl boronic acid (**36c**, 5.00 g, 39.1 mmol, 1.00 equiv) was dissolved in methanol (100 mL). Aqueous potassium hydrogen fluoride (50 mL, 4.5 M, 225 mmol) was then added. The resulting white slurry was stirred at room temperature for 30 min, concentrated in vacuo and dissolved in hot acetone. The mixture was filtered, the filtrate was concentrated in vacuo and the residue recrystallized from a minimal amount of ether, to afford potassium cyclohexyl trifluoroborate (**11c**, 1.20 g, 6.3 mmol, 16%).

^{1}H NMR (400 MHz, DMSO) δ 1.63 – 1.51 (m, 3H, cyclic-C*H*$_2$), 1.51 – 1.40 (m, 2H, cyclic-C*H*$_2$), 1.19 – 0.95 (m, 3H, cyclic-C*H*$_2$), 0.88 (q, *J* = 12.4 Hz, 2H, cyclic-C*H*$_2$), -0.02 (bs, 1H, cyclic-C*H*-BF_3^-).

^{13}C NMR (101 MHz, DMSO) δ 31.2, 29.4, 28.7, 28.0. Corresponds to reported literature data.[24]

3. Photochemical experimental set-up

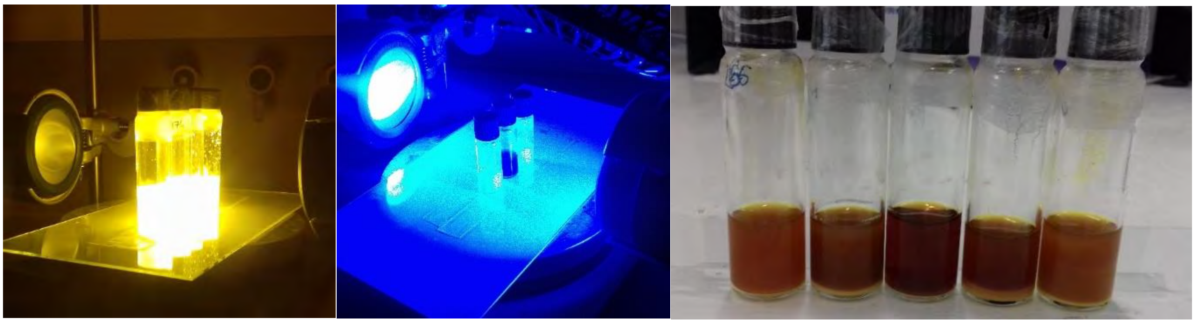

[23] Huang, H.; Zhang, G.; Gong, L.; Zhang, S.; Chen, Y. *J. Am. Chem. Soc.* **2014**, *136* (6), 2280–2283.
[24] Cazorla, C.; Métay, E.; Lemaire, M. *Tetrahedron* **2011**, *67*, 8615–8621.

Figure S1. Left: Scope scale reactions (photo taken with a filter applied to it). Middle: optimization scale. Right: Scope scale reactions after irradiation (with PC, same appearance for PC-free reactions without)

4. Optimization of the photomediated deoxygenation-alkynylation

4.1. Optimization studies method B (Excited state PhEBX **1a**)

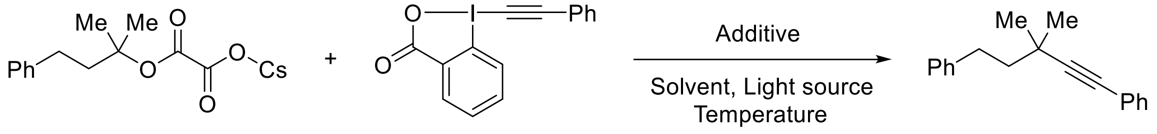

3a **1a** **4a**

Experimental procedure: an oven dried dram vial (2 mL), equipped with a magnetic stirrer, was charged with the solid components following table S1: cesium oxalate **3a**, PhEBX (**1a**), CsOBz, Cs_2CO_3. The reaction vial was sealed with a septum. After 3 vacuum/N2 cycles (backfilling with Ar on the last cycle), dry degassed (freeze pump thaw) solvent was added, followed by the liquid additive THF or γ-terpinene (as specified) and the septum was replaced with a screw cap under a flux of Ar.[25] The reactions were placed between 2 x 440 nm Kessil lamps (unless specified otherwise) at ca. 10 cm distance from both lamps (no ventilation, T = ca. 50 °C, with ventilation T = ca. 30-35°C as specified) and stirred under irradiation for 18 hours or 24 hours (as specified). The reaction was filtered through a small celite plug which was washed with CH_2Cl_2. The reaction crude was concentrated *in vacuo*, diluted with $CDCl_3$ and 1 equiv of CH_2Br_2 was added as internal standard for ^1H NMR analysis.

Table S1. Optimization of direct excitation strategy

Entry	1a (equiv)	Additive (equiv)	solvent (M)	T (°C)	λ (nm)	^{1}H NMR yield (%)
1	1.5	-	MeCN (0.1 M)	50	440	4
2	1.5	-	MeCN:H2O (0.1 M)	50	440	6
3	1.5	-	DMSO-d_6 (0.1 M)	50	440	4
4	1.5	-	MeOH (0.1 M)	50	440	17
5	1.5	-	DCM (0.1 M)	30-35	440	50
6[a]	1.5	-	DCM (0.1 M)	30-35	360[b]	50
7[b]	1.5	-	DCM (0.1 M)	30-35	460[c]	nd
8	2.5	-	DCM (0.1 M)	30-35	440	57
9[c]	2.5	-	DCM (0.1 M)	30-35	440	67
10[c,d]	2.5	-	DCM (0.1 M)	30-35	440	41
11[c,d]	2.5	-	DCM (0.1 M)	30-35	427	43
12[c,d]	2.5	-	DCM (0.1 M)	30-35	390	34
13[c,d]	2.5	-	DCM (0.1 M)	30-35	467	34
14[c]	2.5	Cs_2CO_3 (0.5)	DCM (0.1 M)	30-35	440	20
15[c]	2.5	CsOBz (1)	DCM (0.1 M)	30-35	440	10
16[c]	2.5	THF (2)	DCM (0.1 M)	30-35	440	nd
17[c]	2.5	γ-terpinene (2)	DCM (0.1 M)	30-35	440	50

[25] Use of a screw cap or crimp cap is of great importance to prevent solvent evaporation as the irradiation causes an increase in temperature. When using a test-tube/septum set-up, the latter would fly off within an hour of irradiation. As shown in the optimization section DCE is not as good a solvent as CH_2Cl_2.

[a]Reaction was performed in Rayonet reactor, [b]Reaction was performed with blue LED strips, [c]Reaction was run for 24 hours, [d]Reaction was performed with 1 Kessil lamp of the corresponding wavelength

4.2. Optimization studies of the 4CzIPN photocatalyzed deoxyalkynylation

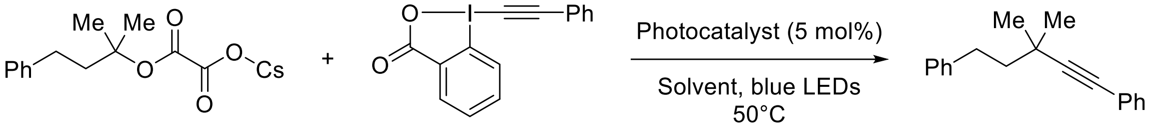

3a **1a** **4a**

Experimental procedure: an oven dried dram vial (2 mL), equipped with a magnetic stirrer, was charged with the solid components following table S2: cesium oxalate **3a**, PhEBX (**1a**), photocatalyst, additive (as specified). The reaction vial was sealed with a septum. After 3 vacuum/N2 cycles (backfilling with Ar on the last cycle), dry degassed (freeze pump thaw) solvent was added and the septum was replaced with a screw cap under a flux of Ar.[25] The reactions were placed between 2 x 440 nm Kessil lamps (at ca. 10 cm distance from both lamps (no ventilation, T = ca. 50 °C, with ventilation T = ca. 30-35°C as specified) and stirred under irradiation for 18 hours or 24 hours (as specified). The reaction was filtered through a small celite plug which was washed with CH_2Cl_2. The reaction crude was concentrated *in vacuo*, diluted with $CDCl_3$ and 1 equiv of CH_2Br_2 was added as internal standard for ^1H NMR analysis.

Table S2. Optimization of the photocatalytic strategy

**Entry**	**Solvent (M)**	**Photocatalyst**	**Stoichiometry (3a: 1a)**	**^1H NMR yield (%)**
**1**	DMSO (0.1 M)	**2a**	1:1.5	52
**2**	MeCN (0.1 M)	**2a**	1:1.5	40
**3**	DME/DMF (0.1 M)	**2a**	1:1.5	70
**4**	DME/DMF + 10 eq H_2O (0.1 M)	**2a**	1:1.5	55
**5**	THF (0.1 M)	**2a**	1:1.5	22
**6**	DCE (0.1 M)	**2a**	1:1.5	67
**7**	DCM (0.1 M)	**2a**	1:1.5	75
**8**	DCM (0.1 M)	**2b**	1:1.5	75
**9**	DCM (0.1 M)	$[Ir(dFCF_3ppy)_2(dtBBPY)]PF_6$	1:1.5	50
**10**	DCM (0.1 M)	DCA	1:1.5	55
**11**	DCM (0.1 M)	$MesAcr.BF_4$	1:1.5	53
**12**	DCM (0.1 M)	$[Ru(bpy)_3]PF_6$	1:1.5	<10% decomp
**13**	DCM (0.1 M)	$[Ru(bpz)_3]PF_6$	1:1.5	20
**14**	DCM (0.1 M)	**2a**	1.2:1	45
**15**	DCM (0.1 M)	**2a**	1:1	64
**16**	DCM (0.1 M)	**2a**	1::1.2	56
**17**	DCM (0.1 M)	**2a**	1:1.8	70
**18**	DCM (0.1 M)	**2a**	1:2.5	75
**19**	DCM (0.5 M)	**2a**	1:1.5	75
**20**	DCM (0.05 M)	**2a**	1:1.5	73
**21**	DCM (0.02 M)	**2a**	1:1.5	55
**22**[a]	DCM (0.1 M)	**2a**	1:1.5	65

[a]Performed with 0.3 equiv BIOAc as an additive

5. Photomediated Alkynylation Reactions:

5.1. General Procedures

5.1.1. General procedure F: Direct excitation of PhEBX for deoxy-alkynylation

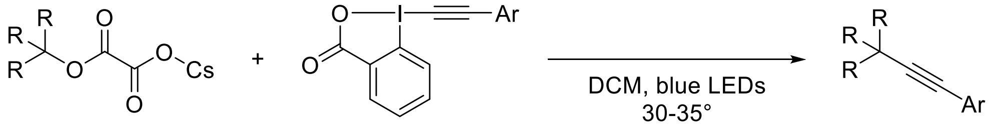

3a **1** **4a-x**

An oven dried (7.5 mL) dram vial equipped with a magnetic stirrer was charged with the cesium salt **3a-x** (0.30 mmol, 1.00 equiv) and ArEBX (**1**, 2.5 mmol, 2.5 equiv). The reaction vial was sealed with a septum. After 3 vacuum/N2 cycles (backfilling with Ar on the last cycle), dichloromethane (3.00 mL) was added and the septums were replaced with a screw cap under a flux of Ar.[25] The reactions were placed between 2 x 440 nm Kessil lamps at ca. 10 cm distance from both lamps (with ventilation, T = 30-35 °C) and stirred under irradiation for 24 hours. The reaction was filtered through a small celite plug which was washed with CH_2Cl_2. A solid deposit was prepared (ca. 2g SiO_2). The compound was purified by column chromatography (SiO_2, pentane:EtOAc).

5.1.2. General procedure G: Decarboxylative alkynylation

All carboxylic acids were commercial, bought from commercial sources and used as such in the reactions.

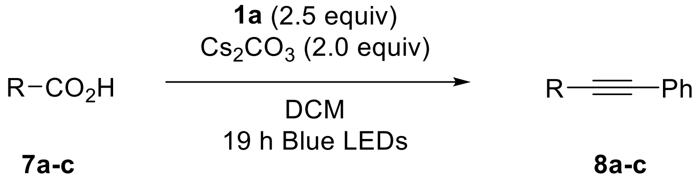

7a-c **8a-c**

Following a modified reported procedure,[26] a dram vial, equipped with a magnetic stirring bar, was charged with **1a** (261 mg, 0.750 mmol, 2.50 equiv), **7** (0.300 mmol, 1.00 equiv), cesium carbonate (196 mg, 0.600 mmol, 2.00 equiv) and 40 mg 4 Å molecular sieves. After 3 vacuum/nitrogen cycles, refilling with argon upon the last cycle, dichloromethane (4.5 mL, degassed by freeze-pump-thaw) was then added and the reaction was irradiated for 21 hours with 2 Kessil lamps PR160 440 nm. A solid deposit was then prepared of the crude on SiO_2 and was purified by column chromatography (SiO_2, Pentane:EtOAc).

---

[26] Zhou, Q.; Guo, W.; Ding, W.; Wu, X.; Chen, X.; Lu, L.; Xiao, W. *Angew. Chem. Int. Ed.* **2015**, *54*, 11196–11199.

5.1.3. General procedure H: Oxime fragmentation-alkynylation

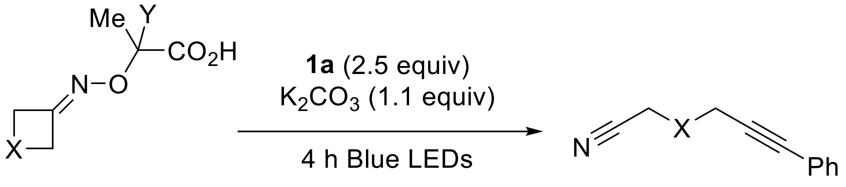

9a-c **10a-c**

Following a modified reported procedure,[27] a dram vial, equipped with a magnetic stirring bar, was charged with **1a** (261 mg, 750 µmol, 2.50 equiv), **9** (0.300 mmol, 1.00 equiv) and potassium carbonate (46 mg, 0.33 mmol, 1.10 equiv). After 3 vacuum/nitrogen cycles, refilling with argon upon the last cycle, 1,2-dichloroethane (2.00 mL, degassed by bubbling Ar) was then added and the reaction was irradiated for 3 h 50 min to 4 hours. A solid deposite of the crude was prepared and the compound was purified by column chromatography (SiO_2, pentane:EtOAc).

5.1.4. General procedure I: Deboronative alkynylation

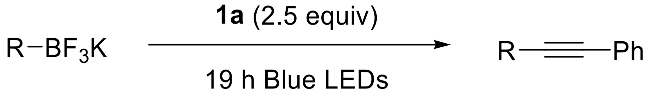

11a-c **12a-c**

Following a modified reported procedure,[23] an oven-dried (7.5 mL) dram vial equipped with a magnetic stirrer was charged with alkyl trifluoroborate (**11**, 0.30 mmol, 1.0 equiv), PhEBX (**1a**, 261 mg, 0.750 mmol, 2.50 equiv) and Na_2CO_3 (64 mg, 0.60 mmol, 2.0 equiv). The vial was sealed with a septum. After 3 vacuum/N_2 cycles, CH_2Cl_2 (1.5 mL) and water (1.5 mL) were added and the septum was replaced with a screw cap. The reaction was placed between 2 x 440 nm Kessil lamps at ca. 7 cm distance from both lamps with a fan and stirred under irradiation for 19 h. The layers were then separated and the aqueous layer was extracted with CH_2Cl_2 (3 x 10 mL). The combined organic layers were dried over $MgSO_4$ and the solvent was removed under reduced pressure. The crude was purified by flash chromatography (SiO_2,pentane:EtOAc) affording the corresponding alkyne.

5.1.5. Difunctionalization

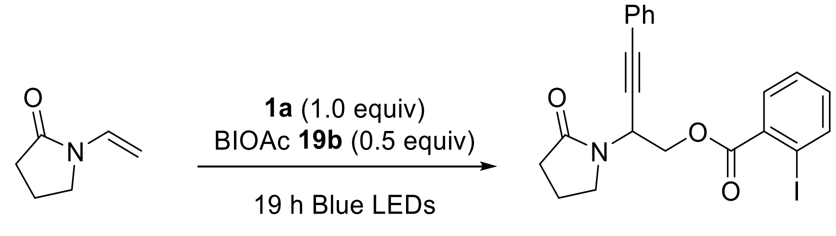

1.5 equiv
13

14

[27] Le Vaillant, F.; Garreau, M.; Nicolai, S.; Gryn'ova, G.; Corminboeuf, C.; Waser, J. *Chem. Sci.* **2018**, *9*, 5883-5889.

Following a modified reported procedure,[28] an oven dried dram vial, equipped with a magnetic stir bar was charged with **1a** (35 mg, 0.10 mmol, 1.0 equiv) and **19b** (15 mg, 0.050 mmol, 0.50 equiv). After 3 vacum/nitrogen cycles refilling with Ar on the last cycle, degassed CH_2Cl_2 (0.40 mL) was added followed by *N*-vinylpyrolidinone **13** (16.7 mg, 16.0 µL, 150 µmol, 1.50 equiv). The reaction was irradiated for 19 hours with 2 x 440 nm Kessil lamps. The reaction was concentrated in vacuo. An NMR sample of the crude was prepared with 1 equiv of CH_2Br_2 (7.0 µL, 0.10 mmol, 1 equiv) in CD_3CN. The ^{1}H NMR yield of **14** was determined using the signal at 5.53 ppm (dd, *J* = 8.6, 4.8 Hz, 1H, NC*H*CH$_2$O): 35%

^{1}H NMR (400 MHz, Acetonitrile-*d*$_3$) δ 8.06 (dd, *J* = 7.9, 1.2 Hz, 1H, Ar*H*), 7.81 (dd, *J* = 7.8, 1.7 Hz, 1H, Ar*H*), 7.58 – 7.47 (m, 3H, Ar*H* and Ph*H*), 7.47 – 7.33 (m, 3H, Ph*H*), 7.27 (td, *J* = 7.7, 1.8 Hz, 1H, Ar*H*), 5.53 (dd, *J* = 8.6, 4.8 Hz, 1H, NC*H*CH$_2$O), 4.64 (dd, *J* = 11.3, 8.6 Hz, 1H, NCHC*H*$_2$O), 4.50 (dd, *J* = 11.2, 4.8 Hz, 1H, NCHC*H*$_2$O), 3.75 – 3.52 (m, 2H, C*H*$_2$), 2.39 – 2.30 (m, 2H, C*H*$_2$), 2.12 – 2.02 (m, 2H, C*H*$_2$). Corresponds to the reported literature data.**Error! Bookmark not defined.**

5.1.6. Deaminative alkynylation

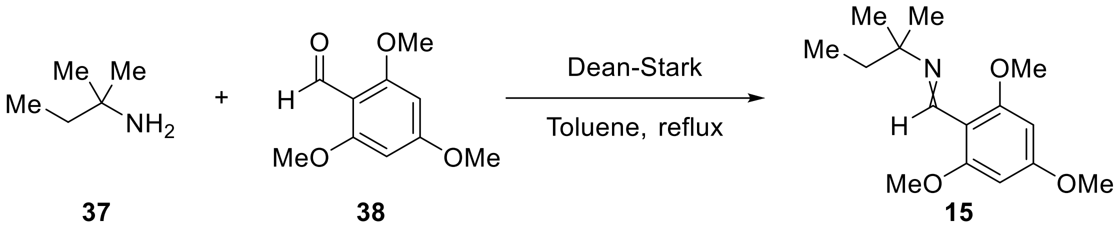

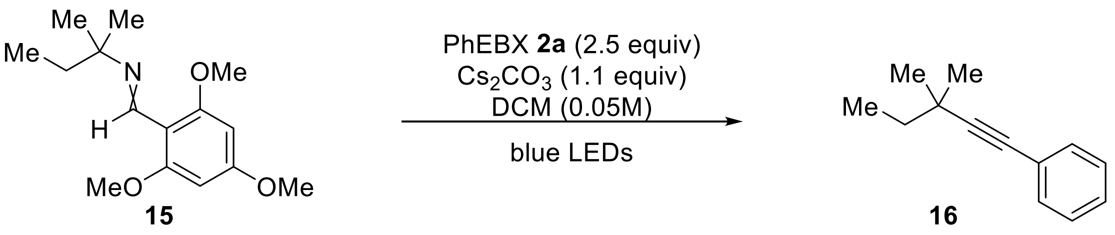

Following a slightly modified reported procedure,[29] a mixture of 2,4,6-trimethoxybenzaldehyde (**38**, 196 mg, 1.00 mmol, 1.00 equiv) and *tert*-amyl amine (**37**, 0.30 mL, 2.6 mmol, 2.6 equiv) in toluene (10 mL, 0.1 M) was heated in a Dean-Stark apparatus to reflux overnight. The reaction was then cooled, dried with Na_2SO_4, filtered, and evaporated affording crude imine *N*-*tert*-amyl-1-(2,4,6-trimethoxyphenyl)methanimine (**15**, 220 mg, 0.580 mmol, 85% pure, 71%) as a light yellow solid used directly in the next step.

An oven dried dram vial (7.5 mL) equipped with a magnetic stirrer was charged with crude imine **15** (80 mg, 85%wt 0.26 mmol, 1.0 equiv), PhEBX (**1a**, 261 mg, 0.750 mmol, 2.9 equiv) and cesium carbonate (108 mg, 0.330 mmol, 1.3 equiv). After 3 vacuum/N_2 cycles CH_2Cl_2 (6.0 mL) was added and the reaction was sealed with a screw cap under a flux of Ar. The reaction was then irradiated for 24 hours with 2 Kessil lamps (440 nm). The crude was purified by preparative TLC heptane:cyclohexane

[28] Amos, S. G. E.; Nicolai, S.; Waser, J. *Chem. Sci.* **2020**, *11*, 11274-11279
[29] Ashley, M. A.; Rovis, T. *J. Am. Chem. Soc.* **2020**, *142*, 18310–18316.

(1:1) affording 3,3-dimethylpent-1-ynylbenzene (**16**, 25.0 mg, 145 µmol, 57% yield) isolated with 2.25 eq of DCM and traces of 1,3-diphenylbutadiene.

^{1}H NMR (400 MHz, $CDCl_3$) δ 7.48 – 7.27 (m, 5H, Ar*H*), 1.52 (q, *J* = 7.5 Hz, 2H, CH_2CH_3), 1.26 (s, 6H, C(CH_3)$_2$), 1.05 (t, *J* = 7.5 Hz, 3H, CH_2CH_3).

^{13}C NMR (101 MHz, $CDCl_3$) δ 131.7, 128.9, 128.3, 127.5, 97.5, 80.5, 36.2, 32.2, 28.9, 9.9.

IR (ν_{max}, cm^{-1}) 2924 (m), 2970 (m), 3055 (m), 3105 (m), 2858 (m), 3101 (m), 1361 (m), 1323 (m), 1041 (m).

HRMS (nanochip-ESI/LTQ-Orbitrap) m/z: $[M + H]^+$ Calcd for $C_{13}H_{17}^+$ 173.1325; Found 173.1324.

5.1.7. HAT

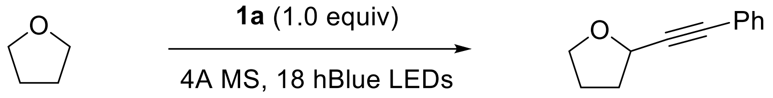

17 **18**

Following a modified reported procedure,[30] an oven dried (7.5 mL) dram vial equipped with a magnetic stirrer was charged with MS 4Å (20 mg) and PhEBX (**1a**, 70 mg, 0.20 mmol, 1.0 equiv). The reaction vial was sealed with a septum. After 3 vacuum/N2 cycles (backfilling with Ar on the last cycle), THF (**17**, 4.00 mL) was added and the septum is replaced with a screw cap under a flux of Ar. The reactions were placed between 2 x 460 nm Kessil lamps at ca. 10 cm distance from both lamps (no ventilation, T = ca. 50 °C) and stirred under irradiation for 18 hours. The reaction was filtered through a small celite plug which was washed with CH_2Cl_2. The reaction crude was concentrated in vacuo. An NMR sample of the crude was prepared with 1 equiv of CH_2Br_2 (14.0 µL, 0.200 mmol, 1 equiv) in $CDCl_3$. The ^{1}H NMR yield of **18** was determined using the signal at 4.81 (dd, *J* = 7.2, 5.2 Hz, 1H, CH_xO): 80%.

^{1}H NMR (400 MHz, $CDCl_3$) δ 7.45–7.41 (m, 2H, Ar*H*), 7.31–7.28 (m, 3H, Ar*H*), 4.81 (dd, *J* = 7.2, 5.2 Hz, 1H, CH_xO), 4.04–3.99 (m, 1H, CH_xO), 3.89–3.83 (m, 1H, CH_xO), 2.29–2.19 (m, 1H, CH_x), 2.15–2.04 (m, 2H, CH_x), 1.99–1.90 (m, 1H, CH_x). Corresponds to the reported literature data.**Error! Bookmark not defined.**

5.1.8. General procedure J: 4CzIPN catalyzed deoxyalkynylation

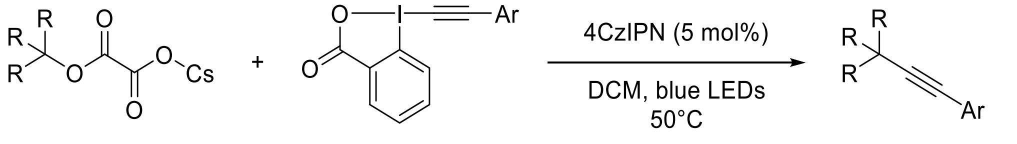

3a-q **1a-h** **4a-x**
3w and **3x**

An oven dried (7.5 mL) dram vial equipped with a magnetic stirrer was charged with the cesium salt **3a-x** (0.30 mmol, 1.00 equiv), the EBX reagent (**1**, 1.5 mmol, 1.5 equiv) and 4CzIPN (**2a**, 0.015 mmol, 5 mol%). The reaction vial was sealed with a septum. After 3 vacuum/N2 cycles (backfilling with Ar on the last cycle), dichloromethane (3.00 mL) was added and the septums were replaced with a screw

[30] Matsumoto, K.; Nakajima, M.; Nemoto, T. *J. Org. Chem.* **2020**, *85* (18), 11802–11811.

cap under a flux of Ar then the seal was wrapped with parafilm.**Error! Bookmark not defined.** The reactions were placed between 2 x 440 nm Kessil lamps at ca. 10 cm distance from both lamps (no ventilation, T = ca. 50 °C)[31] and stirred under irradiation for 15-18 hours. The reaction was filtered through a small celite plug which was washed with CH_2Cl_2. A solid deposit was prepared (ca. 2g SiO_2). The compound was purified by column chromatography (pentane:EtOAc).

5.2. Yields and characterization data

5.2.1. Deoxyalkynylated products

(3,3-dimethylpent-1-yne-1,5-diyl)dibenzene (4a)

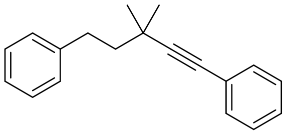

Direct excitation: **4a** was synthesized following *general procedure F* using cesium 2-(methyl-4-phenylbutan-2-yl)oxy-2-oxoacetate (**3a**, 0.110 g, 0.300 mmol, 1.00 equiv) and PhEBX (**1a**, 0.261 g, 0.750 mmol, 2.50 equiv) in degassed CH_2Cl_2 (3 mL, 0.1 M). Column chromatography (SiO_2, pentane) afforded (3,3-dimethylpent-1-yne-1,5-diyl)dibenzene (**4a**, 0.045 g, 0.18 mmol, 60%) as a slightly yellow oil.

Photocatalyzed: **4a** was synthesized following *general procedure J* using cesium 2-(methyl-4-phenylbutan-2-yl)oxy-2-oxoacetate (**3a**, 0.110 g, 0.300 mmol, 1.0 equiv), PhEBX (**1a**, 0.157 g, 0.450 mmol, 1.50 equiv), 4CzIPN (**2a**, 0.012 g, 1.5 µmol, 5 mol%) in degassed CH_2Cl_2 (3 mL, 0.1 M). Column chromatography (SiO_2, pentane) afforded (3,3-dimethylpent-1-yne-1,5-diyl)dibenzene (**4a**, 0.056 g, 0.23 mmol, 75%) as a slightly yellow oil.

Rf (pentane) = 0.4.
^{1}H NMR (400 MHz, $CDCl_3$) δ 7.45 – 7.39 (m, 2H, Ar*H*), 7.33 – 7.27 (m, 5H, Ar*H*), 7.26 – 7.16 (m, 3H, Ar*H*), 2.95 – 2.79 (m, 2H, ArC*H*$_2$), 1.86 – 1.75 (m, 2H, ArCH$_2$C*H*$_2$), 1.36 (s, 6H, C(C*H*$_3$)$_2$).
^{13}C NMR (101 MHz, $CDCl_3$) δ 142.9, 131.7, 128.6, 128.5, 128.3, 127.6, 125.8, 124.1, 97.0, 81.0, 45.7, 32.3, 32.0, 29.4.
IR (ν_{max}, cm^{-1}) 3084 (m), 3060 (m), 3027 (m), 2968 (m), 2945 (m), 2910 (m), 2866 (m), 2224 (m), 1946 (m), 1878 (m), 1804 (m), 1748 (m), 1491 (m), 1265 (m), 1070 (m), 755 (s), 740 (s), 690 (s).
HRMS (ESI/QTOF) m/z: $[M + Ag]^+$ Calcd for $C_{19}H_{20}Ag^+$ 355.0610; Found 355.0615.

(3,3-Dimethylbut-1-yn-1-yl)benzene (4b)

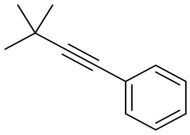

Direbt excitation: **4b** was synthesized following *general procedure F* using cesium (*tert*-butyl)oxy-2-oxoacetate (**3b**, 0.083 g, 0.30 mmol, 1 equiv) and PhEBX (**1a**, 0.261 g, 0.750 mmol, 2.50 equiv) in degassed CH_2Cl_2 (3 mL, 0.1 M). Column chromatography (SiO_2, pentane) afforded (3,3-dimethylbut-1-yne-1-yl)benzene (**4b**, 0.067 g, 49% purity 0.17 mmol, 57%) as a slightly yellow oil.

[31] The reaction temperature was measured with an internal thermometer on a model system using 5 mol% 4CzIPN in DCM.

Photocatalyzed: **4b** was synthesized following *general procedure J* using cesium *tert*-butoxyl-2-oxoacetate (**3b**, 0.083 g, 0.30 mmol, 1 equiv), PhEBX (**1a**, 0.157 g, 0.450 mmol, 1.50 equiv), 4CzIPN (**2a**, 0.012 g, 1.5 µmol, 5 mol%) in degassed CH_2Cl_2 (3 mL, 0.1 M). Column chromatography (SiO_2, pentane) afforded (3,3-dimethylbut-1-yn-1-yl)benzene (**4b**, 0.051 g, 85% purity, 0.27 mmol, 91%) as a colorless oil. The compound could be partially purified from 1,4-diphenylbuta-1,4-diyne (major impurity) by preparative TLC (SiO_2, glass plate, Heptane) allowing full characterization of **4b**.

Rf (pentane) = 0.8.
^{1}H NMR (400 MHz, $CDCl_3$) δ 7.43 – 7.33 (m, 2H, Ar*H*), 7.32 – 7.20 (m, 3H, Ar*H*), 1.32 (s, 9H, C(C*H*$_3$)$_3$).
^{13}C NMR (101 MHz, $CDCl_3$) δ 131.7, 128.3, 127.5, 124.2, 98.7, 79.1, 31.2, 28.1.
IR (ν_{max}, cm^{-1}) 3084 (m), 3054 (m), 2971 (m), 2903 (m), 2871 (m), 1780 (m), 1723 (m), 909 (s).
HRMS (APPI/LTQ-Orbitrap) m/z: $[M]^+$ Calcd for $C_{12}H_{14}^+$ 158.1090; Found 158.1093.

((1-Methylcyclopentyl)ethynyl)benzene (4c)

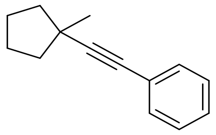

Direct excitation: **4c** was synthesized following *general procedure F* using cesium 2-((1-methylcyclopentyl)oxy)-2-oxoacetate (**3c**, 91 mg, 0.30 mmol, 1.0 equiv) and PhEBX (**1a**, 0.261 g, 0.75 mmol, 2.50 equiv) in degassed CH_2Cl_2 (3 mL, 0.1 M). Column chromatography (SiO_2, pentane) afforded ((1-methylcyclopentyl)ethynyl)benzene (**4c**, 57 mg, 42% purity, 0.16 mmol, 54%) with major impurity 1,4-diphenylbutadiyne.

Photocatalyzed: **4c** was synthesized following the *general procedure J* using cesium 2-((1-methylcyclopentyl)oxy)-2-oxoacetate (**3c**, 91 mg, 0.30 mmol, 1.0 equiv), PhEBX (**1a**, 157 mg, 0.450 mmol, 1.50 equiv) and 4CzIPN (**2a**, 12 mg, 0.015 mmol, 5 mol%). Column chromatography (SiO_2, Pentane) afforded ((1-methylcyclopentyl)ethynyl)benzene (**4c**, 39 mg, 0.20 mmol, 69%) as a pale yellow oil.
Rf (pentane) = 0.6.
^{1}H NMR (400 MHz, $CDCl_3$) δ: 7.39 - 7.36 (m, 2H, Ar*H*), 7.29 - 7.23 (m, 3H, Ar*H*), 2.01 - 1.95 (m, 2H, C*H*$_2$), 1.90 - 1.80 (m, 2H, C*H*$_2$) 1.75 - 1.66 (m, 2H, C*H*$_2$), 1.62 - 1.51 (m, 2H, C*H*$_2$), 1.35 (s, 3H, C*H*$_3$)
^{13}C NMR (101 MHz, $CDCl_3$) δ: 131.7, 128.3, 127.4, 124.4, 98.6, 79.6, 41.8, 38.5, 27.6, 24.5.
IR (ν_{max}, cm^{-1}): 3060 (m), 2960 (s), 2869 (m), 1742 (m), 1488 (m), 1451 (m), 1322 (m), 1186 (m).
HRMS (APPI/LTQ-Orbitrap) m/z: $[M]^+$ Calcd for $C_{14}H_{16}^+$ 184.1247; Found 184.1248.

2-(1-Methylcyclohexyl)ethynylbenzene (4d)

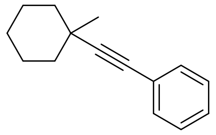

Direct excitation: **4d** was synthesized following *general procedure F* using cesium 2-(1-methylcyclohexan-1-yl)oxy-2-oxoacetate (**3d**, 95 mg, 0.30 mmol, 1.0 equiv) and PhEBX (**1a**, 0.261 g, 0.750 mmol, 2.50 equiv) in degassed CH_2Cl_2 (3.0 mL, 0.1 M). Column chromatography (SiO_2, pentane) afforded ((1-methylcyclohexyl)ethynyl)benzene (**4d**, 0.063 mg (55% purity), 0.18 mmol, 61%) with major impurity 1,4-diphenylbutadiyne.

Photocatalyzed: **4d** was synthesized following *general procedure J* using cesium 2-(1-methylcyclohexan-1-yl)oxy-2-oxoacetate (**3d**, 0.095 g, 0.30 mmol, 1 equiv), PhEBX (**1a**, 0.157 g, 0.450 mmol, 1.50 equiv), 4CzIPN (**2a**, 0.012 g, 1.5 µmol, 5 mol%) in degassed CH_2Cl_2 (3 mL, 0.1 M).

Column chromatography (SiO_2, pentane) afforded (1-methylcyclohexyl)enthynylbenzene (**4d**, 0.053 g (80% purity), 0.22 mmol, 72%) as a colorless oil. The compound could be partially purified from 1,4-diphenylbuta-1,4-diyne (major impurity) by preparative TLC (SiO_2, glass plate, Heptane) allowing full characterisation of **4d**.

Rf (pentane) = 0.7.
^{1}H NMR (400 MHz, $CDCl_3$) δ 7.45 – 7.37 (m, 2H, Ar*H*), 7.32 – 7.22 (m, 3H, Ar*H*), 1.84 – 1.55 (m, 8H, C*H*$_2$), 1.28 (s, 3H, C*H*$_3$), 1.27 – 1.09 (m, 2H, C*H*$_2$).
^{13}C NMR (101 MHz, $CDCl_3$) δ 131.7, 128.3, 127.5, 124.4, 96.9, 81.9, 39.7, 33.3, 30.4, 26.1, 23.6.
Consistent with the reported NMR data.[32]

1-Methyl-1-(phenylethynyl)cyclododecane (4e)

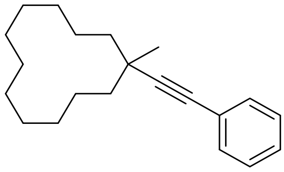

Direct excitation: **4e** was synthesized following *general procedure F* using cesium 2-(1-methylcyclododecan-1-yl)oxy-2-oxoacetate (**3e**, 151 mg (purity 80%), 0.300 mmol, 1.00 equiv) and PhEBX (**1a**, 0.261 g, 0.750 mmol, 2.50 equiv) in degassed CH_2Cl_2 (3.0 mL, 0.1 M). Column chromatography (SiO_2, pentane) afforded ((1-methylcyclododecyl)ethynyl)benzene (**4e**, 0.062 mg (47% purity), 0.11 mmol, 37%) with major impurity 1,4-diphenylbutadiyne.

Photocatalyzed: **4e** was synthesized following the *general procedure J* using cesium 2-((1-methylcyclododecyl)oxy)-2-oxoacetate (**3e**, 121 mg (purity 80%), 0.240 mmol, 1.00 equiv), PhEBX (**1a**, 157 mg, 0.450 mmol, 1.9 equiv) and 4CzIPN (**2a**, 12 mg, 0.015 mmol, 6 mol%). Column chromatography (SiO_2, Pentane) afforded 1-methyl-1-(phenylethynyl)cyclododecane (**4e**, 43 mg, 0.15 mmol, 63%) as a pale yellow oil.
Rf (pentane) = 0.6.
^{1}H NMR (400 MHz, $CDCl_3$) δ: 7.40 – 7.36 (m, 3H, Ar*H*), 7.29 – 7.23 (m, 2H, Ar*H*), 1.46 – 1.29 (m, 22H, C*H*$_2$), 1.23 (s, 3H, C*H*$_3$).
^{13}C NMR (101 MHz, $CDCl_3$) δ: 131.7, 128.2, 127.4, 124.4, 98.4, 80.6, 35.0, 34.4, 27.5, 26.6, 26.3, 22.7, 22.3, 19.9.
IR (ν_{max}, cm^{-1}): 3058 (w), 2936 (s), 2859 (m), 2226 (w), 1597 (w), 1479 (m), 1449 (m), 1273 (w).
HRMS (ESI/QTOF) m/z: $[M + Ag]^+$ Calcd for $C_{21}H_{30}Ag^+$ 389.1393; Found 389.1390.

4-Methyl-4-(phenylethynyl)tetrahydro-2*H*-pyran (4f)

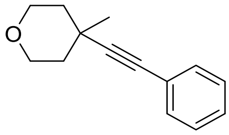

Direct excitation: **4f** was synthesized following *general procedure F* using ethyl (4-methyltetrahydro-2H-pyran-4-yl) oxalate (**3f**, 0.096 g, 0.30 mmol, 1.0 equiv) and PhEBX (**1a**, 0.261 g, 0.750 mmol, 2.50 equiv) in degassed CH_2Cl_2 (3.0 mL, 0.1 M). Column chromatography (SiO_2, pentane) afforded 4-methyl-4-(phenylethynyl)tetrahydro-2*H*-pyran (**4f**, 0.040 g (93% purity), 0.19 mmol, 62%).

---

[32] Gao, C.; Li, J.; Yu, J.; Yang, H.; Fu, H. *Chem. Commun.* **2016**, *52*, 7292–7294.

Photocatalyzed: **4f** was synthesized following the *general procedure J* using ethyl (4-methyltetrahydro-2H-pyran-4-yl) oxalate (**3f**, 96 mg, 0.30 mmol, 1.0 equiv), PhEBX (**1a**, 157 mg, 0.450 mmol, 1.50 equiv) and 4CzIPN (**2a**, 12 mg, 0.015 mmol, 5 mol%). Column chromatography (SiO_2, 1% to 5% EtOAc in Pentane) afforded, 4-methyl-4-(phenylethynyl)tetrahydro-2*H*-pyran (**4f**, 40 mg, purity: 94%, 0.19 mmol, 67%) as a colorless oil.
Rf (pentane:EtOAc 95:5) = 0.4.
^{1}H NMR (400 MHz, $CDCl_3$) δ: 7.44 – 7.39 (m, 2H, Ar*H*), 7.32 – 7.27 (m, 3H, Ar*H*), 3.90 – 3.77 (m, 4H, OCH_2), 1.76 – 1.68 (m, 2H, CH_2), 1.61 (ddd, *J* = 13.2, 11.2, 5.0 Hz, 2H, CH_2), 1.35 (s, 3H, CH_3).
^{13}C NMR (101 MHz, $CDCl_3$) δ: 131.7, 128.4, 127.9, 123.8, 94.7, 83.0, 65.4, 39.4, 31.1, 30.2.
IR (ν_{max}, cm^{-1}): 3058 (m), 2959 (s), 2857 (m), 1746 (m), 1492 (m), 1448 (m), 1174 (s), 1107 (s).
HRMS (APPI/LTQ-Orbitrap) m/z: $[M + H]^+$ Calcd for $C_{14}H_{17}O^+$ 201.1274; Found 201.1273.

1-(3,3-Dimethyl-5-phenylpent-1-yn-1-yl)-4-methylbenzene (**4g**)

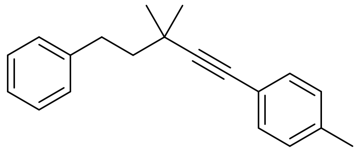

Direct excitation: **4g** was synthesized following *general procedure F* using cesium 2-(methyl-4-phenylbutan-2-yl)oxy-2-oxoacetate (**3a**, 0.110 g, 0.300 mmol, 1.00 equiv) and *p*TolEBX (**1b**, 0.271 g, 0.750 mmol, 2.50 equiv) in degassed CH_2Cl_2 (3 mL, 0.1 M). Column chromatography (SiO_2, pentane) afforded (3,3-dimethylpent-1-yne-1,5-diyl)dibenzene (**4g**, 0.055 g, 0.21 mmol, 70%) as a slightly yellow oil.

Photocatalyzed: **4g** was synthesized following *general procedure J* using cesium 2-(methyl-4-phenylbutan-2-yl)oxy-2-oxoacetate (**3a**, 0.110 g, 0.300 mmol, 1.0 equiv), *p*TolEBX (**1b**, 0.163 g, 0.450 mmol, 1.50 equiv), 4CzIPN (**2a**, 0.012 g, 1.5 µmol, 5 mol%) in degassed CH_2Cl_2 (3 mL, 0.1 M). Column chromatography (SiO_2, pentane) afforded (3,3-dimethylpent-1-yne-1,5-diyl)dibenzene (**4g**, 0.050 g, 0.19 mmol, 64%) as a colorless oil.

Rf (pentane) = 0.4
^{1}H NMR (400 MHz, $CDCl_3$) δ 7.34 – 7.26 (m, 4H, Ar*H*), 7.25 –7.15 (m, 3H, Ar*H*), 7.12 – 7.07 (m, 2H, Ar*H*), 2.96 – 2.76 (m, 2H, PhCH_2), 2.34 (s, 3H, ArCH_3), 1.85 – 1.74 (m, 2H, $PhCH_2CH_2$), 1.35 (s, 6H, $C(CH_3)_2$).
^{13}C NMR (101 MHz, $CDCl_3$) δ 143.0, 137.6, 131.6, 129.1, 128.6, 128.5, 125.8, 121.1, 96.2, 81.0, 45.8, 32.3, 32.0, 29.4, 21.6.
IR (ν_{max}, cm^{-1}) 2858 (m), 2924 (s), 2970 (s), 3028 (m), 1508 (s), 1454 (s), 818 (s), 741 (s).
HRMS (ESI/QTOF) m/z: $[M + H]^+$ Calcd for $C_{20}H_{23}^+$ 263.1794; Found 263.1793.

1-Methyl-1-(phenylethynyl)cycloheptane (**4i**)

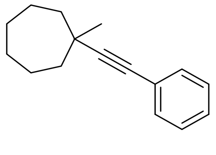

4i was synthesized following *general procedure J* using cesium 2-(1-methylcycloheptan-1-yl)oxy-2-oxoacetate (**3i**, 0.110 g, 0.300 mmol, 1.00 equiv), PhEBX (**1a**, 0.157 g, 0.450 mmol, 1.5 equiv), 4CzIPN (**2a**, 0.012 g, 1.5 µmol, 5 mol%) in degassed CH_2Cl_2 (3 mL, 0.1 M).
Column chromatography (SiO_2, pentane) afforded (3,3-dimethylpent-1-yne-1,5-diyl)dibenzene (**4i**, 0.068 g, 75% purity 0.22 mmol, 74%) as a colorless oil. The compound could be partially purified

from 1,4-diphenylbuta-1,4-diyne (major impurity) by preparative TLC (SiO_2, glass plate, Heptane) allowing full characterisation of **4i**.

Rf (pentane) = 0.7.
^{1}H NMR (400 MHz, $CDCl_3$) δ 7.42 – 7.39 (m, 2H, Ar*H*), 7.31 – 7.24 (m, 3H, Ar*H*), 1.95 – 1.84 (m, 2H, C*H₂*), 1.82 – 1.64 (m, 4H, C*H₂*), 1.64 – 1.56 (m, 2H, C*H₂*), 1.55 – 1.44 (m, 4H, C*H₂*), 1.29 (s, 3H, C*H₃*).
^{13}C NMR (101 MHz, $CDCl_3$) δ 131.7, 128.3, 127.4, 124.5, 98.1, 81.1, 42.3, 36.1, 31.5, 28.4, 24.0.
IR (ν_{max}, cm^{-1}) 3081 (w), 3054 (w), 2961 (m), 2925 (s), 2855 (m), 1598 (m), 1491 (m), 1460 (m), 1231 (m), 912 (m), 755 (s).
HRMS (APPI/LTQ-Orbitrap) m/z: [M]$^+$ Calcd for $C_{16}H_{20}^+$ 212.1560; Found 212.1558.

1-(Phenylethynyl)adamantane (4j)

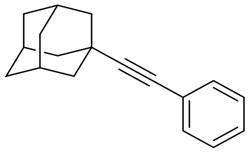

4j was synthesized following the *general procedure J* using cesium 2-(((1*S*,3*S*)-adamantan-1-yl)oxy)-2-oxoacetate (**3j**, 107 mg, 0.300 mmol, 1.00 equiv), PhEBX (**1a**, 157 mg, 0.450 mmol, 1.50 equiv) and 4CzIPN (**2a**, 12 mg, 0.015 mmol, 5 mol%).

Column chromatography (SiO_2, Pentane) afforded 1-(phenylethynyl)adamantane (**4j**, 20 mg, 0.080 mmol, 28%) as a pale yellow oil.

Rf (pentane) = 0.5.
^{1}H NMR (400 MHz, $CDCl_3$) δ: 7.44 – 7.32 (m, 2H, Ar*H*), 7.32 – 7.19 (m, 3H, Ar*H*), 2.07 – 1.97 (m, 3H, C*H*), 1.97 – 1.92 (m, 6H, C*H₂*), 1.75 – 1.69 (m, 6H, C*H₂*).
^{13}C NMR (101 MHz, $CDCl_3$) δ: 131.8, 128.2, 127.5, 124.2, 98.6, 79.5, 43.0, 36.6, 30.2, 28.2.
IR (ν_{max}, cm^{-1}): 3060 (w), 2912 (s), 2853 (m), 1491 (m), 1450 (m).
HRMS (nanochip-ESI/LTQ-Orbitrap) m/z: [M]$^+$ Calcd for $C_{18}H_{20}^+$ 236.1560; Found 236.1561.

(3-Ethyl-3-methylpent-1-yne-1,5-diyl)dibenzene (4k)

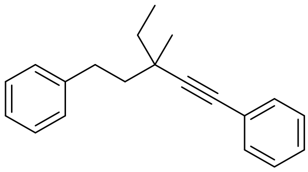

4k was synthesized following the *general procedure J* using cesium 2-((3-methyl-1-phenylpentan-3-yl)oxy)-2-oxoacetate (**3k**, 115 mg, 0.300 mmol, 1.00 equiv), PhEBX (**1a**, 157 mg, 0.450 mmol, 1.50 equiv) and 4CzIPN (**2a**, 12 mg, 0.015 mmol, 5 mol Column chromatography (SiO_2, Pentane) affording (3-ethyl-3-methylpent-1-yne-1,5-diyl)dibenzene (**4k**, 57 mg, 0.22 mmol, 72%) as a pale yellow oil.

Rf (pentane) = 0.3
^{1}H NMR (400 MHz, $CDCl_3$) δ: 7.44 – 7.41 (m, 2H, Ar*H*), 7.32 – 7.27 (m, 5H, Ar*H*), 7.25 – 7.22 (m, 2H, Ar*H*), 7.21 – 7.17 (m, 1H, Ar*H*), 2.90 – 2.77 (m, 2H, ArC*H₂*), 1.90 – 1.82 (m, 1H, C*H₂*), 1.76 – 1.63 (m, 2H, C*H₂*), 1.59 – 1.50 (m, 1H, C*H₂*), 1.30 (s, 3H, C*H₃*), 1.07 (t, *J* = 7.40 Hz, 3H, CH_2C*H₃*).
^{13}C NMR (101 MHz, $CDCl_3$) δ: 143.1, 131.8, 128.6, 128.5, 128.3, 127.6, 125.8, 124.3, 96.1, 82.2, 43.7, 36.2, 34.5, 31.9, 26.0, 9.5.
IR (ν_{max}, cm^{-1}): 3062 (w), 3031 (m), 2969 (m), 2929 (m), 2858 (w), 1599 (m), 1493 (m), 1454 (m).
HRMS (APPI/LTQ-Orbitrap) m/z: [M]$^+$ Calcd for $C_{20}H_{22}^+$ 262.1716; Found 262.1716.

(3,3,4-trimethylpent-1yn-1-yl)benzene (4l)

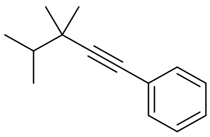

4l was synthesized following *general procedure J* using cesium (2,3-dimethylbutan-2-yl)oxy-2-oxoacetate (**3l**, 0.092 g, 0.30 mmol, 1.0 equiv), PhEBX (**1a**, 0.157 g, 0.450 mmol, 1.50 equiv), 4CzIPN (**2a**, 0.012 g, 1.5 µmol, 5 mol%) in degassed CH_2Cl_2 (3 mL, 0.1 M).

Column chromatography (SiO_2, pentane) afforded (3,3,4-trimethylpent-1-yn-1-yl)benzene (**4l**, 0.053 g, 85% purity, 0.21 mmol, 72%) as a colorless oil. The compound could be partially purified from 1,4-diphenylbuta-1,4-diyne (major impurity) by preparative TLC (SiO_2, glass plate, Heptane) allowing full characterisation of **4l**.

Rf (pentane) = 0.75.
^{1}H NMR (400 MHz, $CDCl_3$) δ 7.43 – 7.35 (m, 2H, Ar*H*), 7.32 – 7.22 (m, 3H, Ar*H*), 1.64 (hept, *J* = 6.8 Hz, 1H, C*H*(CH_3)$_2$), 1.25 (s, 6H, C(C*H*$_3$)$_2$), 1.03 (d, *J* = 6.8 Hz, 6H, CH(C*H*$_3$)$_2$).
^{13}C NMR (101 MHz, $CDCl_3$) δ 131.7, 128.3, 127.4, 124.4, 97.0, 81.0, 38.0, 35.6, 27.1, 18.5.
IR (ν_{max}, cm^{-1}) 3083 (m), 3055 (m), 2971 (s), 2939 (m), 2874 (m), 2228 (m), 1599 (m), 1489 (m), 1460 (m), 1369 (m), 1157 (m), 1061 (m), 911 (m), 755 (s), 691 (s).
HRMS (APPI/LTQ-Orbitrap) m/z: [M]$^+$ Calcd for $C_{14}H_{18}^+$ 186.1403; Found 186.1403.

1-(2,2-Dimethyl-4-phenylbut-3-yn-1-yl)-4-methoxybenzene (4m)

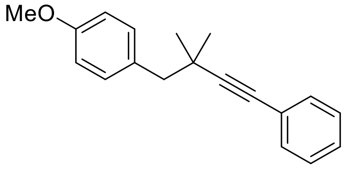

4m was synthesized following *general procedure J* using cesium (1-(4-methoxyphenyl)-2-methylpropan-2-yl)oxy-2-oxoacetate (**3m**, 0.115 g, 0.300 mmol, 1.00 equiv), PhEBX (**1a**, 0.157 g, 0.450 mmol, 1.50 equiv), 4CzIPN (**2a**, 0.012 g, 1.5 µmol, 5 mol%) in degassed CH_2Cl_2 (3 mL, 0.1 M).
Column chromatography (SiO_2, pentane:EtOAc 100:0 to 90:10) afforded 1-(2,2-dimethyl-4-phenylbut-3-yn-1-yl)-4-methoxybenzene (**4m**, 0.044 g, 0.17 mmol, 55%).

Rf (pentane:EtOAc 9:1) = 0.4.
^{1}H NMR (400 MHz, $CDCl_3$) δ 7.40 – 7.33 (m, 2H, Ar*H*), 7.31 – 7.21 (m, 5H, Ar*H*), 6.88 – 6.81 (m, 2H, Ar*H*), 3.80 (s, 3H, OC*H*$_3$), 2.74 (s, 2H, ArC*H*$_2$), 1.28 (s, 6H, (C*H*$_3$)$_2$).
^{13}C NMR (101 MHz, $CDCl_3$) δ 158.4, 131.7, 131.6, 130.7, 128.3, 127.6, 124.2, 113.2, 97.2, 81.7, 55.4, 48.4, 33.1, 29.1.
IR (ν_{max}, cm^{-1}) 3057 (m), 3034 (m), 2961 (m), 2933 (m), 2835 (m), 1786 (m), 1611 (m), 1512 (s), 1465 (m), 1302 (m), 1246 (s), 1177 (s), 1037 (s), 757 (s), 739 (s).
HRMS (ESI/QTOF) m/z: [M + Ag]$^+$ Calcd for $C_{19}H_{20}AgO^+$ 371.0560; Found 371.0552.

1-(2,2-Dimethyl-4-phenylbut-3-yn-1-yl)-2-fluorobenzene (4n)

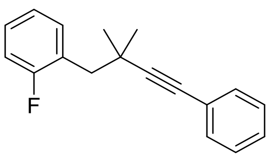

4n was synthesized following *general procedure J* using cesium (1-(2-fluorophenyl)-2-methylpropan-2-yl)oxy-2-oxoacetate (**3n**, 0.112 g, 0.300 mmol, 1.00 equiv), PhEBX (**1a**, 0.157 g, 0.450 mmol, 1.50 equiv), 4CzIPN (**2a**, 0.012 g, 1.5 µmol, 5 mol%) in degassed CH_2Cl_2 (3 mL, 0.1 M).
Column chromatography (SiO_2, pentane) afforded 1-(2,2-dimethyl-4-phenylbut-3-yn-1-yl)-2-fluorobenzene (**4n**, 0.023 g, 0.091 mmol, 30%).

Rf (pentane) = 0.4.
^{1}H NMR (400 MHz, CDCl3) δ 7.43 (td, *J* = 7.6, 1.9 Hz, 1H, Ar*H*), 7.38 – 7.36 (m, 1H, Ar*H*), 7.35 (d, *J* = 2.0 Hz, 1H, Ar*H*), 7.30 – 7.25 (m, 3H, Ar*H*), 7.25 – 7.17 (m, 1H, Ar*H*), 7.13 – 6.99 (m, 2H, Ar*H*), 2.87 (d, *J* = 1.5 Hz, 2H, ArC*H*$_2$), 1.33 (d, *J* = 1.0 Hz, 6H, C(CH_3)$_2$).
^{1}H NMR {^{19}F} δ 7.42 (dd, *J* = 7.6, 1.8 Hz, 1H, Ar*H*), 7.39 – 7.32 (m, 2H, Ar*H*), 7.31 – 7.18 (m, 4H, Ar*H*), 7.13 – 7.01 (m, 2H, Ar*H*), 2.87 (s, 2H, ArC*H*$_2$), 1.33 (s, 6H, C(C*H*$_3$)$_2$).
^{13}C NMR (101 MHz, $CDCl_3$) δ 161.7 (d, *J* = 245 Hz), 133.1 (d, *J* = 5 Hz), 131.6, 128.3 (d, *J* = 8 Hz), 128.3, 127.7, 125.5 (d, *J* = 16 Hz), 124.1, 123.5 (d, *J* = 4 Hz), 115.2 (d, *J* = 23 Hz), 96.7, 81.5, 41.3, 33.3, 29.1.
^{19}F NMR (376 MHz, $CDCl_3$) δ -116.1.

IR (ν_{max}, cm^{-1}) 3061 (w), 2969 (w), 2925 (w), 1489 (m), 1488 (m), 1467 (m), 1280 (m), 1183 (m), 752 (s), 721 (m).
HRMS (APPI/LTQ-Orbitrap) m/z: [M]$^+$ Calcd for $C_{18}H_{17}F^+$ 252.1309; Found 252.1308.

1-(((3,3-Dimethyl-5-phenylpent-4-yn-1-yl)oxy)methyl)-4-methylbenzene (4o)

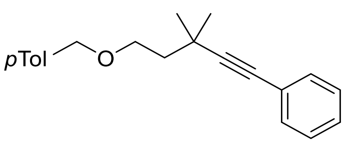

4o was synthesized following *general procedure J* using cesium (2-methyl-4-((4-methylbenzyl)oxy)butan-2-yl)oxy-2-oxoacetate (**3o**, 0.124 g, 0.300 mmol, 1.00 equiv), PhEBX (**1a**, 0.157 g, 0.450 mmol, 1.50 equiv), 4CzIPN (**2a**, 0.012 g, 1.5 µmol, 5 mol%) in degassed CH_2Cl_2 (3 mL, 0.1 M).
Column chromatography (SiO_2, pentane:EtOAc 100:0 to 80:20) afforded 1-(((3,3-dimethyl-5-phenylpent-4-yn-1-yl)oxy)methyl)-4-methylbenzene (**4o**, 0.049 g, 0.17 mmol, 56%).

Rf(pentane:EtOAc 8:2) = 0.5.
^{1}H NMR (400 MHz, $CDCl_3$) δ 7.37 – 7.29 (m, 2H, Ar*H*), 7.30 – 7.21 (m, 5H, Ar*H*), 7.17 – 7.11 (m, 2H, Ar*H*), 4.50 (s, 2H, ArC*H*$_2$), 3.74 (dd, *J* = 7.6, 6.9 Hz, 2H, C*H*$_2$), 2.34 (s, 3H, ArC*H*$_3$), 1.89 – 1.81 (m, 2H, C*H*$_2$), 1.32 (s, 6H, C(C*H*$_3$)$_2$).
^{13}C NMR (101 MHz, $CDCl_3$) δ 137.3, 135.6, 131.7, 129.2, 128.3, 127.9, 127.6, 124.0, 96.7, 80.8, 73.0, 68.2, 42.6, 30.6, 29.9, 21.3.
IR (ν_{max}, cm^{-1}) 3052 (m), 3033 (m), 2969 (m), 2907 (m), 2863 (m), 1960 (w), 1900 (w), 1715 (w), 1598 (m), 1490 (m), 1443 (m), 1361 (m), 1096 (s), 802 (s), 754 (s).
HRMS (APPI/LTQ-Orbitrap) m/z: [M]$^+$ Calcd for $C_{21}H_{24}O^+$ 292.1822; Found 292.1818.

tert-Butyl((3,3-dimethyl-5-phenylpent-4-yn-1-yl)oxy)dimethylsilane (4p)

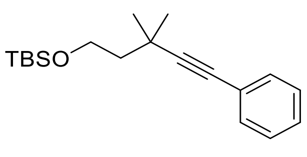

4p was synthesized following the *general procedure J* using cesium 2-((4-((*tert*-butyldimethylsilyl)oxy)-2-methylbutan-2-yl)oxy)-2-oxoacetate (**3p**, 127 mg, 0.300 mmol, 1.00 equiv), PhEBX (**1a**, 157 mg, 0.450 mmol, 1.50 equiv) and 4CzIPN (**2a**, 12 mg, 0.015 mmol, 5 mol%).
Column chromatography (SiO_2, 5% DCM in Pentane) afforded (3- *tert*-butyl((3,3-dimethyl-5-phenylpent-4-yn-1-yl)oxy)dimethylsilane (**4p**, 55 mg, 0.18 mmol, 61%) as a yellow oil.

Rf (pentane:DCM 95:5) = 0.4.
^{1}H NMR (400 MHz, $CDCl_3$) δ: 7.39 – 7.34 (m, 2H, Ar*H*), 7.30 – 7.25 (m, 3H, Ar*H*), 3.90 (t, *J* = 7.5 Hz, 2H, OC*H*$_2$), 1.76 (t, *J* = 7.5 Hz, 2H, C*H*$_2$), 1.31 (s, 6H, C(C*H*$_3$)$_2$), 0.91 (s, 9H, C(C*H*$_3$)$_3$), 0.08 (s, 6H, Si(C*H*$_3$)$_2$).
^{13}C NMR (101 MHz, $CDCl_3$) δ: 131.7, 128.3, 127.6, 124.1, 96.8, 80.7, 61.1, 45.8, 30.5, 29.9, 26.1, 18.5, -5.1.
IR (ν_{max}, cm^{-1}): 3668 (w), 2962 (s), 2901 (s), 1467 (m), 1393 (m), 1254 (m), 1092 (s), 1057 (s).
HRMS (nanochip-ESI/LTQ-Orbitrap) m/z: [M + H]$^+$ Calcd for $C_{19}H_{31}OSi^+$ 303.2139; Found 303.2137.

tert-Butyl((3,3-dimethyl-5-phenylpent-4-yn-1-yl)oxy)diphenylsilane (4q)

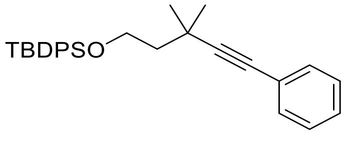

4q was synthesized following the *general procedure J* using cesium 2-((4-((*tert*-butyldiphenylsilyl)oxy)-2-methylbutan-2-yl)oxy)-2-oxoacetate (**3q**, 164 mg, 0.300 mmol, 1.00 equiv), PhEBX (**1a**, 157 mg, 0.450 mmol, 1.50 equiv) and 4CzIPN (**2a**, 12 mg, 0.015 mmol, 5 mol%).
Column chromatography (SiO_2, 5% DCM in Pentane) afforded *tert*-butyl((3,3-dimethyl-5-phenylpent-4-yn-1-yl)oxy)diphenylsilane (**4q**, 53 mg, 0.12 mmol, 41%) as a yellow oil.

Rf (pentane:DCM, 95:5) = 0.4.
^{1}H NMR (400 MHz, $CDCl_3$) δ: 7.74 – 7.64 (m, 4H, Ar*H*), 7.44 – 7.31 (m, 6H, Ar*H*), 7.29 – 7.21 (m, 5H, Ar*H*), 3.96 (dd, *J* = 7.6, 6.8 Hz, 2H, OCH_2), 1.82 (dd, *J* = 7.6, 6.8 Hz, 2H, CH_2), 1.27 (s, 6H, C(CH_3)$_2$), 1.05 (s, 9H, C(CH_3)$_3$).
^{13}C NMR (101 MHz, $CDCl_3$) δ: 135.7, 134.1, 131.7, 129.7, 128.2, 127.8, 127.6, 124.0, 96.7, 80.8, 62.0, 45.5, 30.5, 30.0, 27.0, 19.3.
IR (ν_{max}, cm^{-1}): 3668 (m), 3061 (m), 2966 (s), 2935 (s), 1478 (m), 1392 (m), 1258 (m), 1084 (s).
HRMS (nanochip-ESI/LTQ-Orbitrap) m/z: [M + Na]$^+$ Calcd for $C_{29}H_{34}NaOSi^+$ 449.2271; Found 449.2269.

1-(3,3-Dimethyl-5-phenylpent-1-yn-1-yl)-3-fluorobenzene (**4r**)

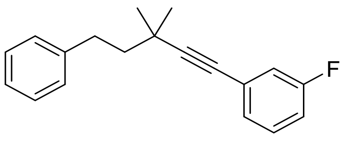

4r was synthesized following *general procedure J* using cesium 2-(methyl-4-phenylbutan-2-yl)oxy-2-oxoacetate (**3a**, 0.110 g, 0.300 mmol, 1 equiv), mFPhEBX (**1d**, 0.164 g, 0.450 mmol, 1.50 equiv), 4CzIPN (**2a**, 0.012 g, 1.5 µmol, 5 mol%) in degassed CH_2Cl_2 (3 mL, 0.1 M).
Column chromatography (SiO_2, pentane) afforded 1-(3,3-dimethyl-5-phenylpent-1-yn-1-yl)-3-fluorobenzene (**4r**, 0.045 g, 0.17 mmol, 56%).

Rf (pentane) = 0.5.
^{1}H NMR (400 MHz, $CDCl_3$) δ 7.34 – 7.15 (m, 7H, Ar*H*), 7.10 (ddd, *J* = 9.6, 2.7, 1.4 Hz, 1H, Ar*H*), 6.98 (tdd, *J* = 8.3, 2.7, 1.2 Hz, 1H, Ar*H*), 2.87 – 2.79 (m, 2H, ArCH_2), 1.84 – 1.75 (m, 2H, CH_2), 1.35 (s, 6H, C(CH_3)$_2$).
^{13}C NMR (101 MHz, $CDCl_3$) δ 162.4 (d, *J* = 245.8 Hz), 142.6, 129.7 (d, *J* = 8.7 Hz), 128.4, 127.5 (d, *J* = 2.9 Hz), 126.1 – 125.3 (m), 118.4 (d, *J* = 22.5 Hz), 114.8 (d, *J* = 21.1 Hz), 98.0, 79.8, 45.4, 32.1, 31.9, 29.1. 2 carbons are not resolved.
^{19}F NMR (376 MHz, $CDCl_3$) δ -113.5 (d, *J* = 4.5 Hz).
IR (ν_{max}, cm^{-1}) 3087 (m), 3062 (m), 2972 (s), 2937 (s), 2911 (s), 1608 (s), 1580 (s), 1075 (s), 1056 (s), 909 (s), 873 (s), 784 (s).
HRMS (APPI/LTQ-Orbitrap) m/z: [M]$^+$ Calcd for $C_{19}H_{19}F^+$ 266.1465; Found 266.1473.

1-(3,3-Dimethyl-5-phenylpent-1-yn-1-yl)-4-(trifluoromethyl)benzene (**4s**)

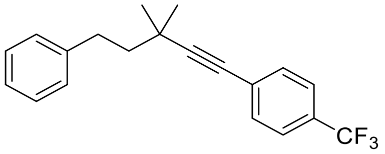

4s was synthesized following *general procedure J* using cesium 2-(methyl-4-phenylbutan-2-yl)oxy-2-oxoacetate (**3a**, 0.110 g, 0.300 mmol, 1 equiv), *p*CF_3PhEBX (**1e**, 0.187 g, 0.450 mmol, 1.50 equiv), 4CzIPN (**2a**, 0.012 g, 1.5 µmol, 5 mol%) in degassed CH_2Cl_2 (3 mL, 0.1 M).
Column chromatography (SiO_2, pentane) afforded 1-(3,3-dimethyl-5-phenylpent-1-yn-1-yl)-4-(trifluoromethyl)benzene (**4s**, 0.055 g, 0.17 mmol, 58%).

Rf (pentane) = 0.4.
^{1}H NMR (400 MHz, $CDCl_3$) δ 7.58 – 7.46 (m, 4H, Ar*H*), 7.34 – 7.17 (m, 5H, Ar*H*), 2.88 – 2.79 (m, 2H, ArCH_2), 1.85 – 1.77 (m, 2H, CH_2), 1.36 (s, 6H, C(CH_3)$_2$).
^{13}C NMR (101 MHz, $CDCl_3$) δ 142.7, 132.0, 128.5 (m), 125.9, 125.2 (d, *J* = 3.9 Hz), 99.8, 80.0, 45.5, 32.3, 32.1, 29.2. 4 carbons not resolved.
^{19}F NMR (376 MHz, $CDCl_3$) δ -62.7.
IR (ν_{max}, cm^{-1}) 3028 (w), 2975 (w), 2940 (m), 2859 (m), 2822 (w), 2239 (w), 1617 (m), 1505 (m), 1324 (s), 1168 (m), 1130 (s), 1066 (s), 910 (s), 766 (m), 743 (s).
HRMS (ESI/QTOF) m/z: [M + Ag]$^+$ Calcd for $C_{20}H_{19}AgF_3^+$ 423.0484; Found 423.0479.

1-(3,3-Dimethyl-5-phenylpent-1-yn-1-yl)-4-bromobenzene (4t)

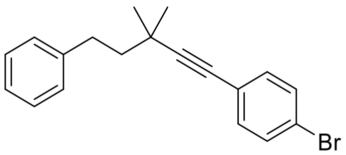

4t was synthesized following *general procedure J* using cesium 2-(methyl-4-phenylbutan-2-yl)oxy-2-oxoacetate (**3a**, 0.110 g, 0.300 mmol, 1.00 equiv), pBrPhEBX (**1f**, 0.192 g, 0.450 mmol, 1.50 equiv), 4CzIPN (**2a**, 0.012 g, 1.5 µmol, 5 mol%) in degassed CH_2Cl_2 (3 mL, 0.1 M).

Column chromatography (SiO_2, pentane) afforded 1-(3,3-dimethyl-5-phenylpent-1-yn-1-yl)-4-bromobenzene (**4t**, 0.044 g, 0.13 mmol, 45%).

Rf (pentane) = 0.3.

^{1}H NMR (400 MHz, $CDCl_3$) δ 7.45 – 7.38 (m, 2H, Ar*H*), 7.33 – 7.25 (m, 2H, Ar*H*), 7.29 – 7.21 (m, 3H, Ar*H*), 7.25 – 7.14 (m, 2H, Ar*H*), 2.87 – 2.78 (m, 2H, ArC*H*$_2$), 1.83 – 1.74 (m, 2H, C*H*$_2$), 1.34 (s, 6H, C(C*H*$_3$)$_2$).

^{13}C NMR (101 MHz, $CDCl_3$) δ 142.8, 133.2, 131.5, 128.5, 125.9, 123.1, 121.7, 98.3, 80.1, 45.5, 32.3, 32.1, 29.3. 1 carbon is not resolved.

IR (ν_{max}, cm^{-1}) 3086 (m), 3062 (m), 3026 (m), 2968 (m), 2920 (m), 2861 (m), 1485 (s), 1469 (m), 1312 (m), 1265 (m), 1070 (s), 1011 (s), 823 (s), 745 (s), 700 (s).

HRMS (ESI/QTOF) m/z: $[M + Ag]^+$ Calcd for $C_{19}H_{19}Ag^{79}Br^+$ 432.9716; Found 432.9707.

1-(3,3-dimethyl-5-phenylpent-1-yn-1-yl)-2-bromobenzene (4u)

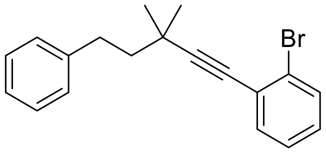

4u was synthesized following *general procedure J* using cesium 2-(methyl-4-phenylbutan-2-yl)oxy-2-oxoacetate (**3a**, 0.110 g, 0.300 mmol, 1.00 equiv), PhEBX (**1g**, 0.192 g, 0.450 mmol, 1.50 equiv), 4CzIPN (**2a**, 0.012 g, 1.5 µmol, 5 mol%) in degassed CH_2Cl_2 (3 mL, 0.1 M).

Column chromatography (SiO_2, pentane) afforded (3,3-dimethylpent-1-yne-1,5-diyl)dibenzene (**4u**, 0.071 g, 0.22 mmol, 72%).

Rf (pentane) = 0.3.

^{1}H NMR (400 MHz, $CDCl_3$) δ 7.57 (dd, *J* = 8.0, 1.2 Hz, 1H, Ar*H*), 7.45 (dd, *J* = 7.7, 1.7 Hz, 1H, Ar*H*), 7.33 – 7.18 (m, 5H, Ar*H*), 7.22 – 7.14 (m, 1H, Ar*H*), 7.12 (td, *J* = 7.7, 1.7 Hz, 1H, Ar*H*), 2.96 – 2.87 (m, 2H, ArC*H*$_2$), 1.87 – 1.78 (m, 2H, C*H*$_2$), 1.38 (s, 6H, C(C*H*$_3$)$_2$).

^{13}C NMR (101 MHz, $CDCl_3$) δ 142.8, 133.2, 132.3, 128.7, 128.5, 128.4, 126.9, 126.0, 125.7, 101.9, 79.7, 45.5, 32.3, 32.2, 29.1. 1 carbon is not resolved.

IR (ν_{max}, cm^{-1}) 3062 (m), 3026 (m), 2968 (s), 2925 (m), 2865 (m), 2226 (m), 1466 (s), 1058 (m), 1047 (s), 1027 (s), 753 (s), 700 (s).

HRMS (APPI/LTQ-Orbitrap) m/z: $[M]^+$ Calcd for $C_{19}H_{19}{}^{79}Br^+$ 326.0665; Found 326.0676.

1-(3,3-Dimethyl-5-phenylpent-1-yn-1-yl)-4-chlorobenzene (4v)

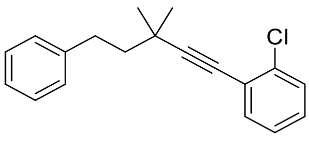

4v was synthesized following *general procedure J* using cesium 2-(methyl-4-phenylbutan-2-yl)oxy-2-oxoacetate (**3a**, 0.110 g, 0.300 mmol, 1.00 equiv), oClPhEBX (**1h**, 0.172 g, 0.450 mmol, 1.50 equiv), 4CzIPN (**2a**, 0.012 g, 1.5 µmol, 5 mol%) in degassed CH_2Cl_2 (3 mL, 0.1 M).

Column chromatography (SiO_2, pentane) afforded 1-(3,3-dimethyl-5-phenylpent-1-yn-1-yl)-4-chlorobenzene (**4v**, 0.066 g, 0.23 mmol, 78%).

Rf (pentane) = 0.3.

^{1}H NMR (400 MHz, $CDCl_3$) δ 7.48 – 7.35 (m, 2H, Ar*H*), 7.33 – 7.14 (m, 7H, Ar*H*), 2.95 – 2.86 (m, 2H, ArC*H*$_2$), 1.86 – 1.77 (m, 2H, C*H*$_2$), 1.38 (s, 6H, C(C*H*$_3$)$_2$).
^{13}C NMR (101 MHz, $CDCl_3$) δ 143.0, 136.0, 133.3, 129.3, 128.7, 128.6, 128.5, 126.4, 125.8, 123.9, 102.7, 78.0, 45.7, 32.4, 32.3, 29.3.
IR (ν_{max}, cm^{-1}) 2972 (m), 2901 (m), 1495 (w), 1406 (m), 1229 (m), 1075 (s), 905 (s), 729 (s).
HRMS (APPI/LTQ-Orbitrap) m/z: $[M]^+$ Calcd for $C_{19}H_{19}{}^{35}Cl^+$ 282.1170; Found 282.1178.

(3*R*,3a*S*,6*S*,7*R*,8a*S*)-3,6,8,8-tetramethyl-6-(phenylethynyl)octahydro-1H-3a,7-methanoazulene (4w)

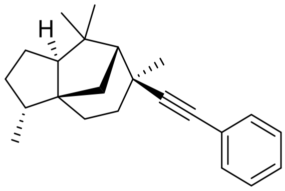

Direct excitation: **4w** was synthesized following *general procedure F* using cedrol derived cesium oxalate **3w** (0.128 g, 0.300 mmol, 1 equiv) and PhEBX (**1a**, 0.261 g, 0.750 mmol, 2.50 equiv) in degassed CH_2Cl_2 (3 mL, 0.1 M). Column chromatography (SiO_2, pentane) afforded (3*R*,3a*S*,6*S*,7*R*,8a*S*)-3,6,8,8-tetramethyl-6-(phenylethynyl)octahydro-1H-3a,7-methanoazulene (**4w**) as a single diasteroisomer (0.077 g (48% purity), dr > 20:1, 0.15 mmol, 50%).

Photocatalyzed: **4w** was synthesized following *general procedure J* using cedrol derived cesium oxalate **3w** (0.128 g, 0.300 mmol, 1 equiv), PhEBX (**1a**, 0.157 g, 0.450 mmol, 1.50 equiv), 4CzIPN (**2a**, 0.012 g, 1.5 µmol, 5 mol%) in degassed CH_2Cl_2 (3 mL, 0.1 M). Column chromatography (SiO_2, pentane) afforded (3*R*,3a*S*,6*S*,7*R*,8a*S*)-3,6,8,8-tetramethyl-6-(phenylethynyl)octahydro-1H-3a,7-methanoazulene (**4w**) as a single diasteroisomer (0.075 g (70% purity), dr > 20:1, 0.17 mmol, 58%). The compound could be partially purified from 1,4-diphenylbuta-1,4-diyne (major impurity) by preparative TLC (SiO_2, glass plate, Heptane) allowing full characterisation of **4w**.

Rf (pentane) = 0.6.
^{1}H NMR (400 MHz, $CDCl_3$) δ 7.42 – 7.35 (m, 2H, Ar*H*), 7.32 – 7.20 (m, 3H, Ar*H*), 2.18 – 2.10 (m, 1H, aliphatic-C*H* or C*H*$_2$), 1.95 – 1.83 (m, 2H, aliphatic-C*H* or C*H*$_2$), 1.85 – 1.76 (m, 1H, aliphatic-C*H* or C*H*$_2$), 1.79 – 1.65 (m, 5H, aliphatic-C*H* or C*H*$_2$), 1.60 – 1.50 (m, 1H, aliphatic-C*H* or C*H*$_2$), 1.48 (s, 3H, C*H*$_3$), 1.46 – 1.34 (m, 2H, aliphatic-C*H* or C*H*$_2$), 1.28 (dtd, *J* = 11.8, 7.7, 6.0 Hz, 1H, aliphatic-C*H* or C*H*$_2$), 1.22 (s, 3H, C*H*$_3$), 1.03 (s, 3H, C*H*$_3$), 0.87 (d, *J* = 7.1 Hz, 3H, C*H*$_3$).
^{13}C NMR (101 MHz, $CDCl_3$) δ 131.6, 128.3, 127.3, 124.6, 100.0, 80.8, 59.0, 57.4, 54.2, 44.4, 44.0, 42.0, 39.1, 37.1, 34.9, 31.9, 29.8, 29.0, 28.5, 25.6, 15.7.
IR (ν_{max}, cm^{-1}) 3055 (m), 3010 (m), 2950 (m), 2870 (m), 2851 (m), 1648 (m), 1474 (m), 1442 (m), 1246 (m), 755 (s), 724 (m), 690 (s).
HRMS (APPI/LTQ-Orbitrap) m/z: $[M]^+$ Calcd for $C_{23}H_{30}^+$ 306.2342; Found 306.2342.

HMBC

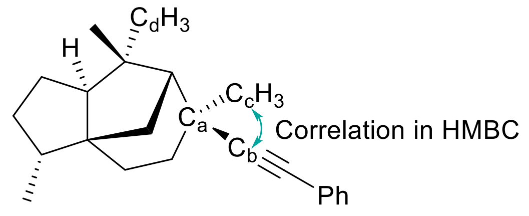

H
C_dH_3
C_a
C_cH_3
C_b
Correlation in HMBC
Ph

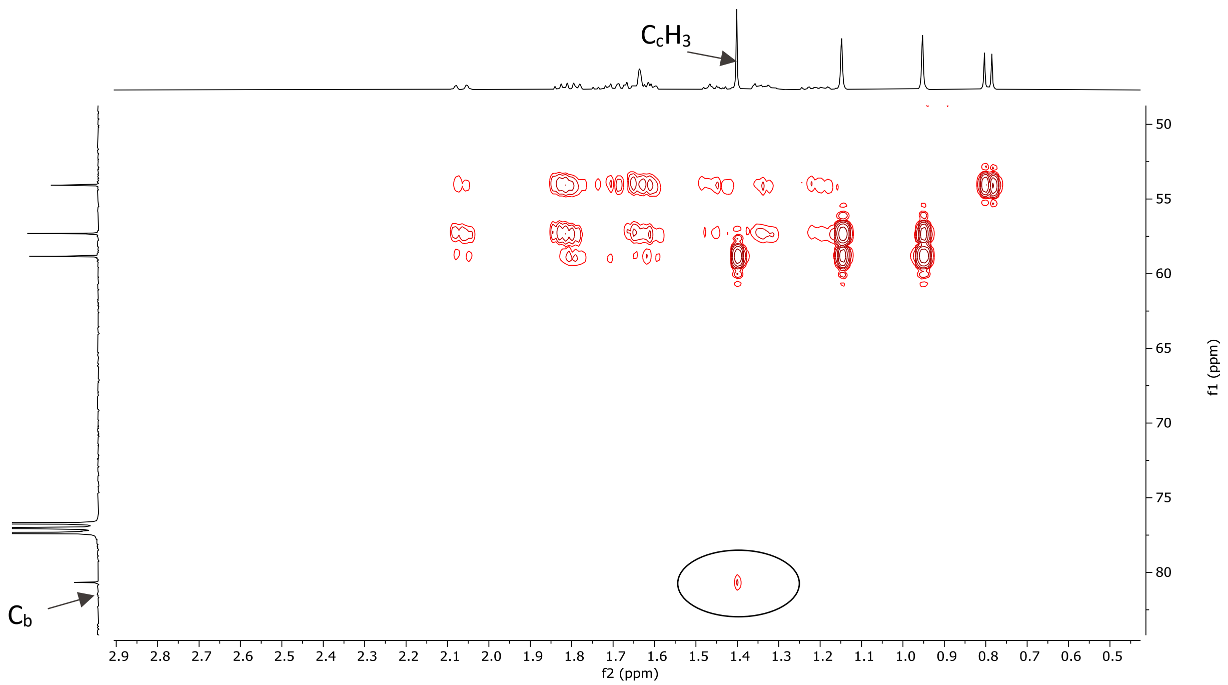

C_cH_3
C_b
50
55
60
65
70
75
80
f1 (ppm)
2.9 2.8 2.7 2.6 2.5 2.4 2.3 2.2 2.1 2.0 1.9 1.8 1.7 1.6 1.5 1.4 1.3 1.2 1.1 1.0 0.9 0.8 0.7 0.6 0.5
f2 (ppm)

NOESY

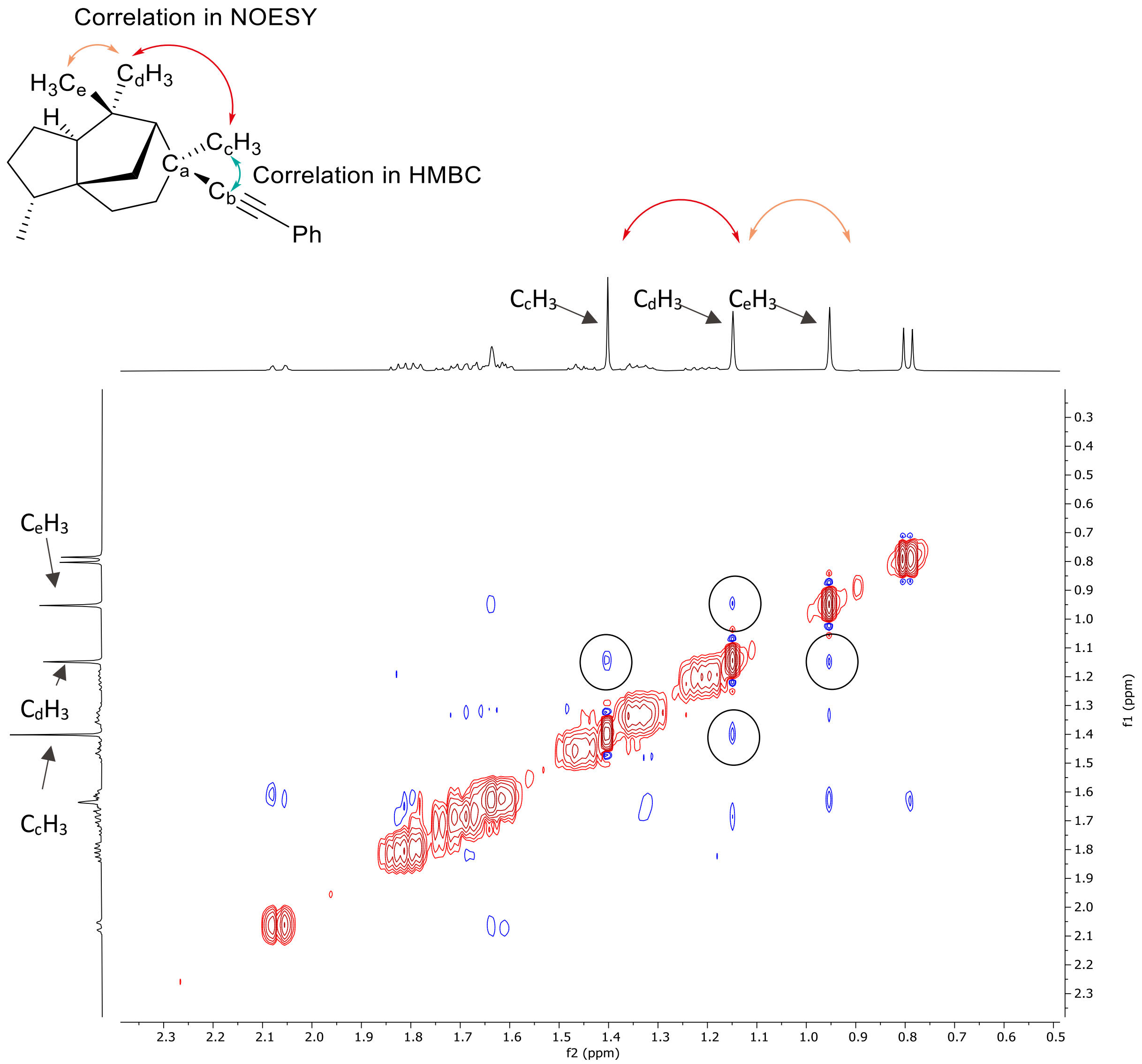
Correlation in NOESY
H3Ce
CdH3
H
CcH3
Ca
Cb
Correlation in HMBC
Ph
CcH3
CdH3
CeH3
CeH3
CdH3
CcH3
f2 (ppm)
f1 (ppm)

(1R,2R,5R)-5-isopropyl-2-methyl-2-(phenylethynyl)-6-oxabicyclo[3.2.1]octan-7-one (**4x**)

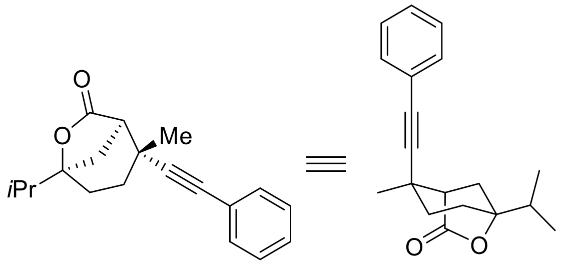

Direct excitation: **4x** was synthesized following the *general procedure F* using Cesium (*R*)-2-((1-isopropyl-4-methylcyclohex-3-en-1-yl)oxy)-2-oxoacetate (**3x**, 107 mg, 0.300 mmol, 1.00 equiv) and PhEBX (**1a**, 261 mg, 0.750 mmol, 2.5 equiv). Column chromatography (SiO_2, 5% EtOAc in Pentane) afforded (1*R*,2*R*,5*R*)-5-isopropyl-2-methyl-2-(phenylethynyl)-6-oxabicyclo[3.2.1]octan-7-one (**4x**, 40 mg, dr > 20:1, 0.16 mmol, 47%) as a colorless oil.

Photocatalyzed: **4x** was synthesized following the *general procedure J* using Cesium (*R*)-2-((1-isopropyl-4-methylcyclohex-3-en-1-yl)oxy)-2-oxoacetate (**3x**, 107 mg, 0.300 mmol, 1.00 equiv), PhEBX (**1a**, 157 mg, 0.450 mmol, 1.50 equiv) and 4CzIPN (**2a**, 12 mg, 0.015 mmol, 5 mol%). Column chromatography (SiO_2, 5% EtOAc in Pentane) afforded (1*R*,2*R*,5*R*)-5-isopropyl-2-methyl-2-(phenylethynyl)-6-oxabicyclo[3.2.1]octan-7-one (**4x**, 45 mg, dr > 20:1, 0.16 mmol, 53%) as a colorless oil.

Rf (pentane:EtOAc 95:5) = 0.4.
^{1}H NMR (400 MHz, $CDCl_3$) δ: 7.44 – 7.37 (m, 2H, Ar*H*), 7.35 – 7.28 (m, 3H, Ar*H*), 2.65 (dd, *J* = 5.6, 1.7 Hz, 1H, C*H*CO_2), 2.47 (d, *J* = 11.9 Hz, 1H, CO_2CHCH_{ax}), 2.15 – 2.08 (m, 1H, CO_2CHCH_{eq}), 2.07 – 2.00 (m, 1H, CCH_3CH_{ax}), 2.00 – 1.91 (m, 1H, $C(CH_3)_2H$), 1.91 – 1.85 (m, 1H, $COCH_{ax}$), 1.84 – 1.77 (m, 1H, $COCH_{eq}$), 1.74 – 1.63 (m, 1H, CCH_3CH_{eq}), 1.48 (s, 3H, CH_3), 1.01 (d, *J* = 6.8 Hz, 3H, $CH(CH_3)_2$), 0.97 (d, *J* = 6.9 Hz, 1H, $CH(CH_3)_2$).
^{13}C NMR (101 MHz, $CDCl_3$) δ: 176.3, 131.8, 128.5, 128.3, 123.2, 93.1, 90.1, 82.6, 51.0, 37.4, 35.3, 34.7, 34.3, 27.3, 26.8, 17.2, 16.8.
IR (ν_{max}, cm^{-1}): 3059 (w), 2967 (m), 2881 (m), 1773 (s), 1593 (w), 1461 (m), 1171 (m), 930 (m).
HRMS (APPI/LTQ-Orbitrap) m/z: $[M + Na]^+$ Calcd for $C_{19}H_{22}NaO_2^+$ 305.1512; Found 305.1512.

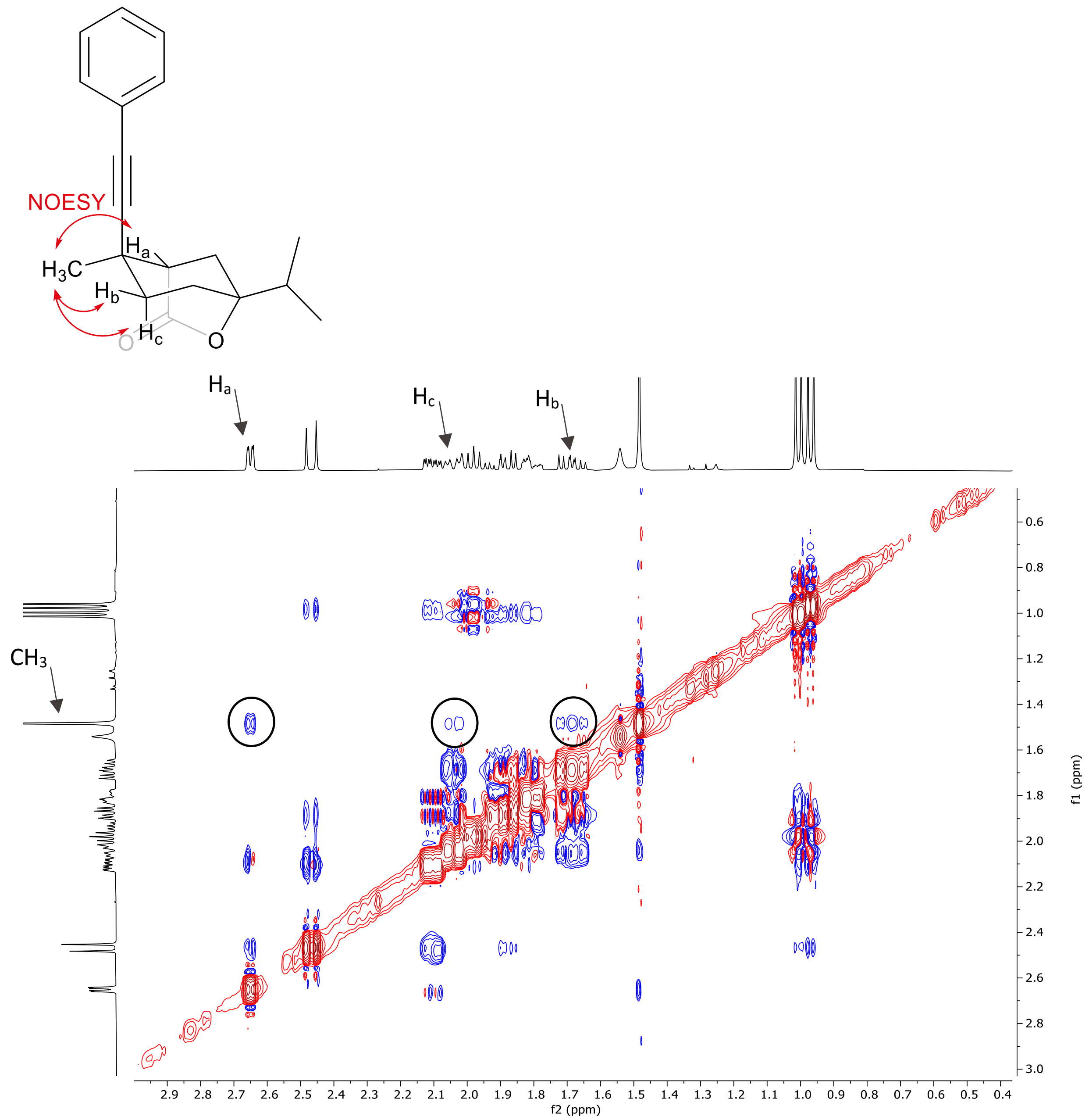
NOESY
H3C
Ha
Hb
Hc
O
O
Ha
Hc
Hb
CH3
f2 (ppm)
f1 (ppm)

5.2.2. Decarboxylation alkynylation

*1,3-Diphenylprop-2-yn-1-one (**8a**)*

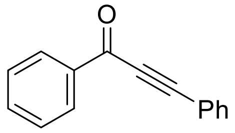

8a was synthesized following *general procedure G* using phenyglyoxylic acid (**7a**, 45 mg, 0.30 µmol, 1.0 equiv), PhEBX (**1a**, 261 mg, 750 µmol, 2.50 equiv), Cs_2CO_3 (195 mg, 600 µmol, 2.0 equiv) in degassed CH_2Cl_2 (6 mL, 0.05 M). Column chromatography (SiO_2, pentane:EtOAc 95:5) afforded 1,3-diphenylprop-2-yn-1-one (**8a**, 50 mg, 0.24 mmol, 81%) as a yellow solid.

Rf (pentane:EtOac 95:5) = 0.5.

^{1}H NMR (400 MHz, $CDCl_3$) δ 8.27 – 8.21 (m, 2H, Ar*H*), 7.72 – 7.68 (m, 2H, Ar*H*), 7.67 – 7.60 (m, 1H, Ar*H*), 7.56 – 7.47 (m, 3H, Ar*H*), 7.46 – 7.39 (m, 2H, Ar*H*).

^{13}C NMR (101 MHz, $CDCl_3$) δ 178.2, 137.0, 134.3, 133.2, 130.9, 129.7, 128.8, 128.8, 120.3, 93.3, 87.0.

Corresponds to reported literature data.[26]

*2-Cyclohexylethynylbenzene (**8b**)*

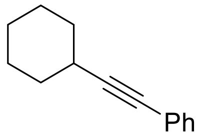

8b was synthesized following *general procedure G* using cyclohexanecarboxylic acid (**7b**, 38 mg, 0.30 µmol, 1.0 equiv), PhEBX (**1a**, 261 mg, 750 µmol, 2.50 equiv), Cs_2CO_3 (195 mg, 600 µmol, 2.0 equiv) in degassed CH_2Cl_2 (6 mL, 0.05 M). Column chromatography (SiO_2, pentane) afforded 1,3-diphenylprop-2-yn-1-one (**8b**, 35 mg, 0.15 mmol, 51%) as a yellow solid.

Rf (pentane) = 0.7.

^{1}H NMR (400 MHz, $CDCl_3$) δ 7.39 (ddd, *J* = 8.0, 3.3, 1.4 Hz, 2H, Ar*H*), 7.28 – 7.25 (m, 3H, Ar*H*), 2.59 (tt, *J* = 9.3, 3.7 Hz, 1H, C*H*-alkyne), 1.89 (ddd, *J* = 15.6, 7.0, 3.3 Hz, 2H, cyclic-C*H*₂), 1.76 (dtd, *J* = 12.2, 6.1, 2.3 Hz, 2H, cyclic-C*H*₂), 1.62 – 1.47 (m, 4H, cyclic-C*H*₂), 1.44 – 1.29 (m, 2H, cyclic-C*H*₂).

^{13}C NMR (101 MHz, $CDCl_3$) δ 131.7, 128.3, 127.5, 124.3, 94.6, 80.6, 32.9, 29.2, 26.1, 25.1.

Corresponds to reported literature data.[26]

*Tert-butyl 4-(phenylethynyl)piperidine-1-carboxylate (**8c**)*

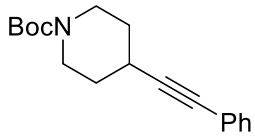

8c was synthesized following *general procedure G* using 1-Boc-piperidine-4-carboxylic acid (**7c**, 38 mg, 0.30 µmol, 1.0 equiv), PhEBX (**1a**, 261 mg, 750 µmol, 2.50 equiv), Cs_2CO_3 (195 mg, 600 µmol, 2.0 equiv) in degassed CH_2Cl_2 (6 mL, 0.05 M). Column chromatography (SiO_2, pentane) afforded 1,3-diphenylprop-2-yn-1-one (**8c**, 35 mg, 0.12 mmol, 41%) as a yellow solid.

Rf (pentane:EtOAc 9:1) = 0.4

^{1}H NMR δ 7.46 – 7.36 (m, 2H, Ar*H*), 7.28 (dt, *J* = 4.6, 2.9 Hz, 3H, Ar*H*), 3.74 (ddd, *J* = 13.5, 6.7, 3.7 Hz, 2H, N(C*H*₂)₂), 3.25 (ddd, *J* = 13.5, 8.4, 3.5 Hz, 2H, N(C*H*₂)₂), 2.80 (tt, *J* = 8.0, 4.0 Hz, 1H, C*H*-alkyne), 1.85 (ddt, *J* = 13.7, 6.9, 3.6 Hz, 2H, cyclic-C*H*₂), 1.67 (dtd, *J* = 15.1, 7.3, 3.3 Hz, 2H, cyclic-C*H*₂), 1.47 (s, 9H, *t*Bu).

Corresponds to reported literature data.[33]

5.2.3. Oxime fragmentation

Tert-*butyl (cyanomethyl)(3-phenylprop-2-yn-1-yl)carbamate (**10a**)*

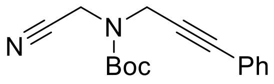

10a was synthesized following *general procedure H* using **9a** (77 mg, 0.30 mmol, 1.0 equiv), PhEBX (**1a**, 261 mg, 750 µmol, 2.50 equiv), K_2CO_3 (46 mg, 0.33 mmol, 1.1 equiv) in degassed CH_2Cl_2 (6 mL, 0.05 M). Column chromatography (SiO_2, pentane:EtOAc 20:1) afforded *tert*-butyl (cyanomethyl)(3-phenylprop-2-yn-1-yl)carbamate (**10a**, 60 mg, 0.22 mmol, 74%) as a yellow oil.

Rf (pentane:EtOac 20:1) = 0.35.

^{1}H NMR H NMR (400 MHz, Acetonitrile-*d_3*) δ 7.46 (m, 2H, Ph*H*), 7.37 (m, 3H, Ph*H*), 4.34 (s, 2H, C*H_2*), 4.27 (s, 2H, C*H_2*), 1.49 (s, 9H, Boc).

^{13}C NMR (101 MHz, CD_3CN) δ 154.9, 132.4, 129.7, 129.5, 128.7, 123.3, 117.6, 85.1, 84.5, 82.7, 36.1, 28.3.

Corresponds to reported literature data.[27]

*Tert-butyl (1-cyano-5-phenylpent-4-yn-2-yl)carbamate (**10b**)*

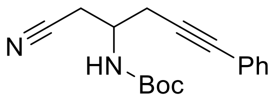

10b was synthesized following *general procedure G* using **9b** (86 mg, 0.30 mmol, 1.0 equiv), PhEBX (**1a**, 261 mg, 750 µmol, 2.50 equiv), K_2CO_3 (46 mg, 0.33 mmol, 1.1 equiv) in degassed CH_2Cl_2 (6 mL, 0.05 M). Column chromatography (SiO_2, pentane:EtOAc 9:1 to 8:2) afforded *tert*-butyl (1-cyano-5-phenylpent-4-yn-2-yl)carbamate (**10b**, 59 mg, 0.21 mmol, 69%) as a yellow solid.

Rf (pentane:EtOac 8:2) = 0.3.

^{1}H NMR (400 MHz, $CDCl_3$) δ 7.51 – 7.38 (m, 2H, Ar*H*), 7.39 – 7.28 (m, 3H, Ar*H*), 4.92 (m, 1H, N*H*), 4.14 (m, 1H, C*H*NHBoc), 2.90 – 2.69 (m, 4H, C*H_2*), 1.46 (s, 9H, *t*Bu).

^{13}C NMR (101 MHz, $CDCl_3$) δ 154.7, 131.6, 128.4, 128.3, 122.5, 116.9, 84.3, 83.3, 80.5, 46.3, 28.2, 24.5, 22.5.

Corresponds to reported literature data.[27]

---

[33] Liu, X.-G.; Zhou, C.-J.; Lin, E.; Han, X.-L.; Zhang, S.-S.; Li, Q.; Wang, H. *Angew. Chem. Int. Ed.* **2018**, *57*, 13096–13100.

*2-((3-Phenylprop-2-yn-1-yl)oxy)acetonitrile (**10c**)*

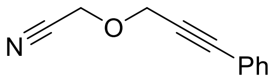

10c was synthesized following *general procedure G* using **9c** (50 mg, 0.30 mmol, 1.0 equiv), PhEBX (**1a**, 261 mg, 750 µmol, 2.50 equiv), K_2CO_3 (46 mg, 0.33 mmol, 1.1 equiv) in degassed CH_2Cl_2 (6 mL, 0.05 M). Column chromatography (SiO_2, pentane:EtOAc 95:5) afforded 2--((3-phenylprop-2-yn-1-yl)oxy)acetonitrile (**10c**, 27 mg, 0.16 mmol, 53%) as an off-white oil.

Rf (pentane:EtOac 95:5) = 0.3.

^{1}H NMR (400 MHz, $CDCl_3$) δ 7.51 – 7.39 (m, 2H, Ar*H*), 7.39 – 7.30 (m, 3H, Ar*H*), 4.55 (s, 2H, C*H*$_2$), 4.44 (s, 2H, C*H*$_2$).

^{13}C NMR (101 MHz, $CDCl_3$) δ 131.9, 129.1, 128.4, 121.8, 115.6, 88.7, 82.1, 59.0, 54.1.

Corresponds to reported literature data.[27]

5.2.4. Deboronative alkynylation

*2-(Phenylethynyl)-2,3-dihydro-1*H*-indene (**12a**)*

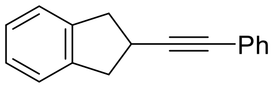

12a was synthesized following *general procedure I* using potassium 2,3-dihydro-1*H*-indenyl trifluoroborate (**11a**, 67 mg, 0.30 mmol, 1.0 equiv), PhEBX (**1a**, 261 mg, 0.750 mmol, 2.50 equiv), Na_2CO_3 (64 mg, 0.60 mmol, 2.0 equiv) in degassed CH_2Cl_2:water (1:1) (3 mL, 0.1 M). Column chromatography (SiO_2, 0 to 2% EtOAc in Pentane) afforded 2-(phenylethynyl)-2,3-dihydro-1*H*-indene (**12a,** 47 mg, 0.22 mmol, 72%) as a pale yellow solid.

Rf (pentane) = 0.4.

^{1}H NMR (400 MHz, $CDCl_3$) δ 7.43 – 7.40 (m, 2H Ar*H*), 7.30 – 7.27 (m, 3H, Ar*H*), 7.25 – 7.21 (m, 2H, Ar*H*), 7.19 – 7.16 (m, 2H, Ar*H*), 3.49 – 3.40 (m, 1H, C*H*), 3.35 – 3.29 (m, 2H, C*H*$_2$), 3.13 (dd, *J* = 15.2, 8.7 Hz, 2H, C*H*$_2$).

^{13}C NMR (101 MHz, $CDCl_3$) δ 142.2 131.8, 128.3, 127.8, 126.7, 124.5, 123.9, 93.2, 80.7, 40.5, 30.9.

Corresponds to reported literature data. [23]

*But-1-yne-1,4-diyldibenzene (**12b**)*

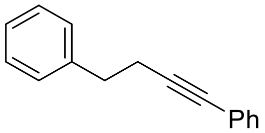

12b was synthesized following *general procedure I* using potassium 2-phenylethyl trifluoroborate (**11a**, 64 mg, 0.30 mmol, 1.0 equiv), PhEBX (**1a**, 261 mg, 0.750 mmol, 2.50 equiv), Na_2CO_3 (64 mg, 0.60 mmol, 2.0 equiv) in degassed CH_2Cl_2:water (1:1) (3 mL, 0.1 M). Column chromatography (SiO_2, 0 to 2% EtOAc in Pentane) afforded but-1-yne-1,4-diyldibenzene (**12b**, 38 mg, 0.18 mmol, 61%) as a pale yellow oil.

Rf (pentane) = 0.4.

^{1}H NMR (400 MHz, $CDCl_3$) δ: 7.39 – 7.36 (m, 2H, Ar*H*), 7.33 – 7.30 (m, 2H, Ar*H*), 7.31 – 7.27 (m, 5H, Ar*H*), 7.25 – 7.21 (m, 1H, Ar*H*), 2.93 (t, *J* = 7.5 Hz, 2H, CH_2), 2.70 (t, *J* = 7.5 Hz, 2H, CH_2).

^{13}C NMR (101 MHz, $CDCl_3$) δ 140.9, 131.7, 128.7, 128.5, 128.3, 127.8, 126.5, 124.0, 89.6, 81.4, 35.3, 21.8.

Corresponds to reported literature data. [23]

*2-Cyclohexylethynylbenzene (**12c** same structure as **8b**)*

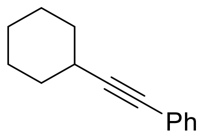

12c was synthesized following *general procedure I* using potassium cyclohexyl trifluoroborate (**11c**, 57 mg, 0.30 mmol, 1.0 equiv), PhEBX (**1a**, 261 mg, 750 µmol, 2.50 equiv), Na_2CO_3 (64 mg, 0.60 mmol, 2.0 equiv) in degassed CH_2Cl_2 (6 mL, 0.05 M). Column chromatography (SiO_2, pentane) afforded 2-cyclohexylethynylbenzene (**12c**, 26 mg, 0.14 mmol, 47%) as a yellow solid.

Rf (pentane) = 0.7.

^{1}H NMR (400 MHz, $CDCl_3$) δ 7.39 (ddd, *J* = 8.0, 3.3, 1.4 Hz, 2H, Ar*H*), 7.28 – 7.25 (m, 3H, Ar*H*), 2.59 (tt, *J* = 9.3, 3.7 Hz, 1H, C*H*-alkyne), 1.89 (ddd, *J* = 15.6, 7.0, 3.3 Hz, 2H, cyclic-CH_2), 1.76 (dtd, *J* = 12.2, 6.1, 2.3 Hz, 2H, cyclic-CH_2), 1.62 – 1.47 (m, 4H, cyclic-CH_2), 1.38 – 1.25 (m, 2H, cyclic-CH_2).

^{13}C NMR (101 MHz, $CDCl_3$) δ 131.7, 128.3, 127.5, 124.3, 94.6, 80.6, 32.9, 29.2, 26.1, 25.1.

Corresponds to reported literature data.[23]

6. Mechanistic studies

6.1. Monitoring of the reaction by ^{1}H NMR

Table S3. Normalized ^{1}H NMR yields taken over 32 hours

Time (hours)	3a	1a	4a	5a	5b	1a	7	total oxalate	total alkyne
**0**	1	2.5	0	0	0	1	0	1	2.5
**0.5**	0.8	1.9	0.11	0.06	0.07	0.76	0.18	0.98	2.5
**1**	0.51	1.4	0.29	0.21	0.17	0.56	0.36	0.97	2.79
**4**	0.1	0.58	0.63	0.33	0.17	0.23	0.43	0.9	2.57
**8**	0.04	0.34	0.66	0.35	0.18	0.14	0.43	0.88	2.39
**16**	0.04	0.26	0.58	0.35	0.24	0.1	0.48	0.86	2.39
**24**	0.04	0.14	0.63	0.44	0.24	0.06	0.5	0.91	2.45
**32**	0.03	0.04	0.62	0.39	0.27	0.02	0.5	**0.92**	**2.32**

We attempted to study the evolution of iodobenzoate formation however, due to shift variations in the NMR that our concentration dependent and its insolubility in chloroform the results were non-conclusive. We attempted a study in DMSO-d_6 but the signal of trace DCM (reaction solvent) overlapped with the signals of **5a** and **5b**.

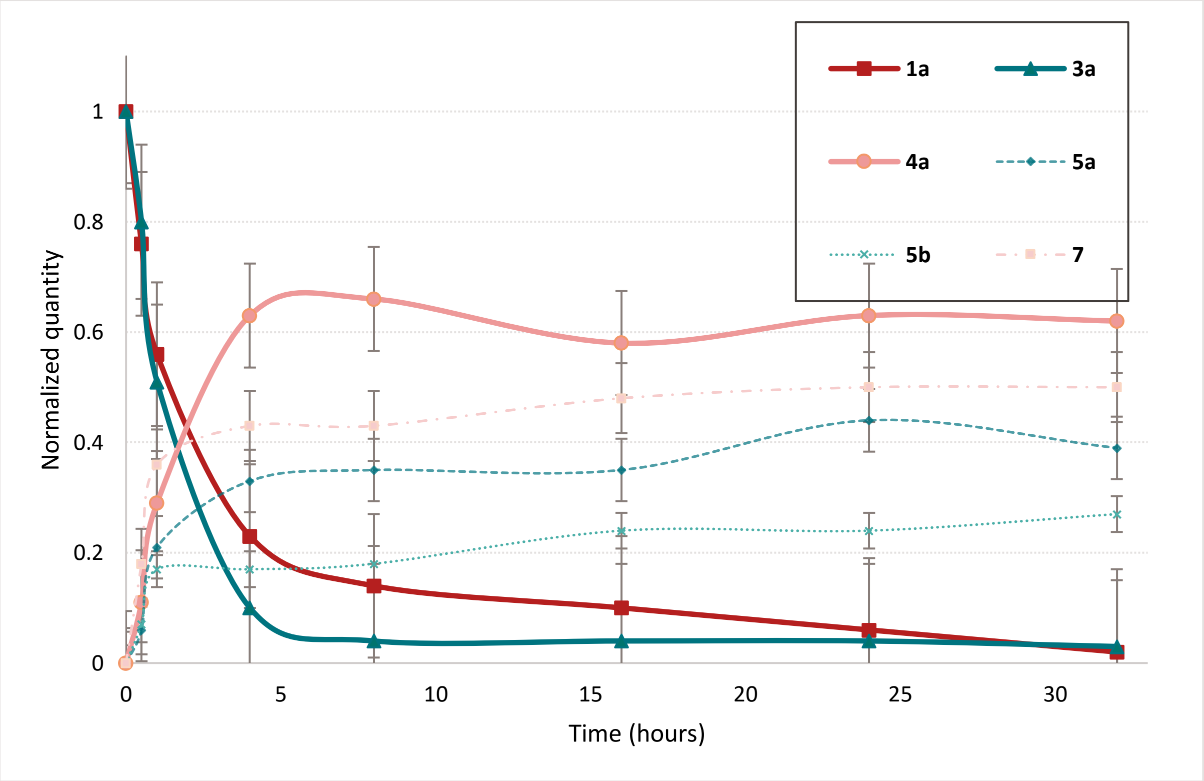

Figure S2. Evolution of **1a**, **3a**, **4a**, **5a**, **5b** and **7** over 32 hours

6.2. Side product formation and reaction with TIPS-EBX (1c) monitored by ^{1}H NMR

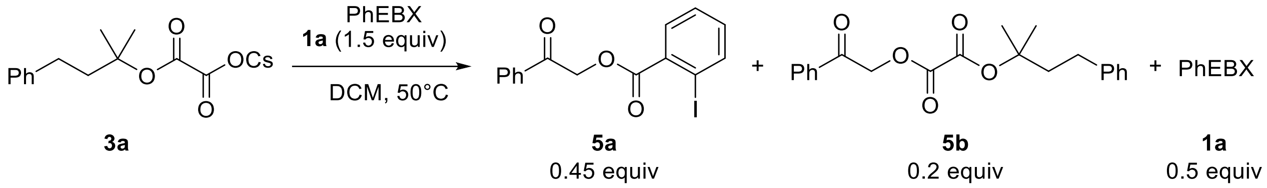

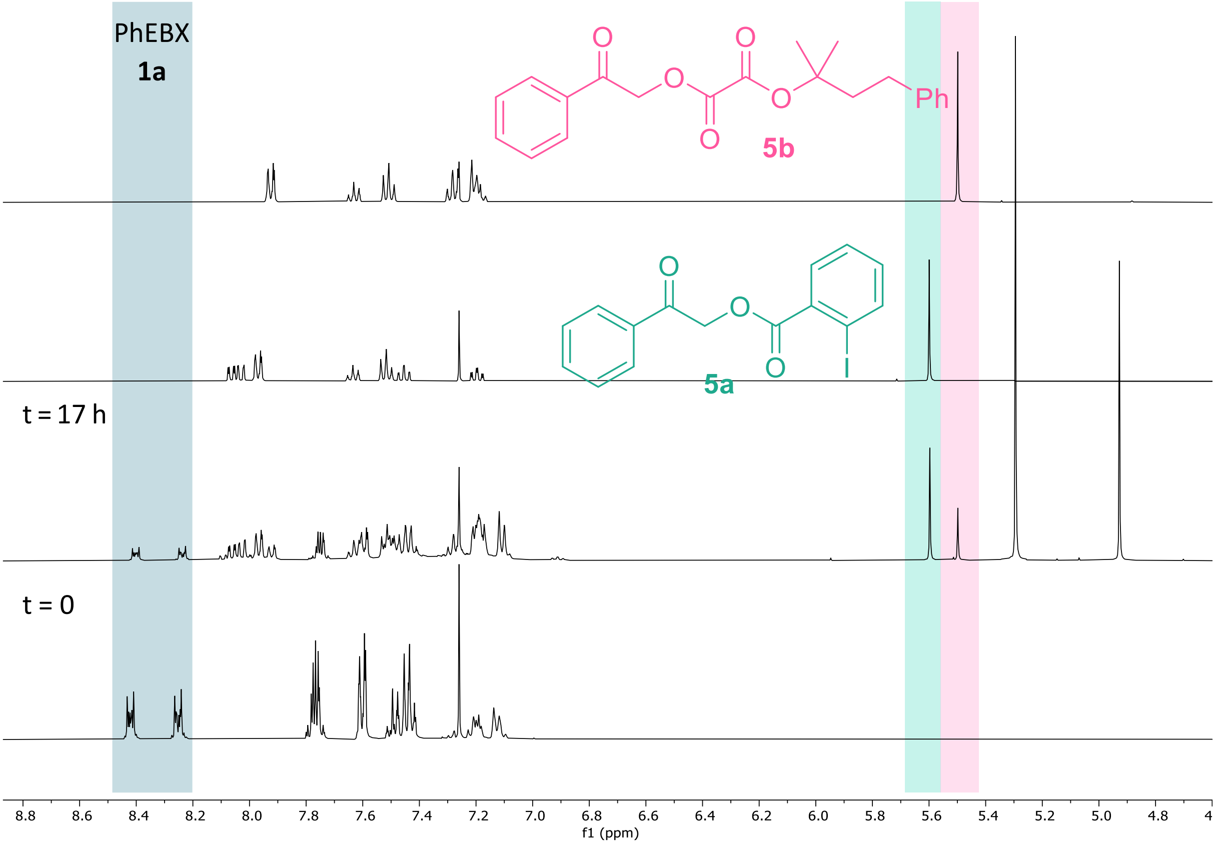

Figure S3. Thermal degradation of PhEBX in presence of **3a**

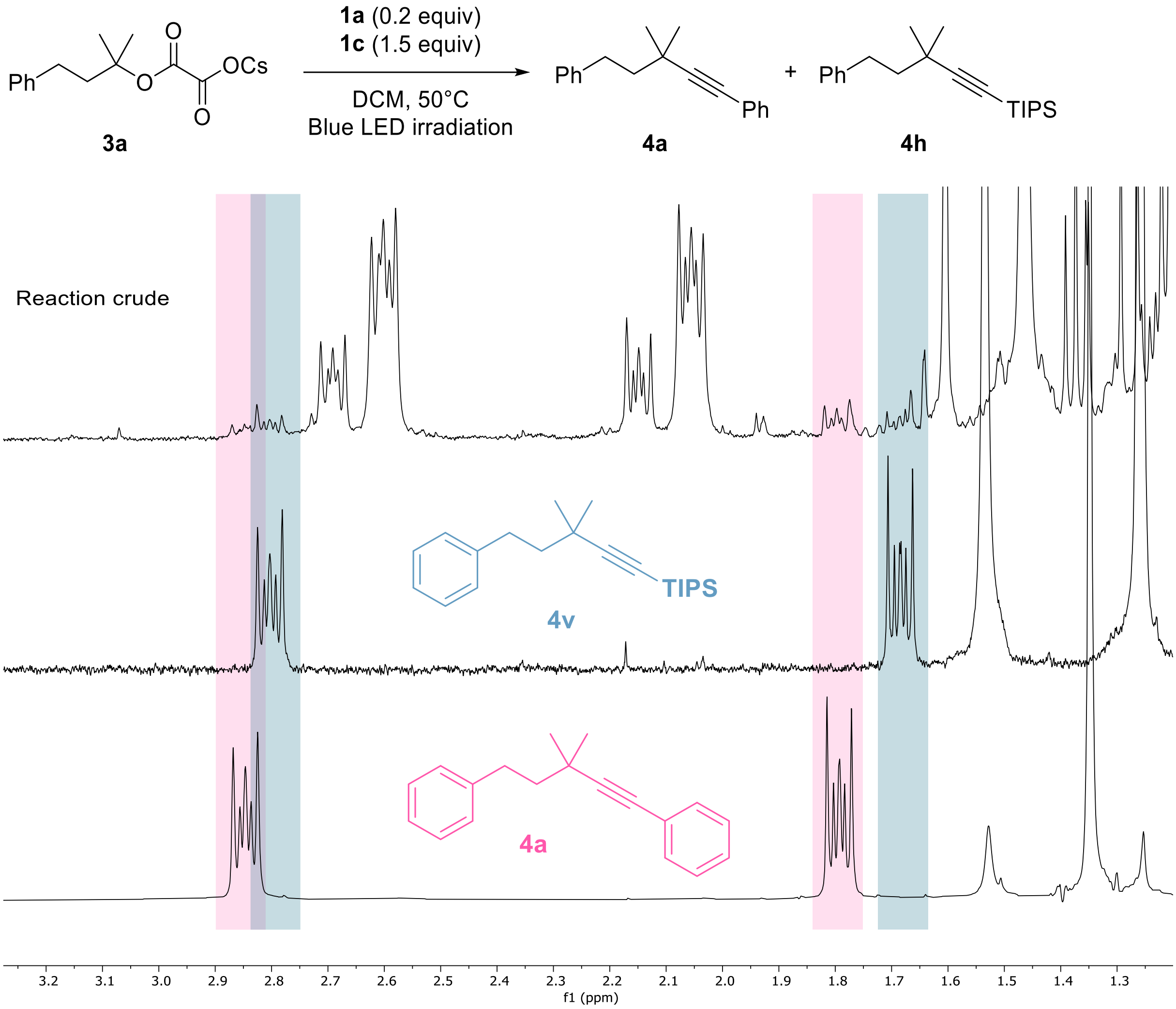

Figure S4. TIPS-alkynylation with PhEBX as a photooxidant

6.3. Synthesis and characterization of **5a, 5b** and **4h**

2-Oxo-2-phenylethyl 2-iodobenzoate (**5a**)

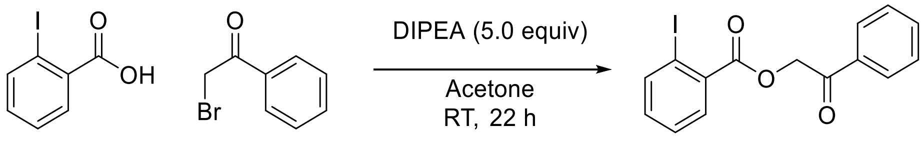

Following a reported procedure,[34] 2-iodobenzoic acid (**20**, 744 mg, 3.00 mmol, 1.00 equiv) and 2-bromo-1-phenylethanone (**39**, 657 mg, 3.30 mmol, 1.10 equiv) were dissolved in acetone (12.0 mL). DIPEA (2.6 mL, 15 mmol, 5.0 equiv) was then added and the reaction mixture was stirred overnight. The mixture was then diluted with EtOAc and washed with water. The organic layer was dried over

[34] Speckmeier, E.; Zeitler, K. *ACS Catal.* **2017**, *7*, 6821–6826.

$MgSO_4$, filtered and the solvent was removed under reduced pressure. The crude product was purified by flash chromatography (SiO_2, 10% EtOAc in pentane, Rf = 0.4) obtaining 2-oxo-2-phenylethyl 2-iodobenzoate (**5a**, 660 mg, 1.80 mmol, 60% yield) as an off-white solid.

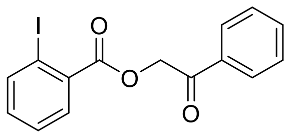

^{1}H NMR (400 MHz, $CDCl_3$) δ: 8.06 (dd, *J* = 7.8, 1.7 Hz, 1H, Ar*H*), 8.03 (dd, *J* = 8.0, 1.2 Hz, 1H, Ar*H*), 8.00 – 7.94 (m, 2H, Ar*H*), 7.68 – 7.59 (m, 1H, Ar*H*), 7.56 – 7.49 (m, 2H, Ar*H*), 7.45 (td, *J* = 7.7, 1.2 Hz, 1H, Ar*H*), 7.20 (td, *J* = 7.7, 1.7 Hz, 1H, Ar*H*), 5.60 (s, 2H, C*H*$_2$).
^{13}C NMR (101 MHz, CDCl3) δ: 191.8, 165.9, 141.6, 134.3, 134.2, 133.2, 131.8, 129.1, 128.2, 128.0, 94.6, 66.9. 1 Carbon atom is unresolved. Constitent with reported literature data.[34]

2-methyl-4-phenylbutan-2-yl (2-oxo-2-phenylethyl) oxalate (5b)

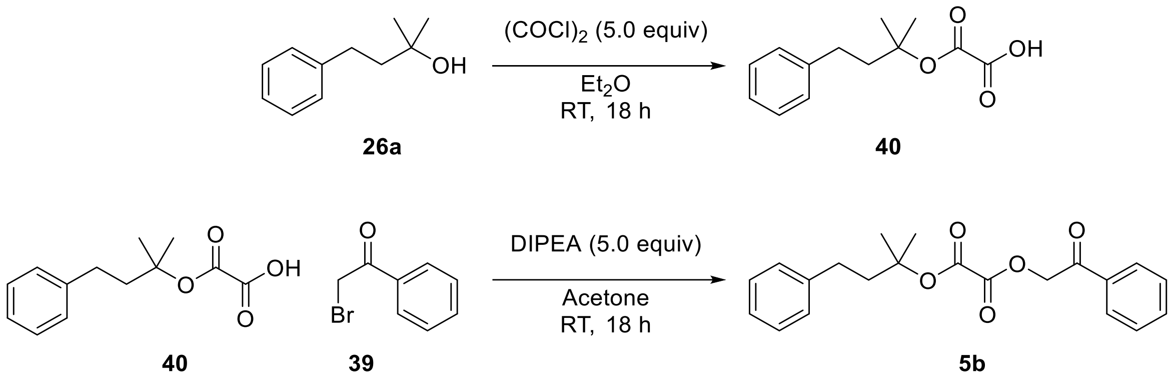

Following a modified reported procedure,[35] a solution of 2-methyl-4-phenylbutan-2-ol (**26a**, 0.85 mL, 5.0 mmol, 1.0 equiv) in Et_2O (40 mL) was cooled to 0 °C. Oxalyl dichloride (0.90 mL, 10 mmol, 2.0 equiv) was then added dropwise. The mixture was warmed to room temperature after 10 min, and after an additional 1.5 h, oxalyl dichloride (0.44 mL, 5.0 mmol, 1.0 equiv) were added. After an additional 1h oxalyl dichloride (0.90 mL, 10 mmol, 2.0 equiv) was added and the reaction was stirred for another hour. The reaction was carefully quenched at 0 °C by the dropwise addition of H2O (30 mL) after addition of a vent needle. The mixture was stirred vigorously and warmed to room temperature. The layers were separated, and the aqueous layer extracted with Et_2O (3 x 15 mL), and the combined organic layers dried with Na_2SO_4, filtered, and concentrated under reduced pressure affording 2-(2-methyl-4-phenylbutan-2-yl)oxy-2-oxoacetic acid as a clear oil (767 mg, 3.25 mmol, 65% yield), which was used directly in the next step. Following a modified reported procedure,[34] the crude oil of 2-(2-methyl-4-phenylbutan-2-yl)oxy-2-oxoacetic acid (**40**, 767 mg, 3.25 mmol, 1.0 equiv) was dissolved in acetone (12 mL). DIPEA (2.4 mL, 15 mmol, 5 equiv) and phenacyl bromide (**39**, 597 mg, 3.00 mmol, 0.9 equiv) were then added. The reaction was stirred overnight. The reaction was quenched with water (5 mL), diluted with EtOAc (20 mL). The organic layer was washed with sat. aq. NH_4Cl (3 x 10 mL), then brine (10 mL). The organic layer was dried over Na_2SO_4, filtered, and concentrated *in vacuo* to afford 2-methyl-4-phenylbutan-2-yl (2-oxo-2-phenylethyl) oxalate as a crude yellow oil (**5b**, 930 mg, 2.62 mmol, 87% yield, 52% yield over both steps).

[35] Su, J. Y.; Grünenfelder, D. C.; Takeuchi, K.; Reisman, S. E. *Org. Lett.* **2018**, *20*, 4912–4916.

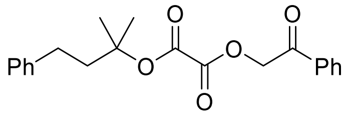

^{1}H NMR (400 MHz, $CDCl_3$) δ 7.96 – 7.89 (m, 2H, Ar*H*), 7.67 – 7.58 (m, 1H, Ar*H*), 7.50 (t, *J* = 7.7 Hz, 2H, Ar*H*), 7.29 (t, *J* = 7.5 Hz, 2H, Ar*H*), 7.25 – 7.17 (m, 3H, Ar*H*), 5.50 (s, 2H, C(O)C*H*$_2$O), 2.77 – 2.69 (m, 2H, PhC*H*$_2$), 2.23 – 2.14 (m, 2H, C*H*$_2$), 1.65 (s, 6H, C(C*H*$_3$)$_2$.
^{13}C NMR (101 MHz, $CDCl_3$) δ 190.1, 157.7, 156.2, 141.6, 134.2, 133.9, 129.0, 128.5, 128.5, 127.9, 126.0, 87.2, 67.6, 42.6, 30.3, 25.8.
IR (ν_{max}, cm^{-1}) 2978 (s), 2904 (s), 1739 (m), 1705 (m), 1381 (m), 1242 (m), 1165 (s), 1111 (s), 1065 (s).
HRMS (ESI/QTOF) m/z: $[M + Na]^+$ Calcd for $C_{21}H_{22}NaO_5^+$ 377.1359; Found 377.1363.

(3,3-Dimethyl-5-phenylpent-1-yn-1-yl)triisopropylsilane (**4h**)

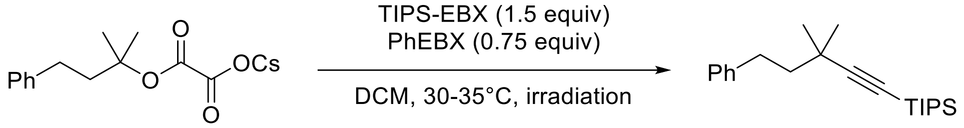

An oven dried dram vial (2 mL), equipped with a magnetic stirrer, was charged with cesium oxalate (**3a**, 0.036 g, 0.10 mmol, 1 equiv), TIPS-EBX (**1c**, 0.064 g, 0.15 mmol, 1.5 equiv) and PhEBX (**1a**, 0.026 g, 0.075 mmol, 0.75 equiv). The reaction vial was sealed with a septum. After 3 vacuum/N2 cycles (backfilling with Ar on the last cycle), dry degassed (freeze pump thaw) CH_2Cl_2 was added and the septum was replaced with a screw cap under a flux of Ar. The reactions were placed between 2 x 440 nm Kessil lamps at ca. 10 cm distance from both lamps (with ventilation T = ca. 30-35°C as specified) and stirred under irradiation for 18 hours. The reaction was filtered through a small celite plug which was washed with CH_2Cl_2. The reaction crude was concentrated *in vacuo*, and purified by preparative TLC (SiO2, heptane), affording (3,3-dimethyl-5-phenylpent-1-yn-1-yl)triisopropylsilane (**4h**, 2 mg, 0.006 mmol, 6% yield)

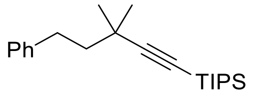

Rf (pentane) = 0.55
^{1}H NMR (400 MHz, $CDCl_3$) δ 7.29 (d, *J* = 7.6 Hz, 2H, Ar*H*), 7.23 – 7.14 (m, 3H, Ar*H*), 2.85 – 2.75 (m, 2H, PhC*H*$_2$), 1.72 – 1.65 (m, 2H, C*H*$_2$), 1.26 (s, 6H, C(C*H*$_3$)$_2$), 1.13 – 0.98 (m, 22H, TIPS).
^{13}C NMR (101 MHz, CDCl3) δ 143.0, 128.4, 128.4, 125.6, 116.2, 79.4, 45.8, 32.2, 29.7, 29.4, 18.7, 11.3.
HRMS (APPI/LTQ-Orbitrap) m/z: $[M + Na]^+$ Calcd for $C_{22}H_{36}NaSi^+$ 351.2478; Found 351.2485.

6.4. Control experiments

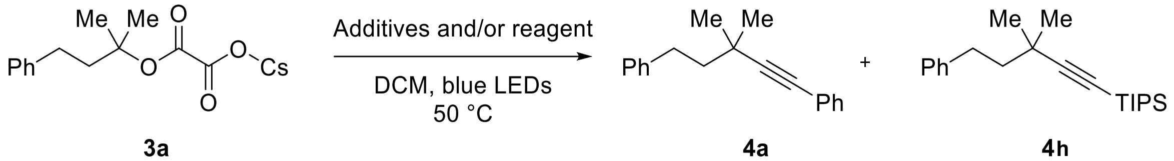

An oven dried dram vial (2 mL), equipped with a magnetic stirrer, was charged with the solid components following table S3: cesium oxalate **3a**, TIPSEBX (**1c**) , PhEBX (**1a**), 4CzIPN (**2a**), **5a**, **5b**, BIOAc (**19a**), BIOH (**19b**). The reaction vial was sealed with a septum. After 3 vacuum/N2 cycles (backfilling with Ar on the last cycle), CH_2Cl_2 (3.0 mL) was added and the septum was replaced with a

screw cap under a flux of Ar.[36] The reactions were placed between 2 x 440 nm Kessil lamps (unless specified otherwise) at ca. 10 cm distance from both lamps (no ventilation, T = ca. 50 °C) and stirred under irradiation for 18 hours. The reaction was filtered through a small celite plug which was washed with CH_2Cl_2. The reaction crude was concentrated *in vacuo*, diluted with $CDCl_3$ and 1 equiv of CH_2Br_2 was added as internal standard for ^1H NMR analysis.

Table S4. Control reactions for the indetification of the photoactive species without photocatalyst

entry	Reagent (1.5 equiv)	additive (equiv)	residual 3a (%)	^{1}H NMR yield (%)
**1**	**1c**	-	100	nd
**2**	**1c**	**1a** (0.05)	30	25
**3**	**1c**	**5a** (0.2)	98	2
**4**	**-**	**5a** (1.0)	100	-
**5**[a]	**1a**	**5b** (1.0)	-	nd[b]
**6**	**1c**	**5b** (0.7)	>90	<5
**7**	**1c**	**19a** (0.2)	92	5
**8**	**1c**	**19b** (0.2)	92	5
**9**	**-**	**19a** (1.5)	100	-
**10**	**-**	**19b** (1.5)	90	-
**11**	**1c** (1.0 equiv)	**19a** (2.5)	>95	-
**12**	**-**	**5b** (1.0)	>95	-
**13**	**1c**	**1a** (0.2)	80	16

[a]No cesium salt was used. [b]No degradation of **5b** was observed, full decomposition of PhEBX. This suggests that **5b** is not a reaction intermediate.

6.5. UV-Vis absorption and fluorescence studies

Absorption and fluorescence studies of PhEBX 1a and the cesium oxalate 3a

A 5 mL 0.2 M stock solution of PhEBX (348 mg, 1.00 mmol) and a 2 mL 0.2 M stock solution of **3a** (147 mg, 0.4 mmol) were prepared in DMSO (from fresh ampoules, degassed and deuterated) were prepared in a 5 mL and 2 mL volumetric flask. The samples were prepared by dissolving 0.50 mL of stock solution with 0.5 mL of fresh DMSO, final concentration: 0.1 M. The samples were then submitted to UV-Vis, fluorescence and fluorescence excitation spectroscopy.

[36] Use of a screw cap or crimp cap is of great importance, the irradiation causes an increase in temperature causing the CH_2Cl_2 to evaporate and an overpressure inside the vessel. When using a septum, the latter would fly off within an hour of irradiation. As shown in the optimization section DCE is not as good a solvent as CH_2Cl_2.

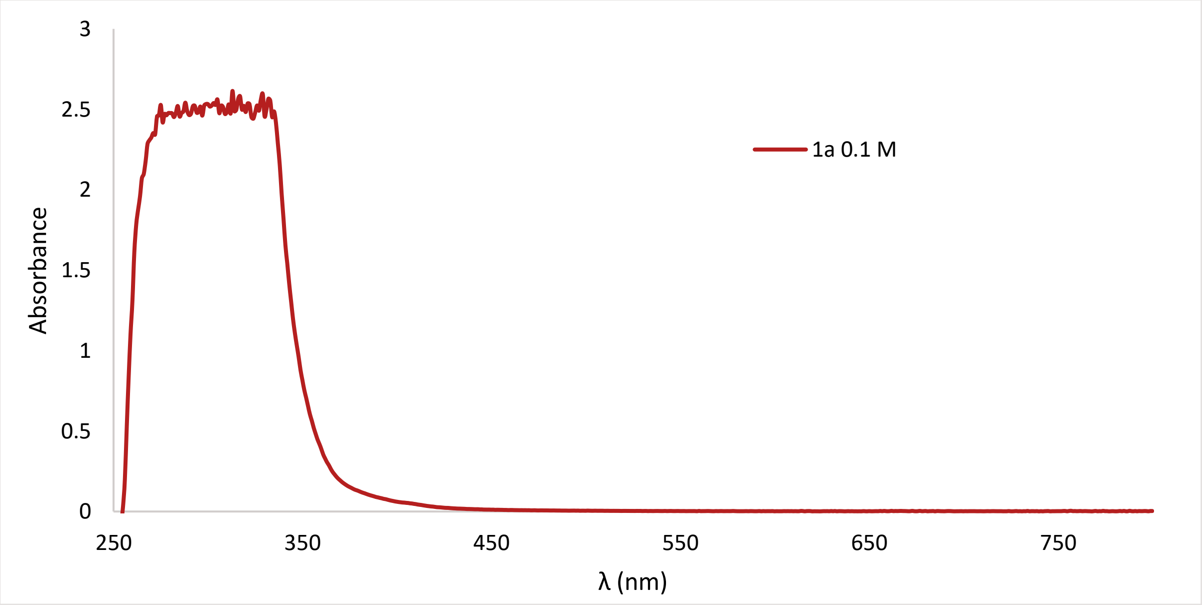

Figure S5. Absorption of PhEBX **2a**, 0.1 M in DMSO

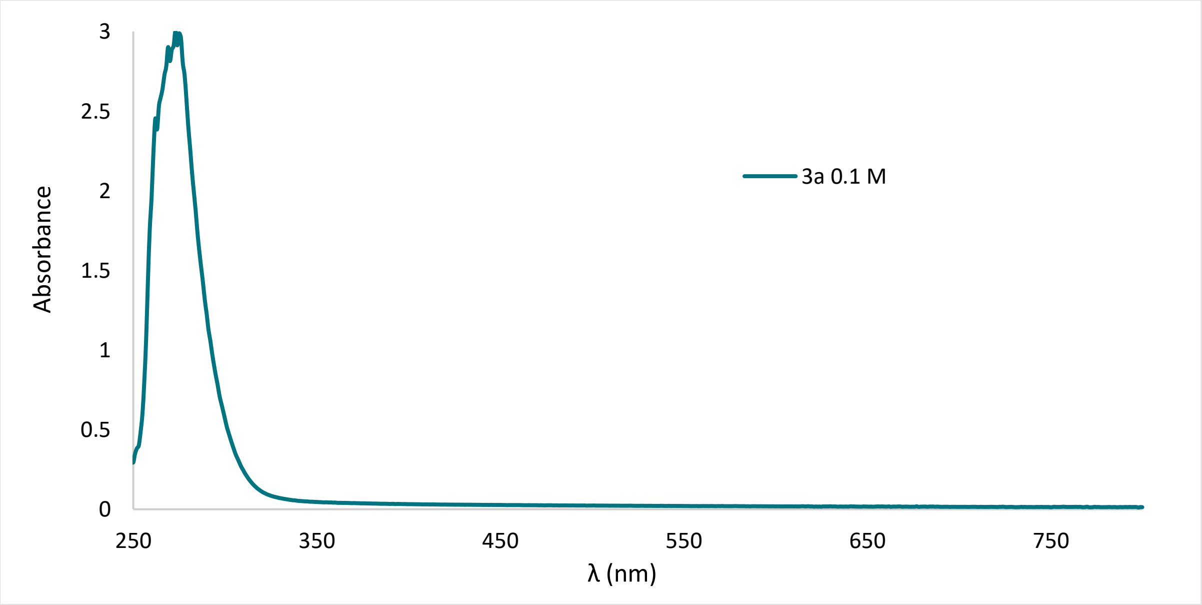

Figure S6. Absorption of **3a** 0.1 M in DMSO

We checked for the presence of an EDA complex by combining 0.50 mL of both stock solutions of **2a** and **3a** and measuring the UV-Vis spectrum, no new band can be observed (Figure S7)

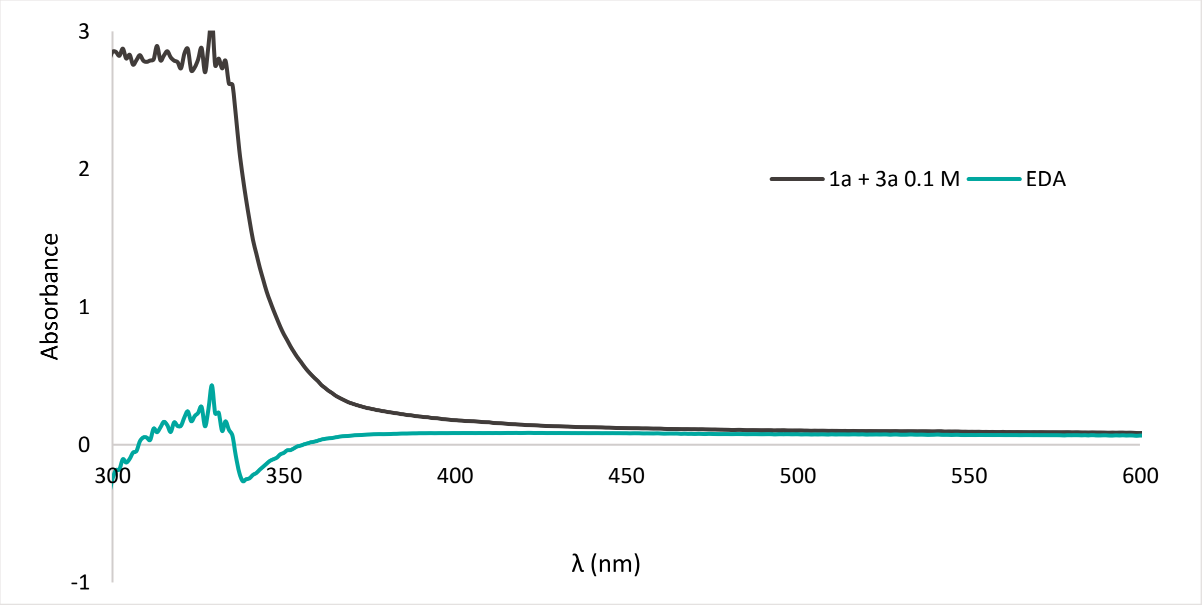

Figure S7. Absorption of a 1:1 mixture **2a**:**3a**.

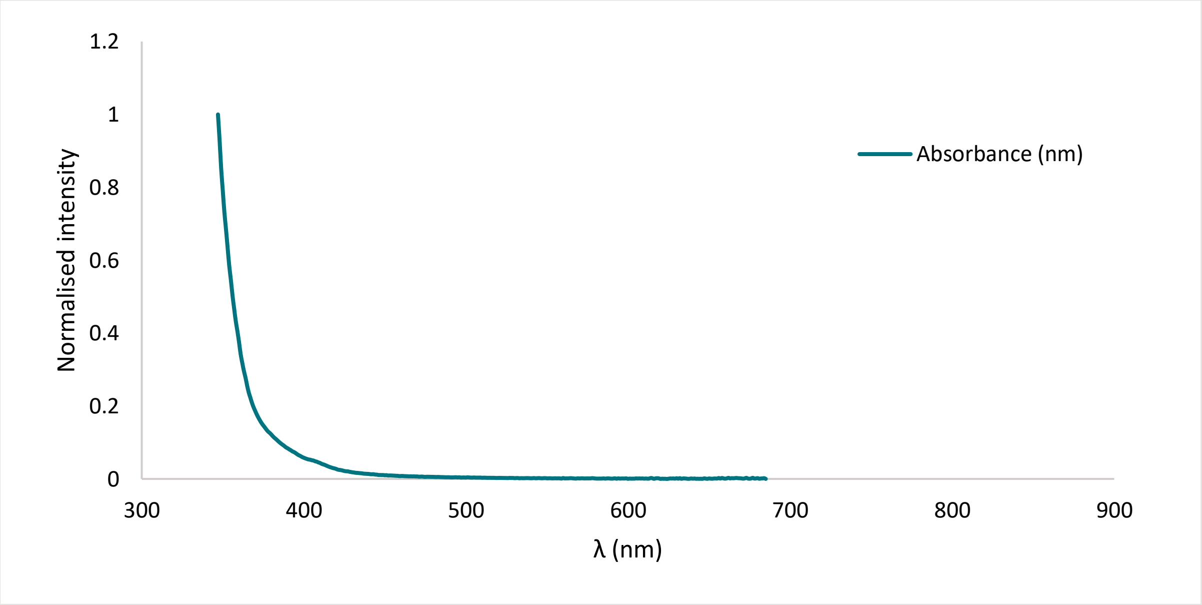

Figure S8. Normalized absorption, fluorescence (390 nm) and fluorescence excitation (485 nm) of **1a** (0.1 M) in DMSO

Absorption and Beer-Lambert linear regression at 420 nm and 440 nm of PhEBX (1a)

A 5 mL 0.14 M stock solution of PhEBX (**1a**, 243 mg, 0.700 mmol) in DMSO (from fresh ampoules, degassed and deuterated) was prepared in a 5 mL volumetric flask. Then 1 mL solutions were prepared following table S4, where C(**1a**) is the concentration of the stock solution, V(**1a**) is the volume of the stock solution used for the sample, V(DMSO) the volume of DMSO added for the dilution C_f(**1a**) the final concentration of the sample. UV-Vis spectra of each sample were then measured. Reproducibility of the measure was verified by repetition of the analyses.

Table S5. Sample preparation table for UV-Vis analyses for the Beer-Lambert linear regression

C(**1a**) (M)	V(**1a**) (mL)	V(DMSO) (mL)	C_f(**1a**) (M)
0.14	0	1.00	0
0.14	0.10	0.90	0.014
0.14	0.30	0.70	0.042
0.14	0.50	0.50	0.07
0.14	0.70	0.30	0.098
0.14	1.00	0	0.14

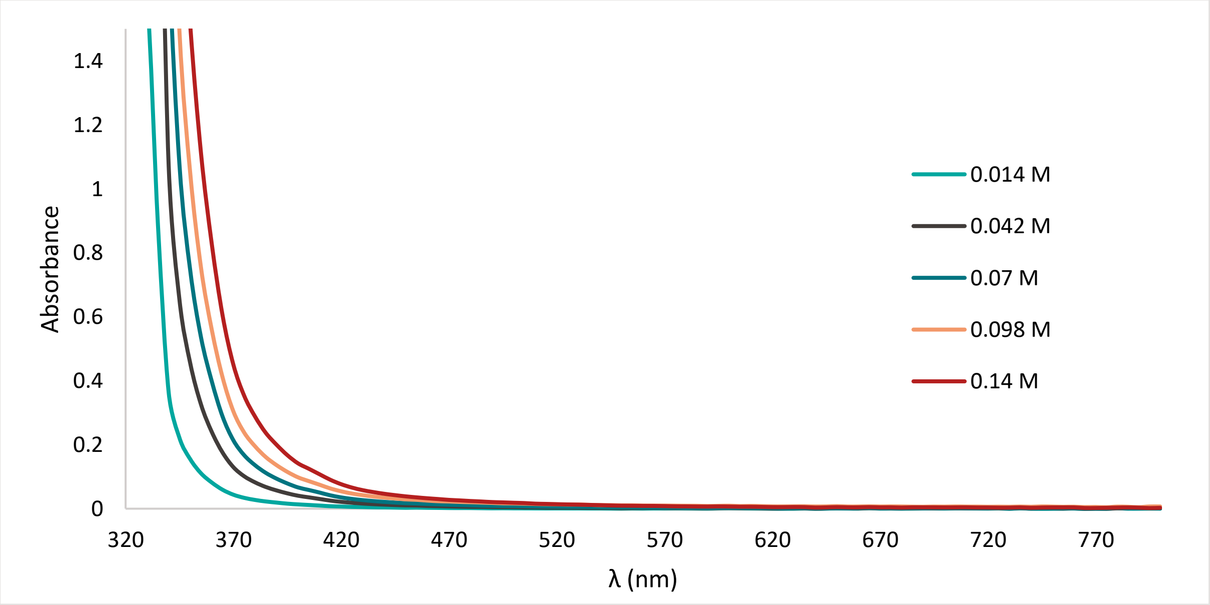

Figure S9. Absorption spectra of **1a** at concentrations from 0.014 M to 0.14 M in DMSO

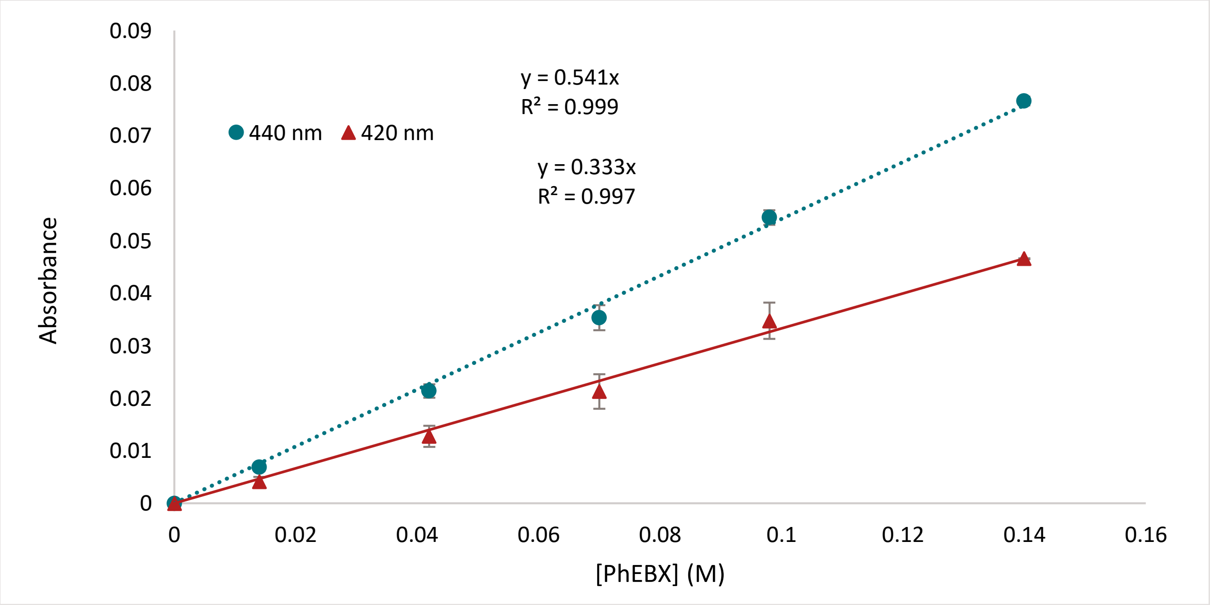

Figure S10. Beer-Lambert linear regression for 420 nm and 440 nm

6.6. Cyclic voltammetry of PhEBX (**1a**)

An Autolab potentiostat with a 3 electrode cell configuration: glassy carbon (working electrode), Pt wire as (control electrode), and Ag/AgCl (KCl, 3 M aq.) as (reference electrode) was used for the measures. Tetrabutyl ammonium hexafluorophosphhate (TBAP, 0.1 M in MeCN) was used as an electrolyte. PhEBX (**1a**, 3.5 mg, 0.01 mmol) was dissolved in a stock solution of TBAP (0.1 M, 10 mL in MeCN) and was degassed by bubbling Argon directly before measure. The redox couple E(**1a**/**1a**$^{\bullet-}$) is defined as the potential E measured for $\frac{I_{max}}{2}$.

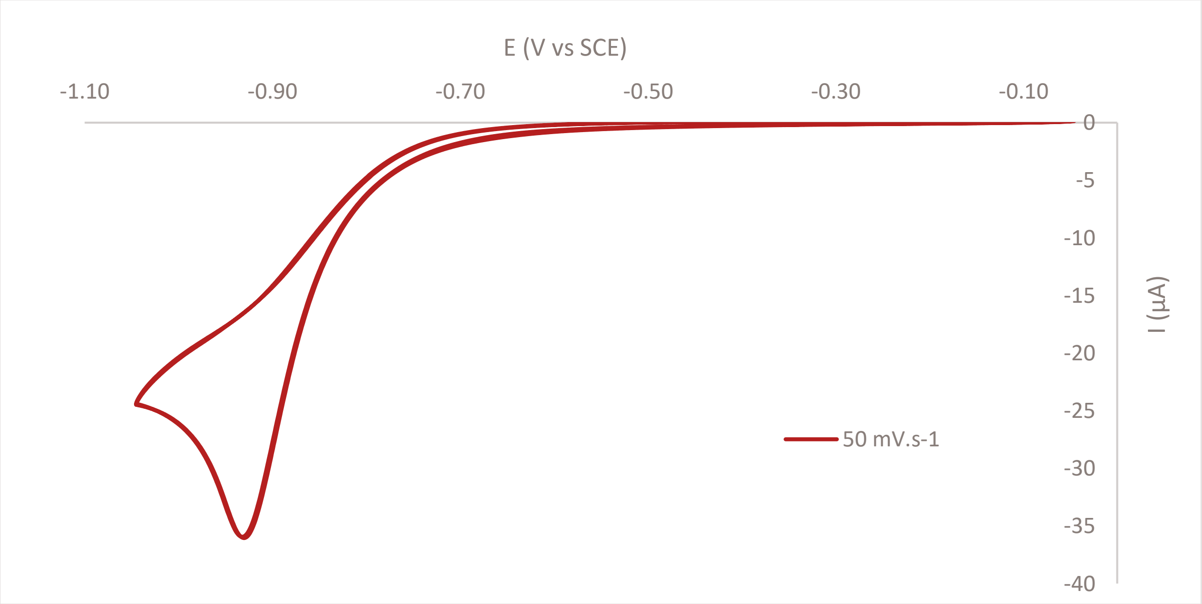

Figure S11. Cyclic voltammogram of **1a**

$$I_{max} = 36\ \mu A;\ \frac{I_{max}}{2} = 18\ \mu A\ E = \ -0.87\ V\ vs\ SCE\ for\ I = 18\mu A$$

$$E_{1/2}(\mathbf{1a}/\mathbf{1a}^{\bullet-}) = \ -0.87\ V\ vs\ SCE$$

$E_{1/2}$(**1a***/**1a**$^{\bullet-}$) = $E_{0\text{-}0}$ + $E_{1/2}$(**1a**/**1a**$^{\bullet-}$). $E_{0\text{-}0}$ was determined experimentally by position of the long wavelength tail of the absorption spectrum at 460 nm (Figure S8).[37]

$$E = \frac{hc}{\lambda}$$

$$E_{0-0} = \frac{1240}{460} = 2.7\ eV$$

$$E_{1/2}(\mathbf{1a}^{*}/\mathbf{1a}^{\bullet-}) = \ E_{0-0} + \ E_{1/2}(\mathbf{1a}/\mathbf{1a}^{\bullet-}) = \ 2.7 - 0.87 = 1.83 = +1.8\ V\ vs\ SCE$$

7. NMR spectra of new compounds

[37] Buzzetti, L.; Prieto, A.; Roy, S. R.; Melchiorre, P. *Angew. Chem. Int. Ed.* **2017**, *56* (47), 15039–15043.

Compound **1h**

^{1}H NMR, $CDCl_3$, 400 MHz

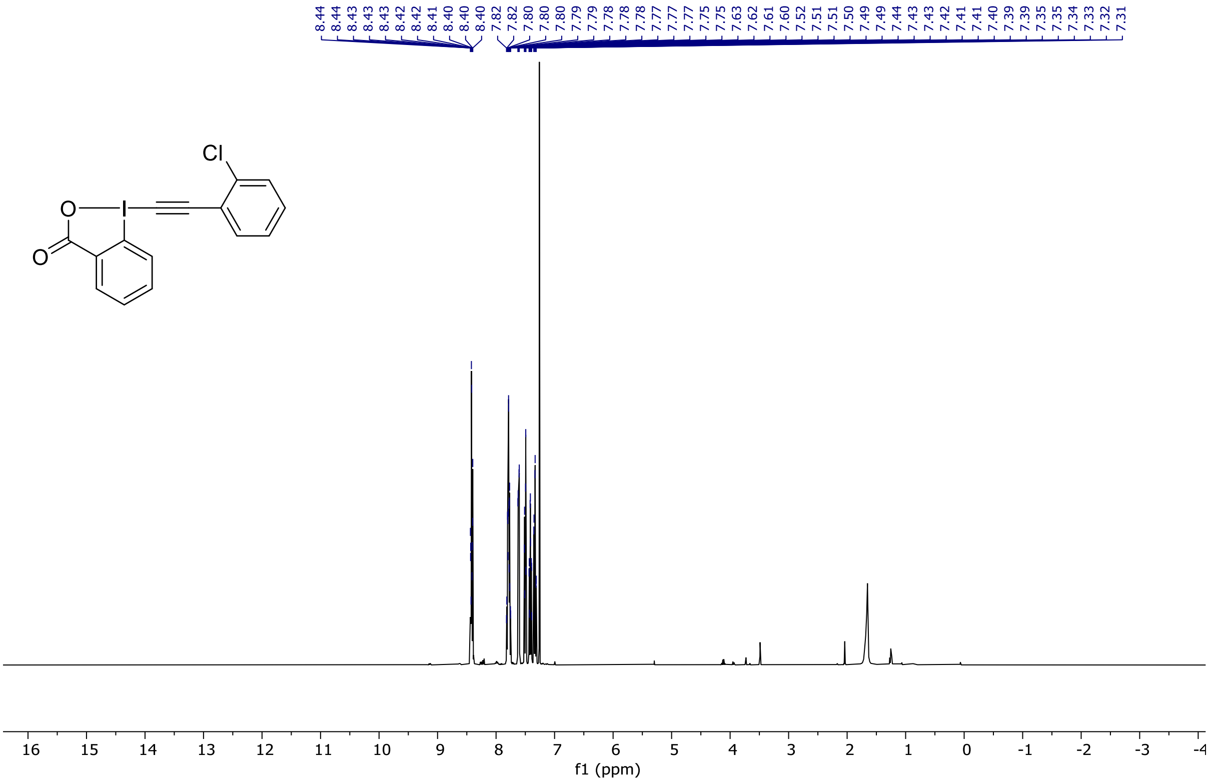

^{13}C NMR, $CDCl_3$, 101 MHz

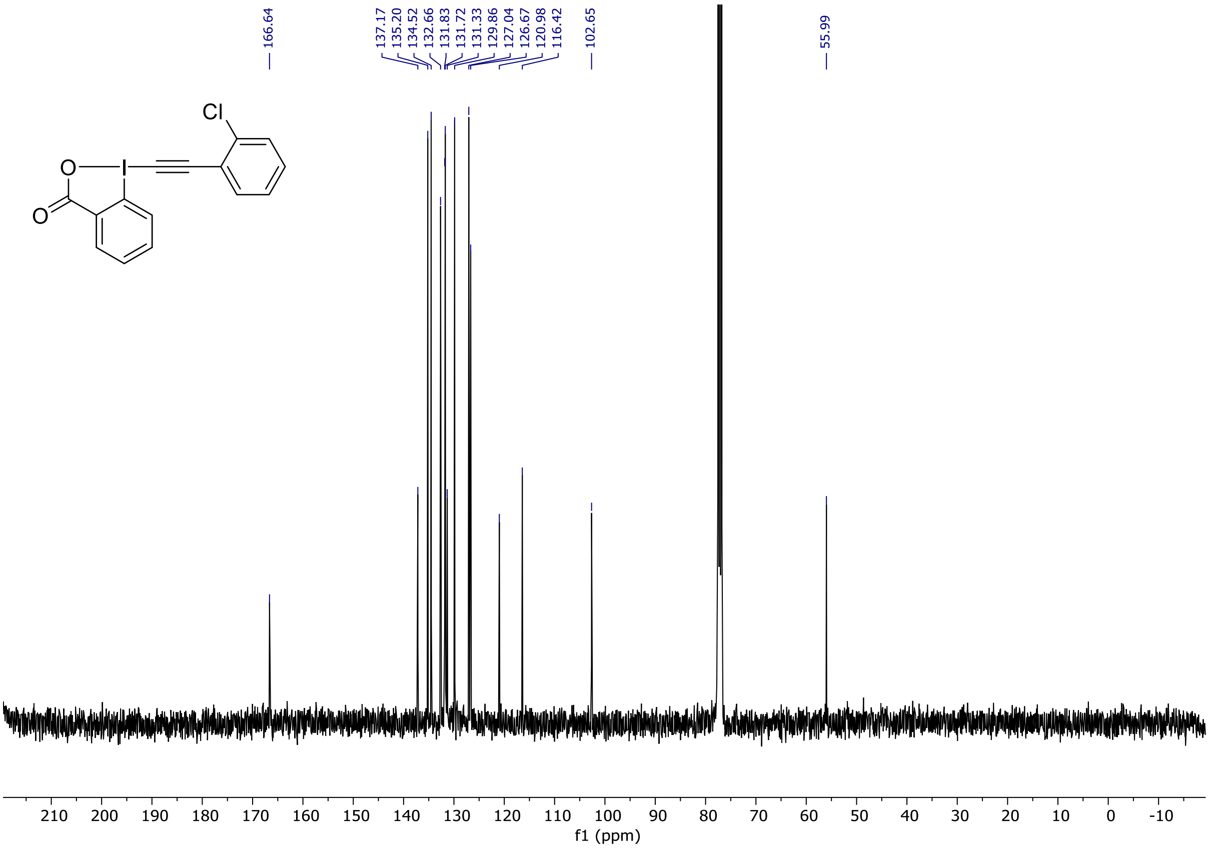

Compound **26e**

^1H NMR, $CDCl_3$, 400 MHz

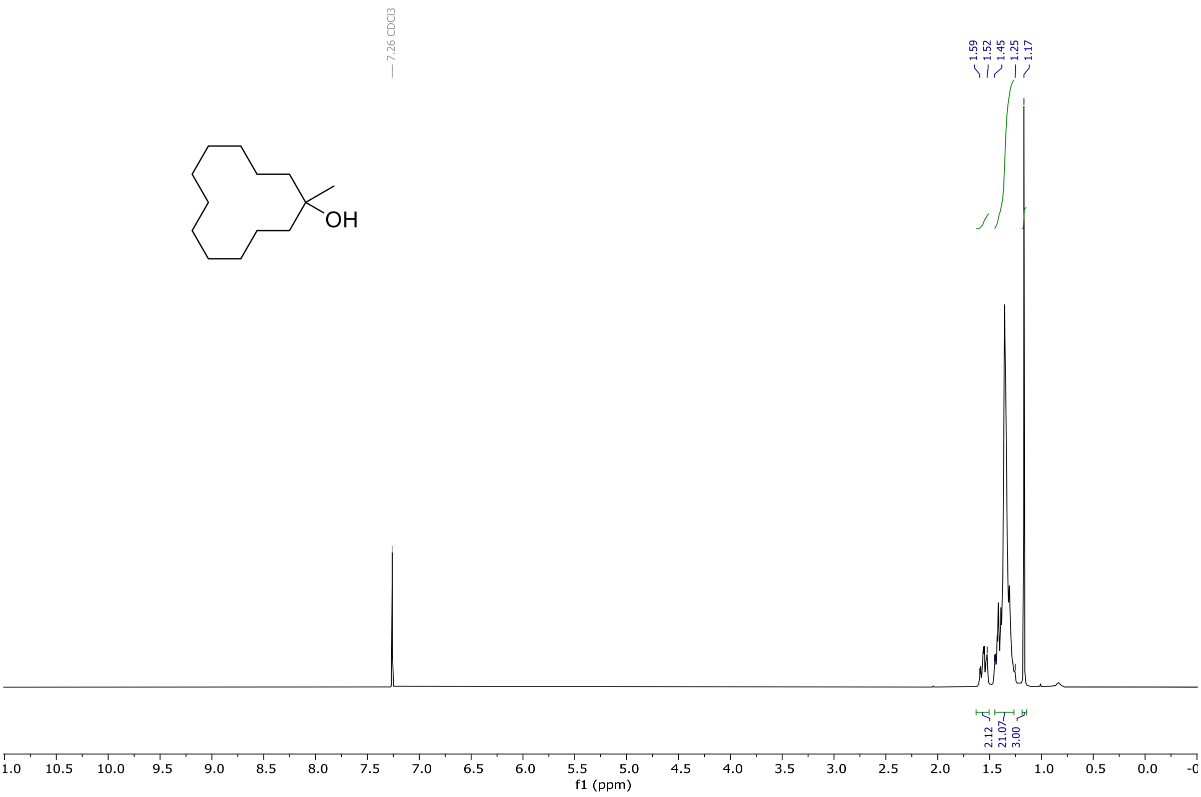

^{13}C NMR, $CDCl_3$, 101 MHz

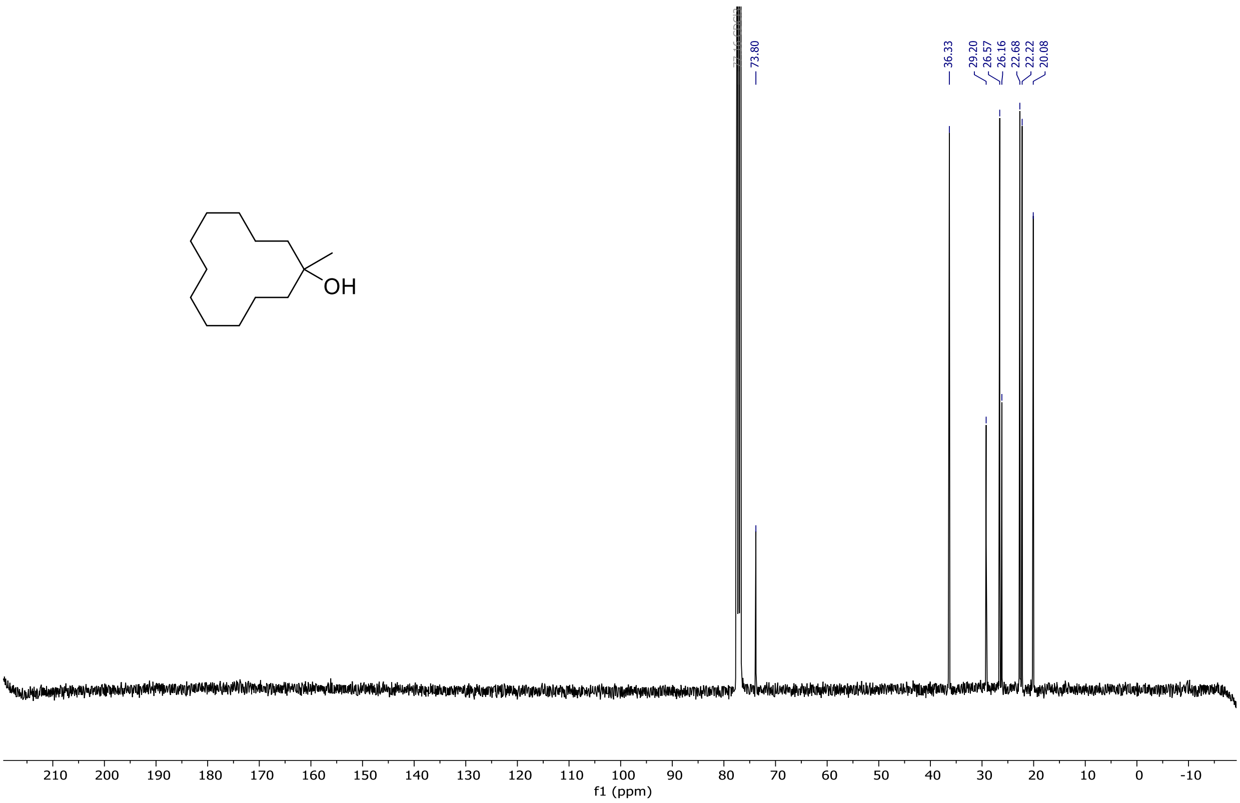

Compound **26f**

^{1}H NMR, $CDCl_3$, 400 MHz

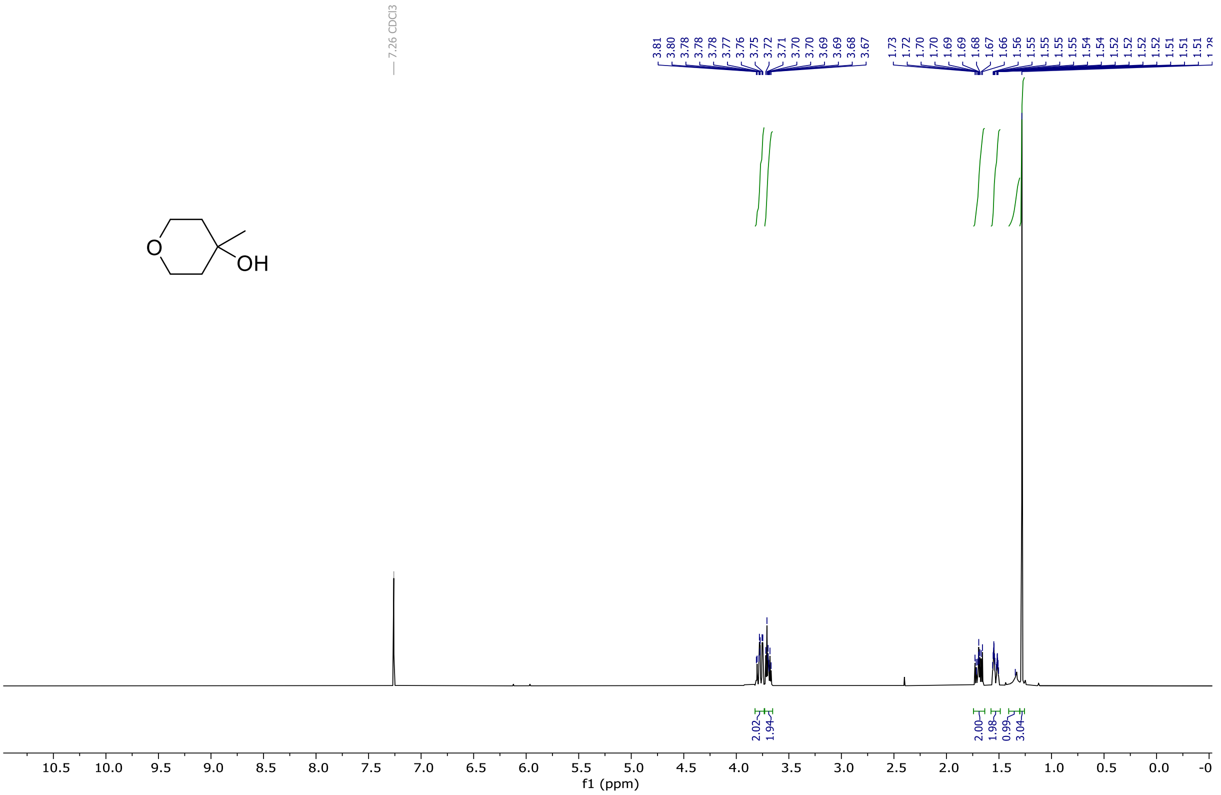

^{13}C NMR, $CDCl_3$, 101 MHz

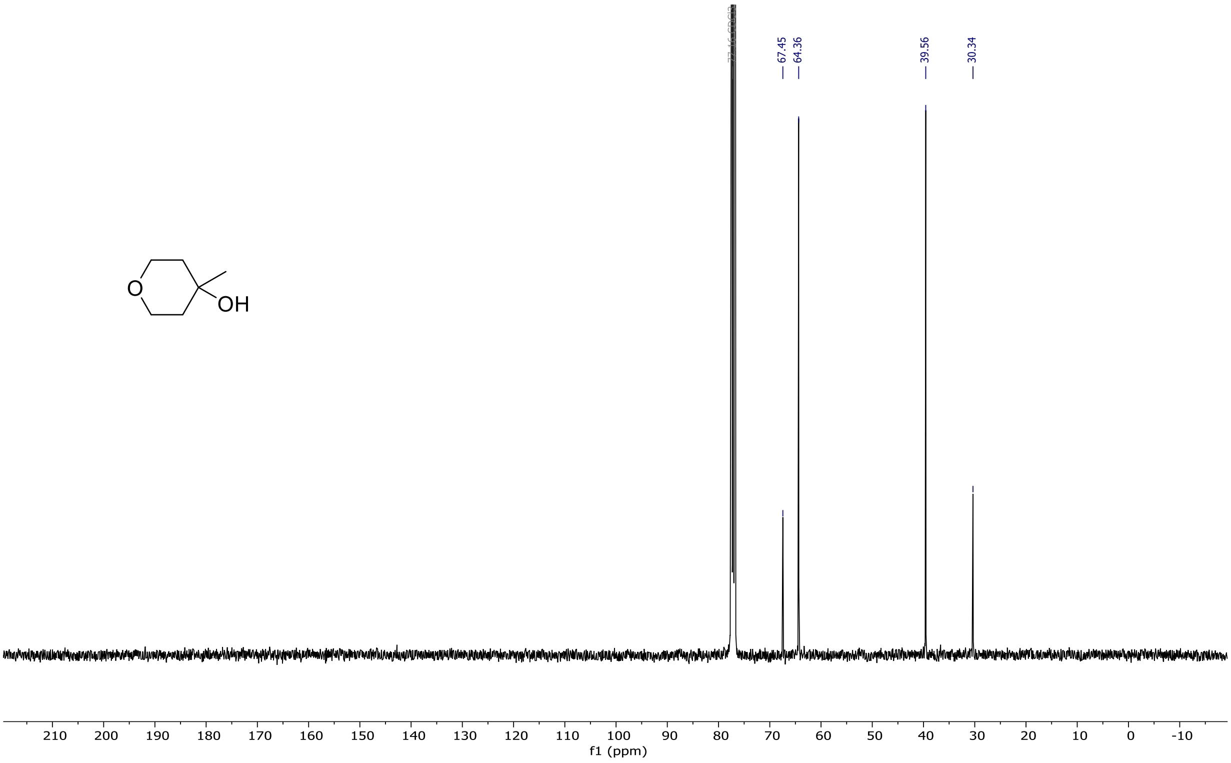

Compound **26m**

^{1}H NMR, $CDCl_3$, 400 MHz

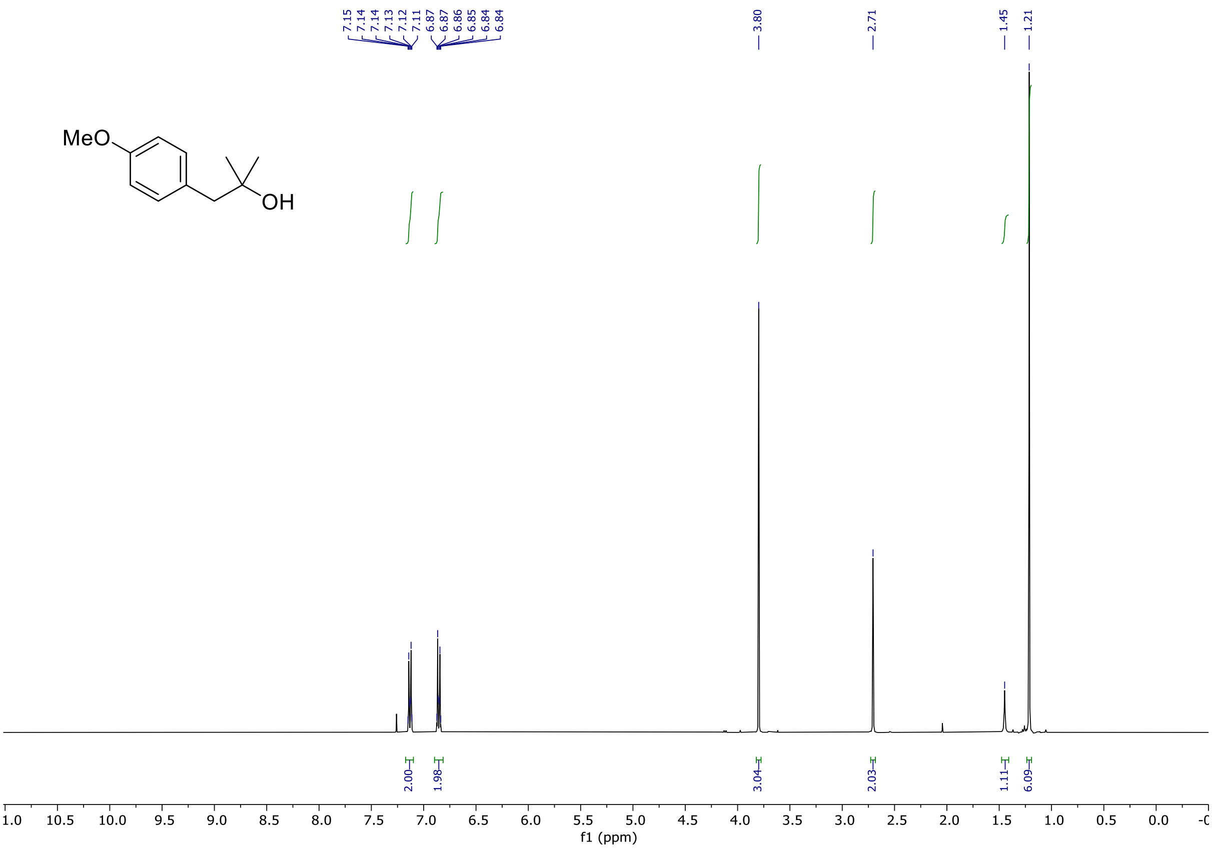

^{13}C NMR, $CDCl_3$, 101 MHz

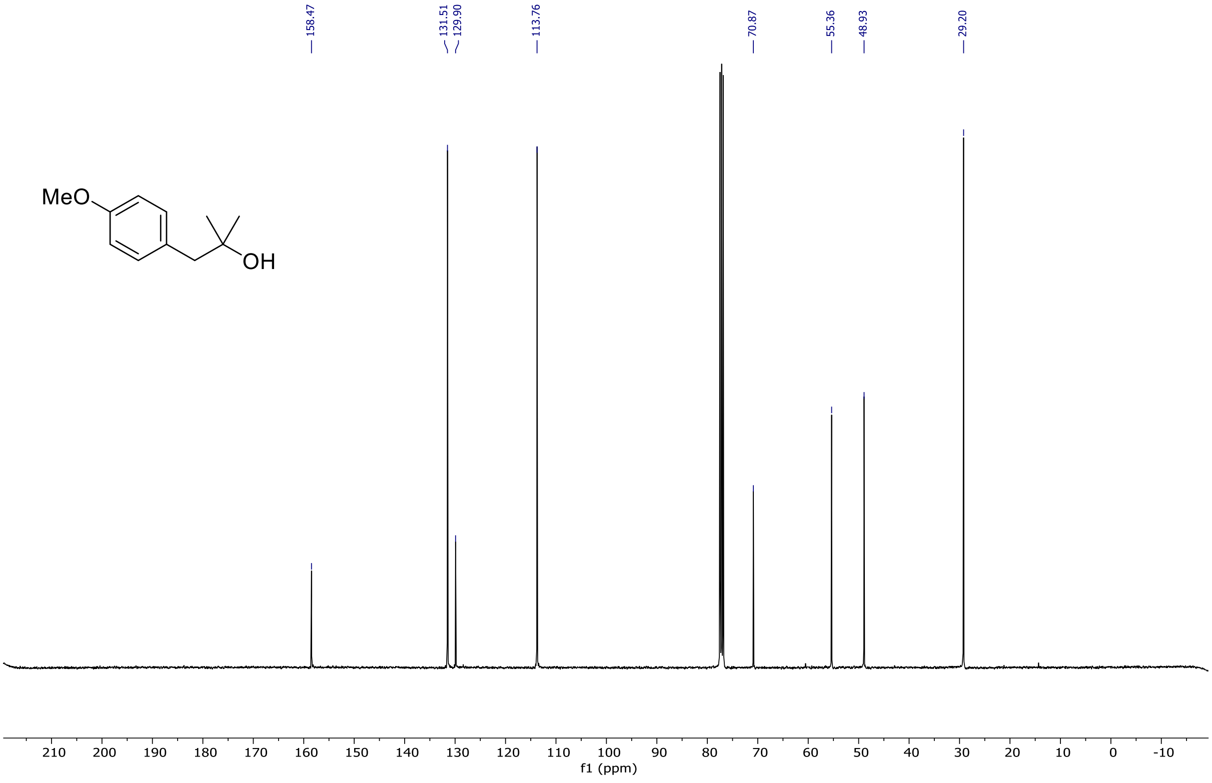

Compound **26n**

^{1}H NMR, $CDCl_3$, 400 MHz

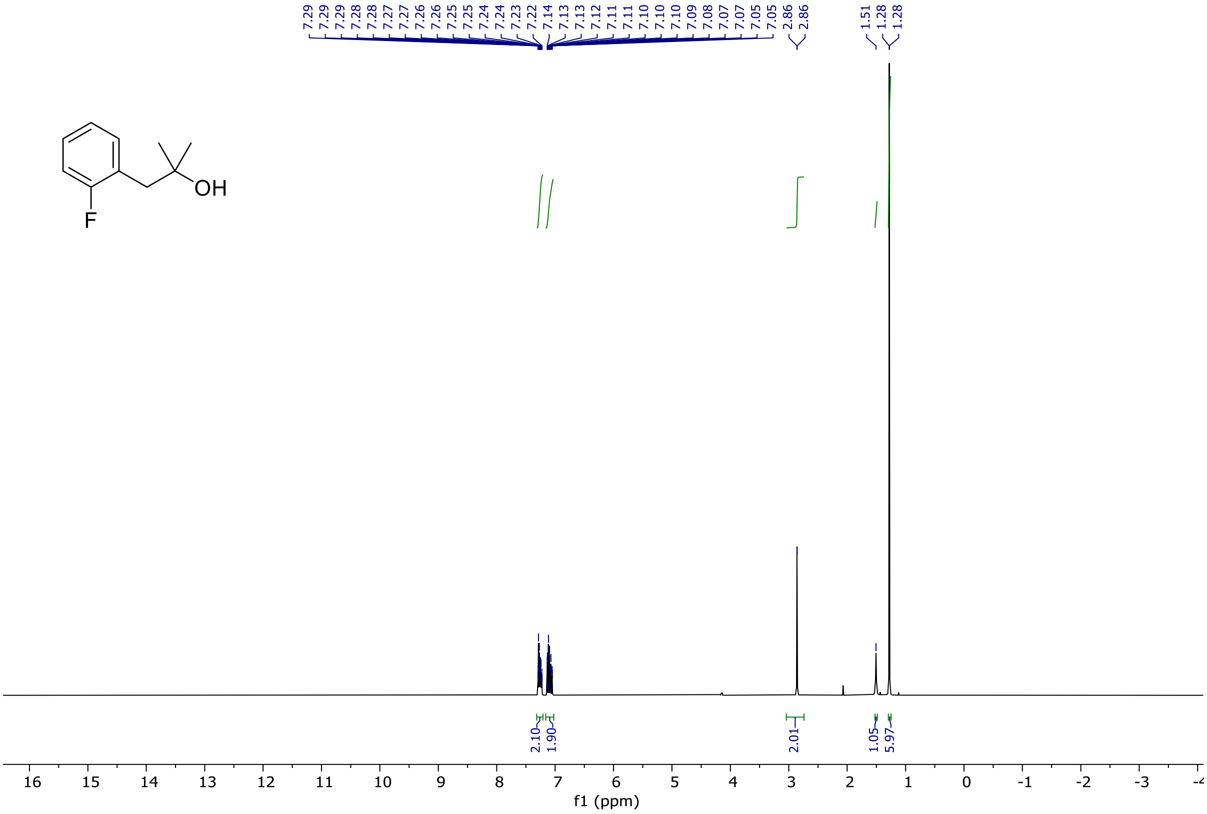

^{13}C NMR, $CDCl_3$, 101 MHz

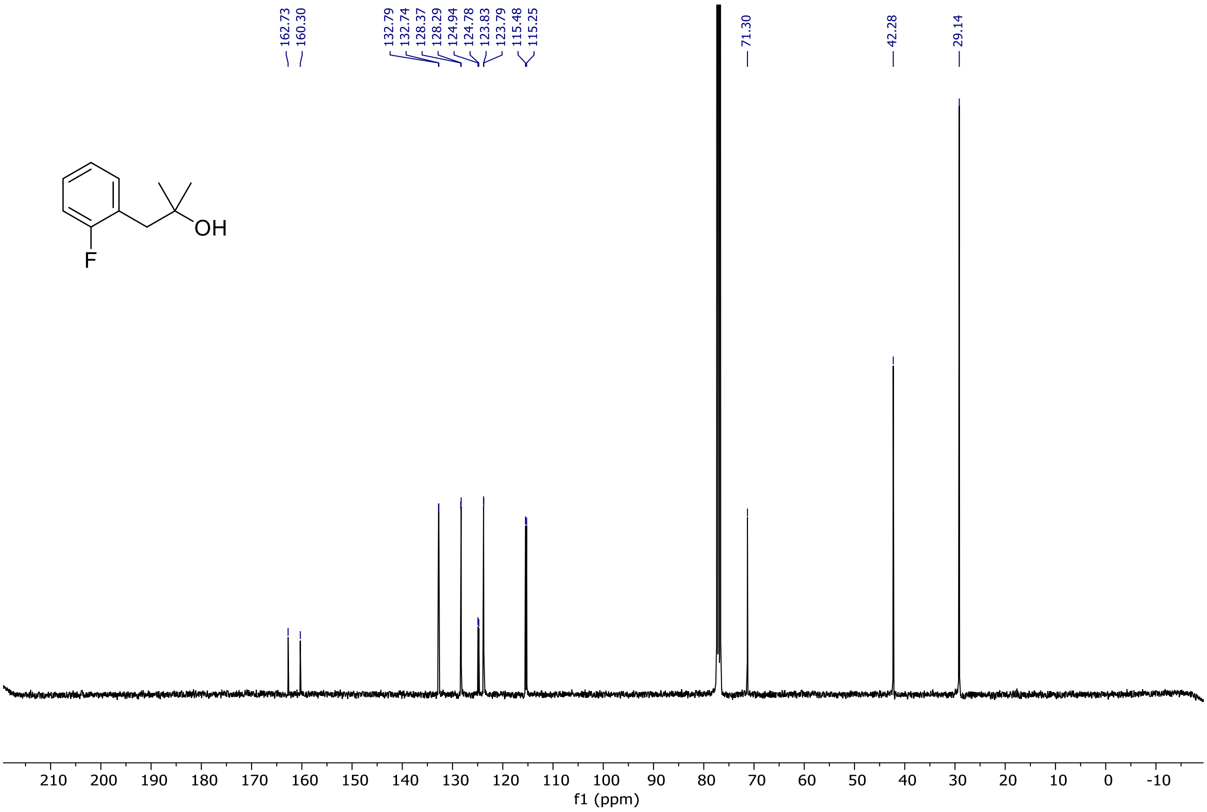

^{19}F NMR, $CDCl_3$, 376 MHz

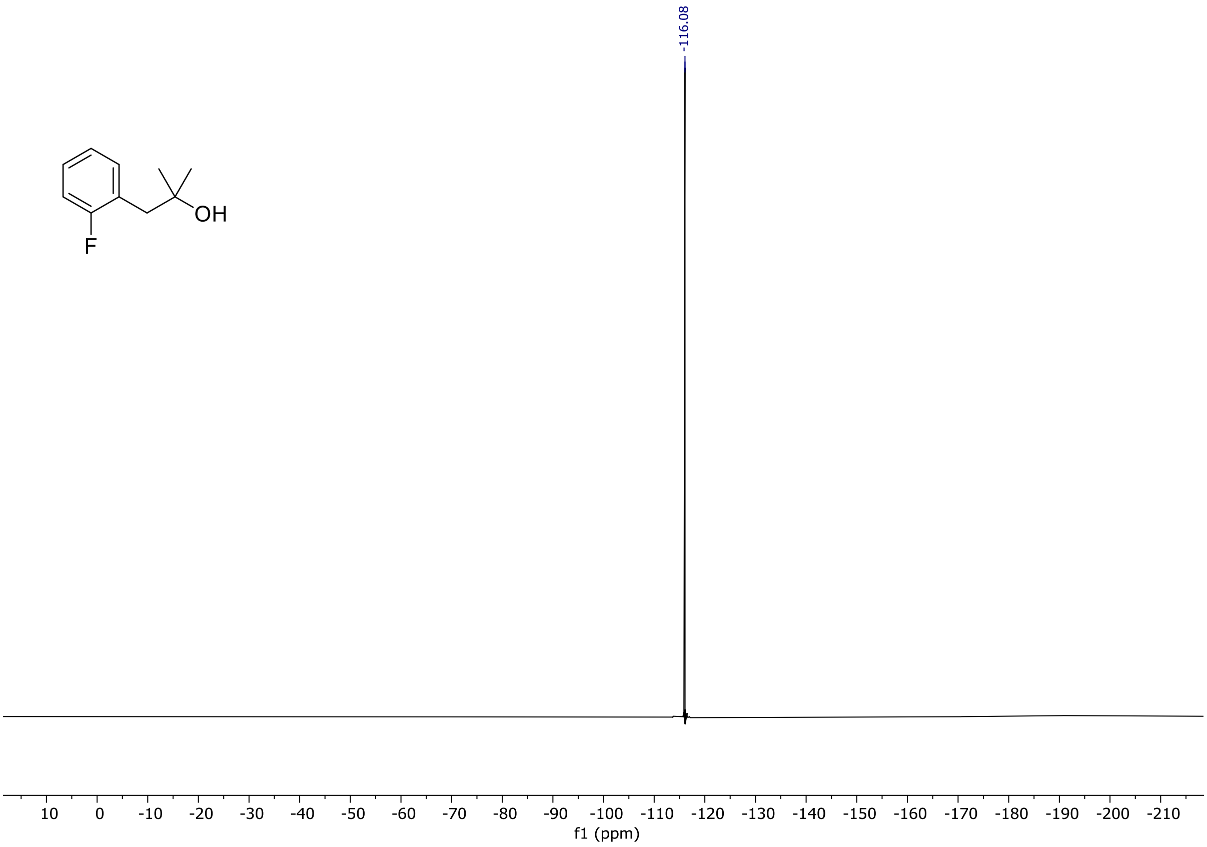

Compound **28a**

^{1}H NMR, $CDCl_3$, 400 MHz

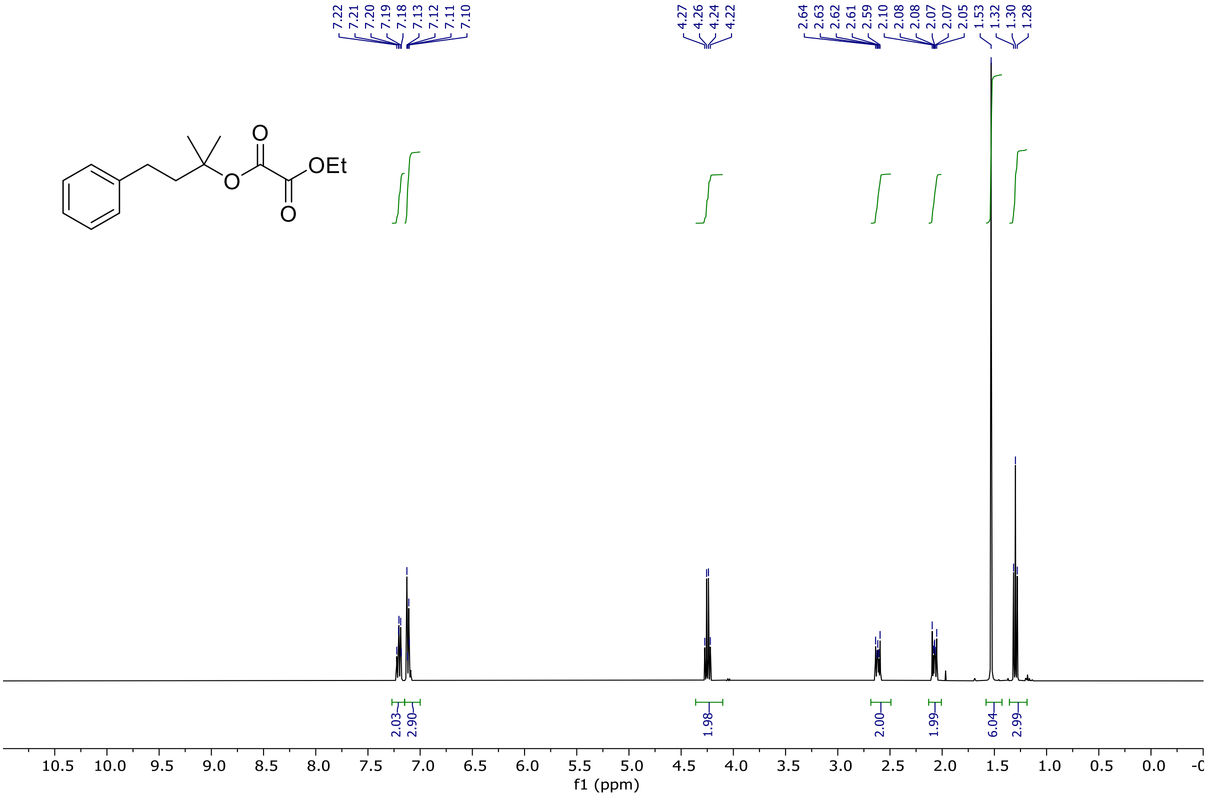

^{13}C NMR, $CDCl_3$, 400 MHz

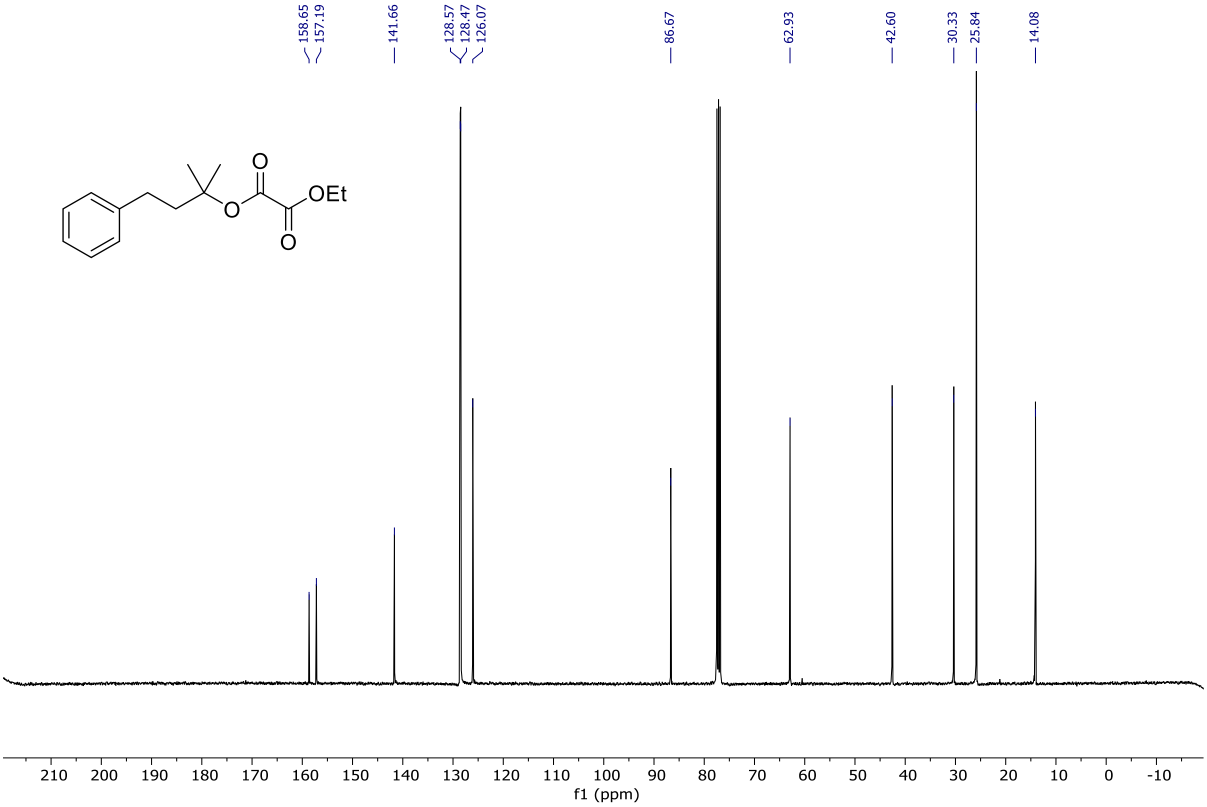

Compound **3a**

^1H NMR, D_2O, 400 MHz

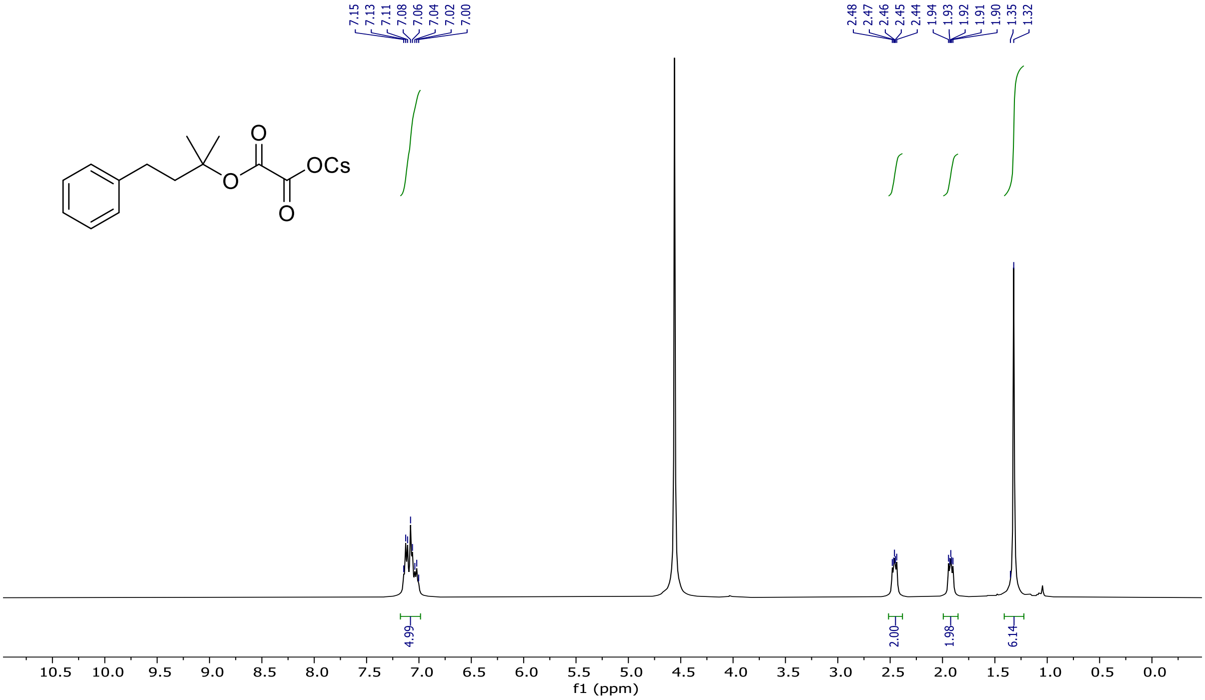

^{13}C NMR, D_2O, 101 MHz

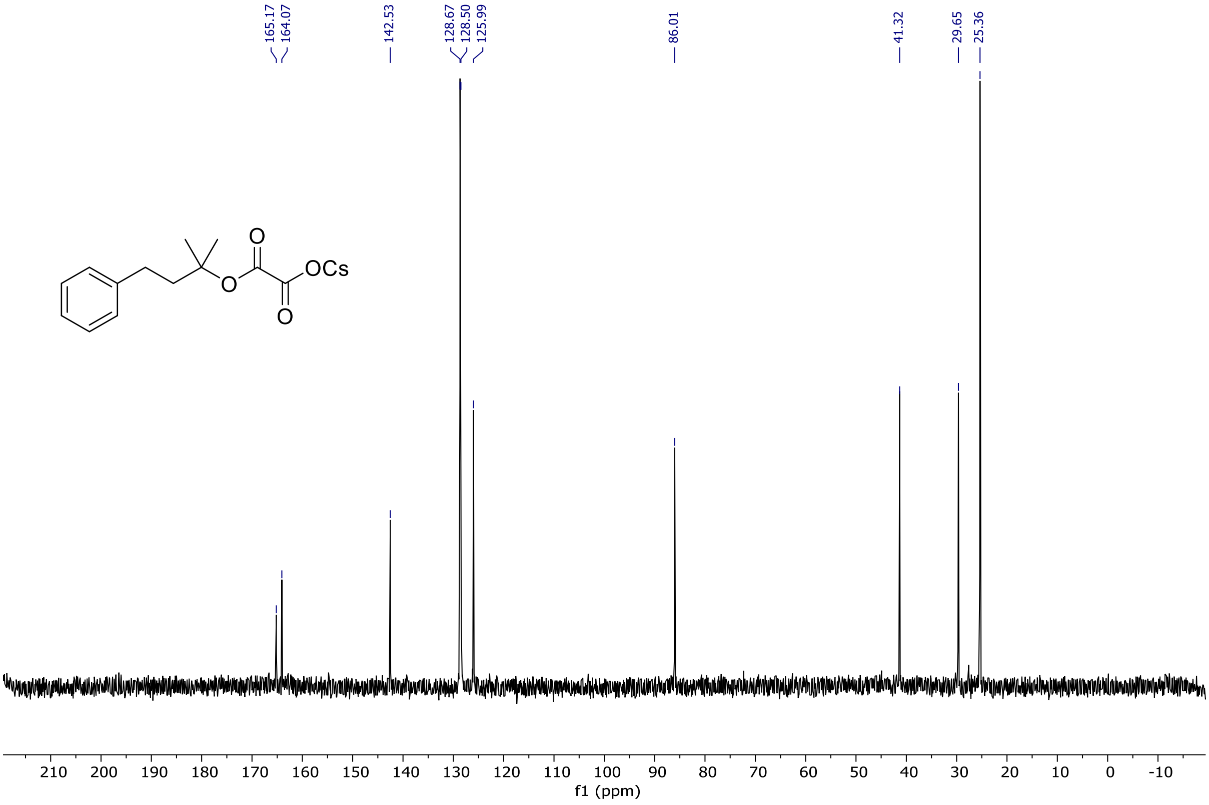

Compound **3b**

^{1}H NMR, DMSO, 400 MHz

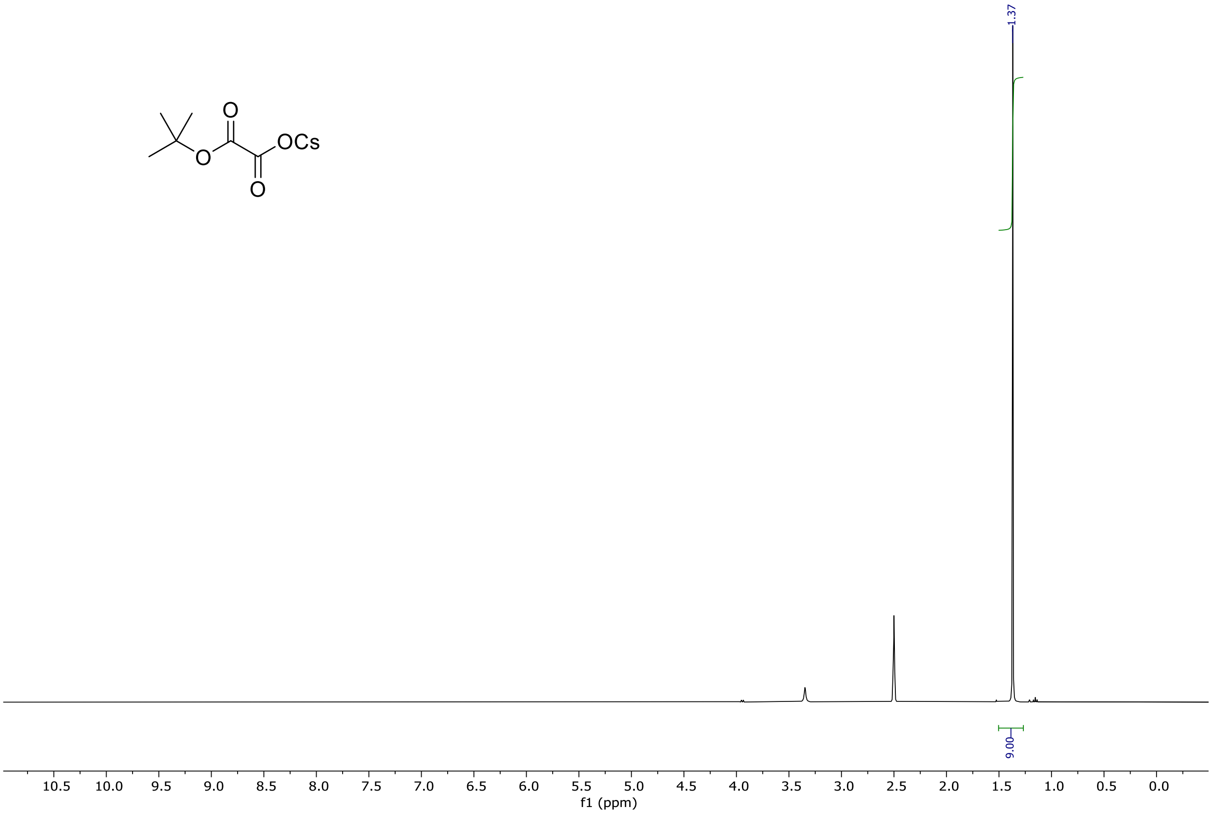

^{13}C NMR, DMSO, 101 MHz

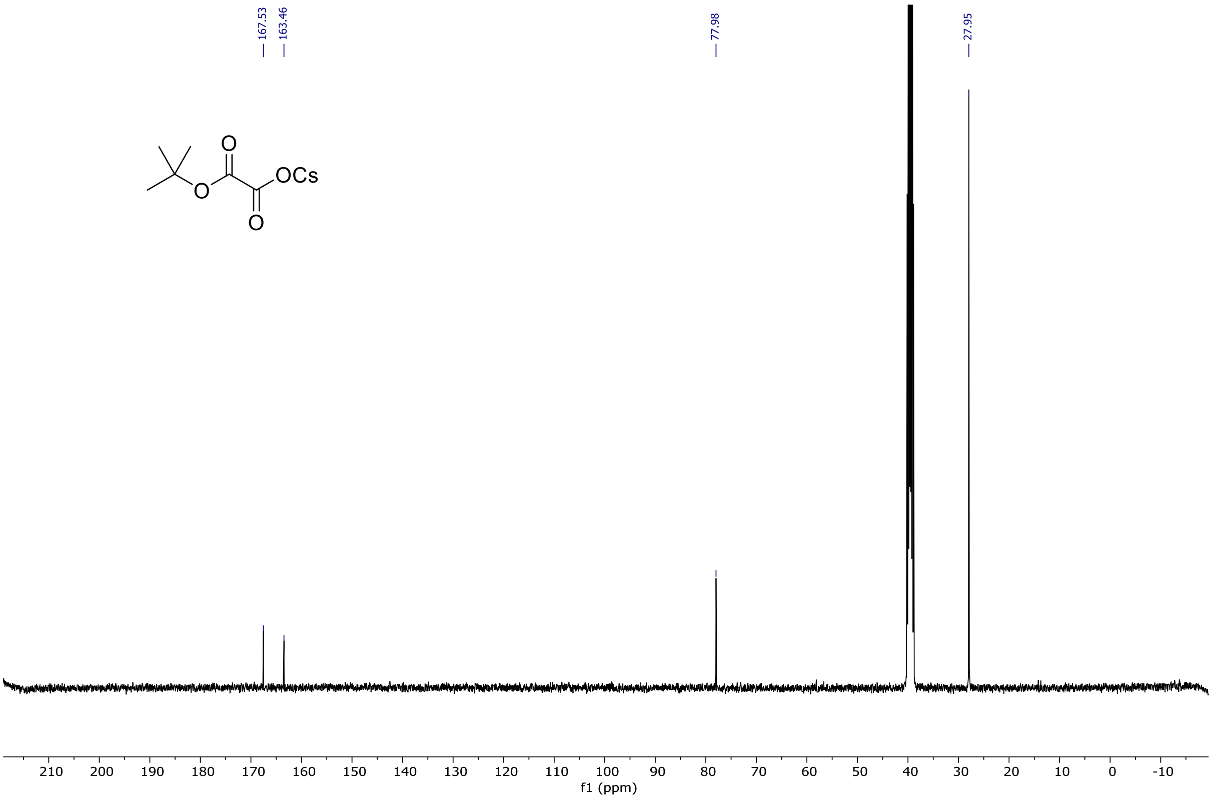

Compound **28c**

^{1}H NMR, $CDCl_3$, 400 MHz

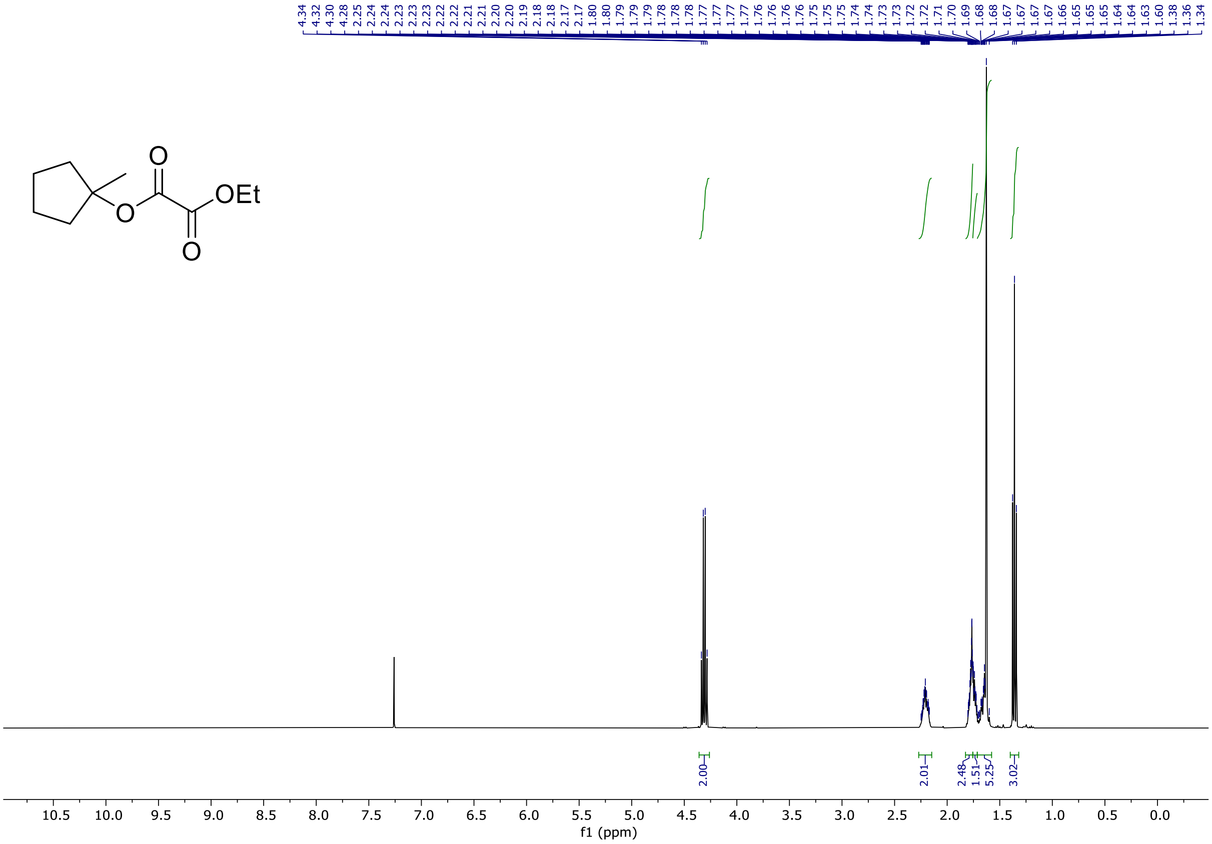

^{13}C NMR, $CDCl_3$, 101 MHz

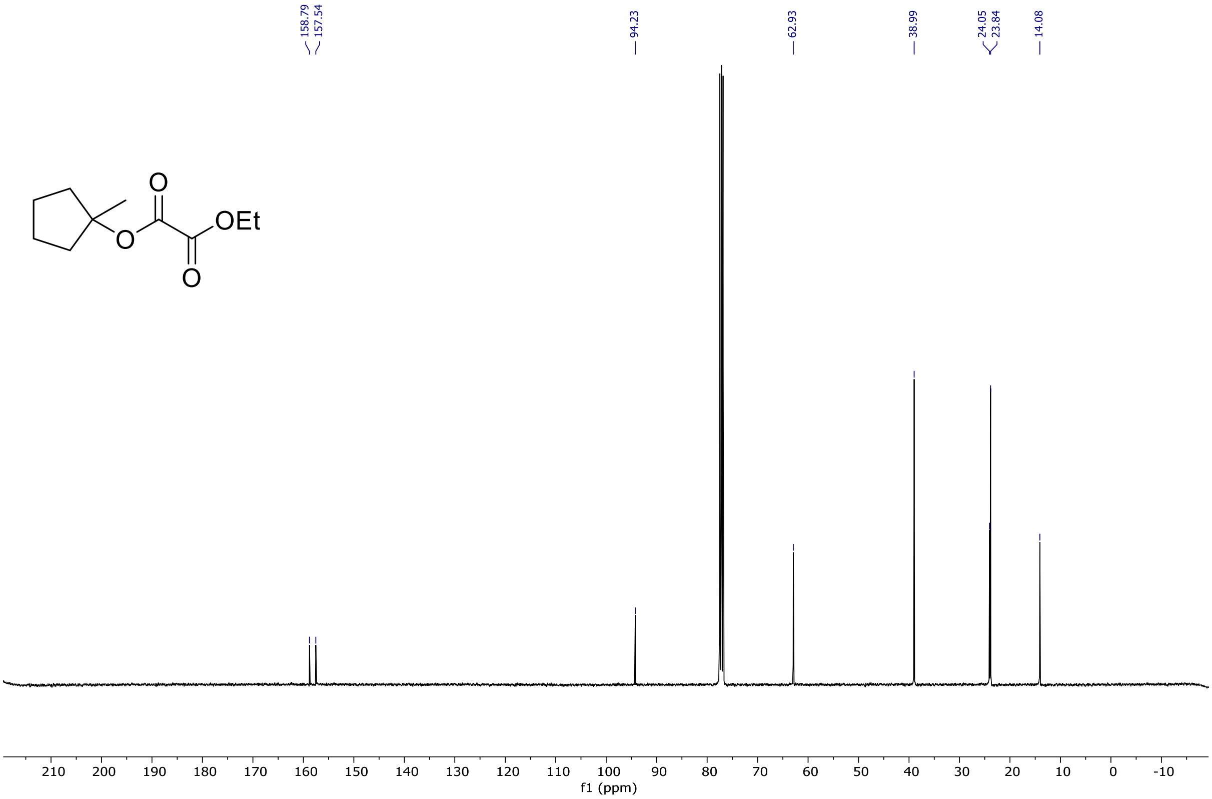

Compound **3c**

^{1}H NMR, DMSO, 400 MHz

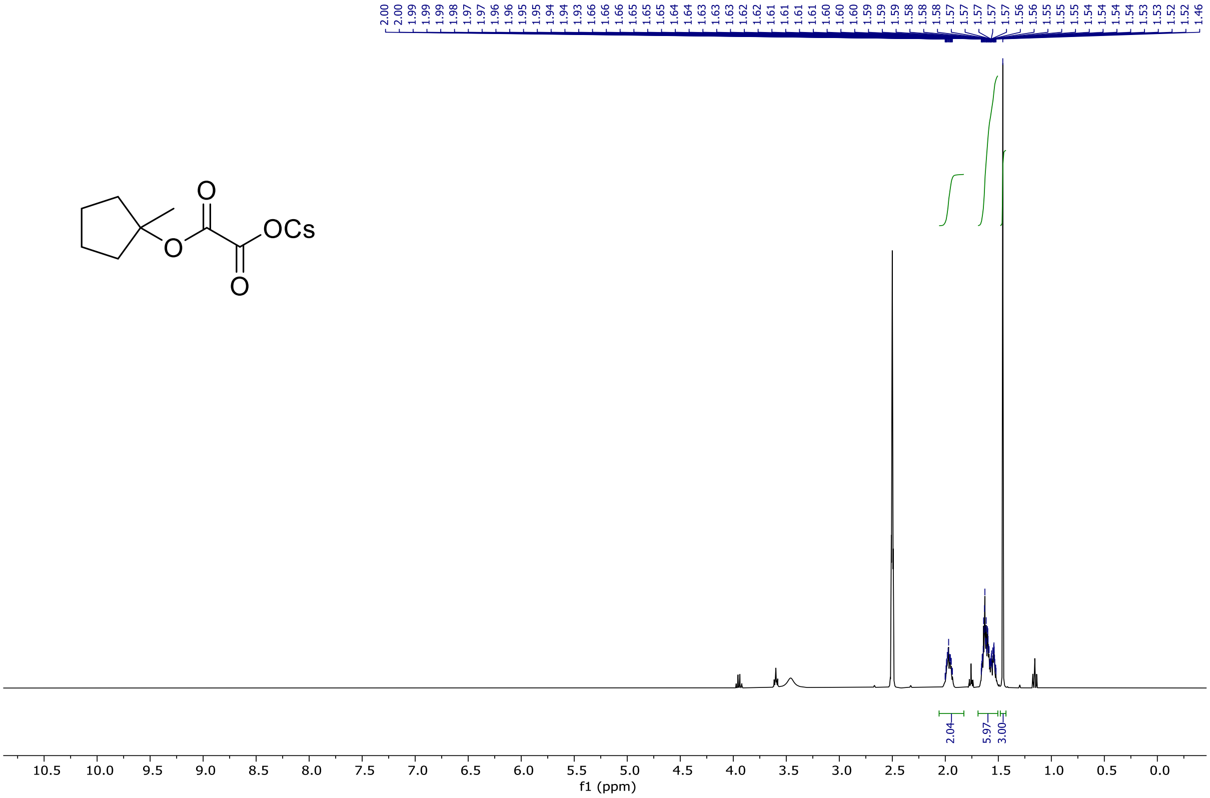

^{13}C NMR, DMSO, 101 MHz

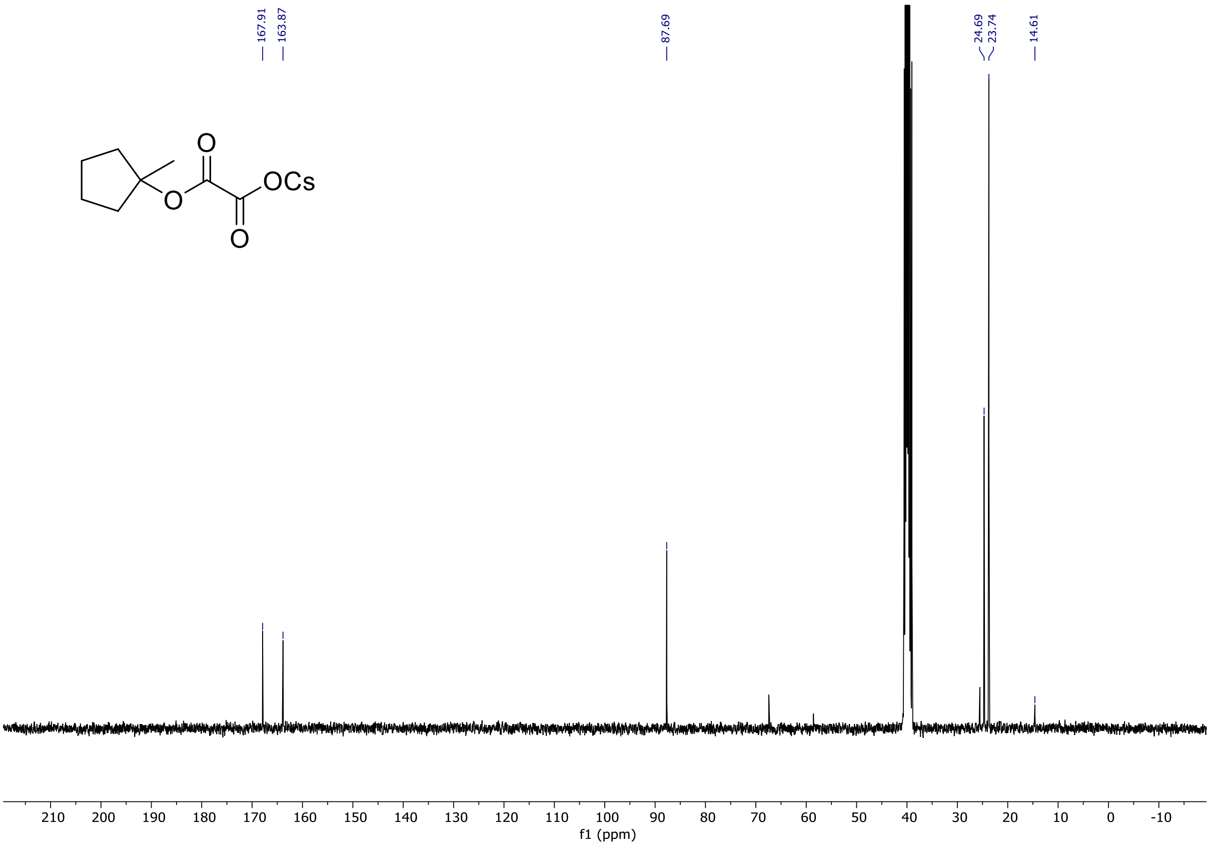

Compound **28d**

^{1}H NMR, $CDCl_3$, 400 MHz

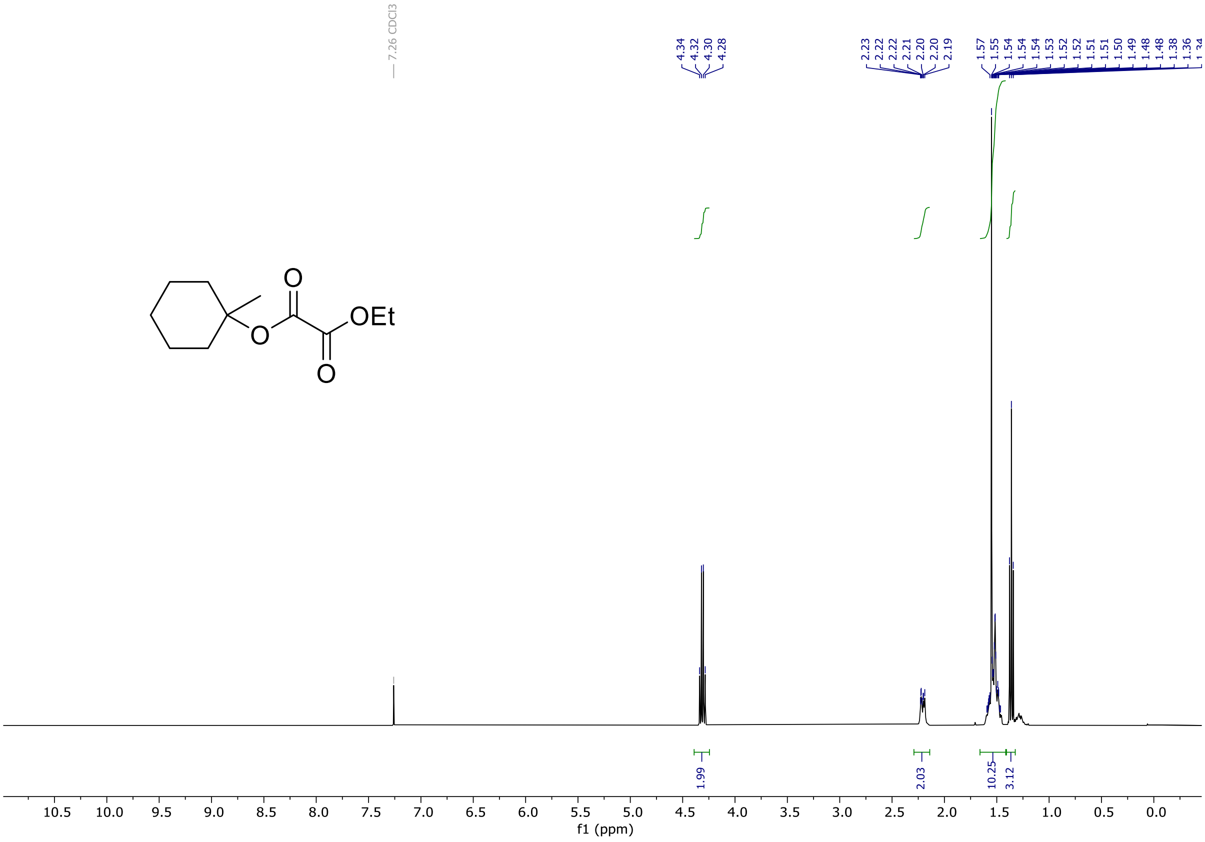

^{13}C NMR, $CDCl_3$, 101 MHz

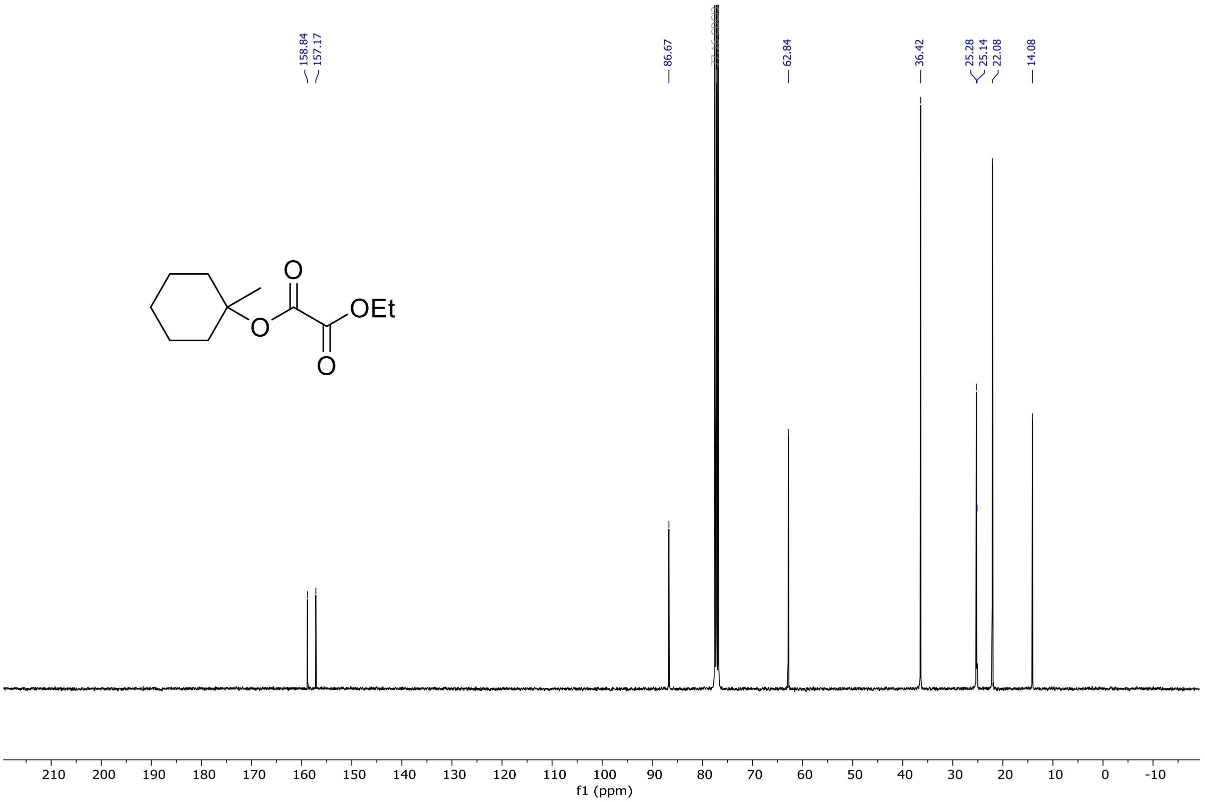

Compound **3d**

^{1}H NMR, DMSO, 400 MHz

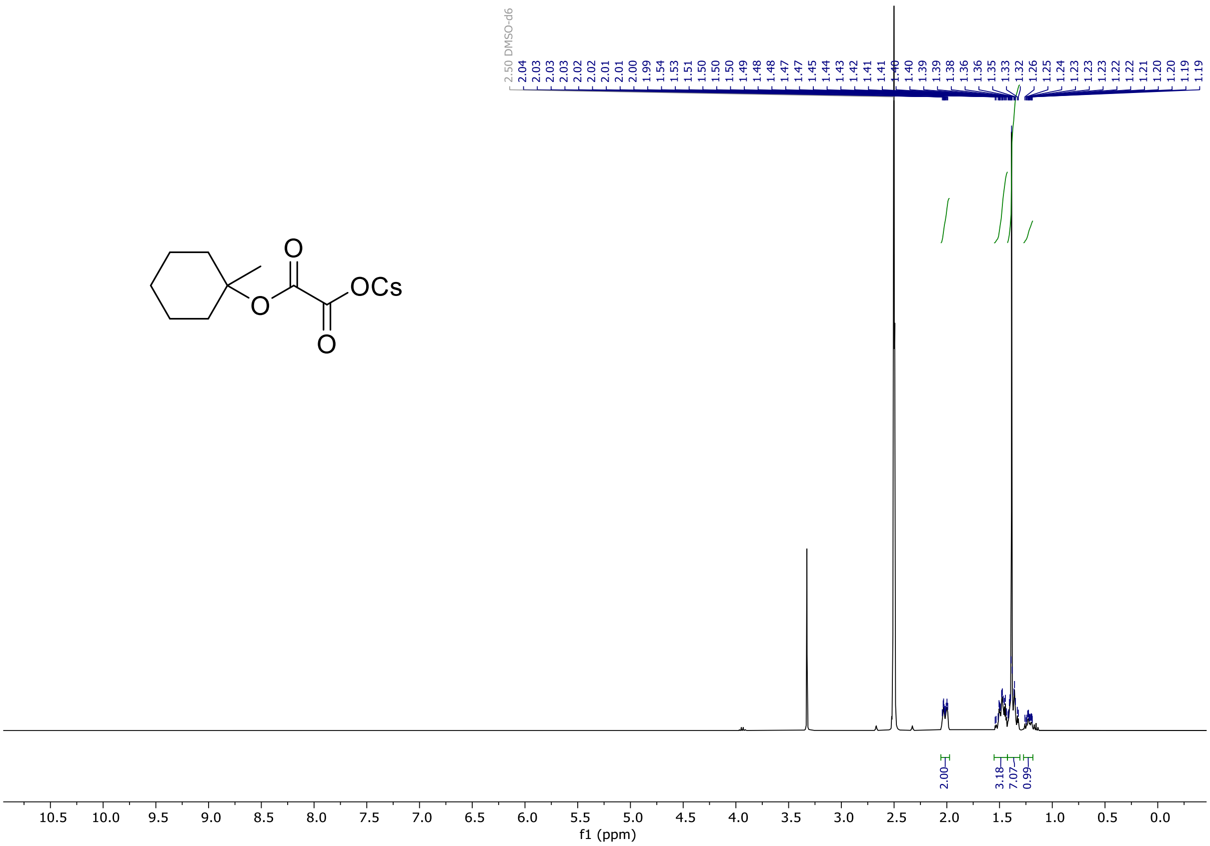

^{13}C NMR, DMSO, 101 MHz

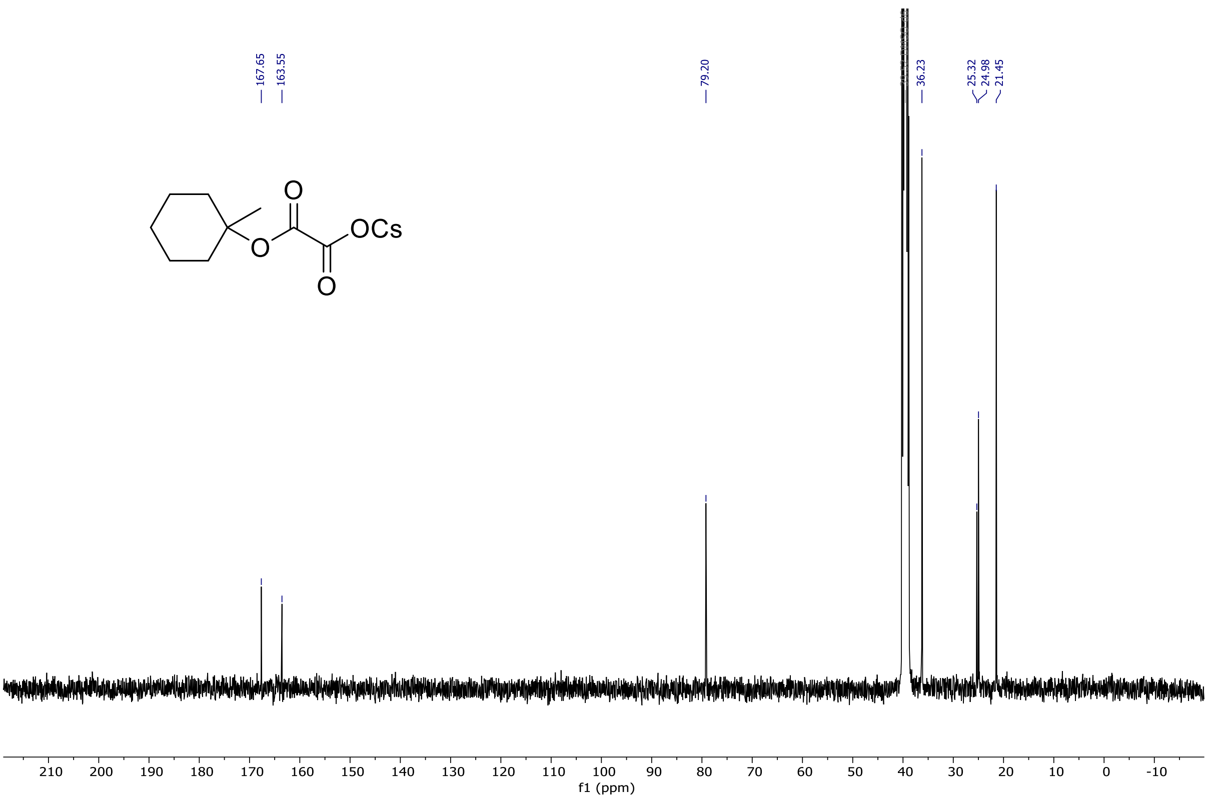

Compound **28e**

^1H NMR, $CDCl_3$, 400 MHz

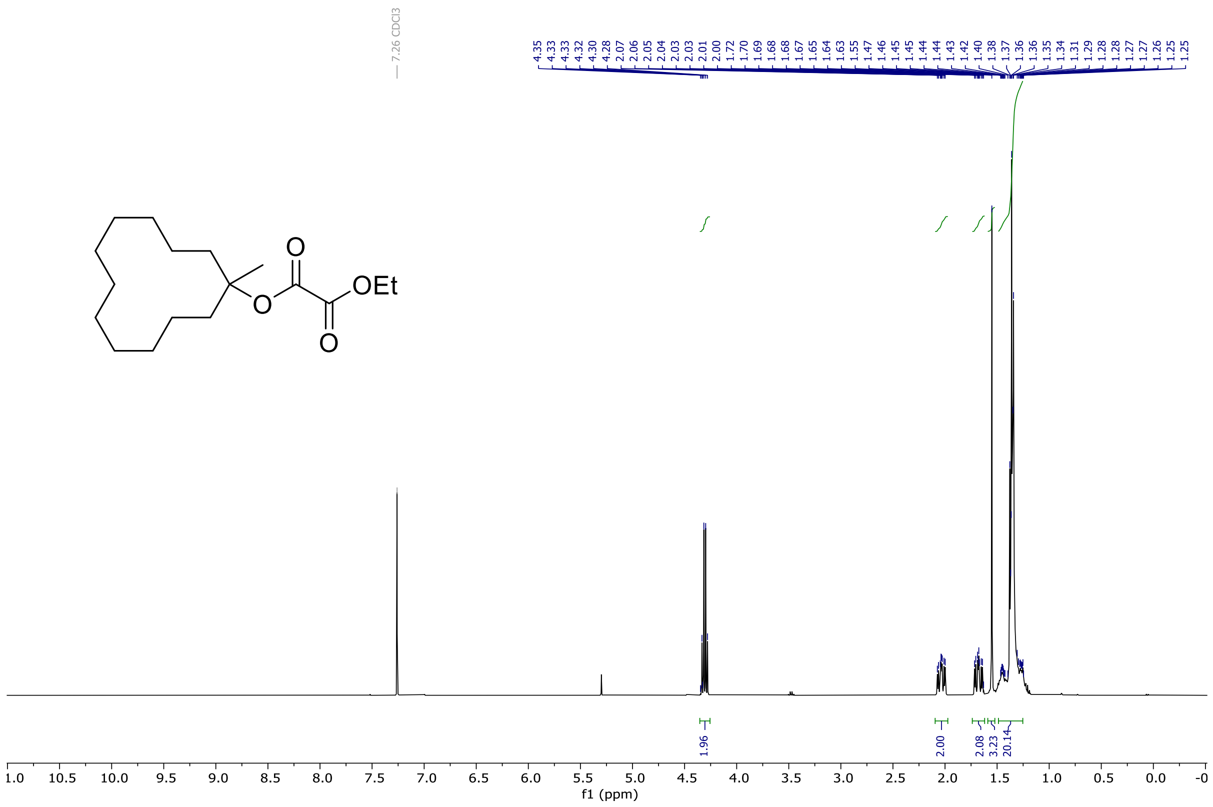

^{13}C NMR, $CDCl_3$, 101 MHz

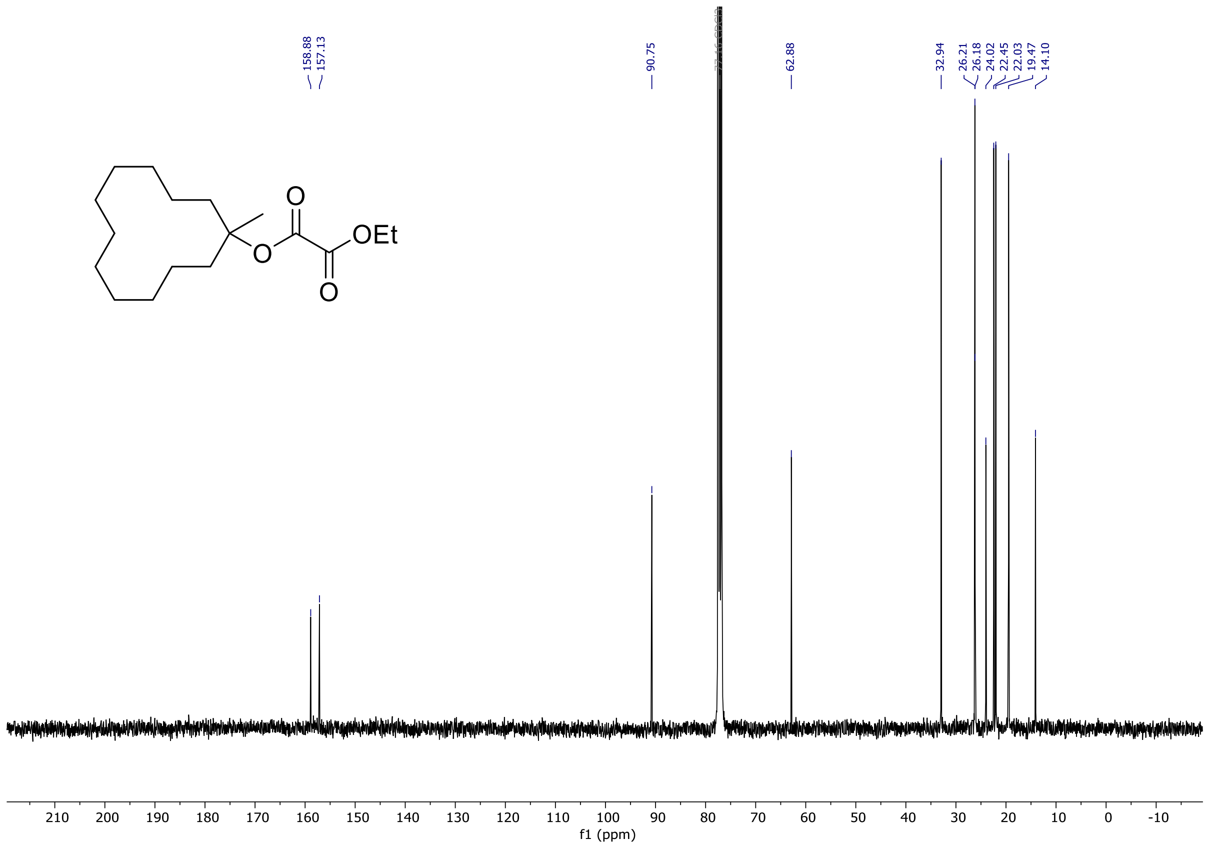

Compound **3e**

^1H NMR, DMSO, 400 MHz

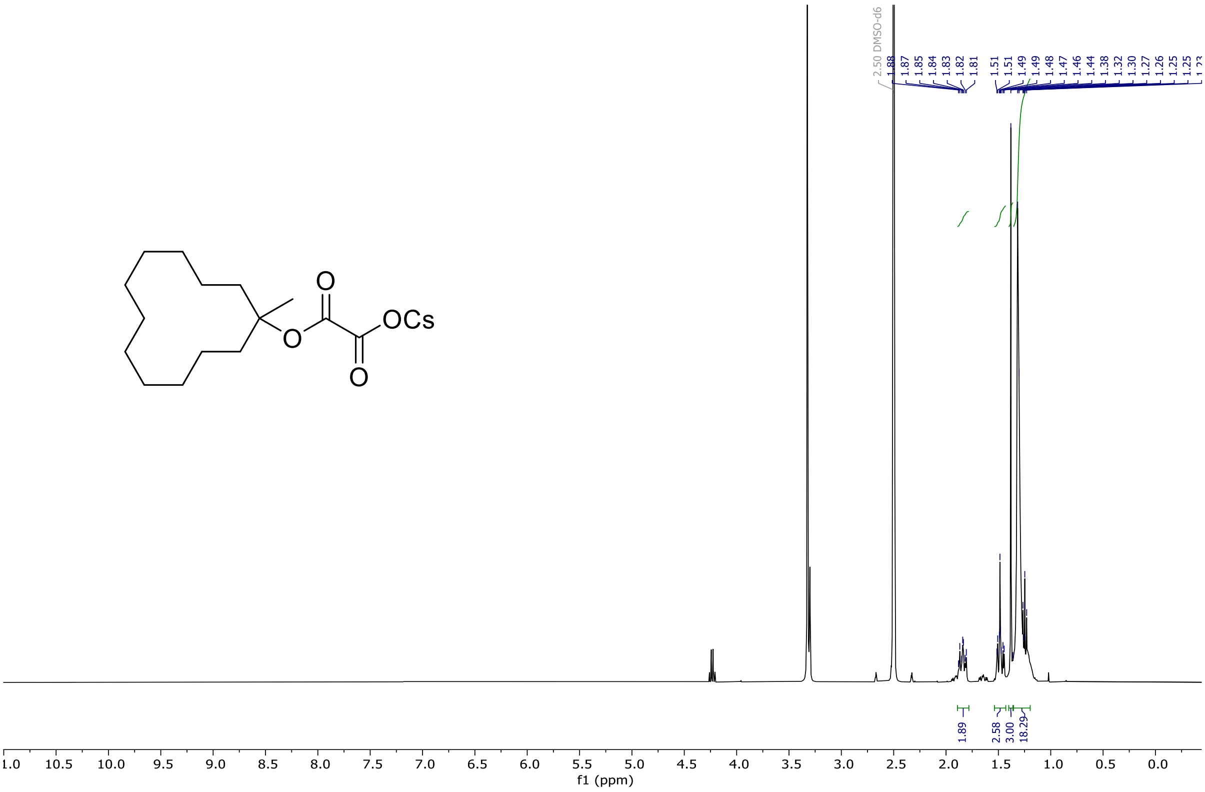

^{13}C NMR, DMSO, 101 MHz

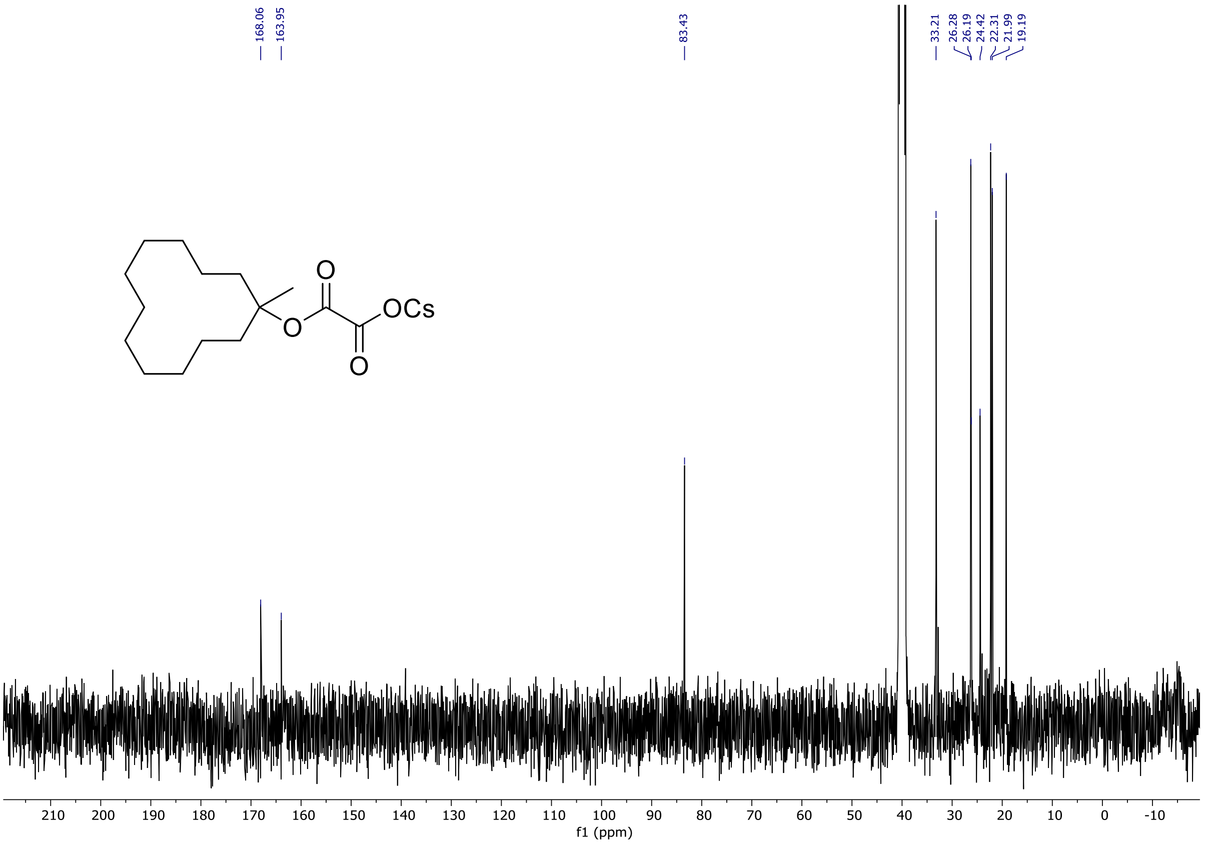

Compound **28f**

^{1}H NMR, $CDCl_3$, 400 MHz

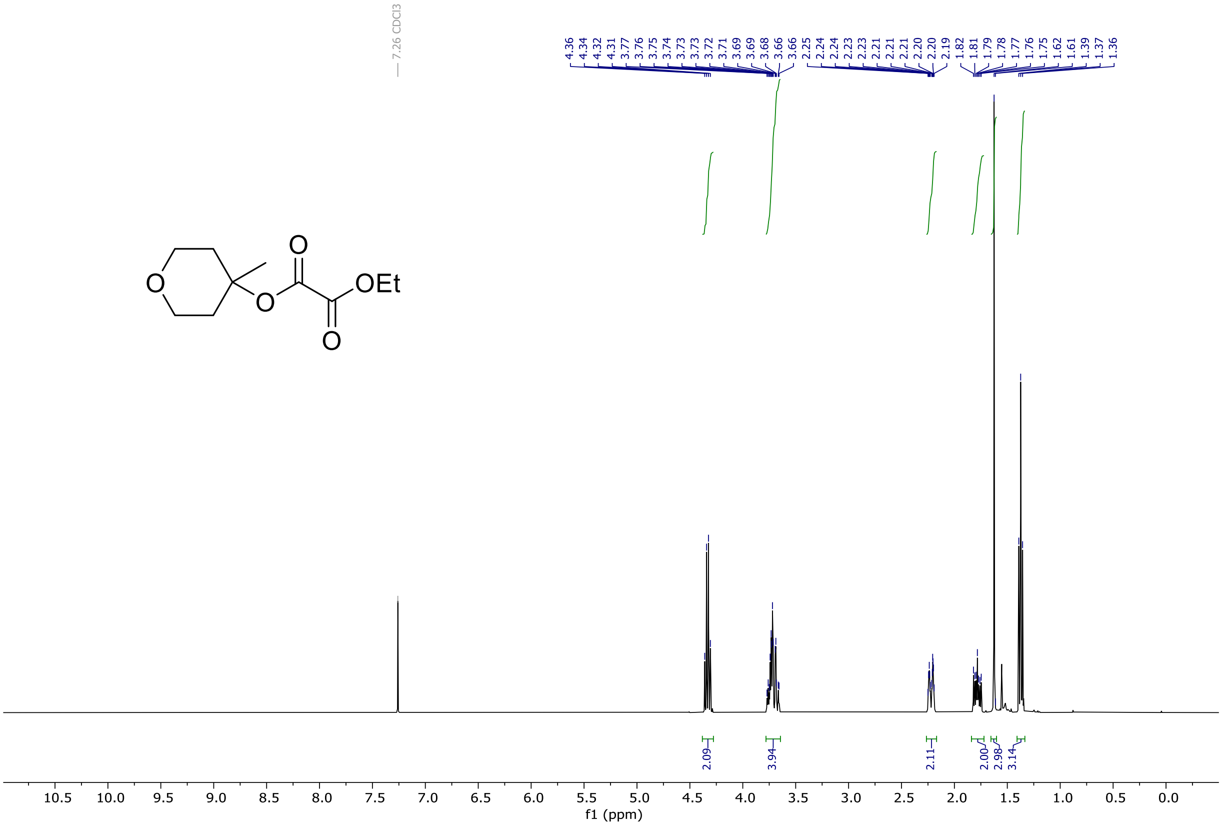

^{13}C NMR, $CDCl_3$, 101 MHz

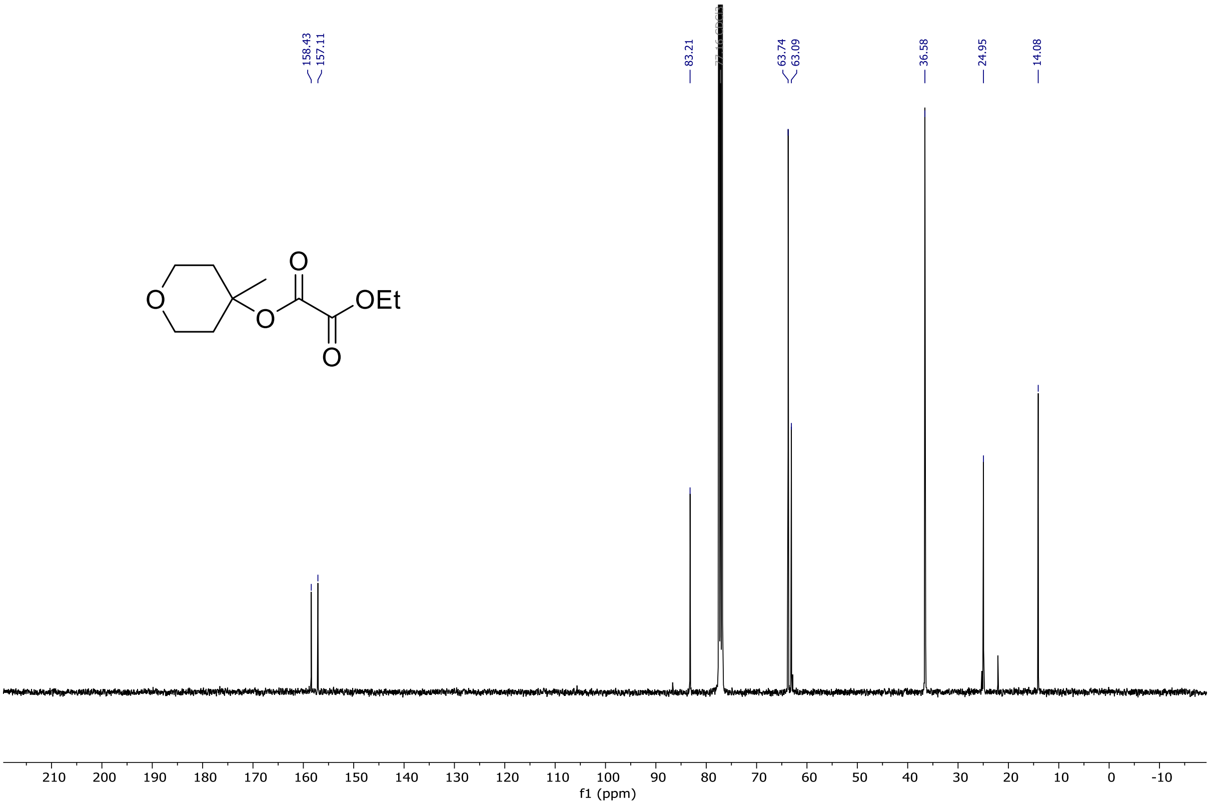

Compound **3f**

^{1}H NMR, DMSO, 400 MHz

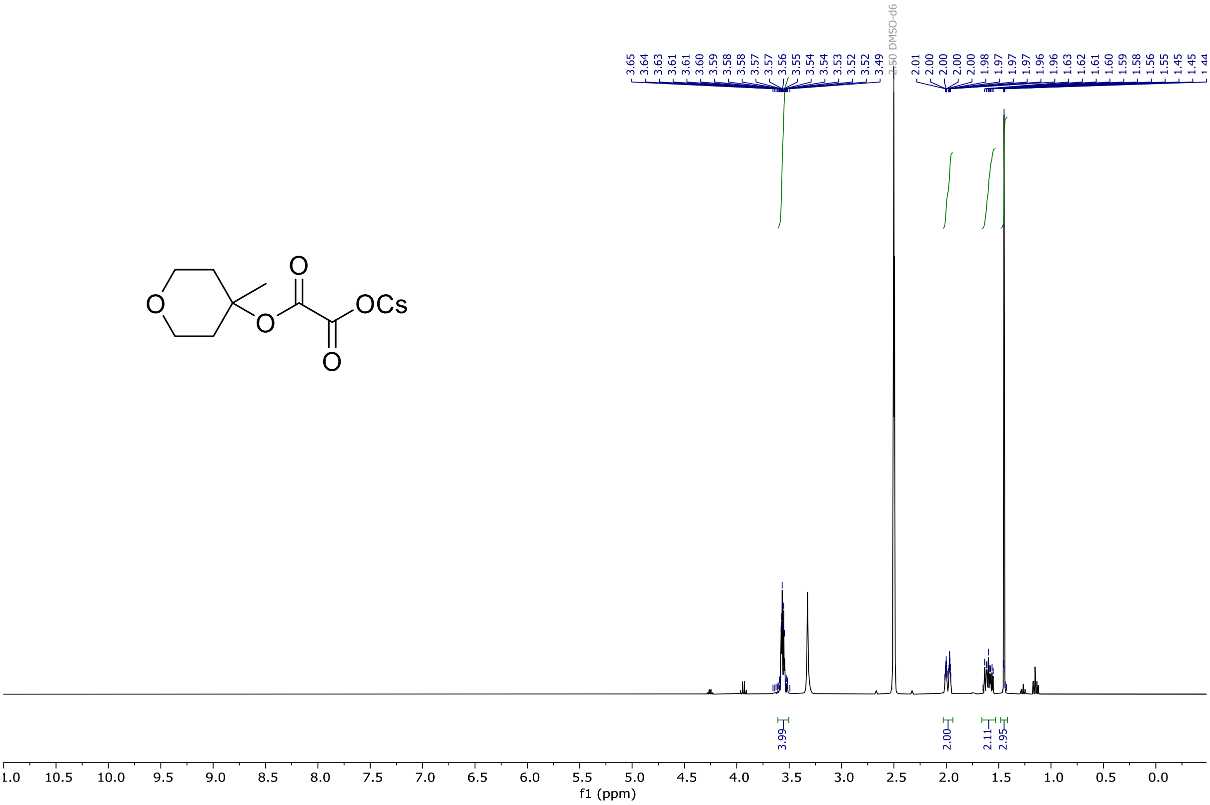

^{13}C NMR, DMSO, 101 MHz

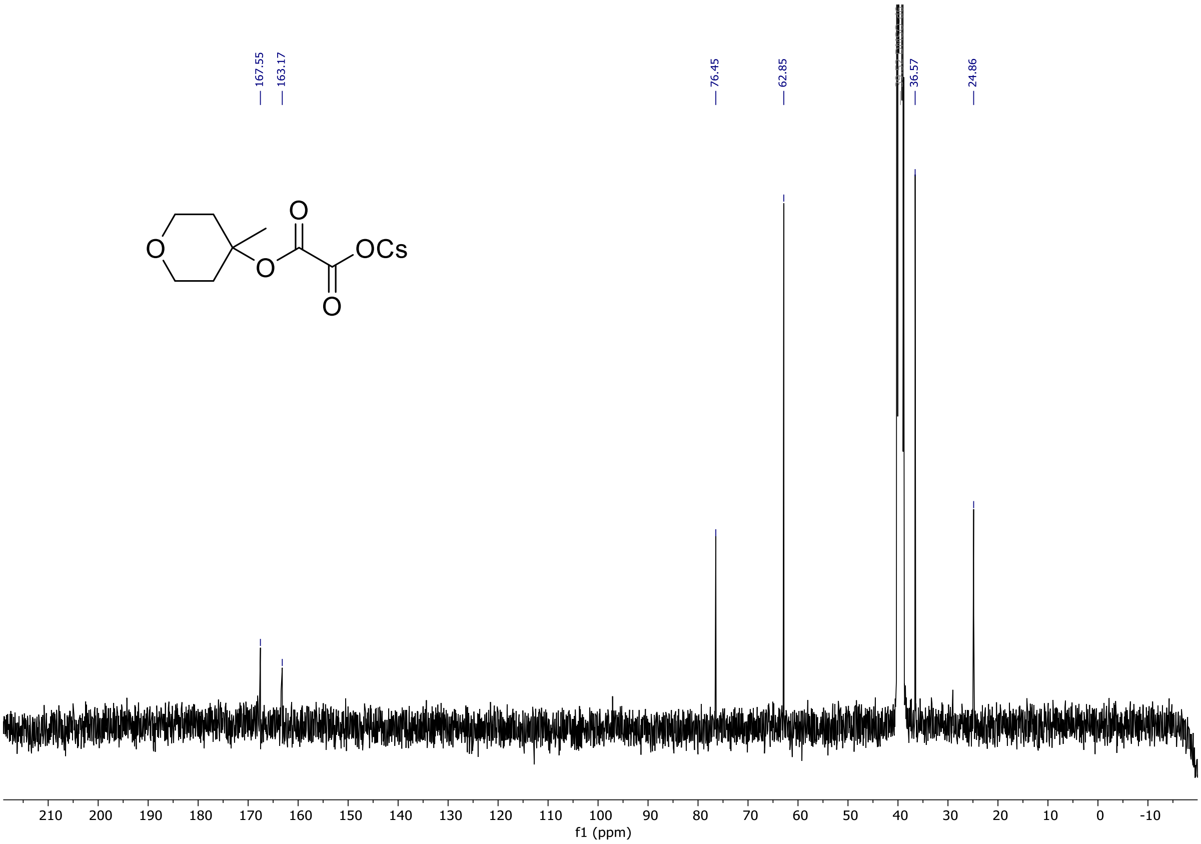

Compound **28i**

^{1}H NMR, $CDCl_3$, 400 MHz

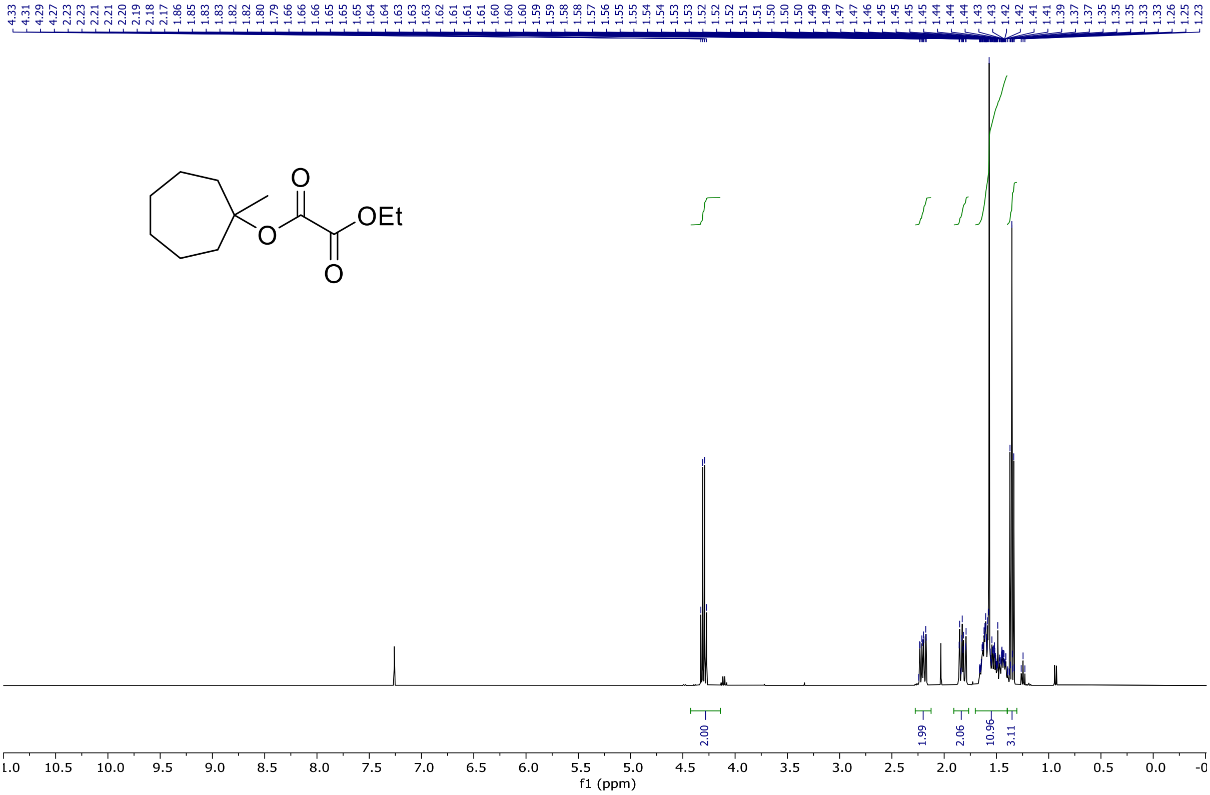

^{13}C NMR, $CDCl_3$, 101 MHz

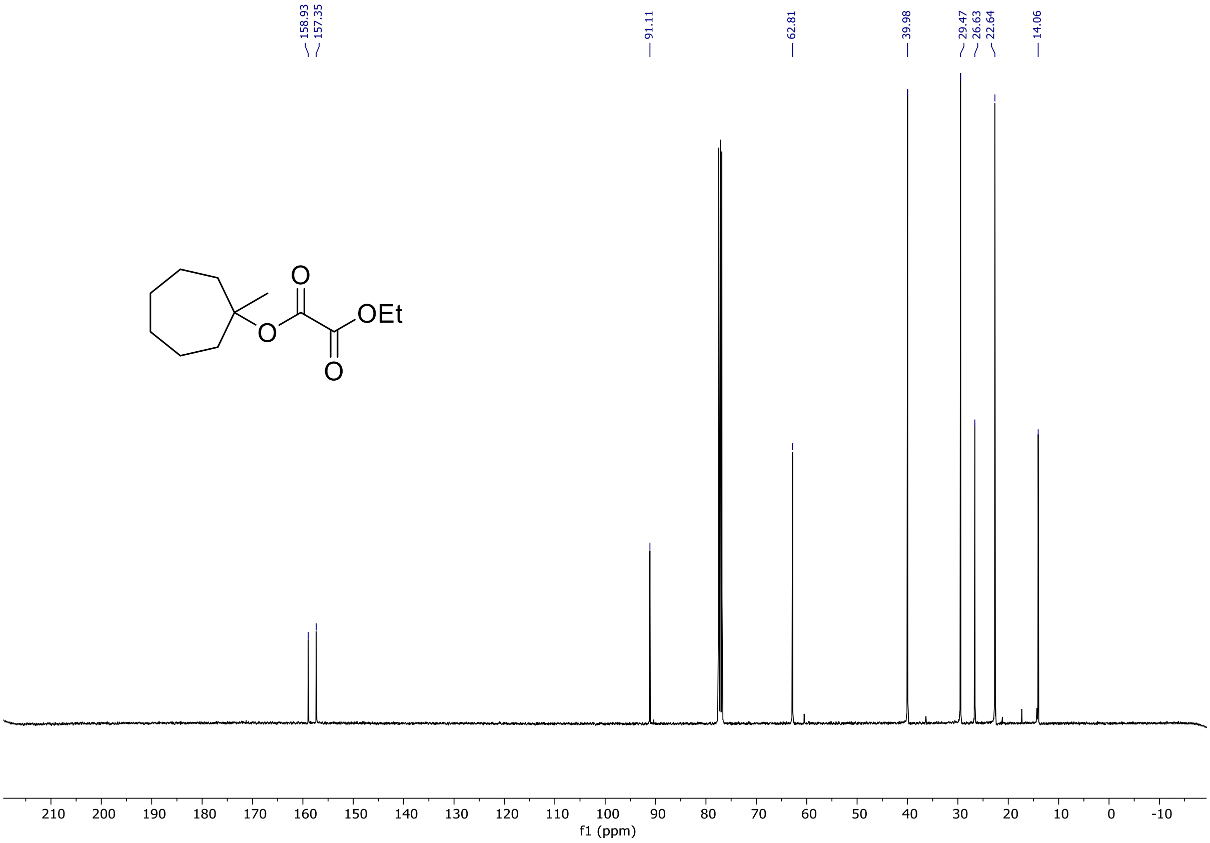

Compound **3i**

^{1}H NMR, DMSO, 400 MHz

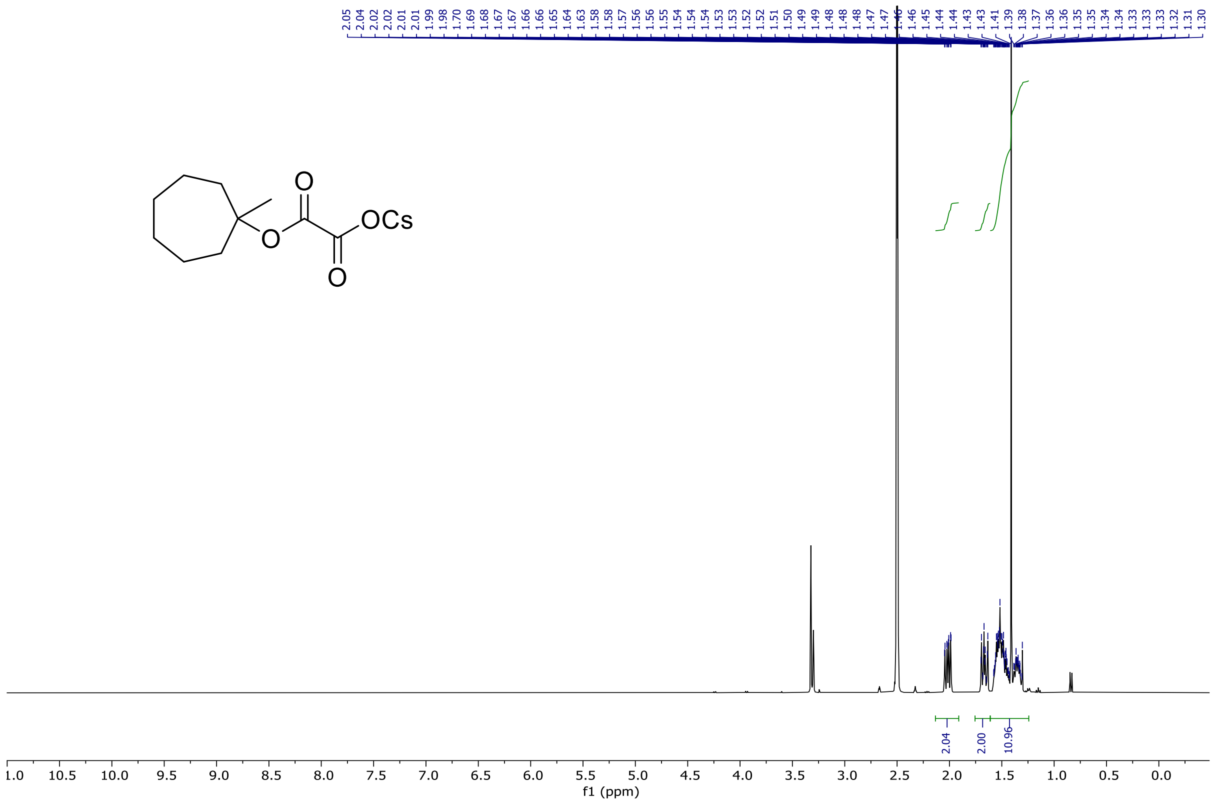

^{13}C NMR, DMSO, 101 MHz

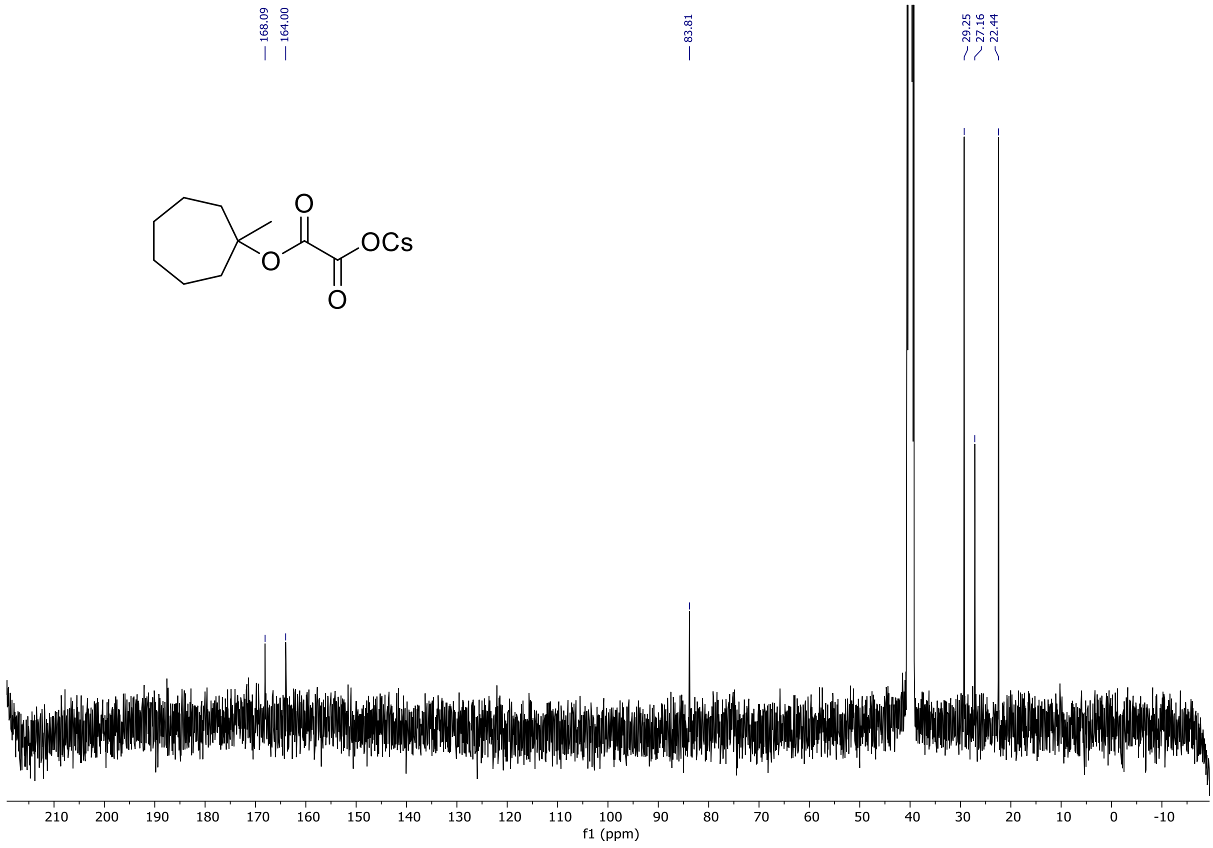

Compound **28j**

^{1}H NMR, $CDCl_3$, 400 MHz

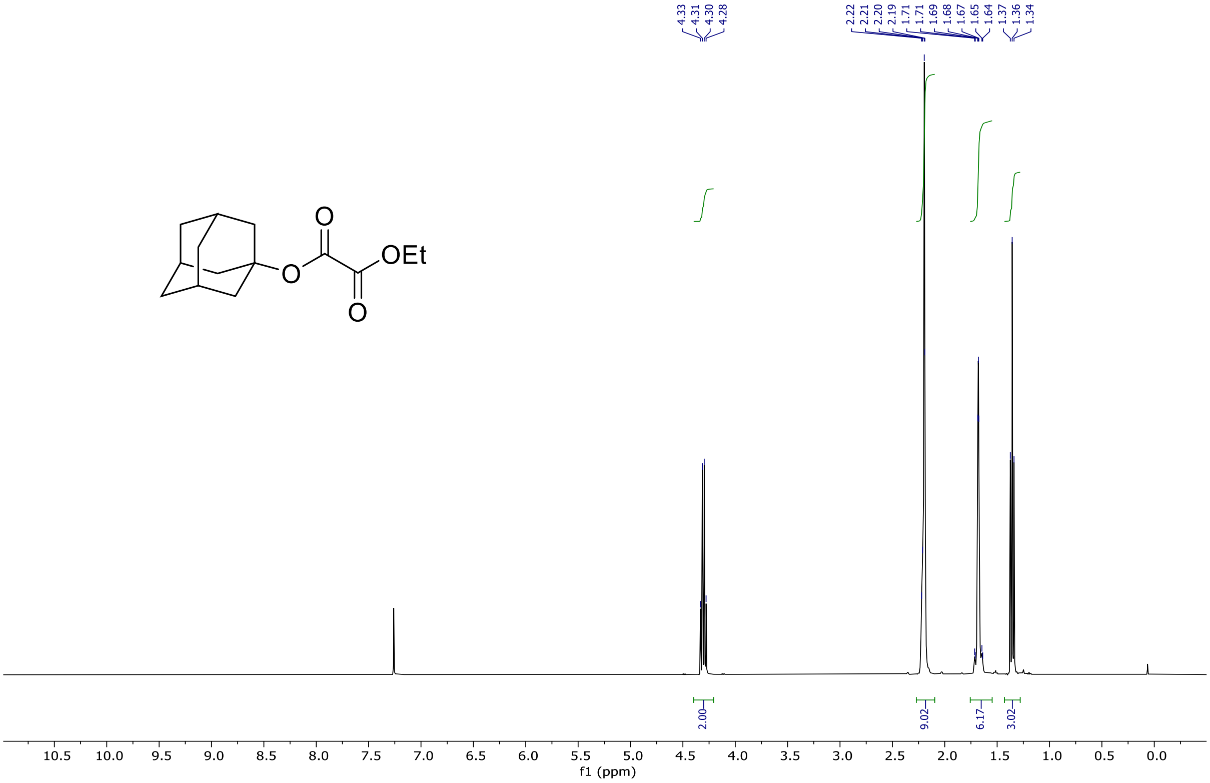

^{13}C NMR, $CDCl_3$, 101 MHz

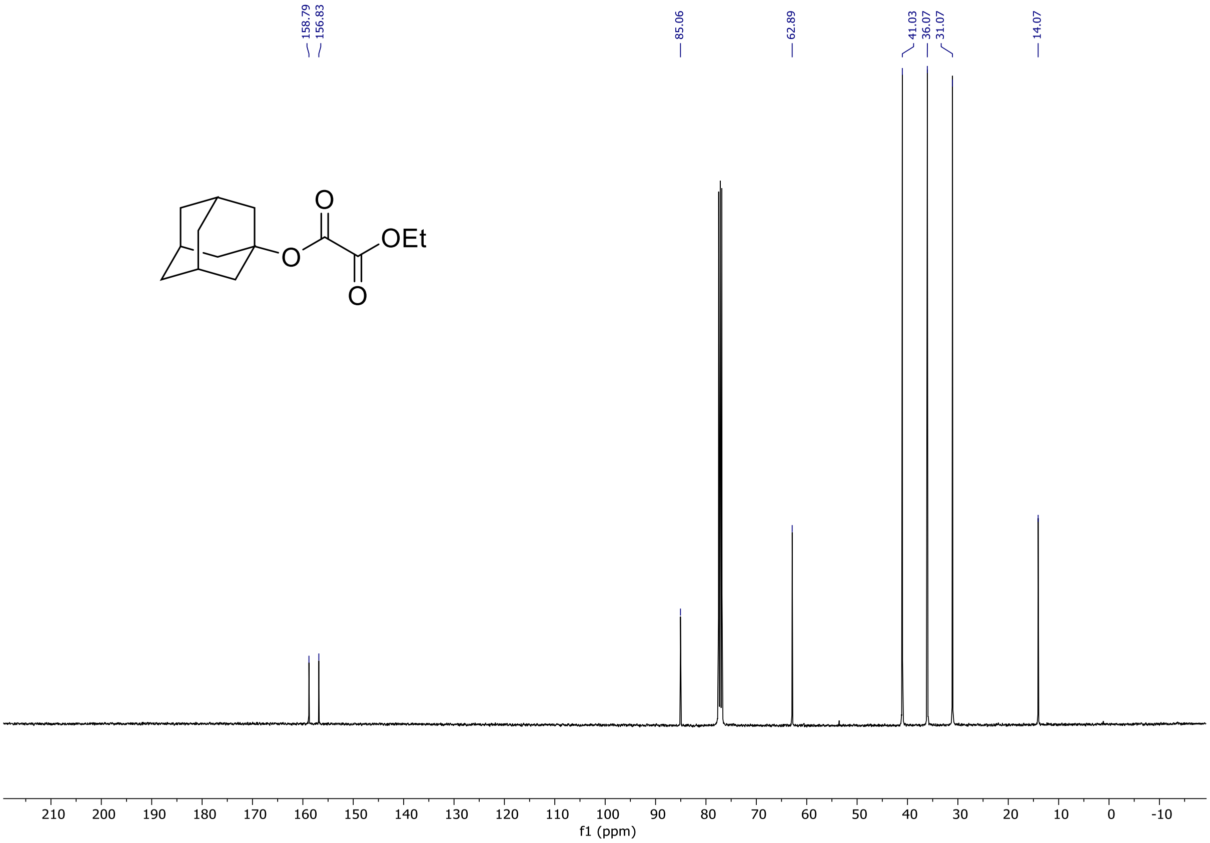

Compound **3j** (with 25% **28j**)

^{1}H NMR, DMSO, 400 MHz

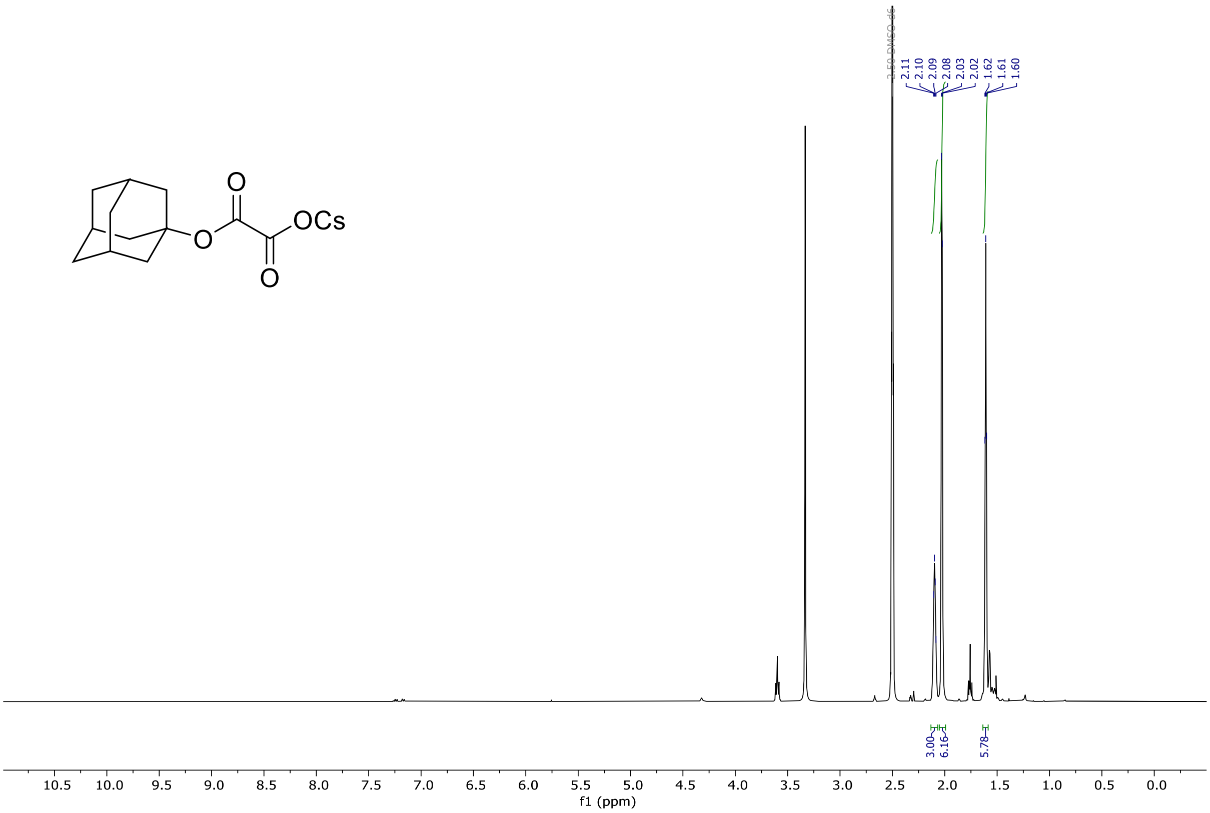

^{13}C NMR, DMSO, 101 MHz

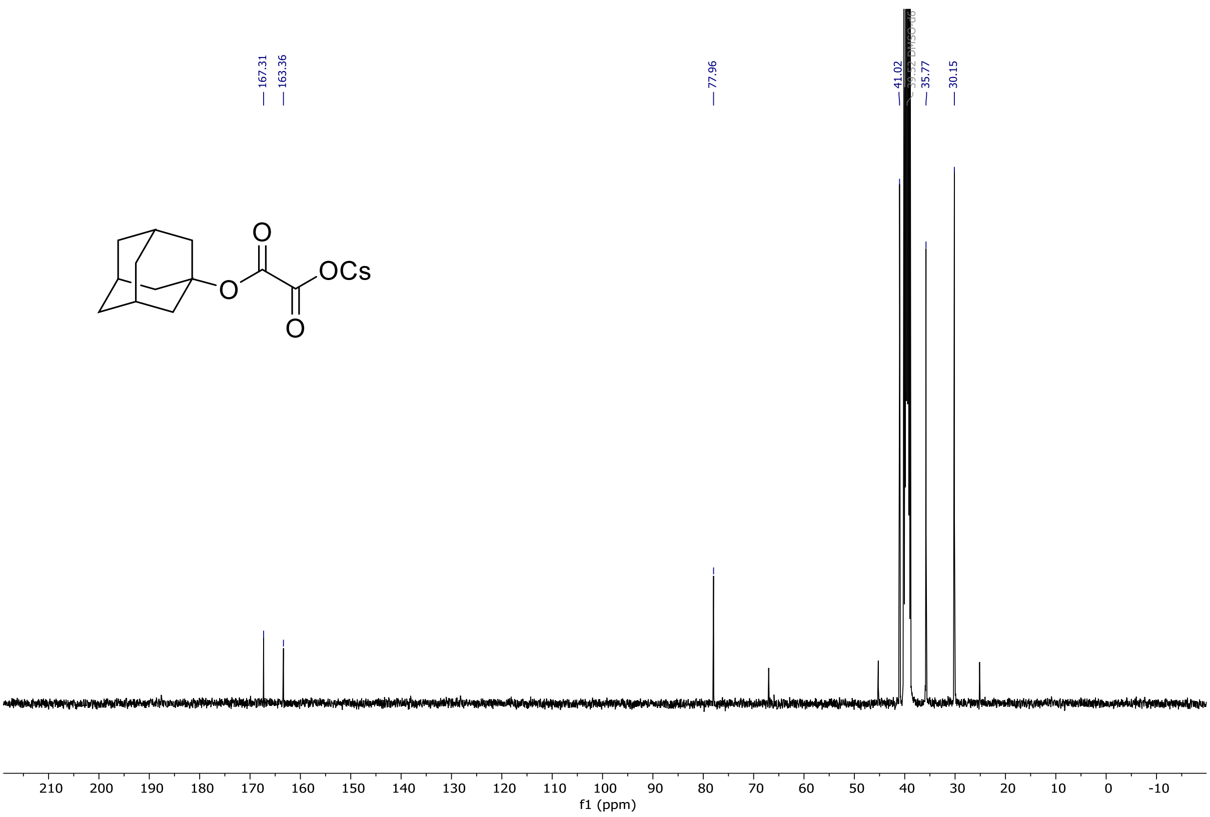

Compound **28k**

^{1}H NMR, $CDCl_3$, 400 MHz

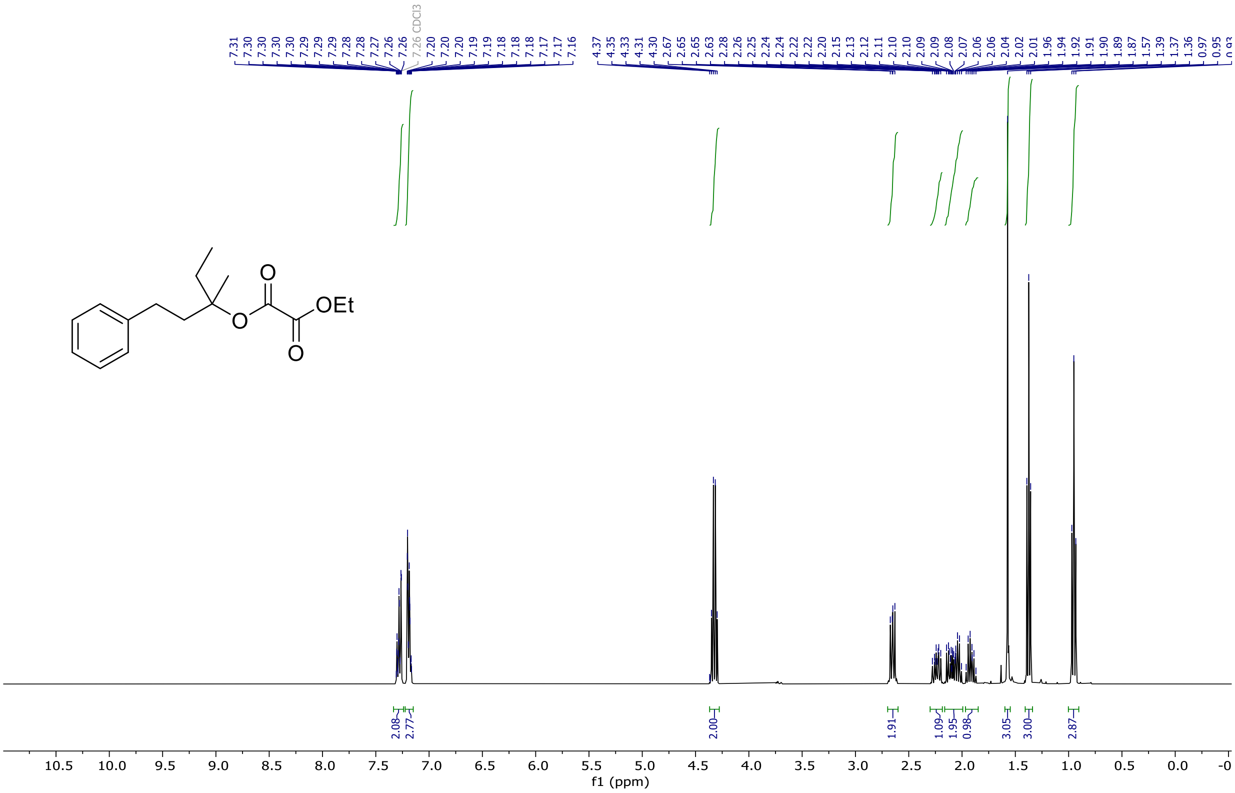

^{13}C NMR, $CDCl_3$, 101 MHz

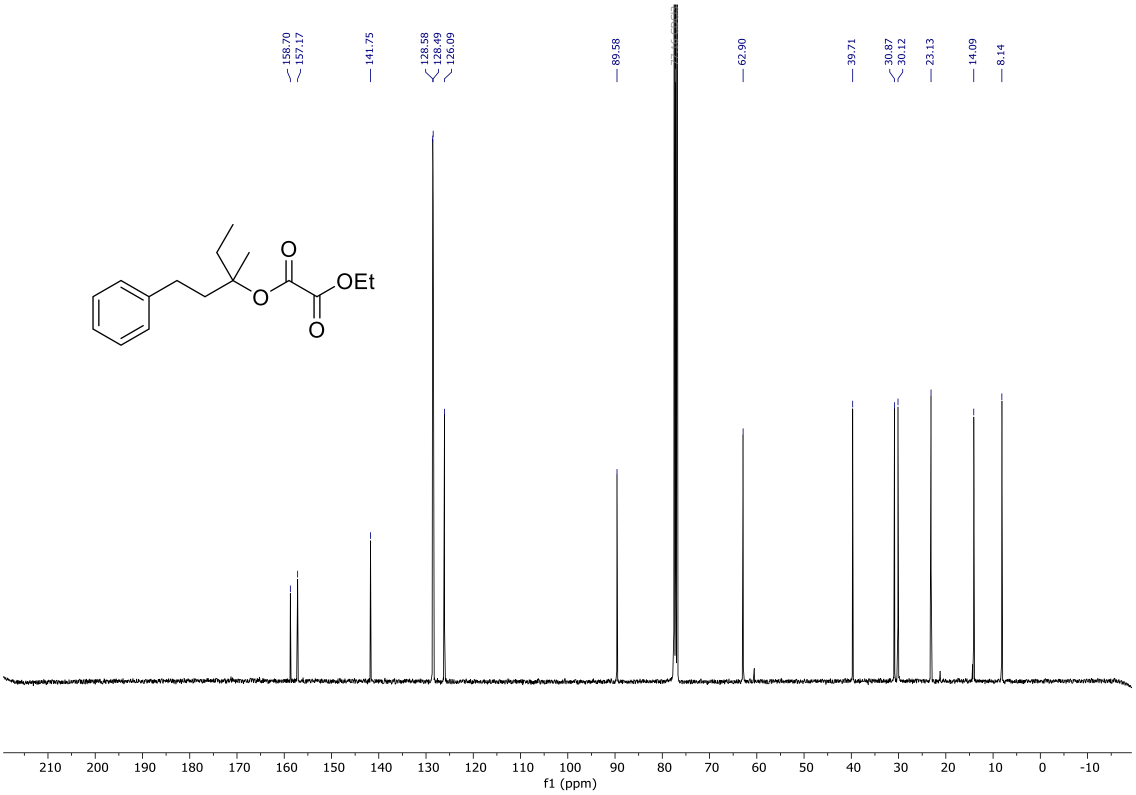

Compound **3k**

^{1}H NMR, DMSO, 400 MHz

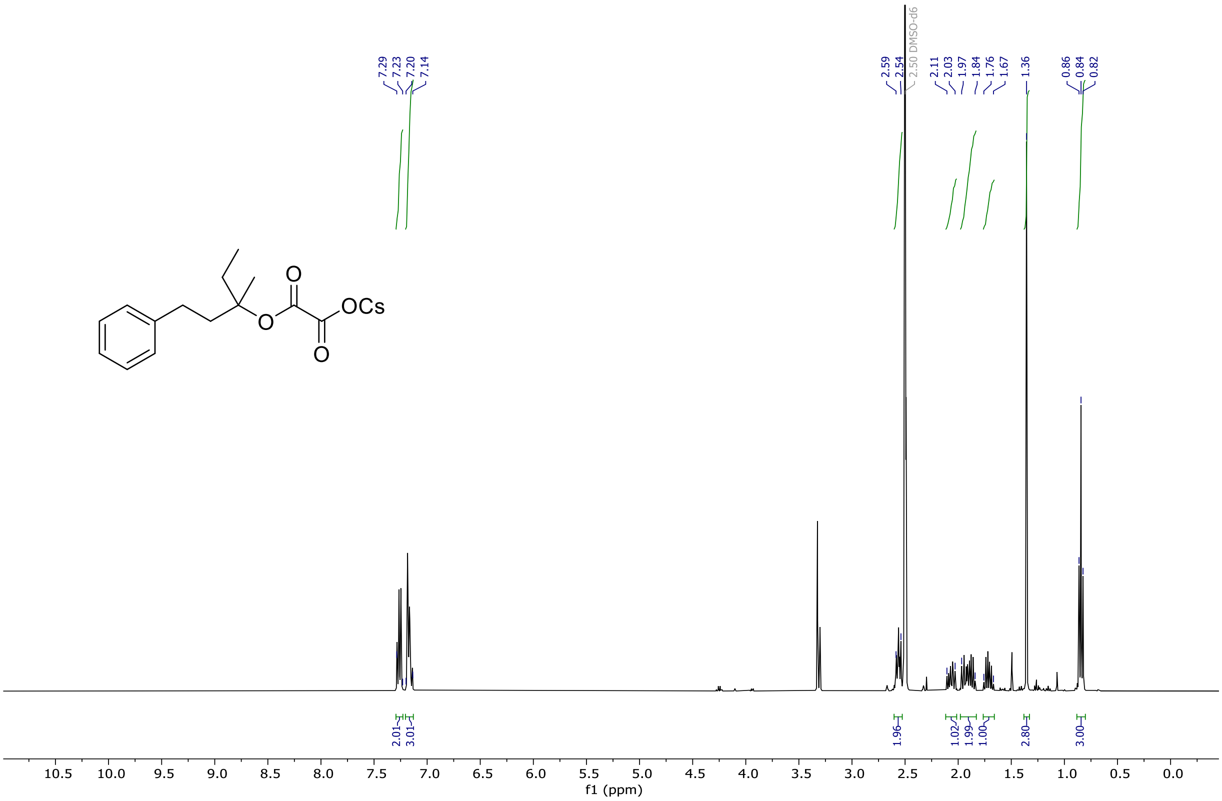

^{13}C NMR, DMSO, 101 MHz

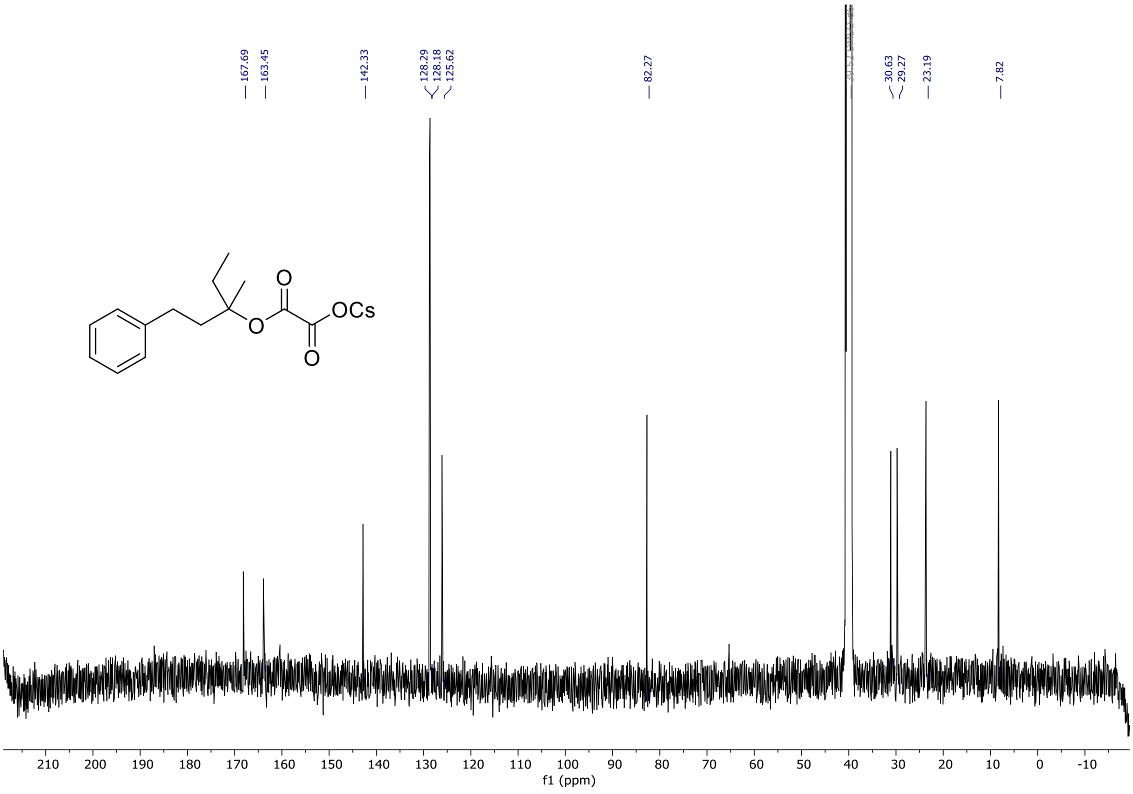

Compound **28l**

^{1}H NMR, $CDCl_3$, 400 MHz

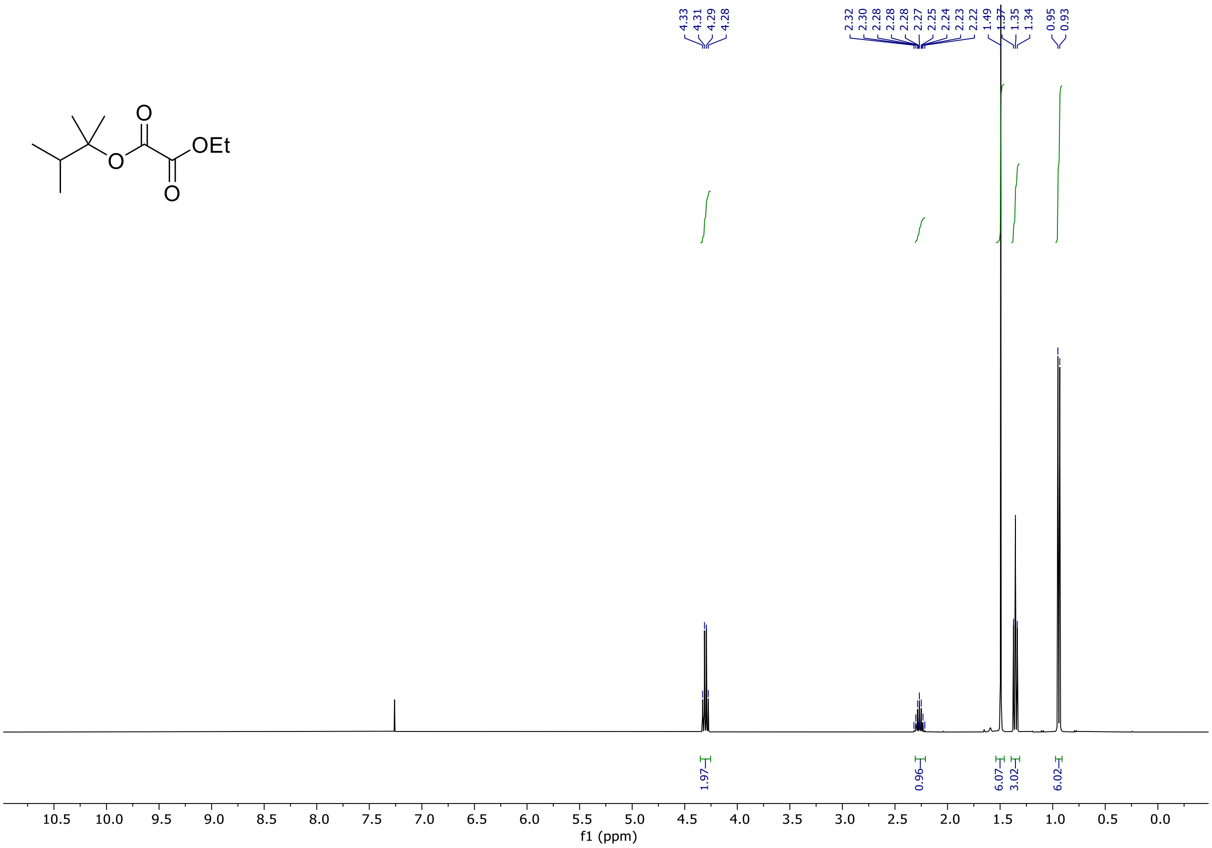

^{13}C NMR, $CDCl_3$, 101 MHz

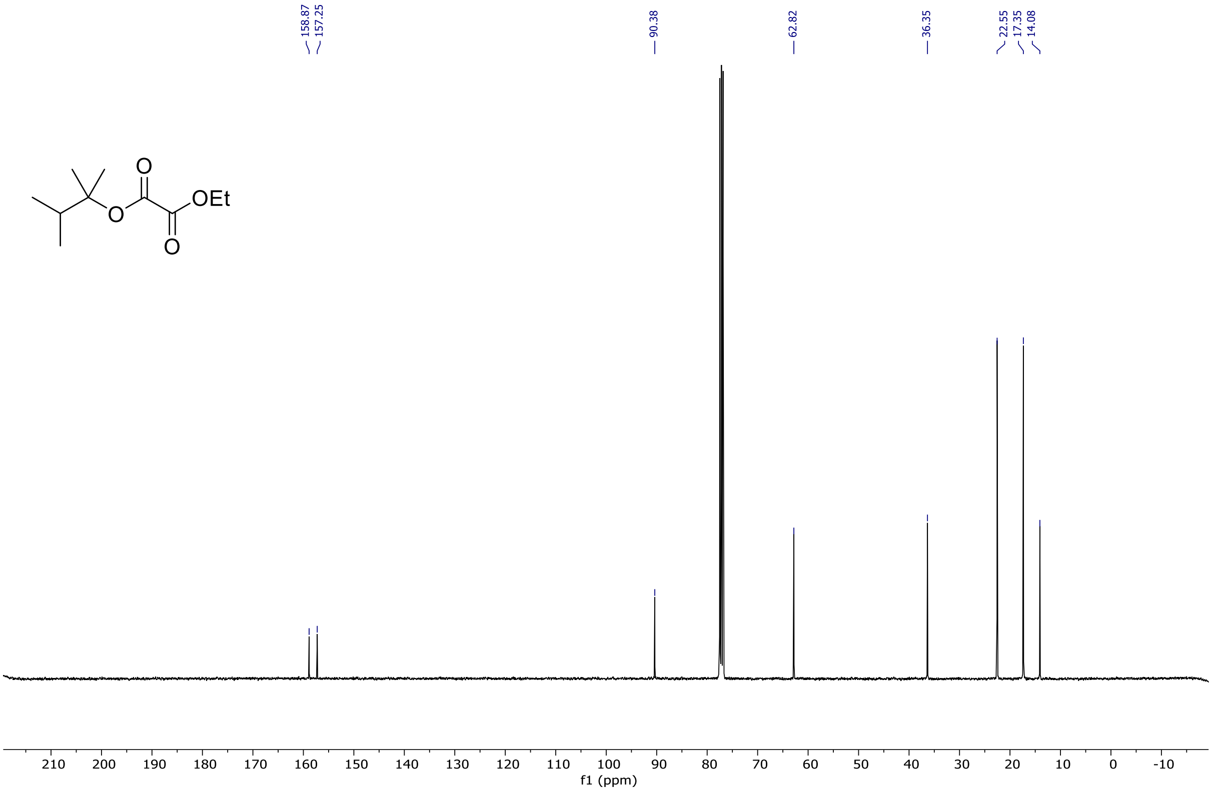

Compound **3l**

^{1}H NMR, DMSO, 400 MHz

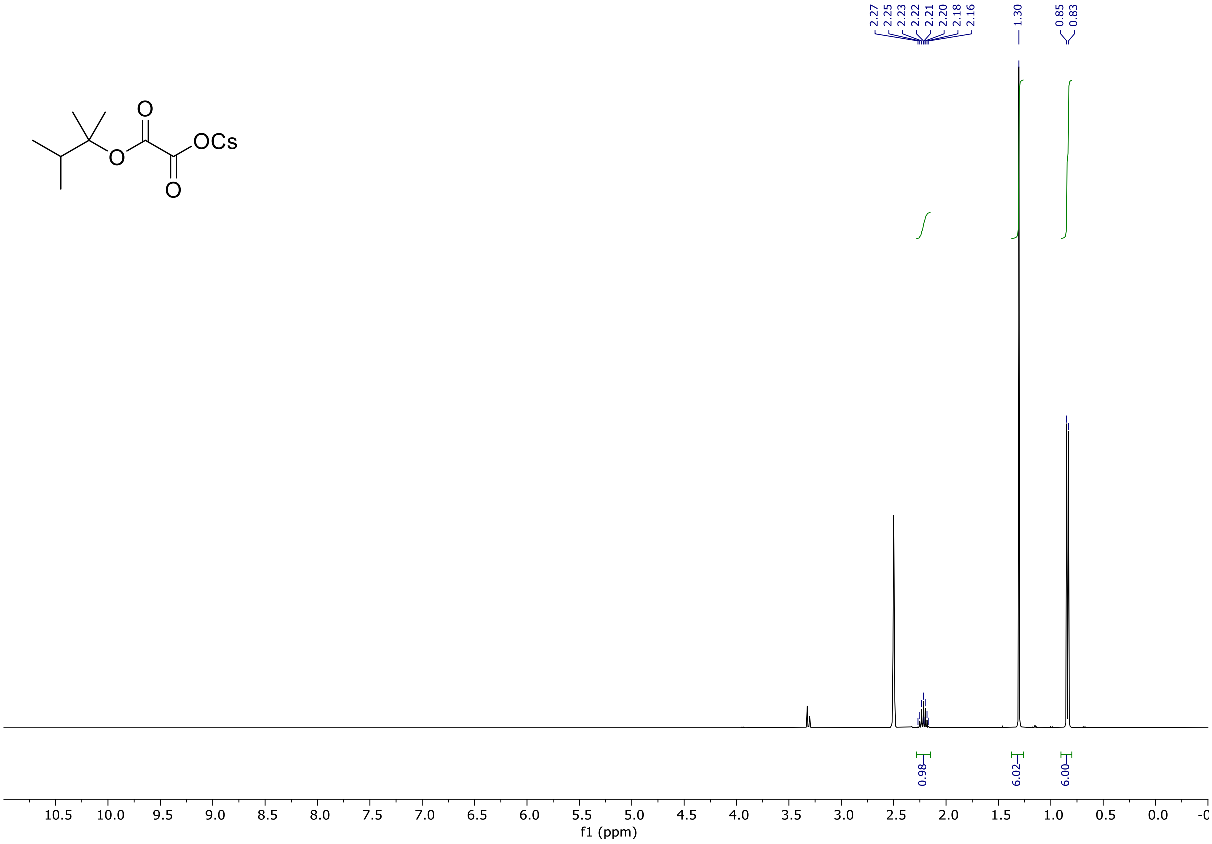

^{13}C NMR, DMSO, 101 MHz

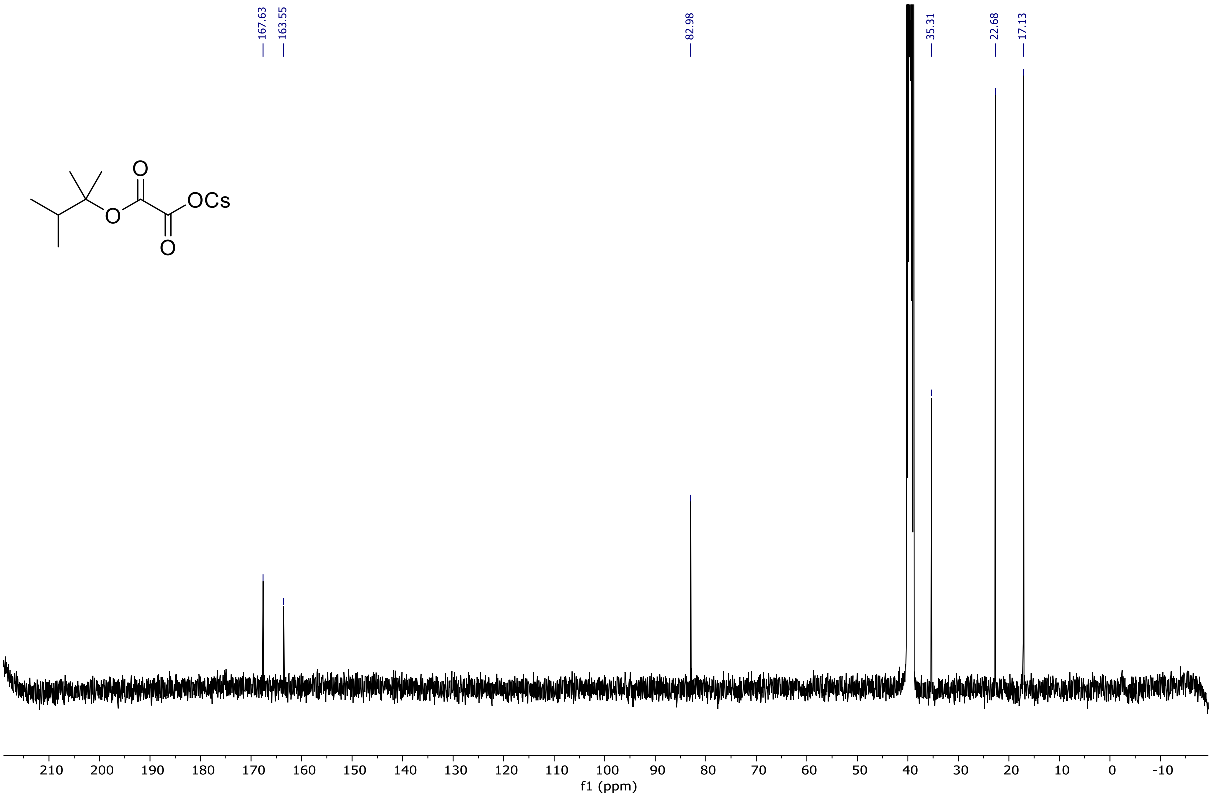

Compound **28m**

^{1}H NMR, $CDCl_3$, 400 MHz

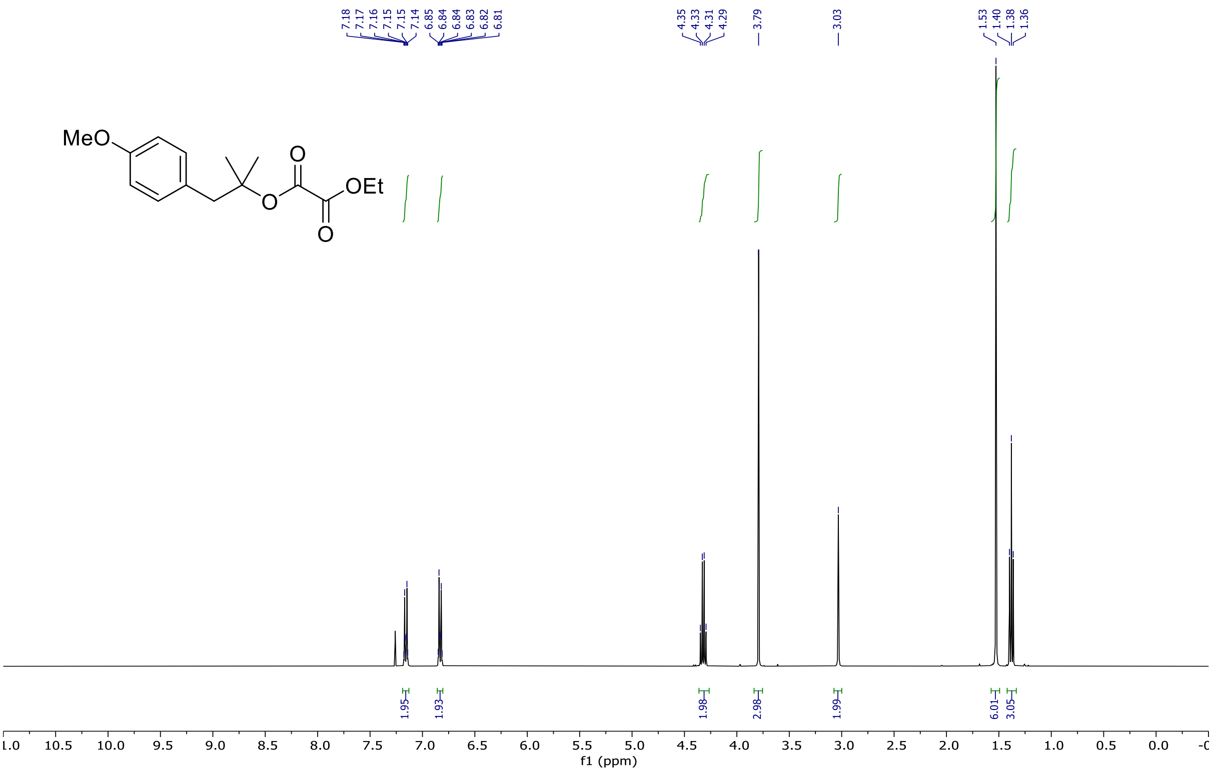

^{13}C NMR, $CDCl_3$, 101 MHz

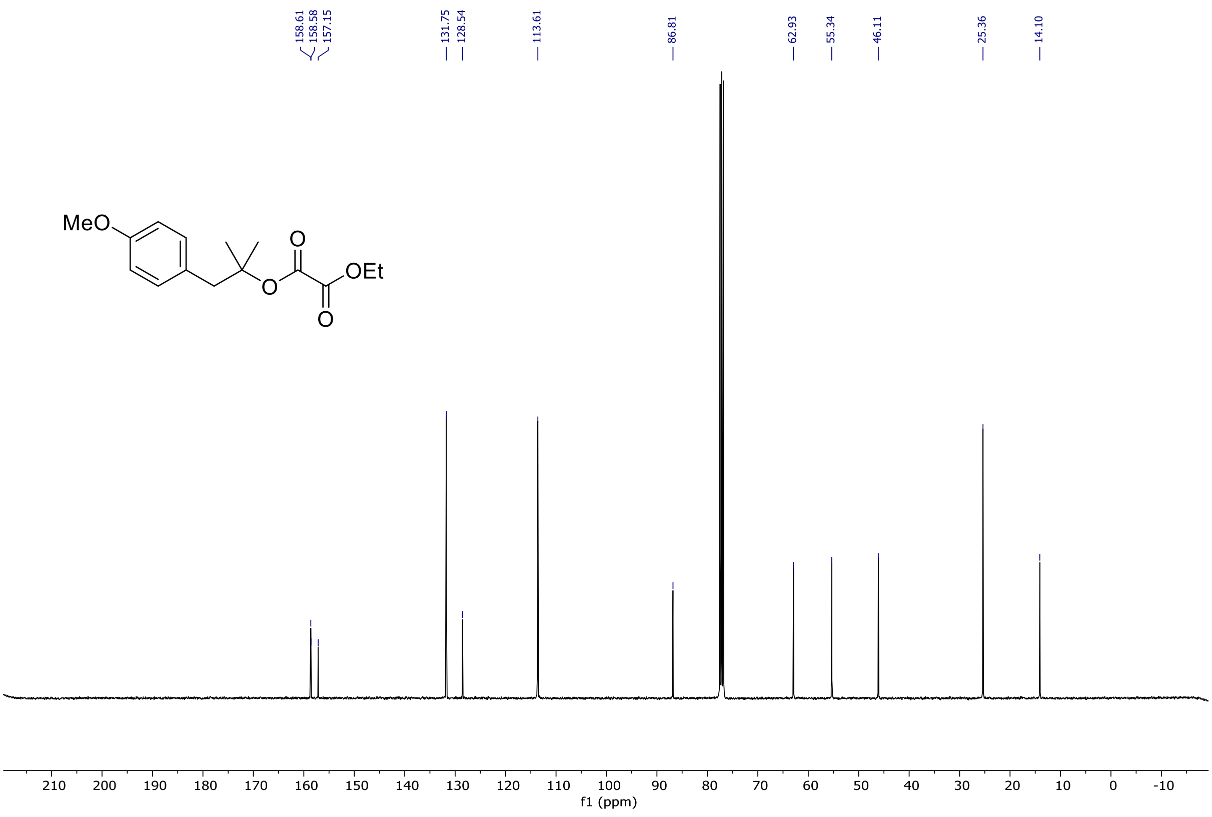

Compound **3m**

^1H NMR, DMSO, 400 MHz

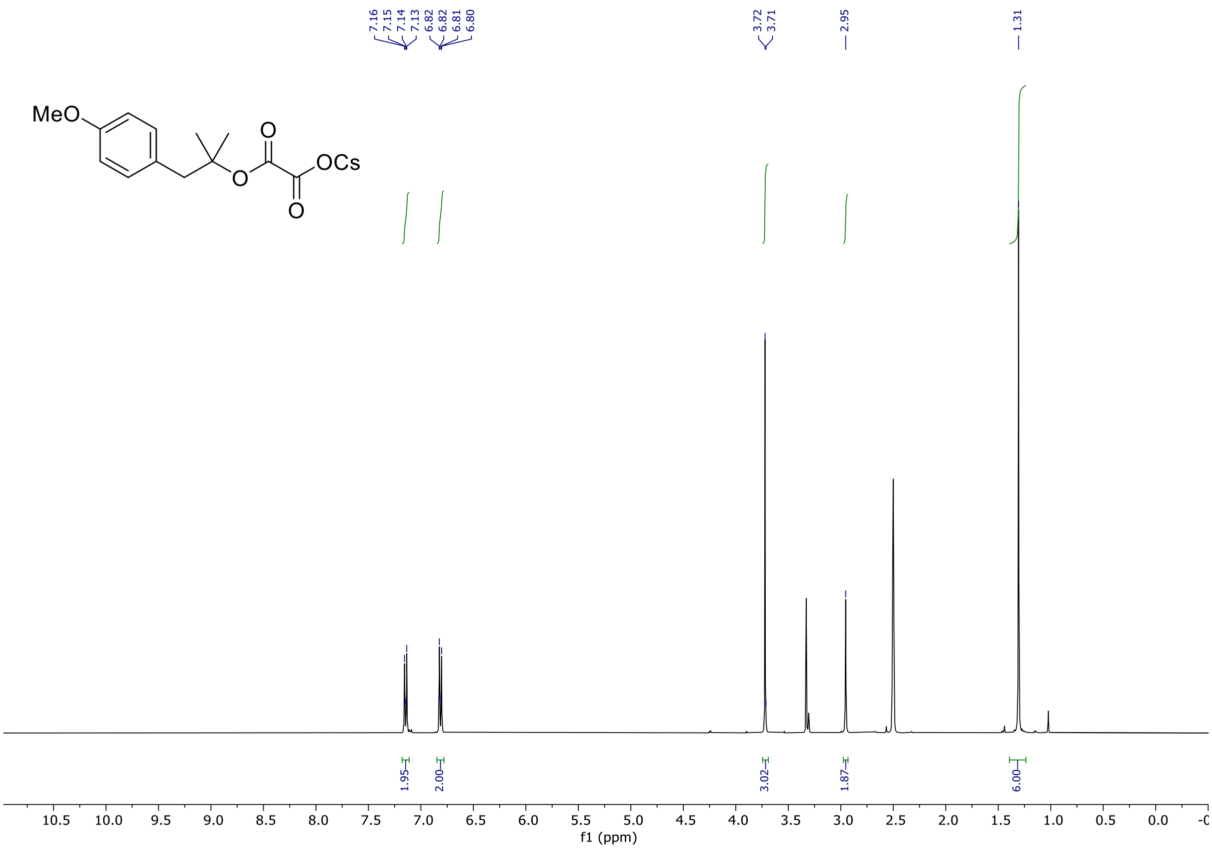

^{13}C NMR, DMSO, 101 MHz

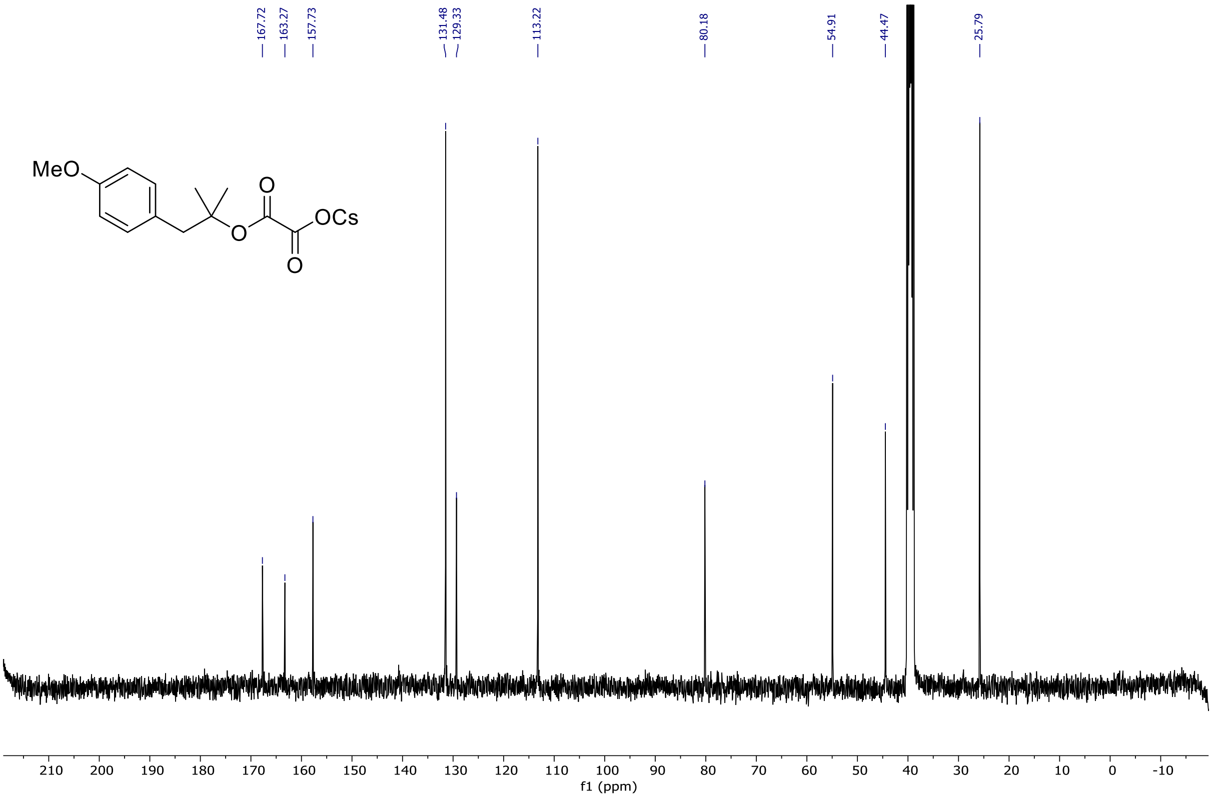

Compound **28n**

^{1}H NMR, $CDCl_3$, 400 MHz

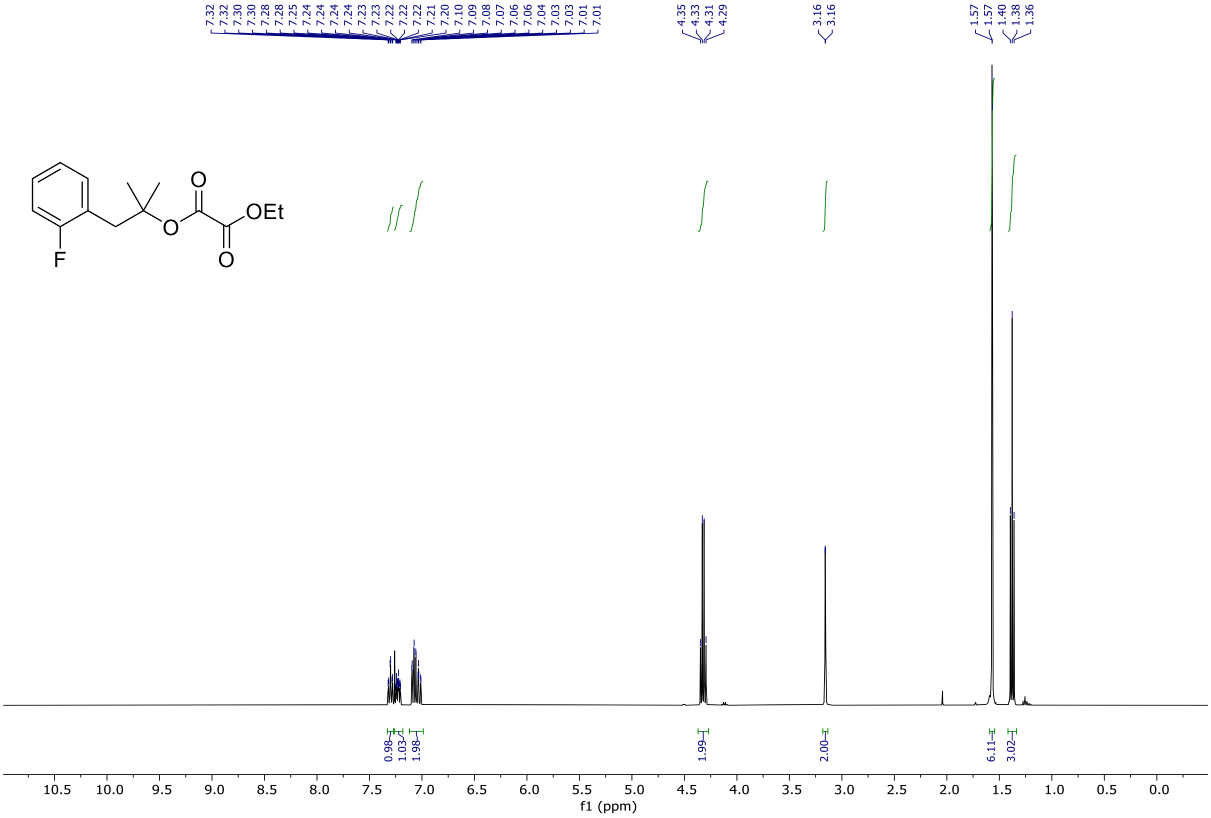

^{13}C NMR, $CDCl_3$, 101 MHz

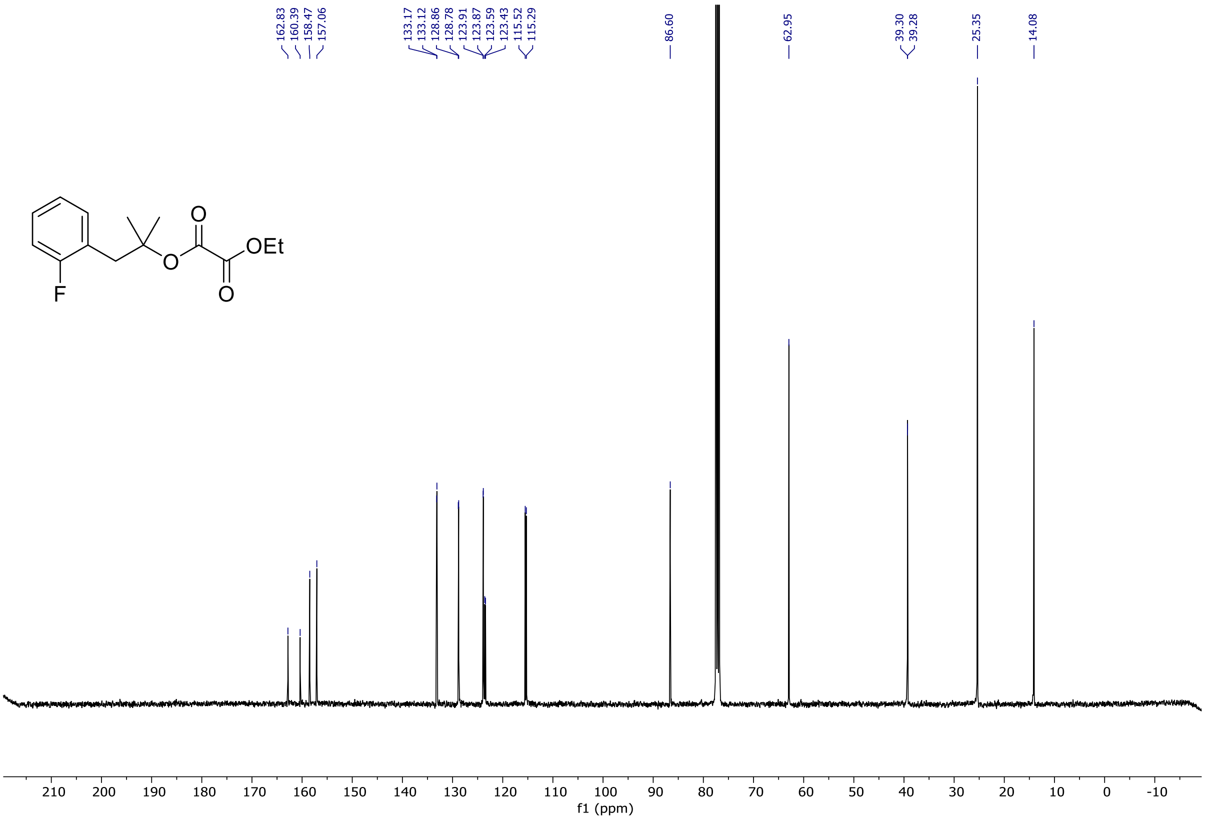

^{19}F NMR, $CDCl_3$, 376 MHz

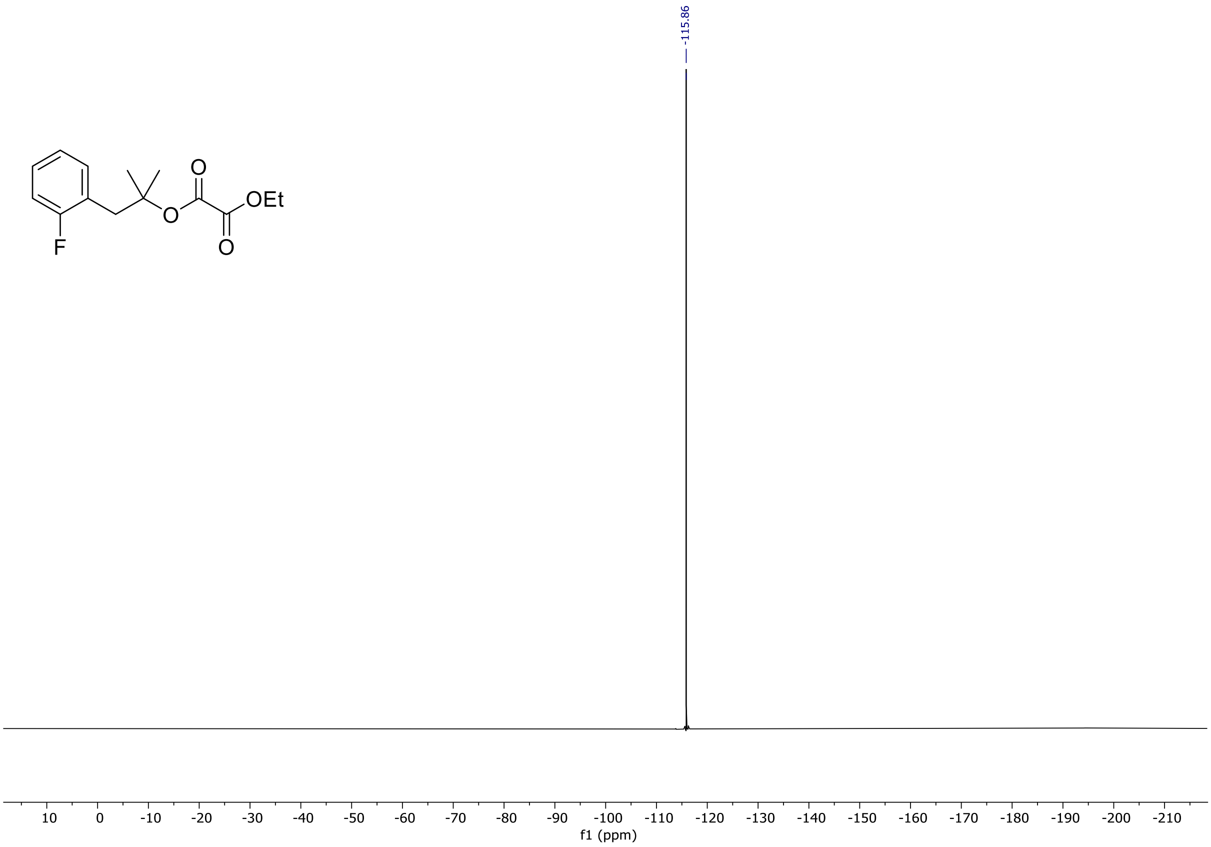

Compound **3n**

^{1}H NMR, DMSO, 400 MHz

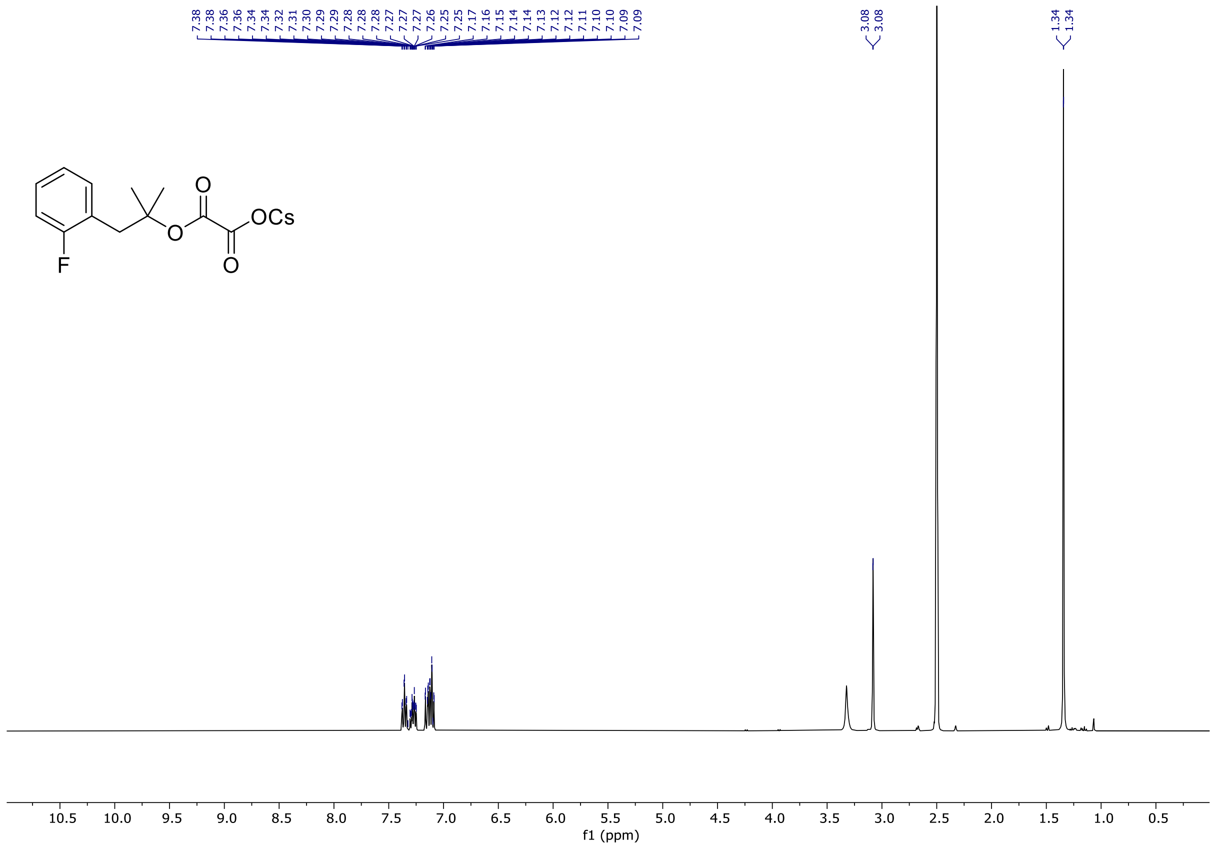

^{13}C NMR, DMSO, 101 MHz

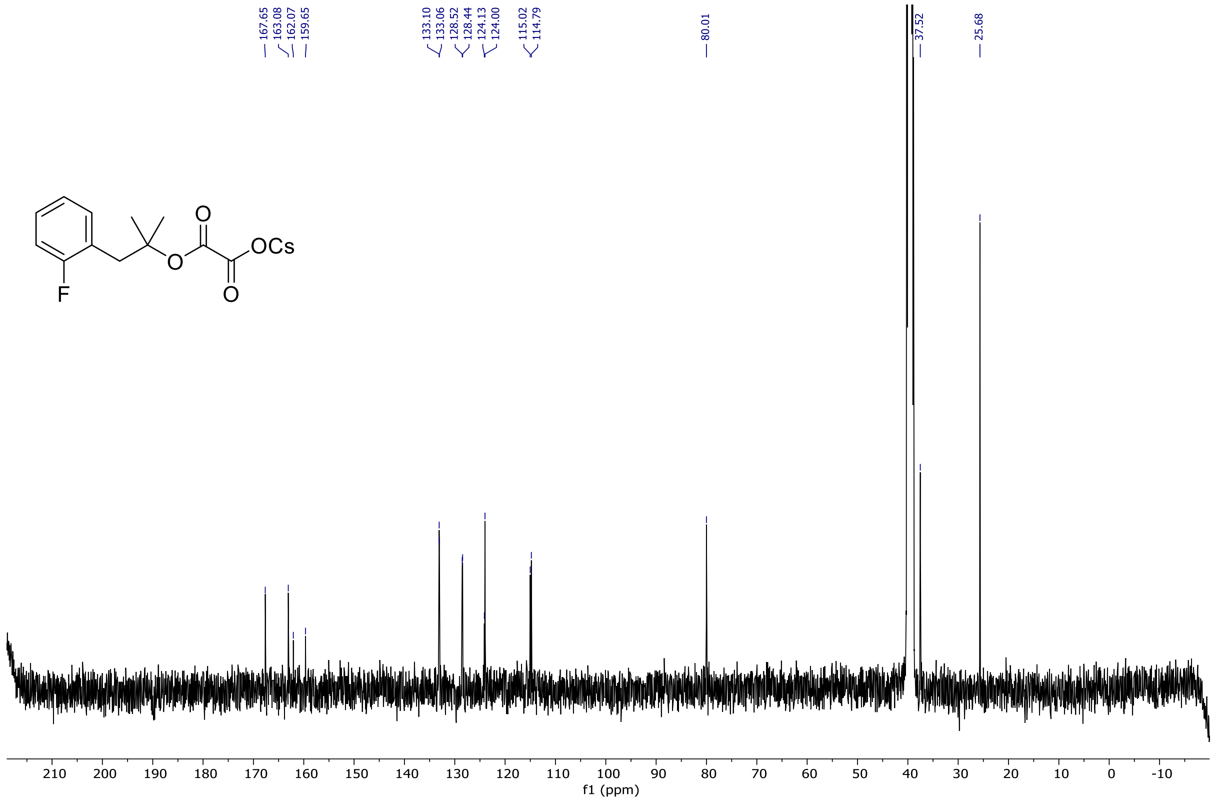

^{19}F NMR, DMSO, 376 MHz

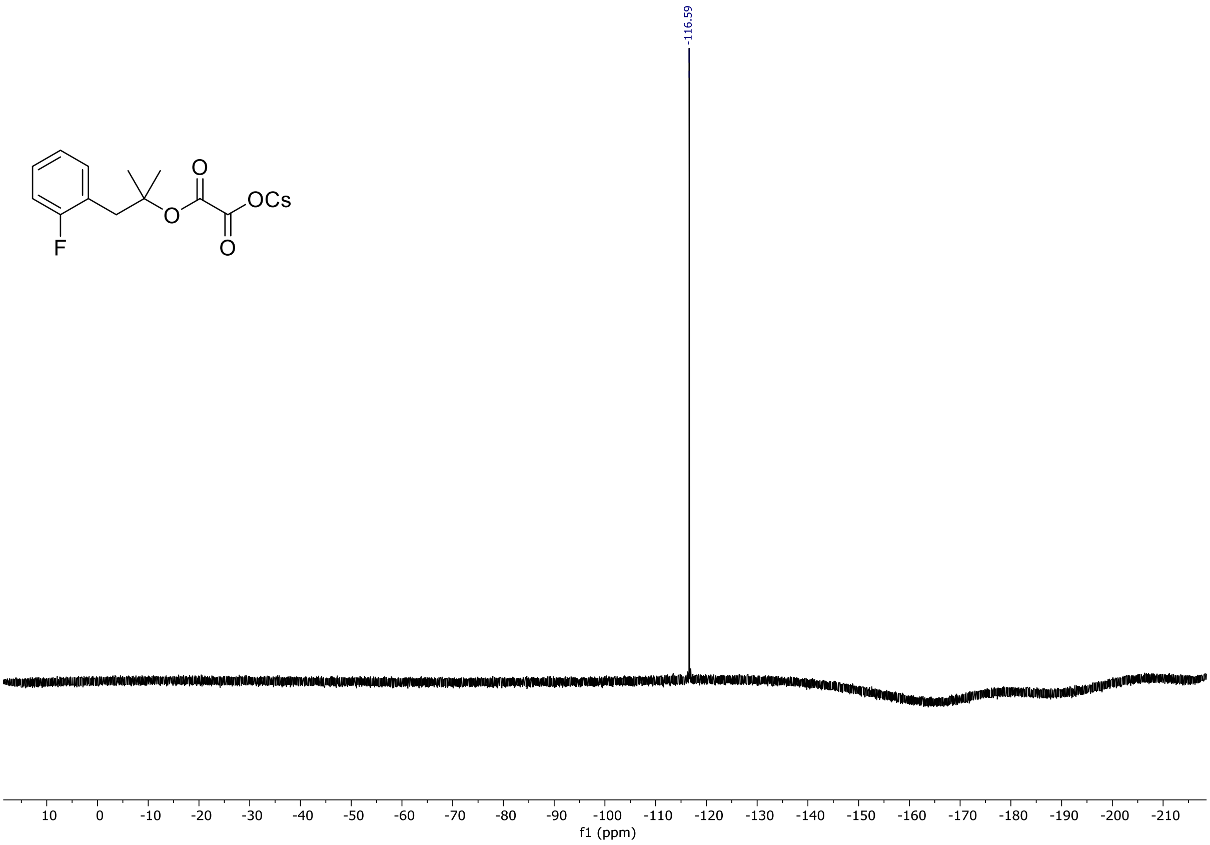

Compound **28o**

^{1}H NMR, $CDCl_3$, 400 MHz

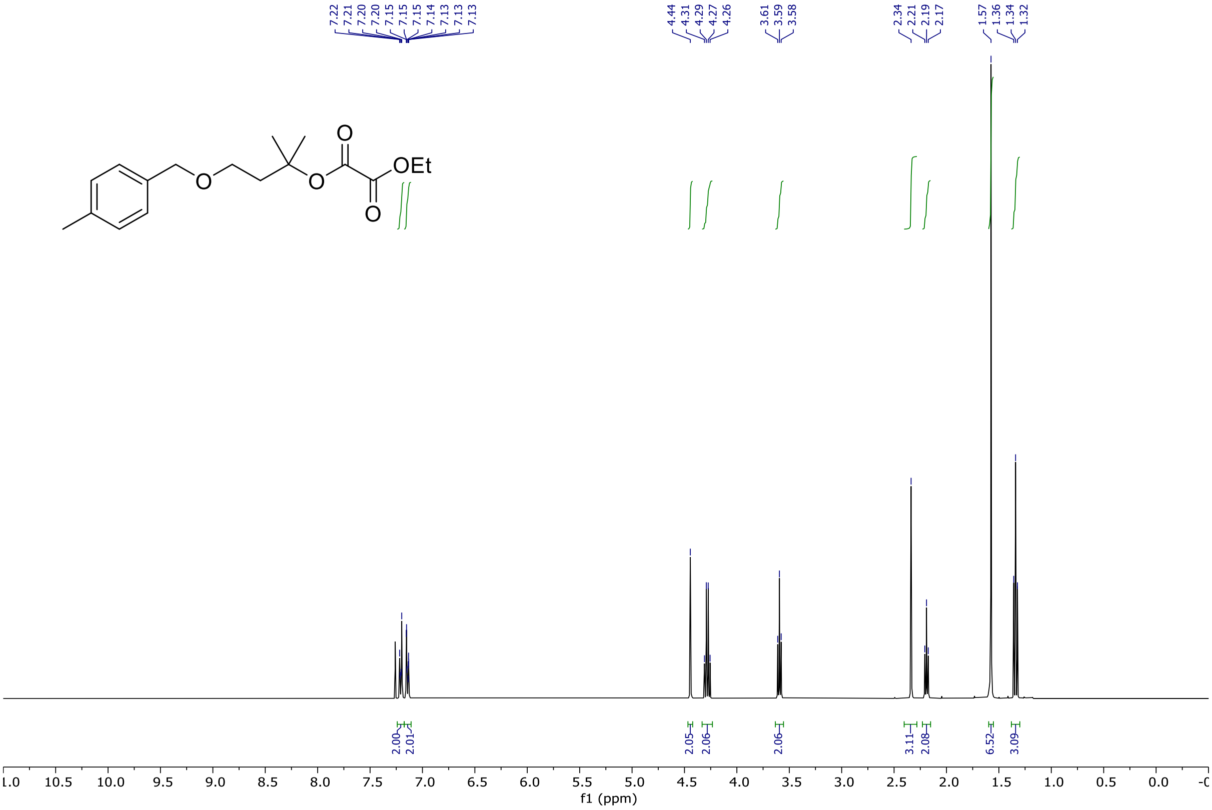

^{13}C NMR, $CDCl_3$, 101 MHz

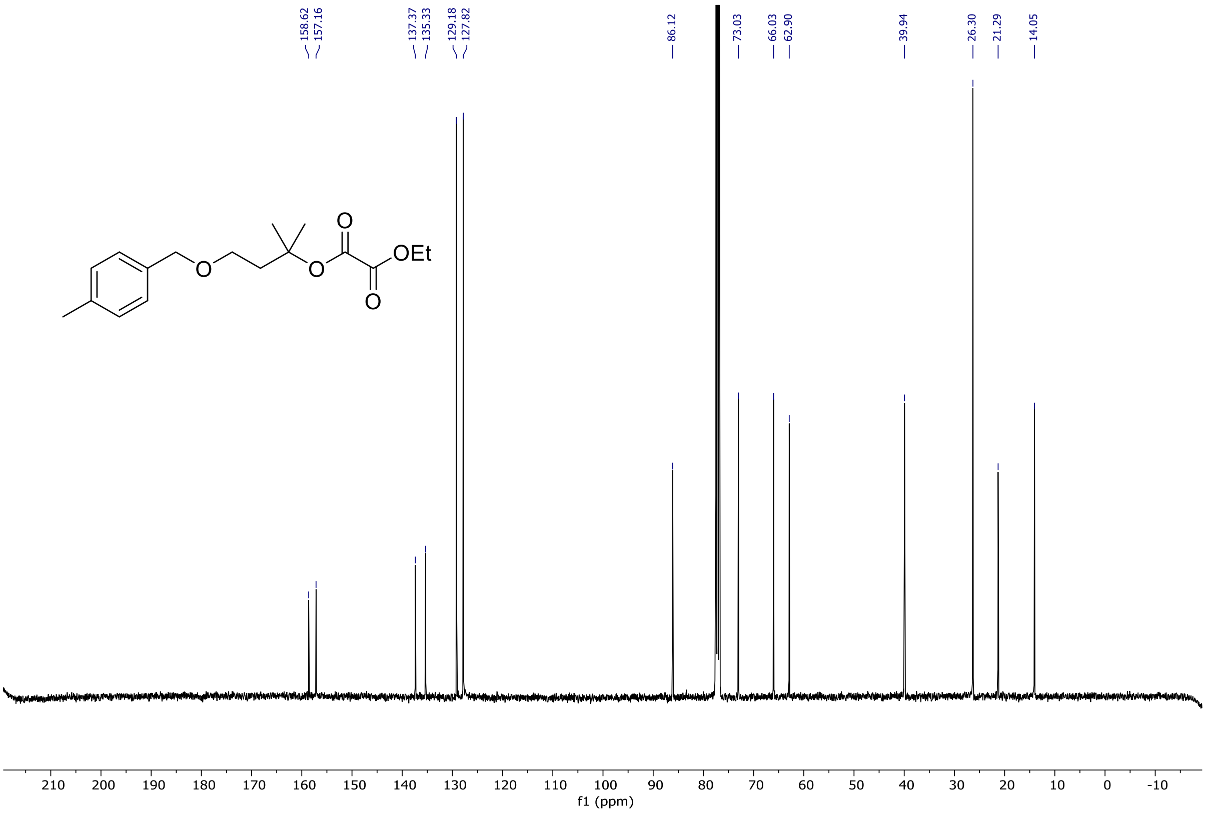

Compound **3o**

^{1}H NMR, DMSO, 400 MHz

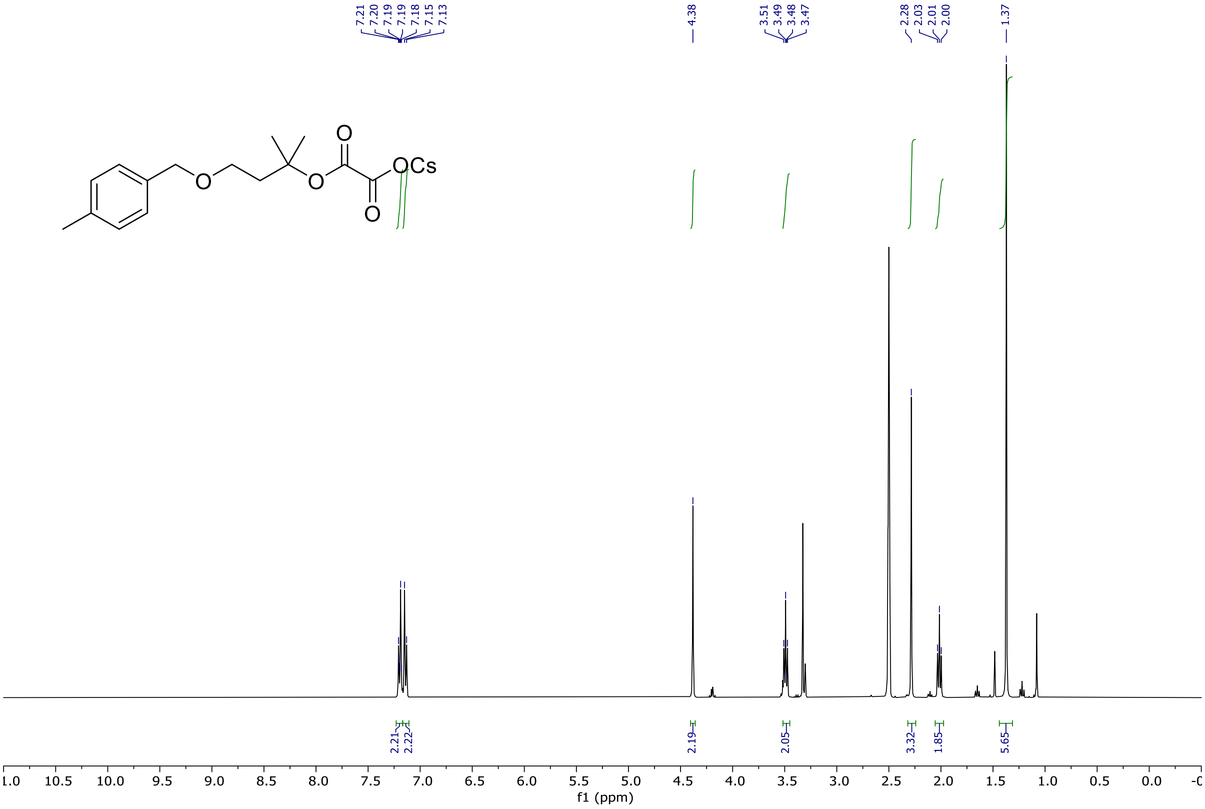

^{13}C NMR, DMSO, 101 MHz

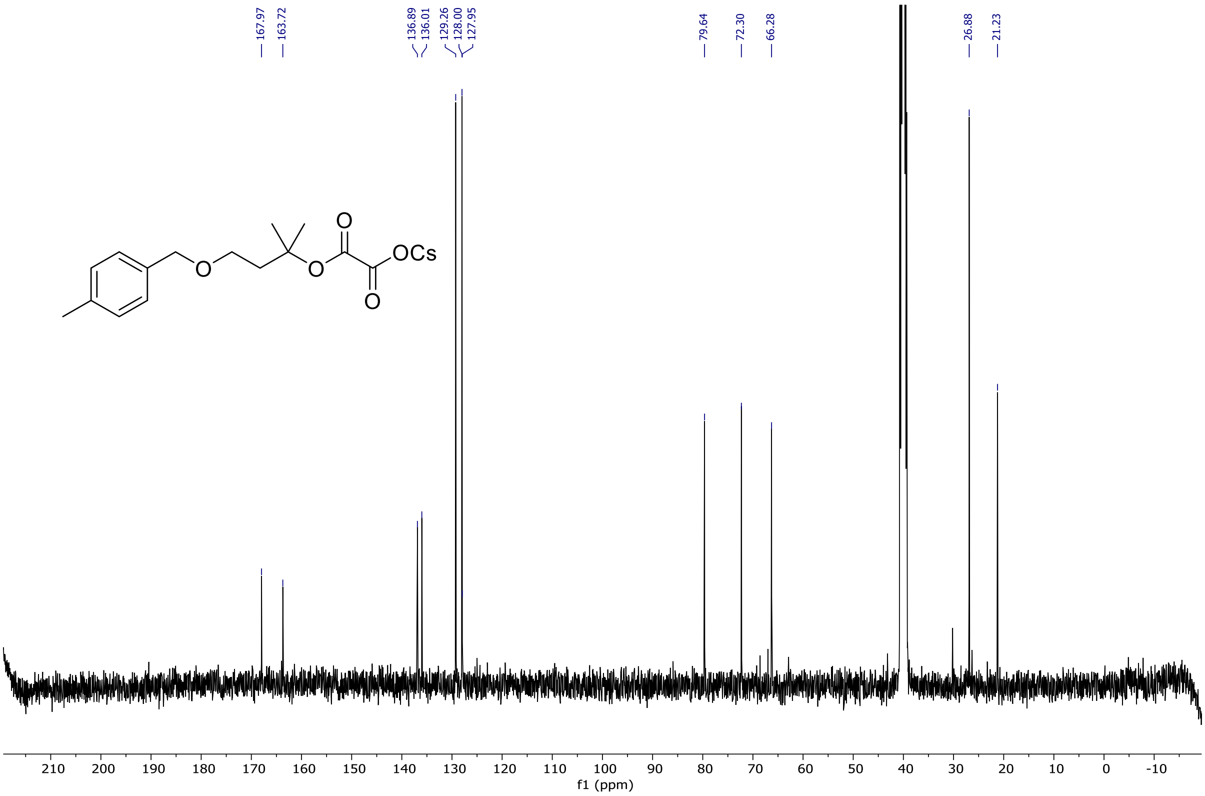

Compound **28p**

^{1}H NMR, $CDCl_3$, 400 MHz

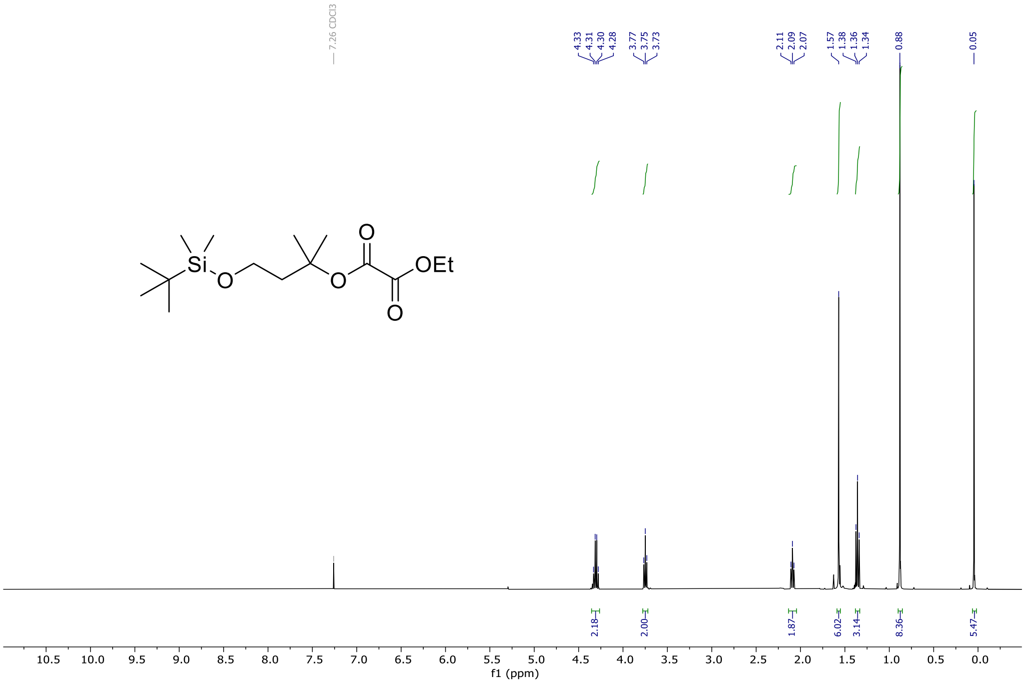

^{13}C NMR, $CDCl_3$, 101 MHz

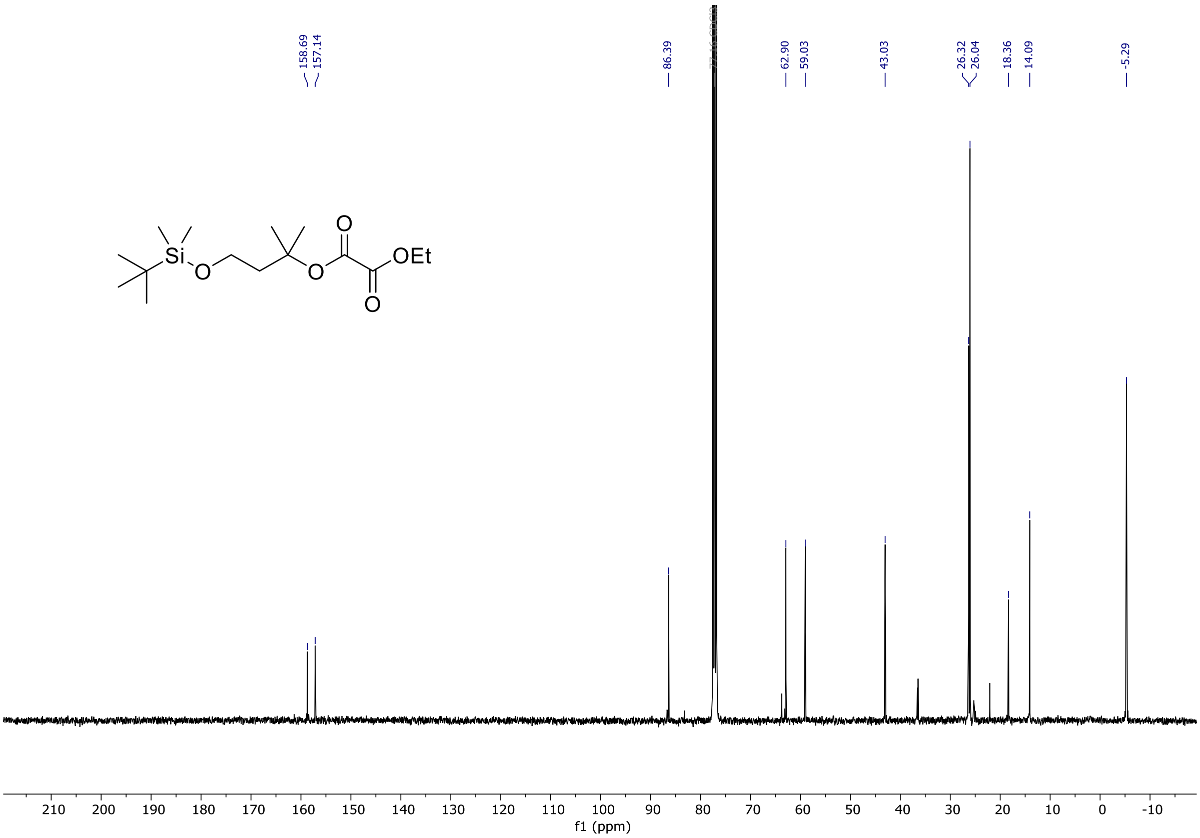

Compound **3p**

^{1}H NMR, DMSO, 400 MHz

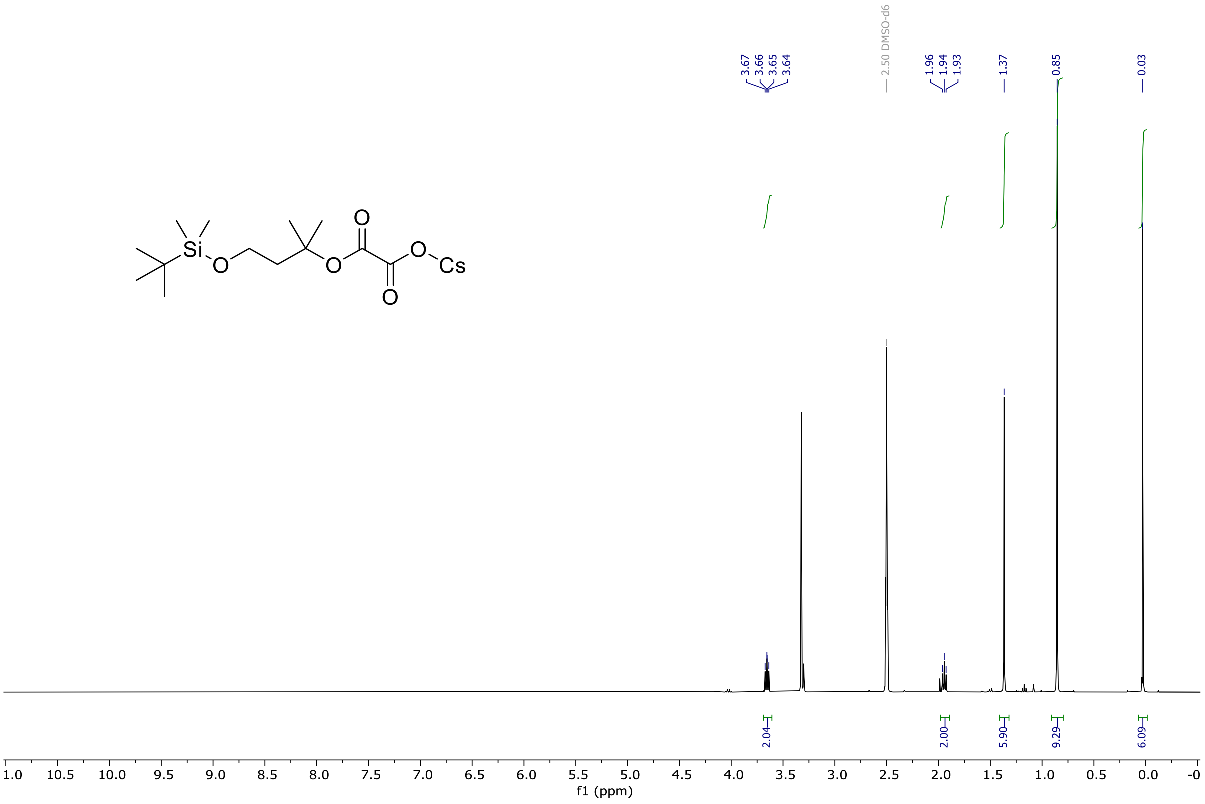

^{13}C NMR, DMSO, 101 MHz

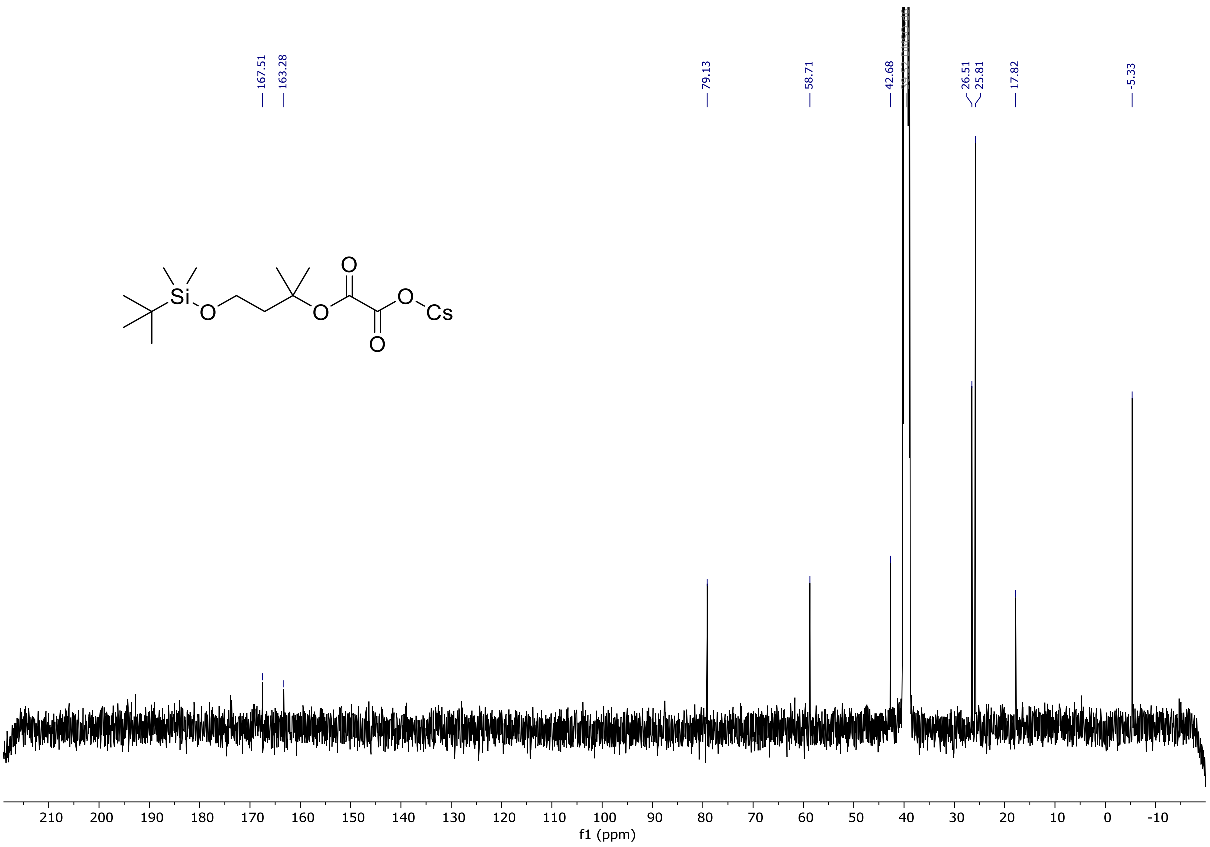

Compound **28q**

^{1}H NMR, $CDCl_3$, 400 MHz

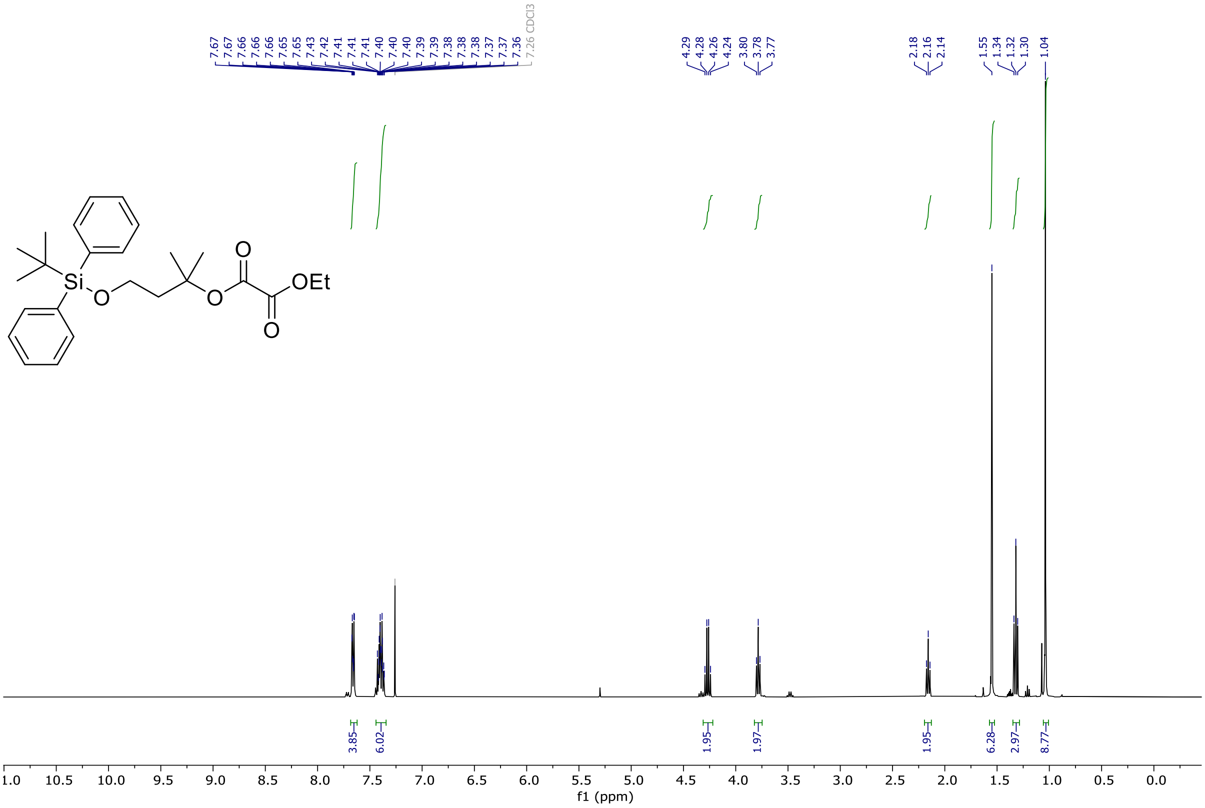

^{13}C NMR, $CDCl_3$, 101 MHz

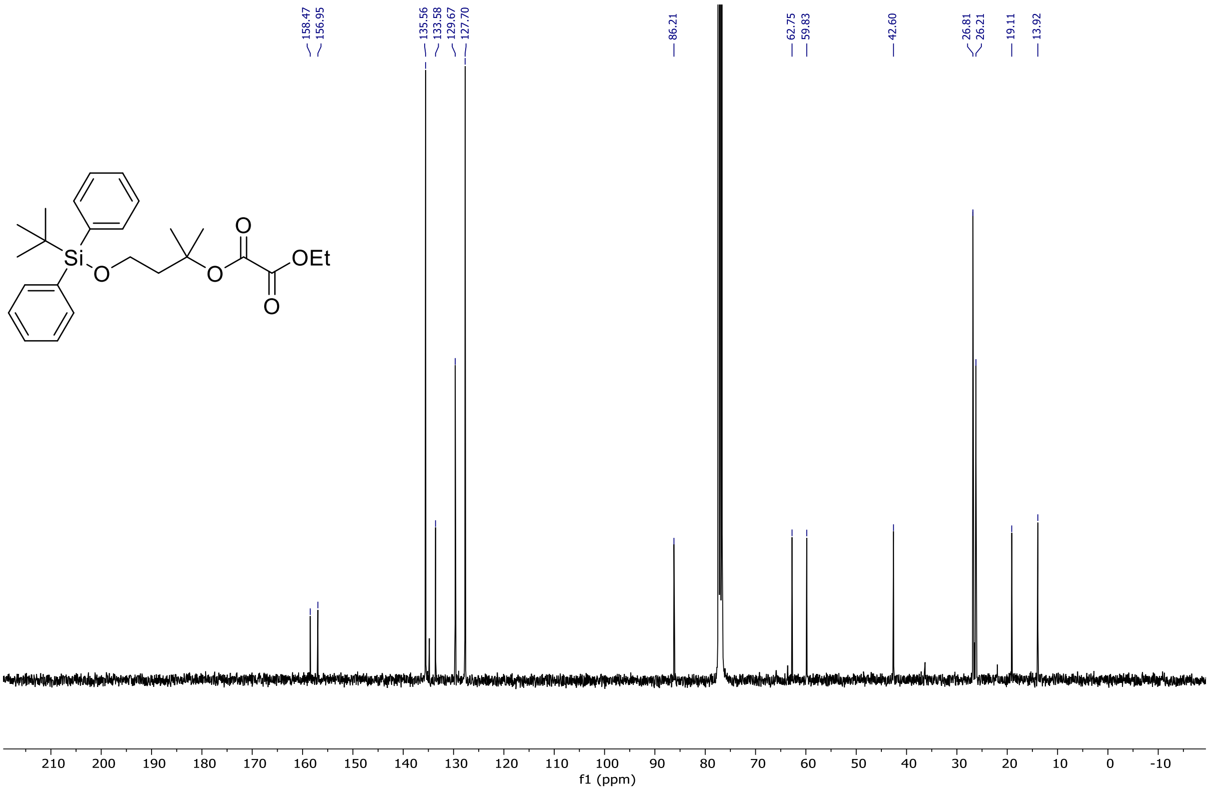

Compound **3q**

^{1}H NMR, DMSO, 400 MHz

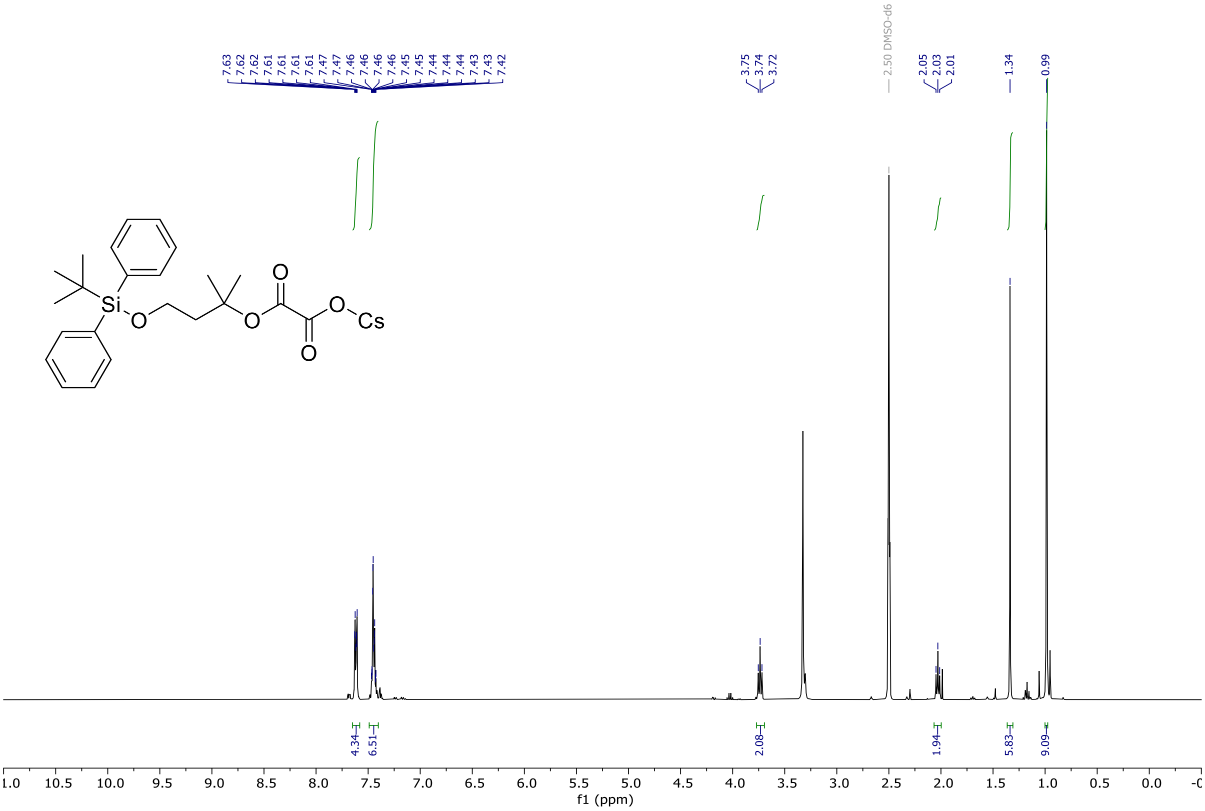

^{13}C NMR, DMSO, 101 MHz

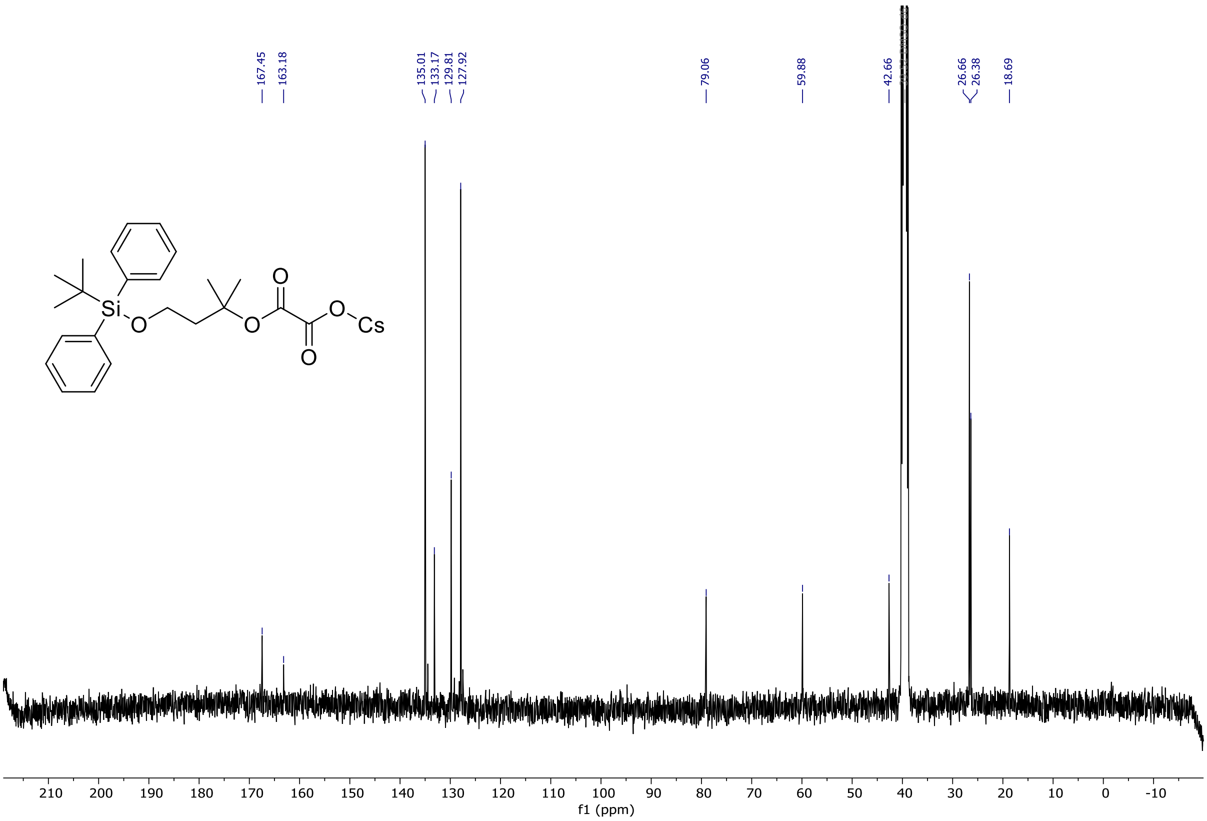

Compound **28w**

^{1}H NMR, $CDCl_3$, 400 MHz

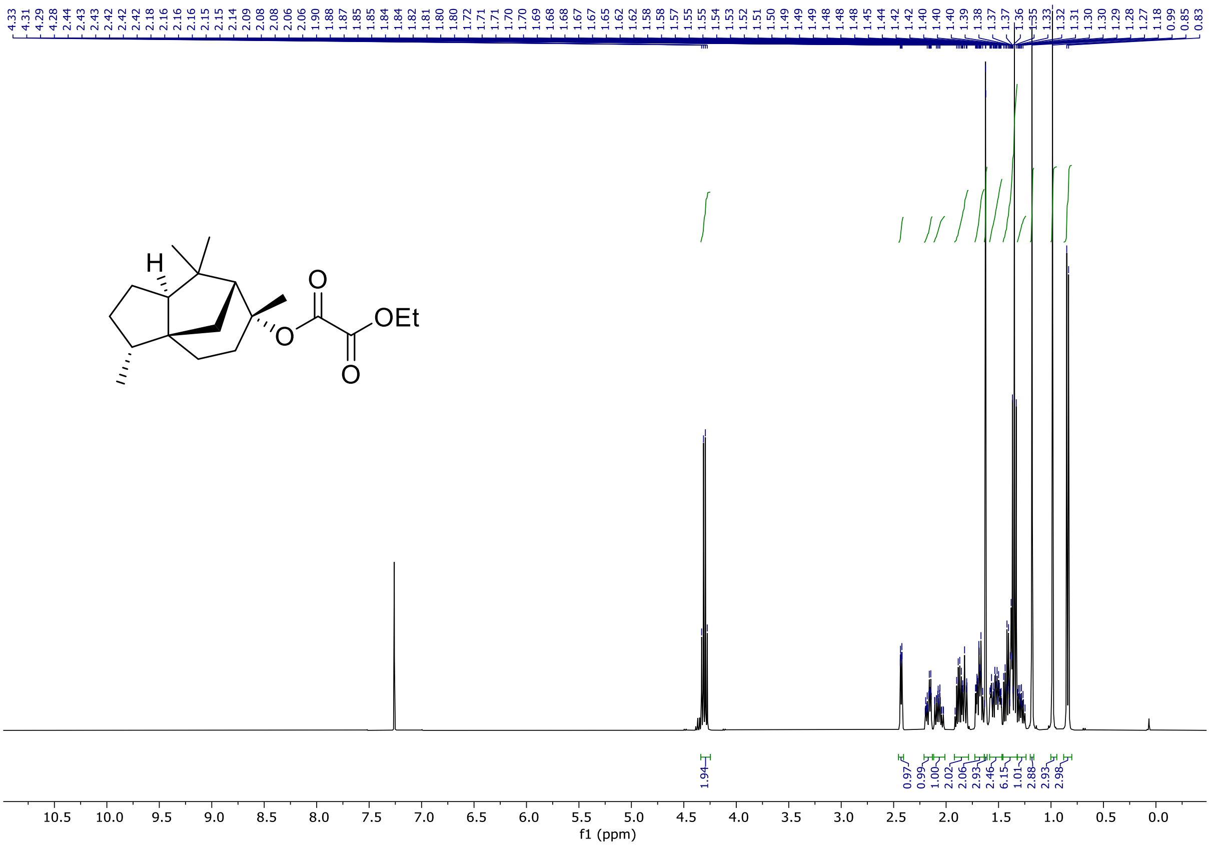

^{13}C NMR, $CDCl_3$, 101 MHz

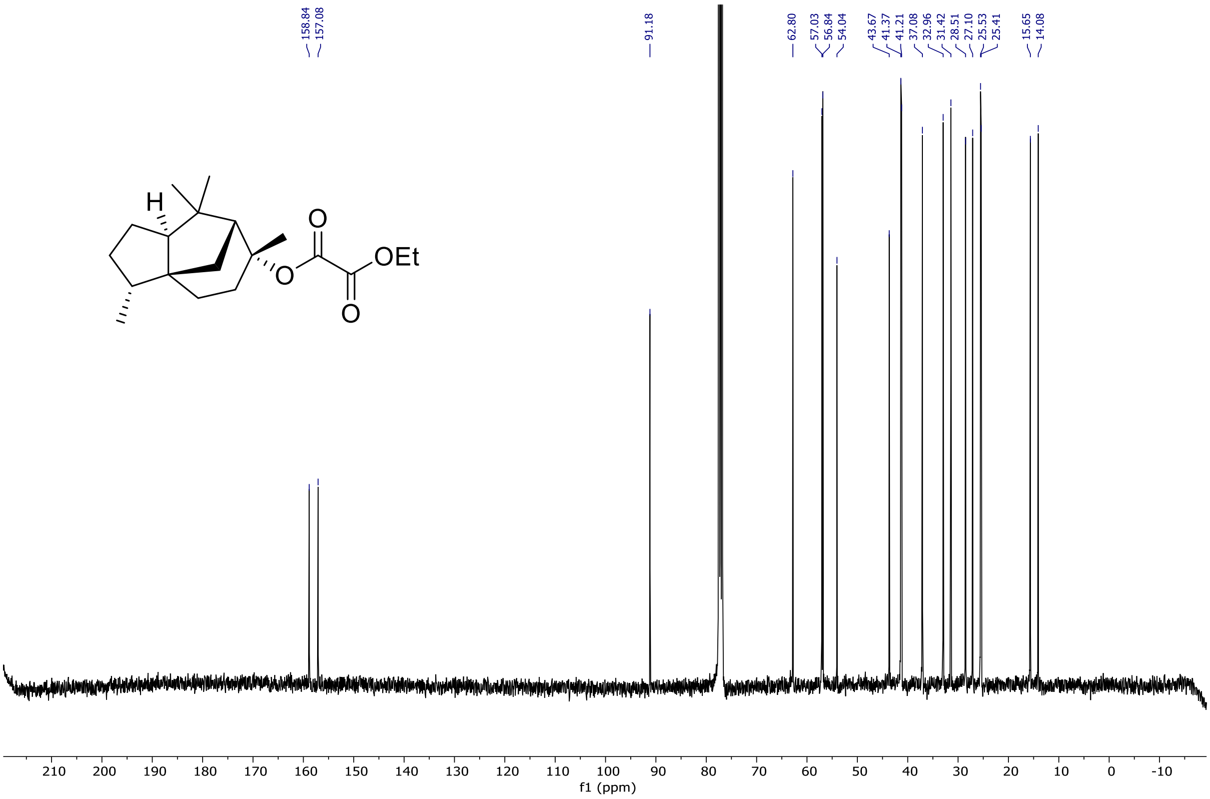

Compound **3w**

^{1}H NMR, DMSO, 400 MHz

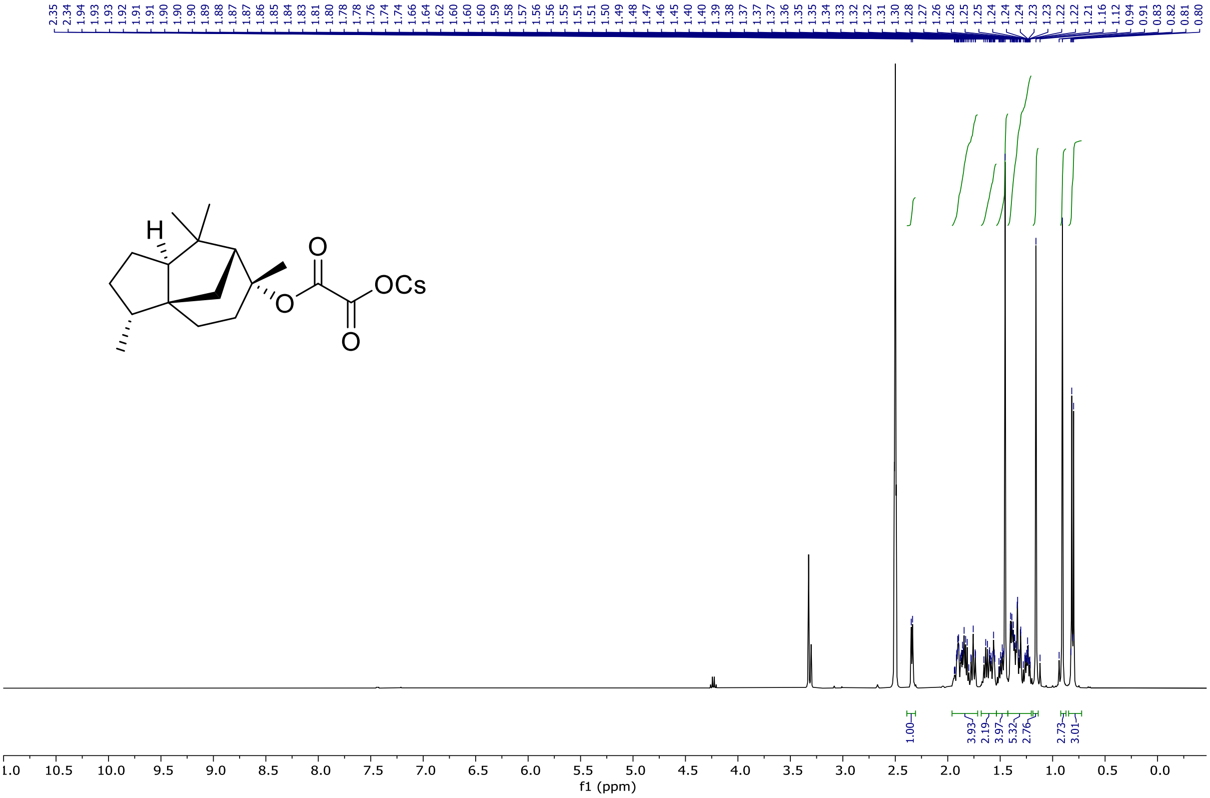

^{13}C NMR, DMSO, 101 MHz

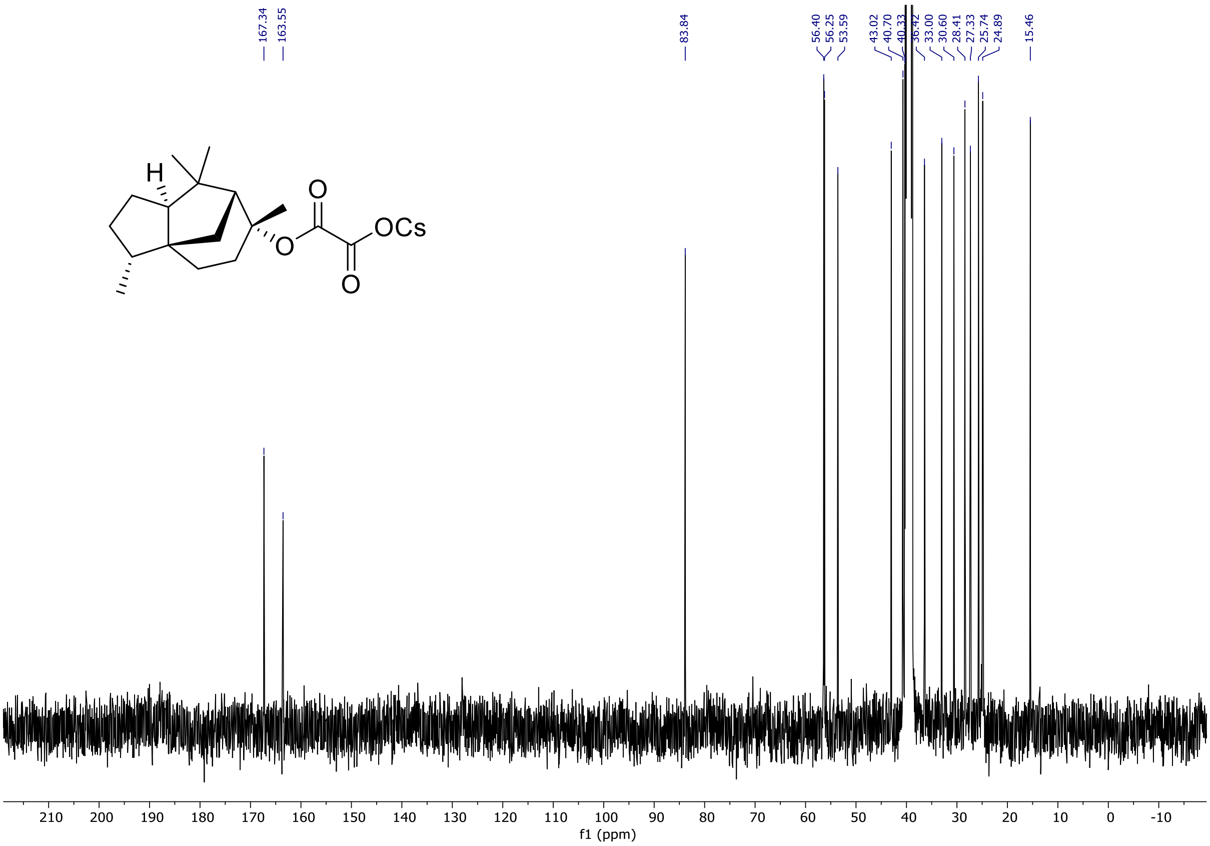

Compound **28x**

^{1}H NMR, $CDCl_3$, 400 MHz

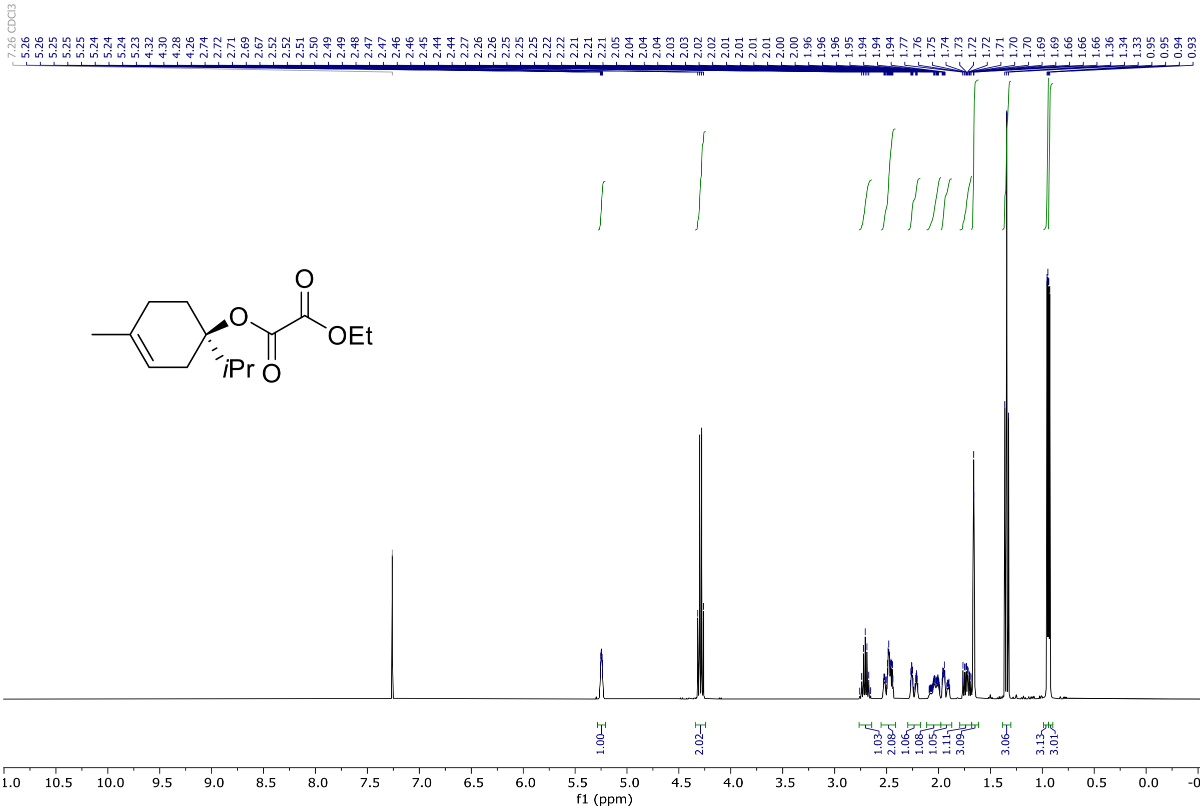

^{13}C NMR, $CDCl_3$, 101 MHz

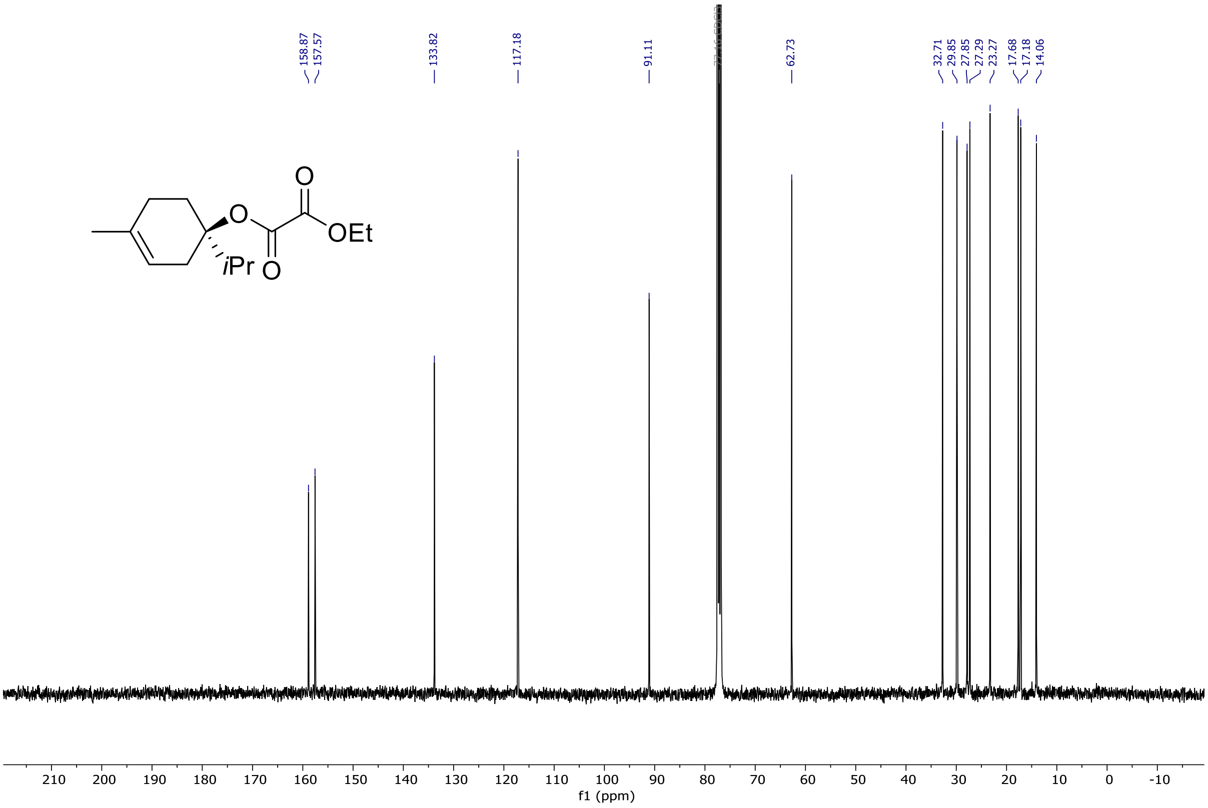

Compound **3x**

^{1}H NMR, DMSO, 400 MHz

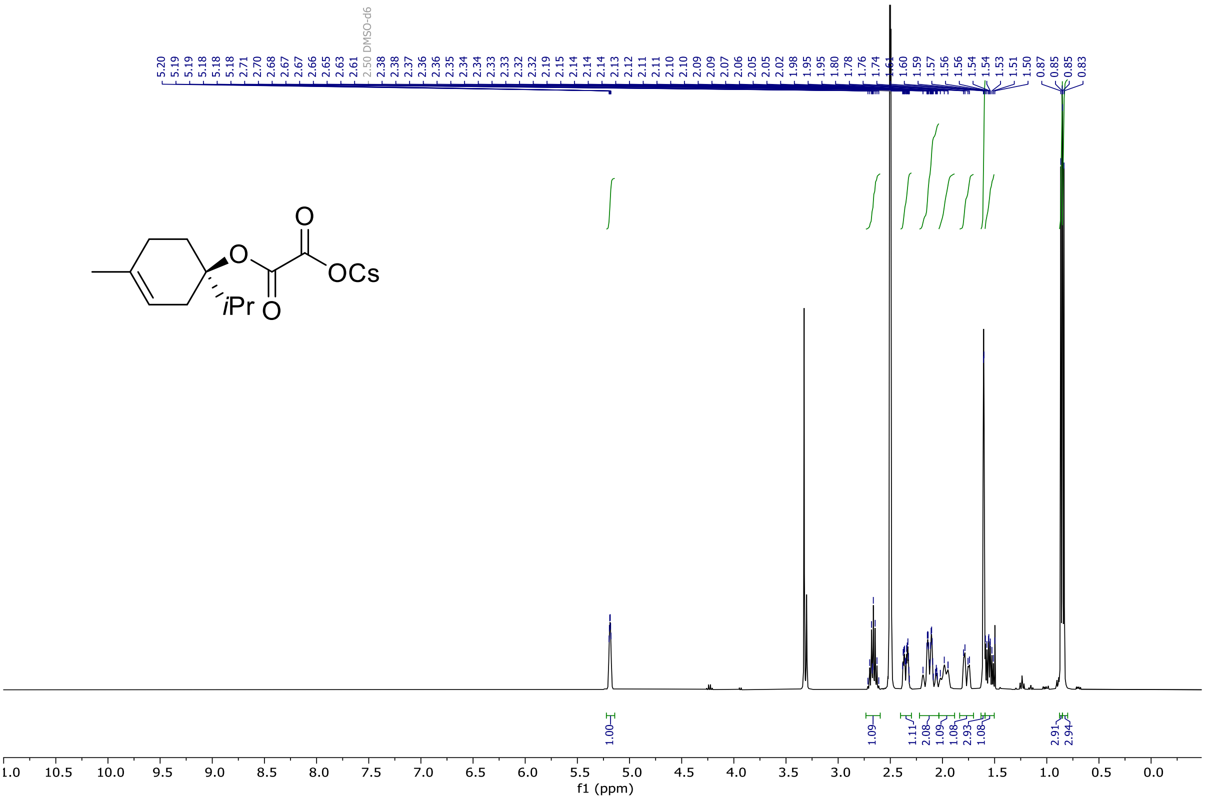

^{13}C NMR, DMSO, 101 MHz

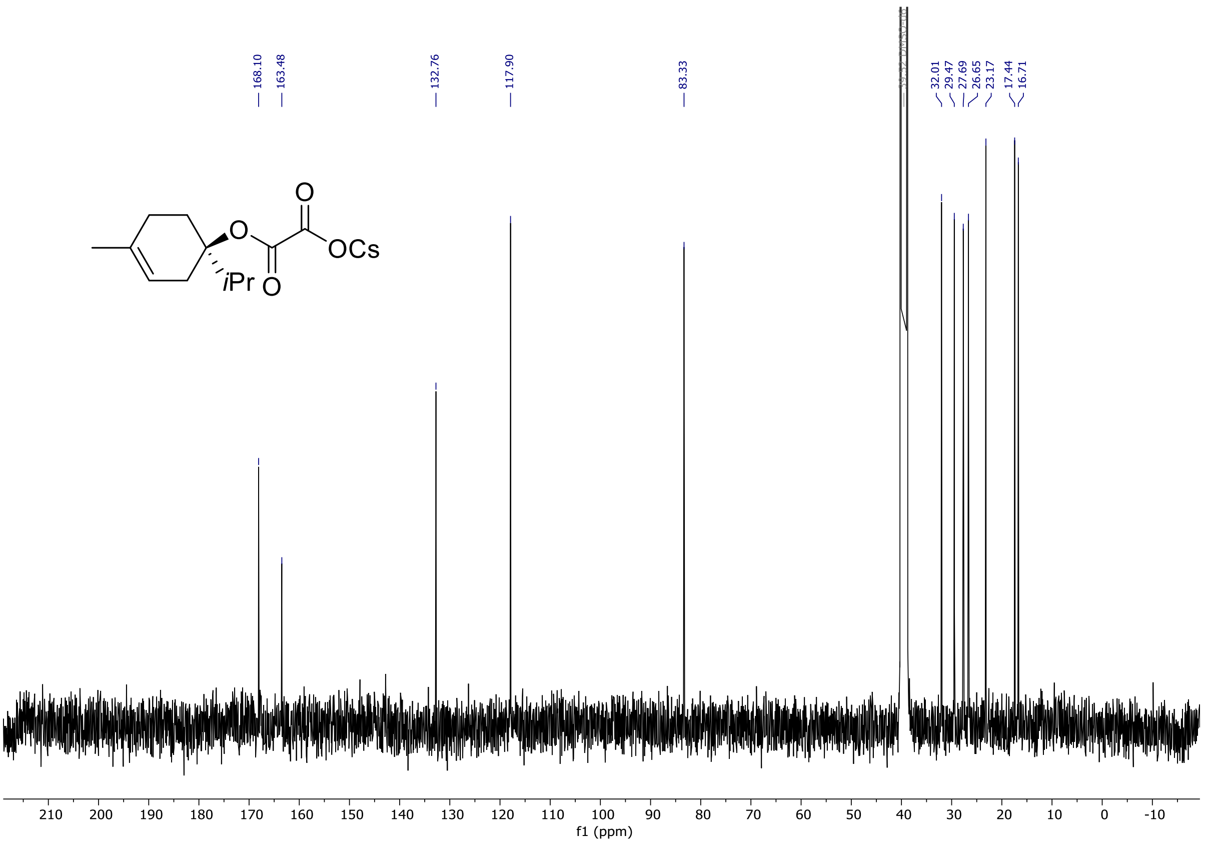

Compound **9a**

^{1}H NMR, $CDCl_3$, 400 MHz

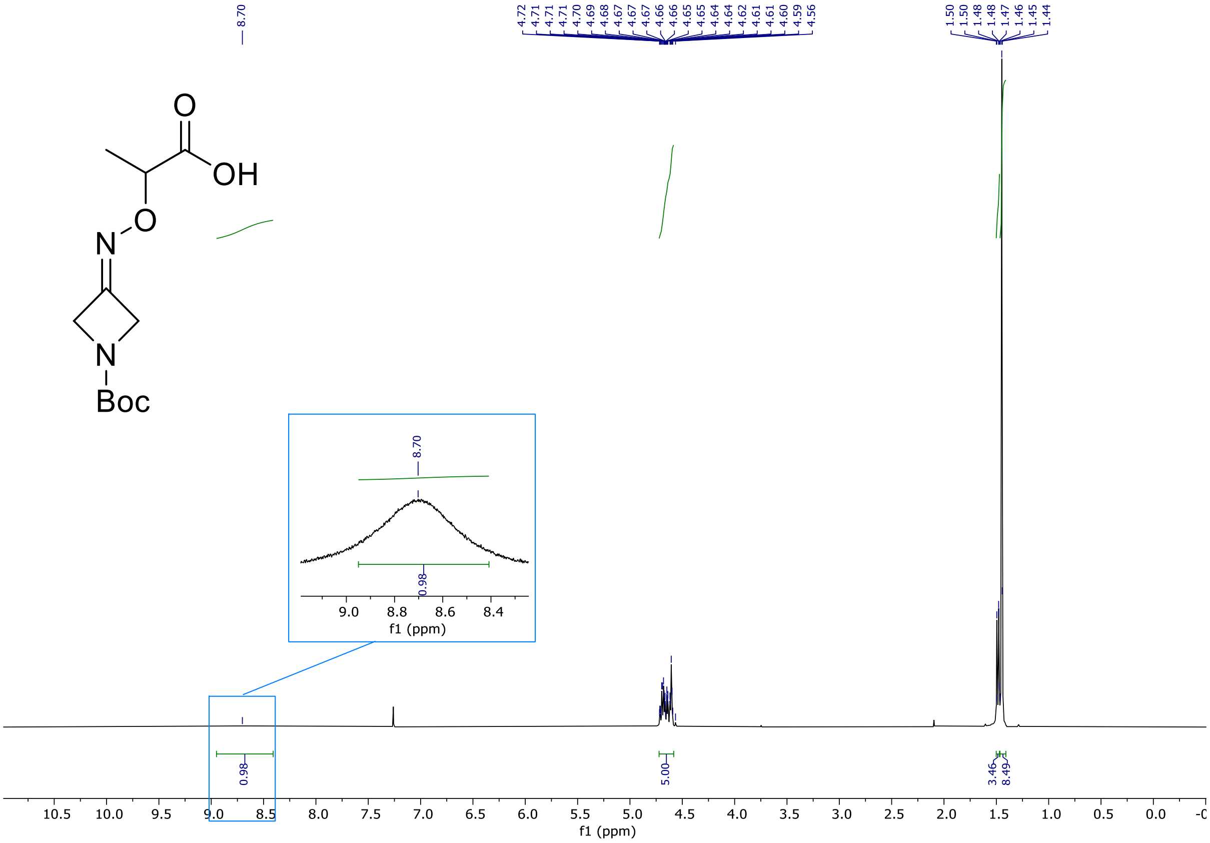

^{13}C NMR, $CDCl_3$, 101 MHz

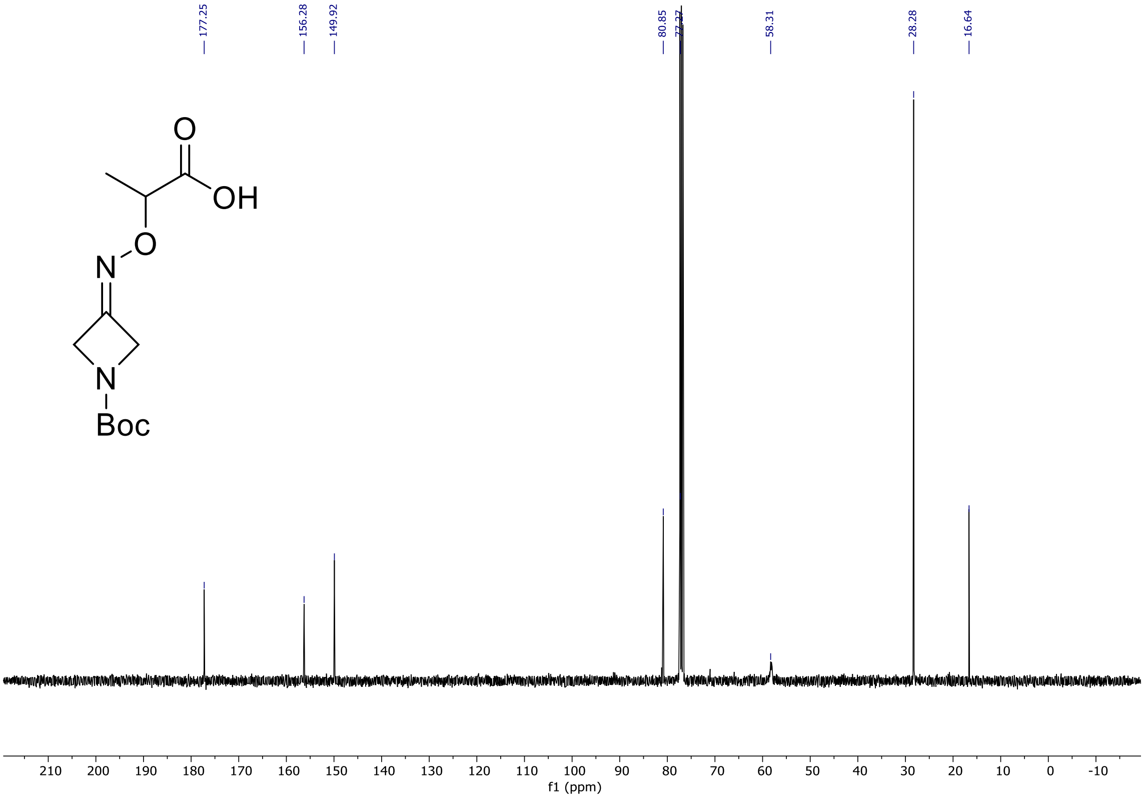

Compound **9c**

^{1}H NMR, $CDCl_3$, 400 MHz

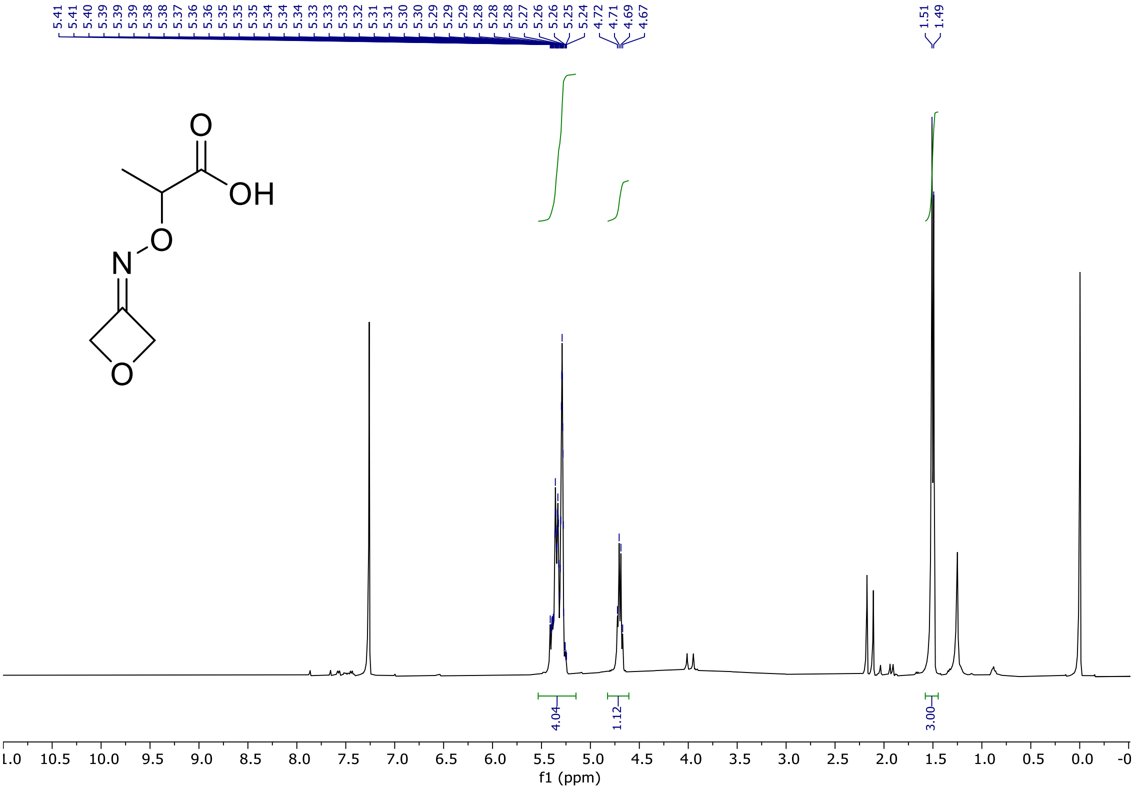

^{13}C NMR, $CDCl_3$, 101 MHz

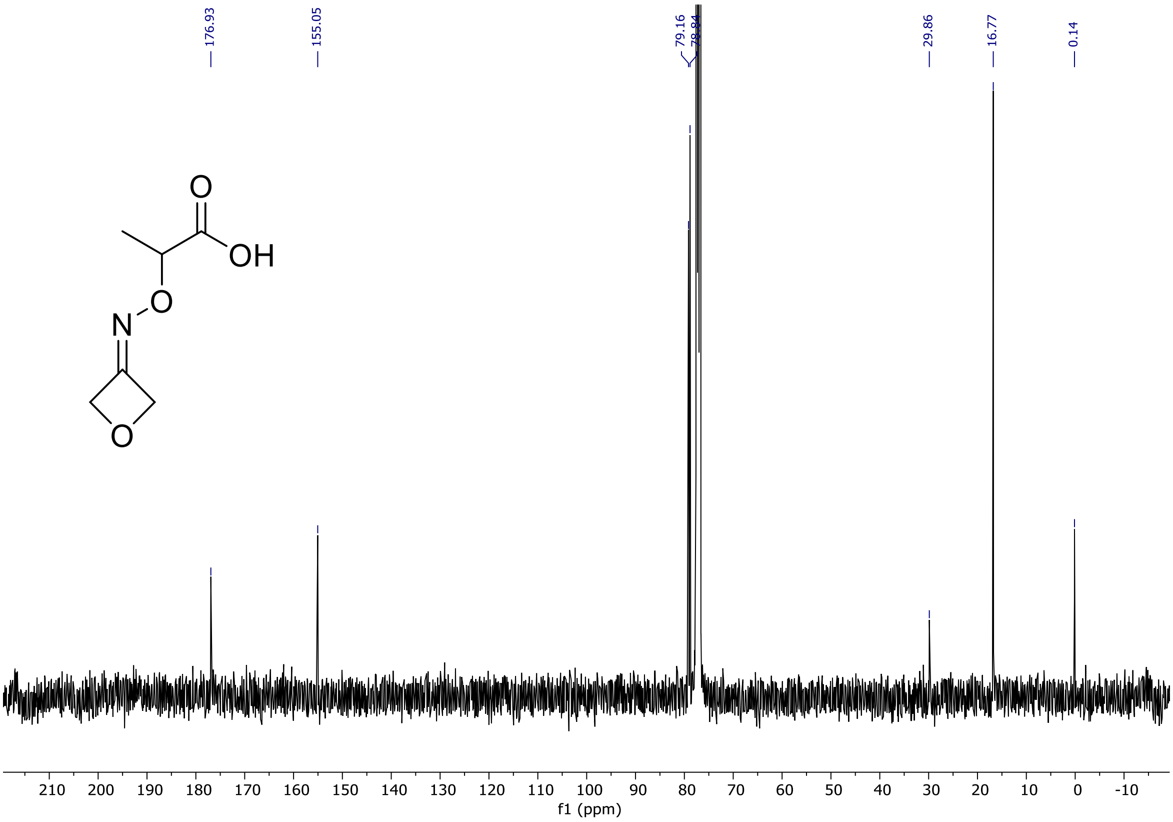

Compound **11a** (previously reported)

^{1}H NMR, Acetone, 400 MHz

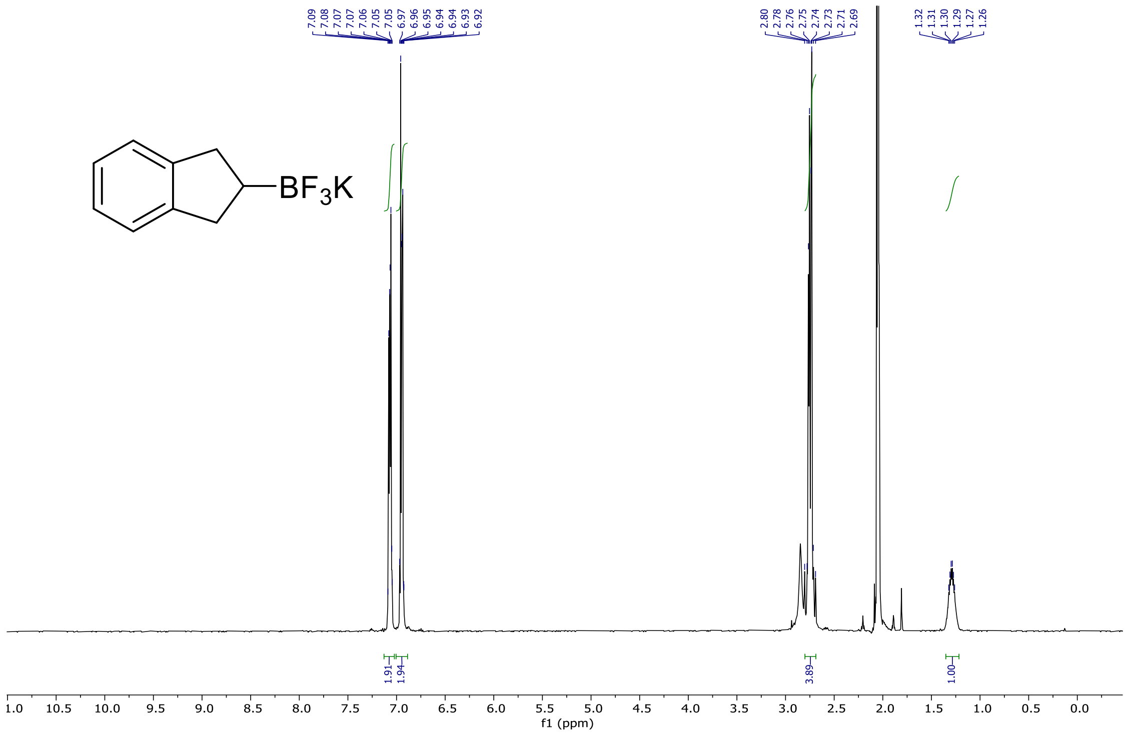

Compound **11c** (previously reported)

^{1}H NMR, DMSO, 400 MHz

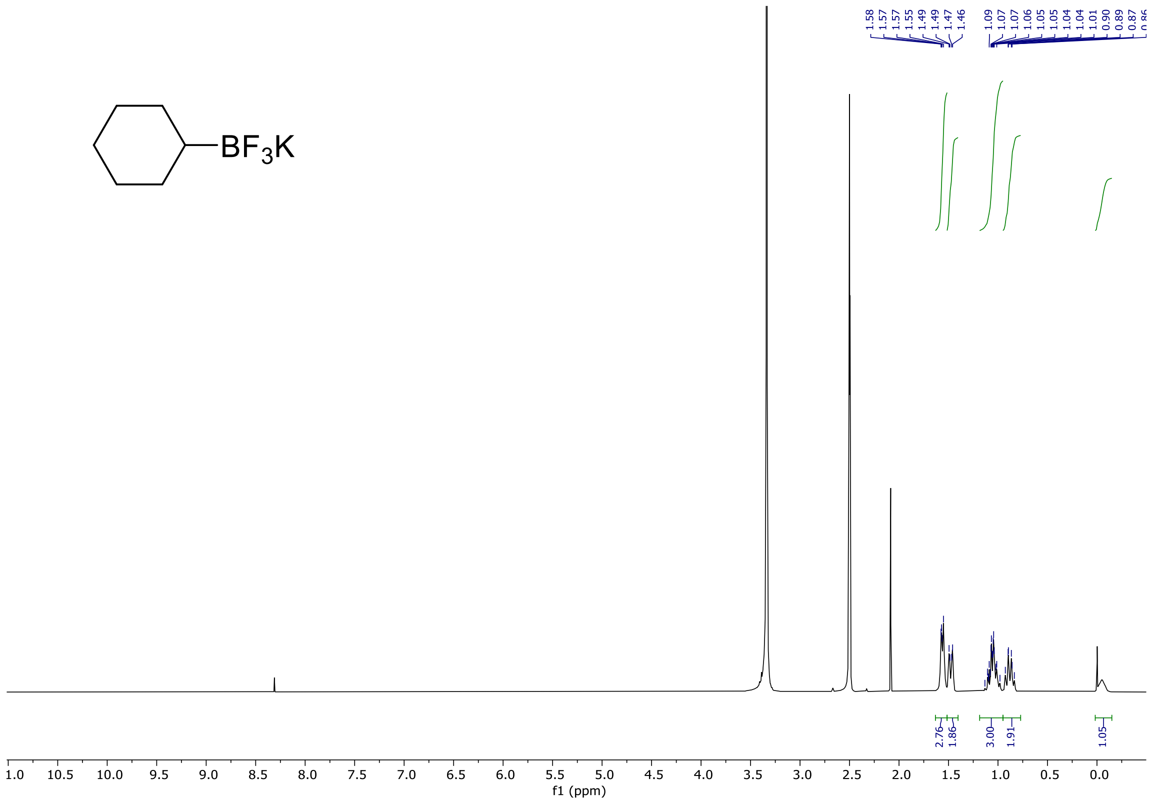

Compound **4a**

^{1}H NMR, $CDCl_3$, 400 MHz

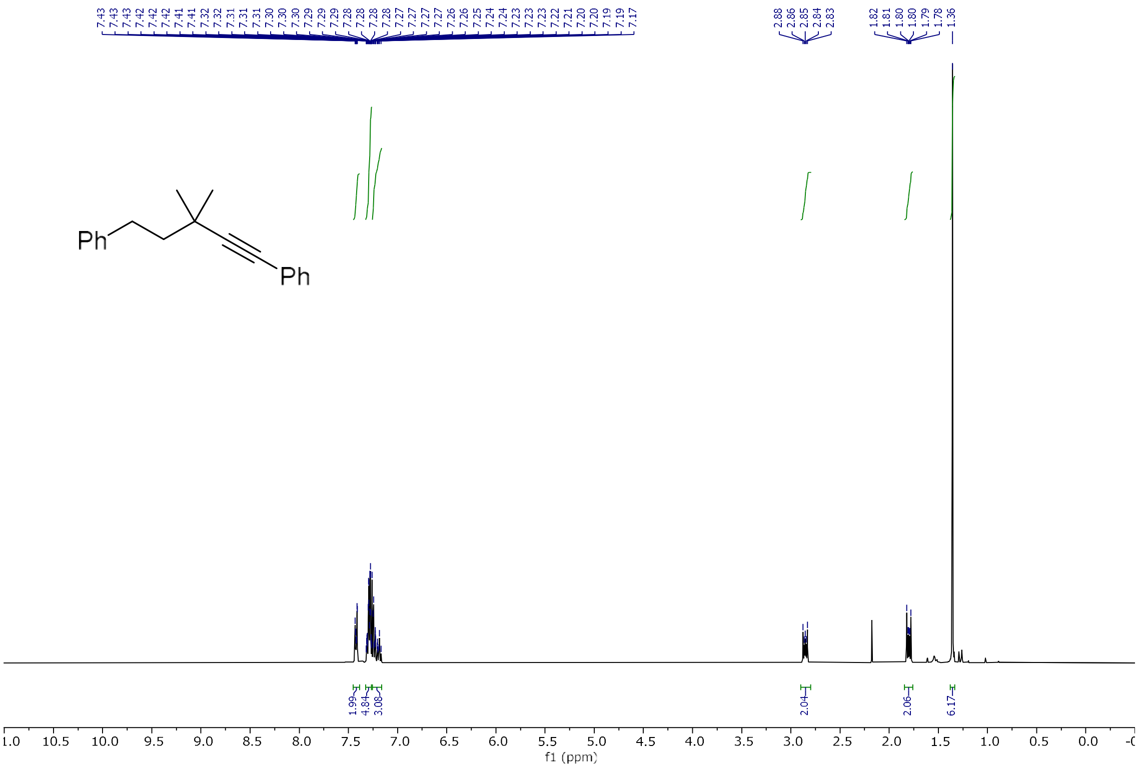

^{13}C NMR, $CDCl_3$, 101 MHz

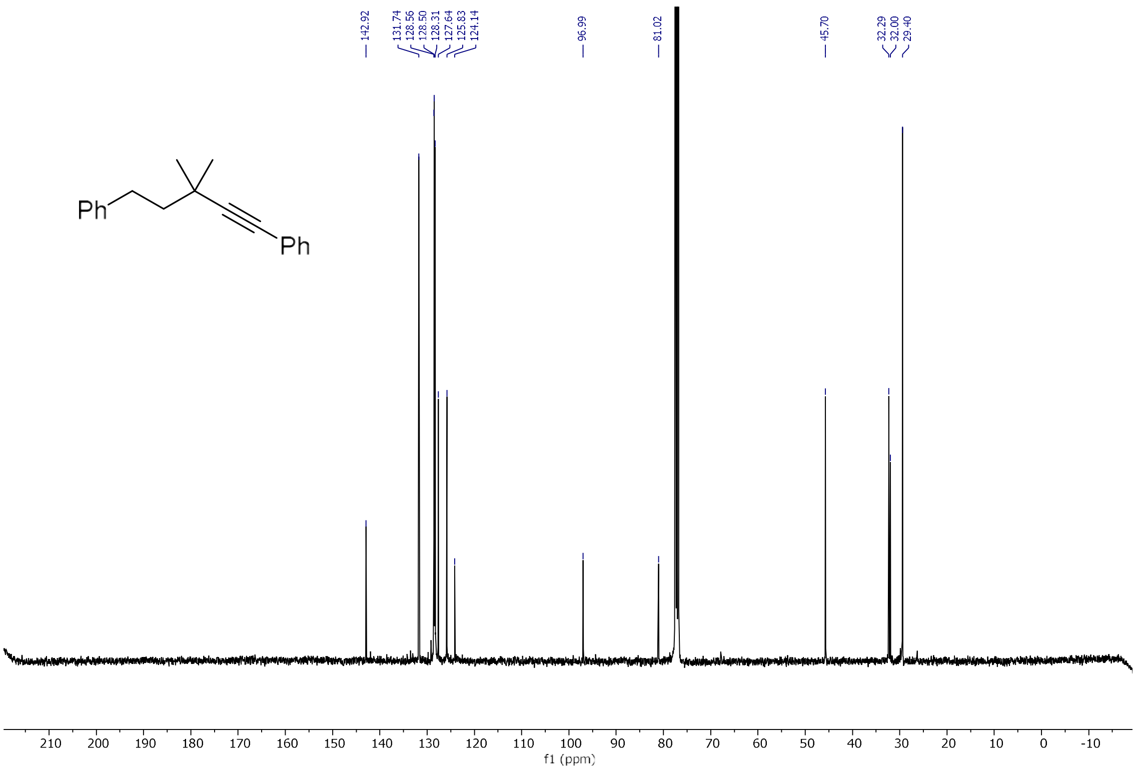

Compound **4b**

^{1}H NMR, $CDCl_3$, 400 MHz

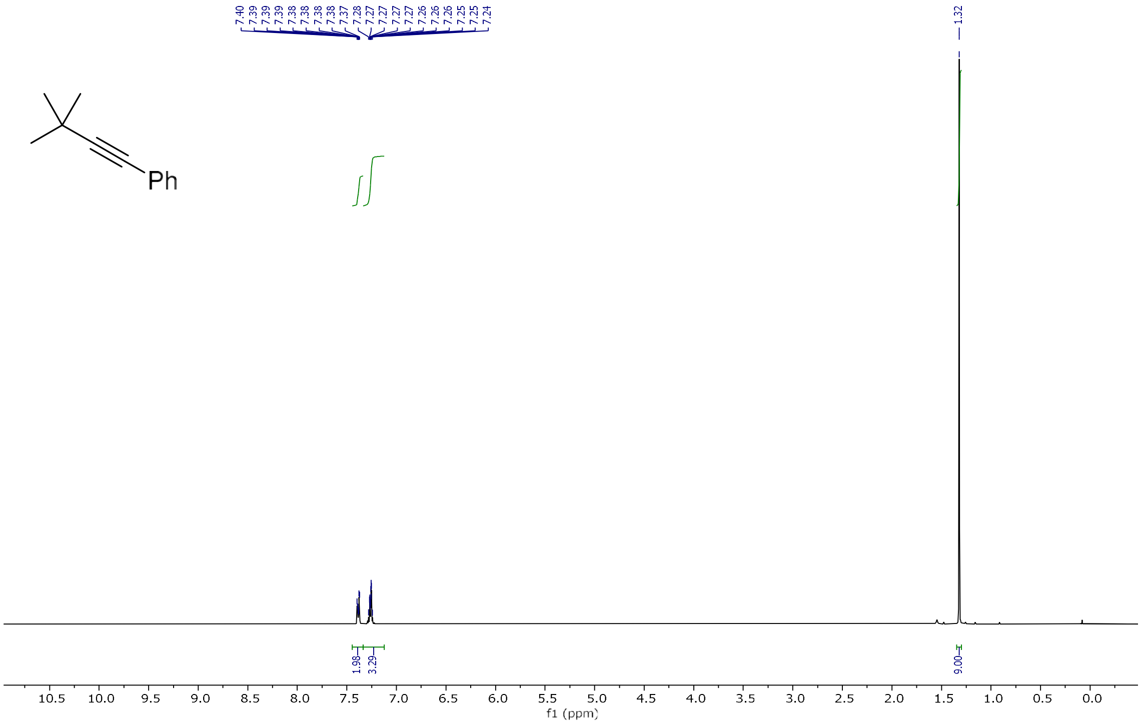

^{13}C NMR, $CDCl_3$, 101 MHz

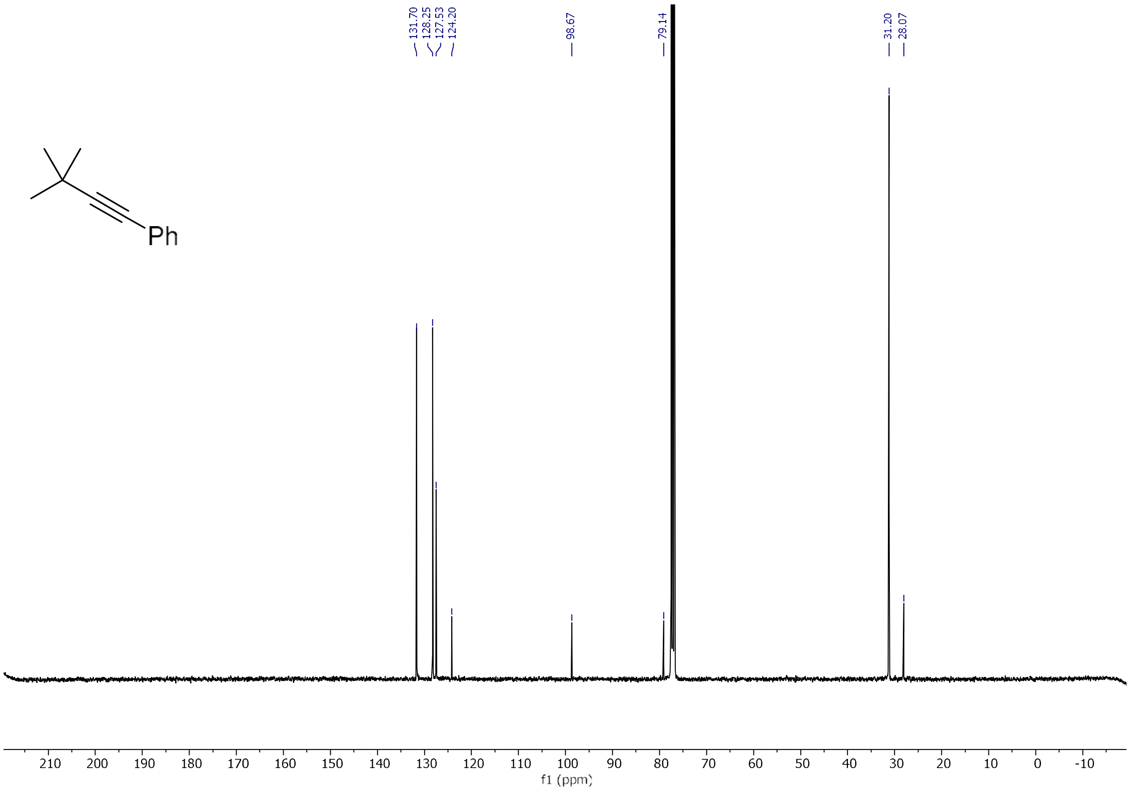

Compound **4c**

^{1}H NMR, $CDCl_3$, 400 MHz

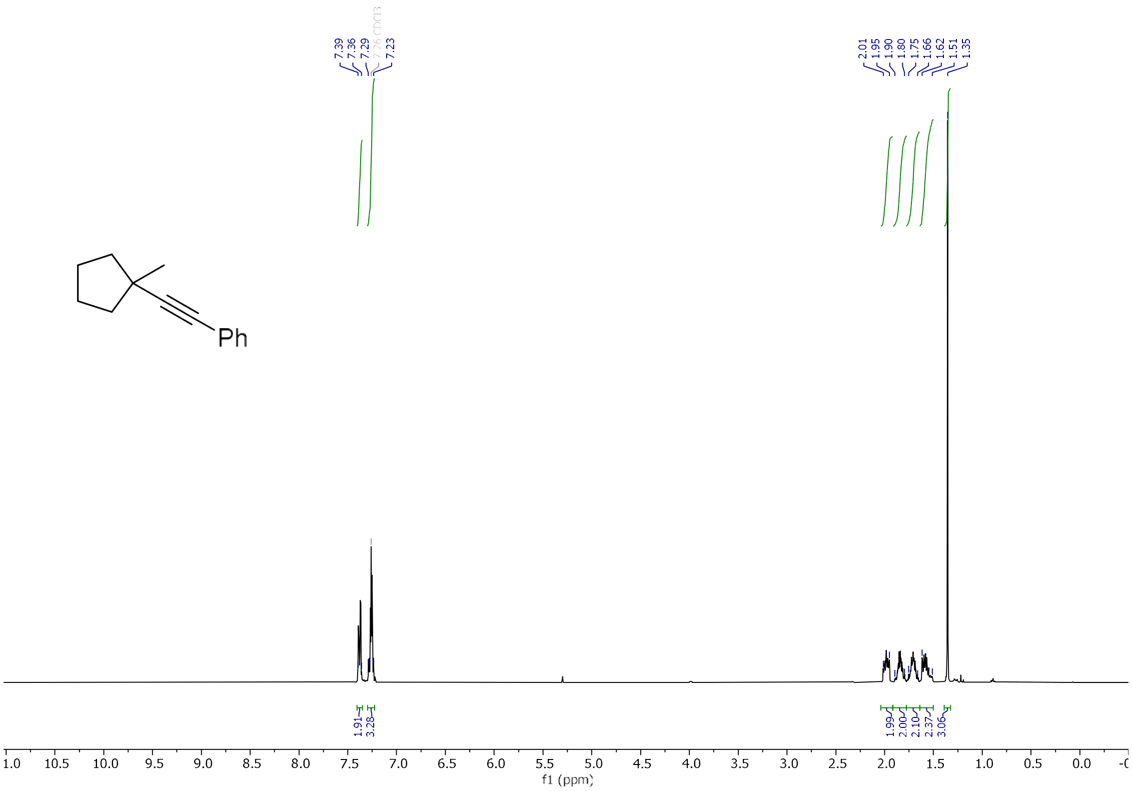

^{13}C NMR, $CDCl_3$, 101 MHz

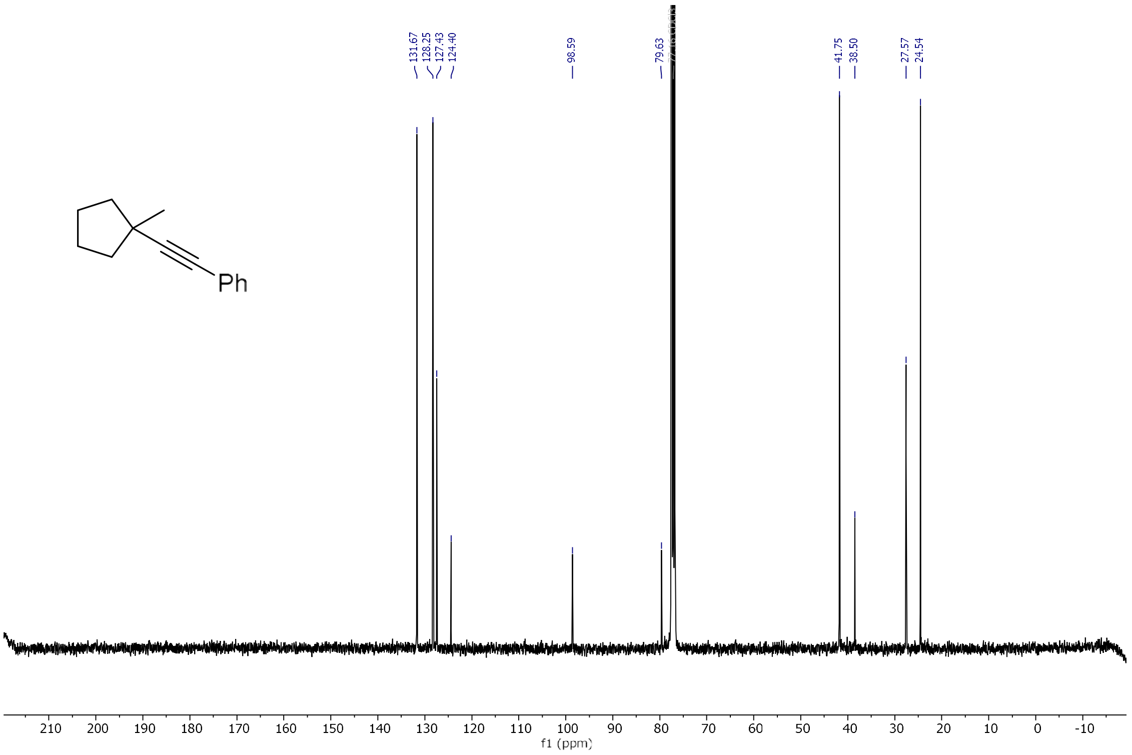

Compound **4d**

^{1}H NMR, $CDCl_3$, 400 MHz

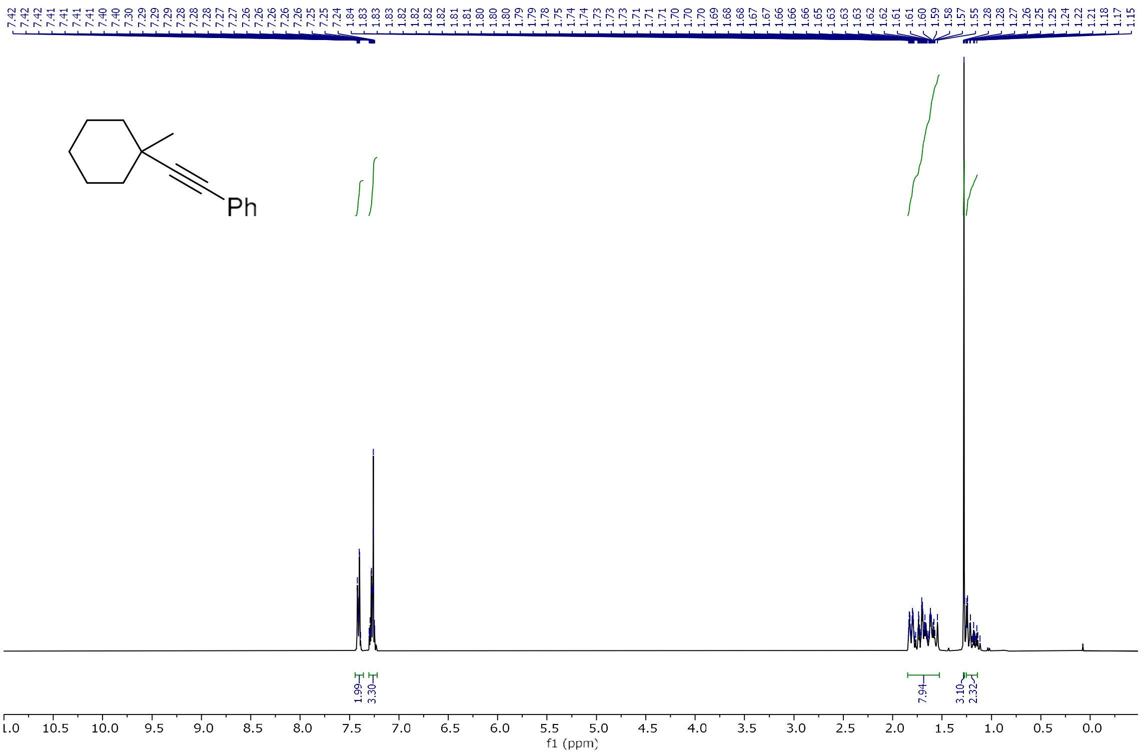

^{13}C NMR, $CDCl_3$, 400 MHz

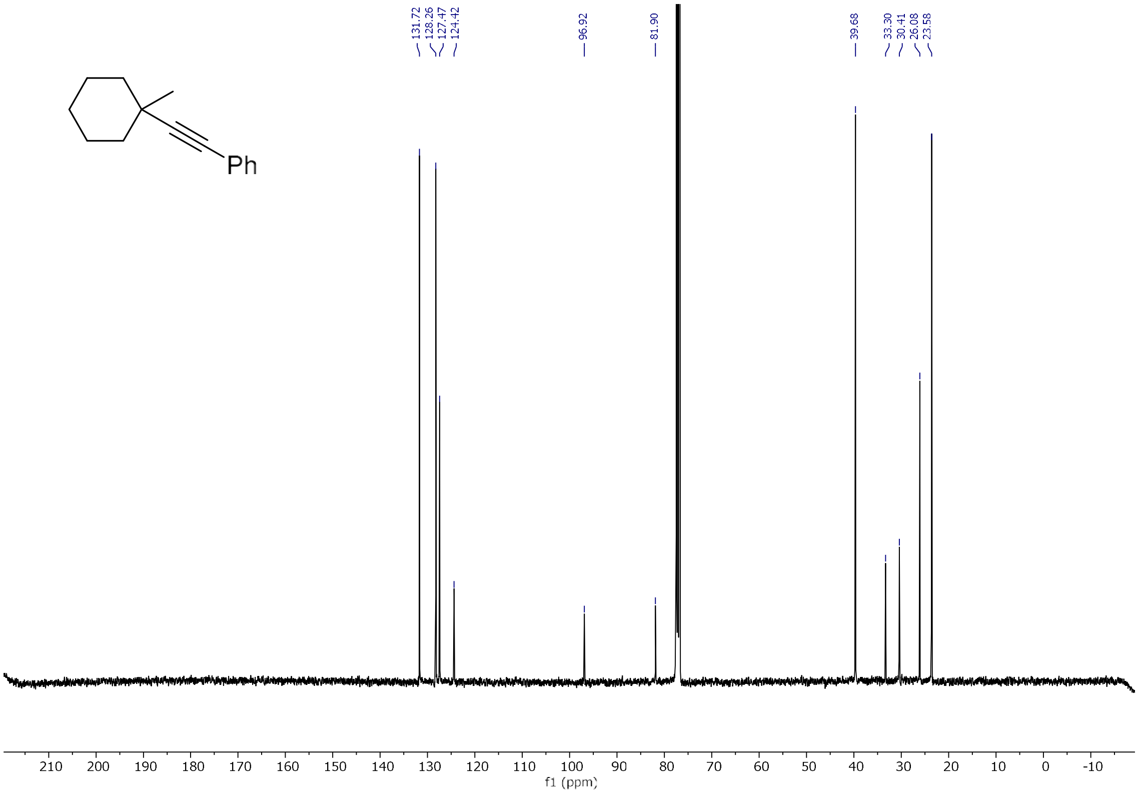

Compound **4e**

^{1}H NMR, $CDCl_3$, 400 MHz

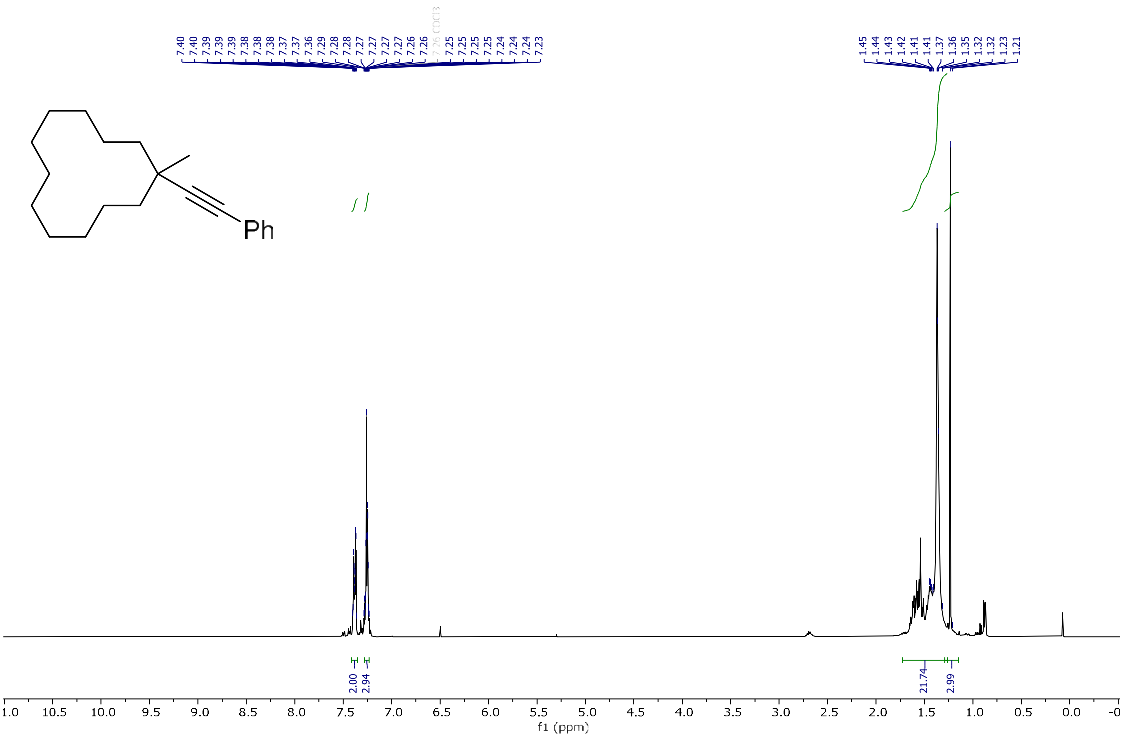

^{13}C NMR, $CDCl_3$, 101 MHz

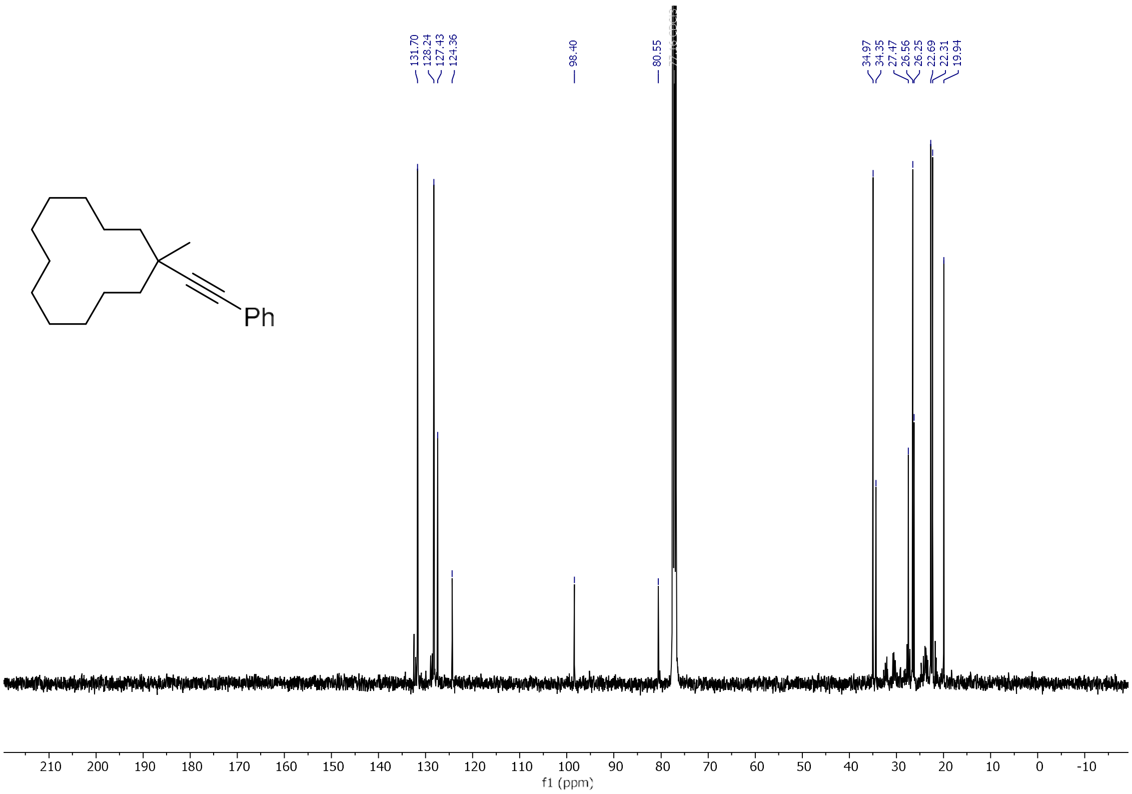

Compound **4f**

^{1}H NMR, $CDCl_3$, 400 MHz

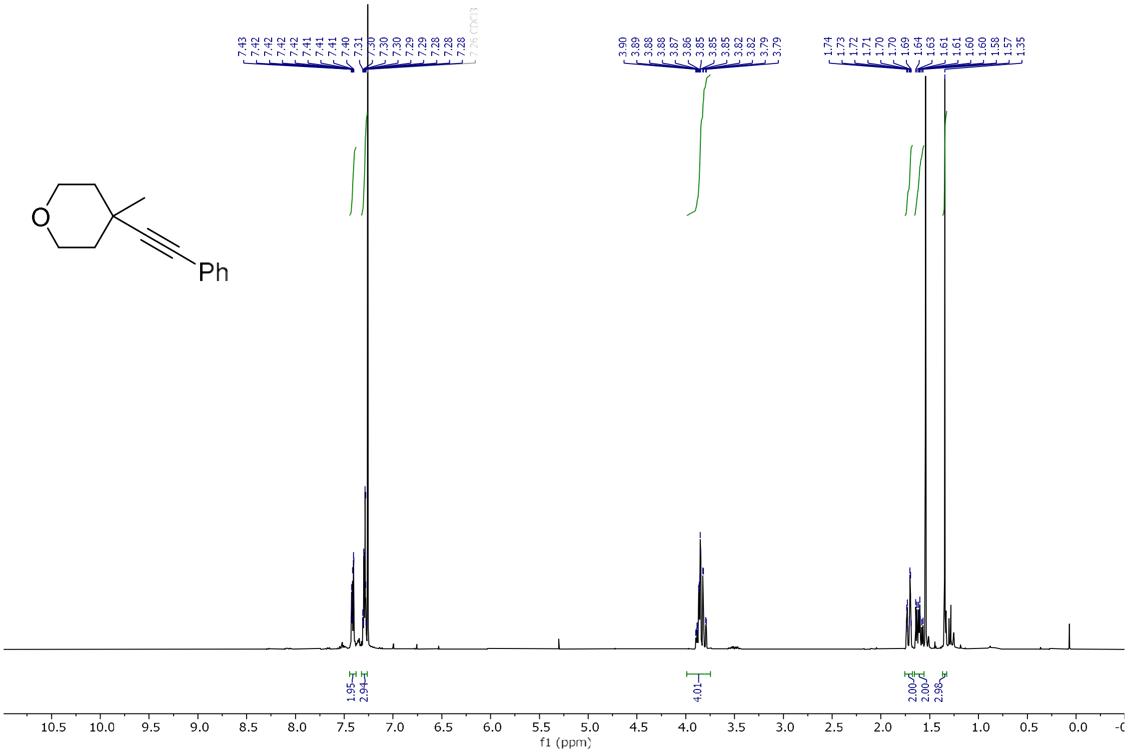

^{13}C NMR, $CDCl_3$, 101 MHz

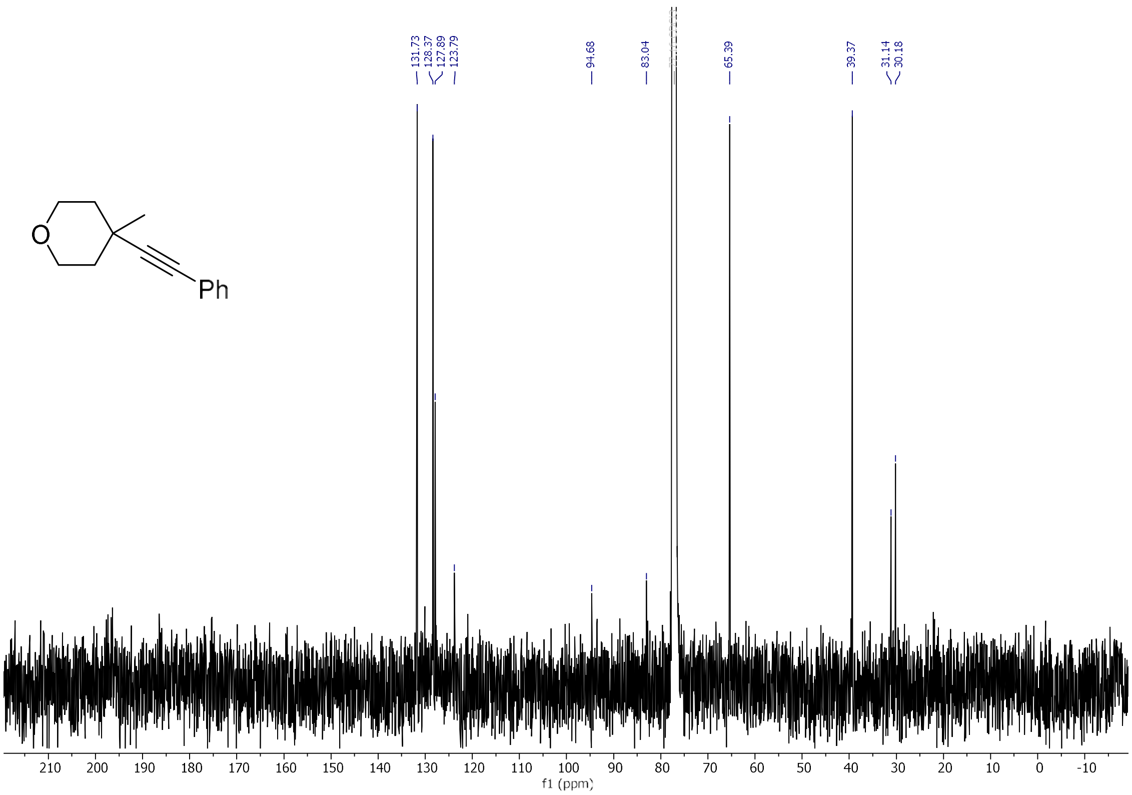

Compound **4g**

^{1}H NMR, $CDCl_3$, 400 MHz

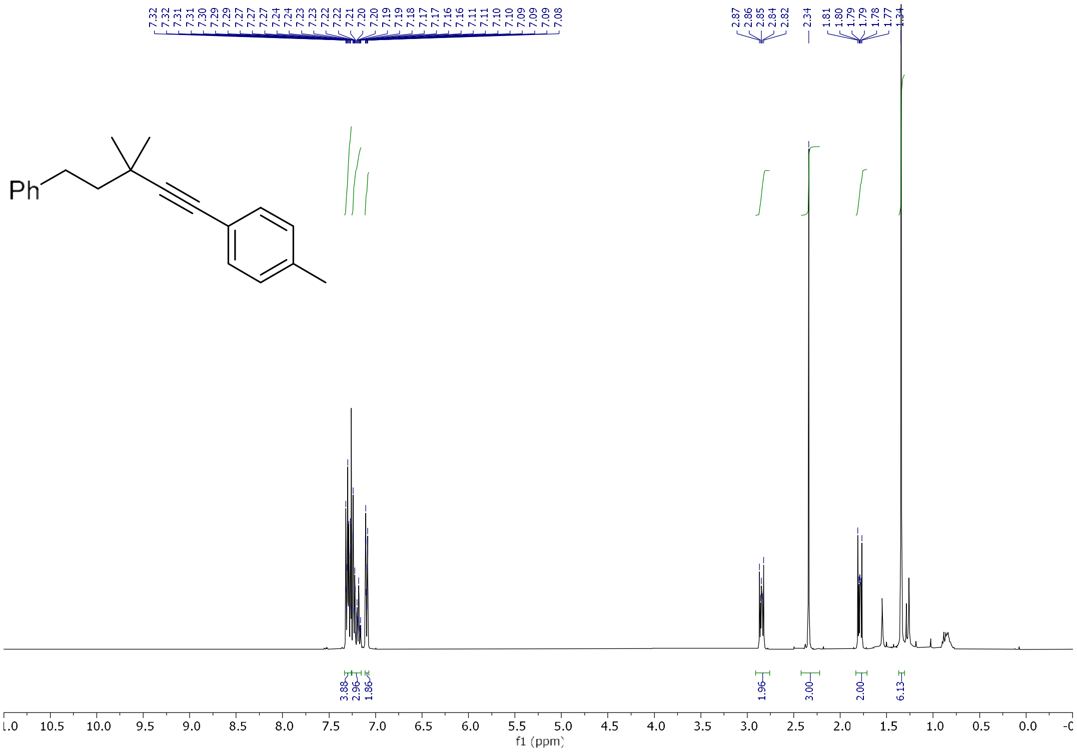

^{13}C NMR, $CDCl_3$, 101 MHz

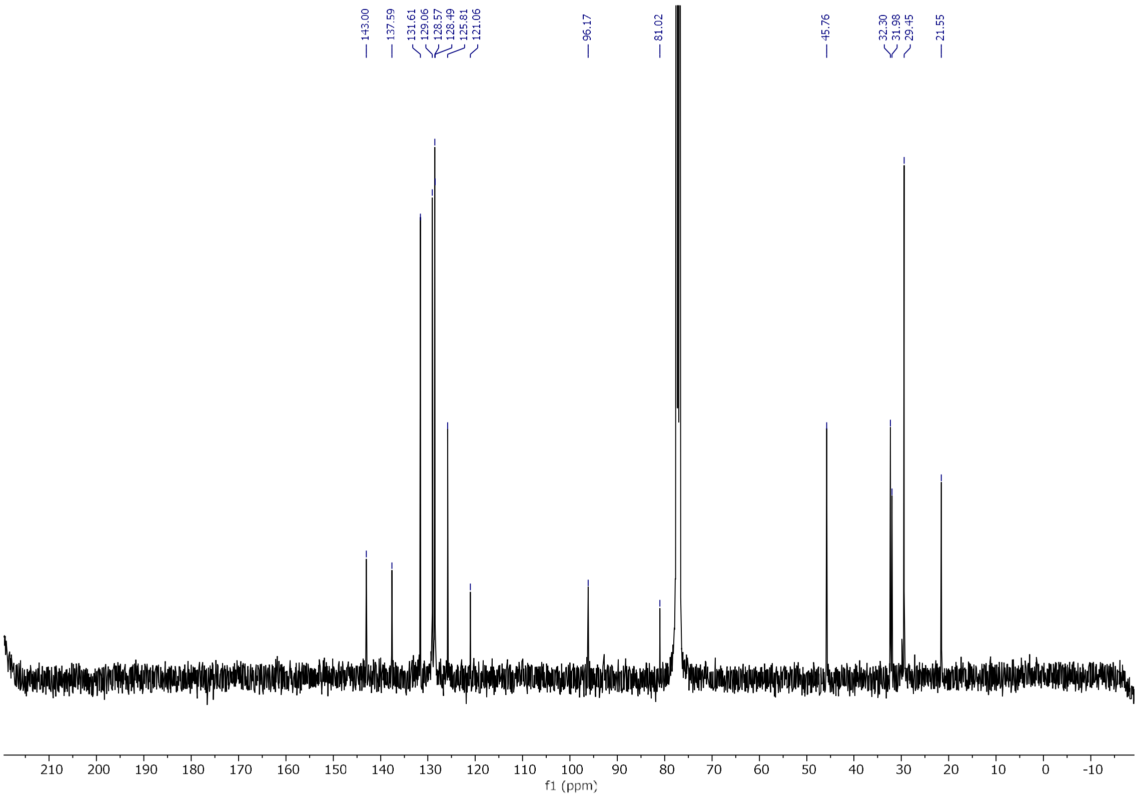

Compound **4h**

^{1}H NMR, $CDCl_3$, 400 MHz

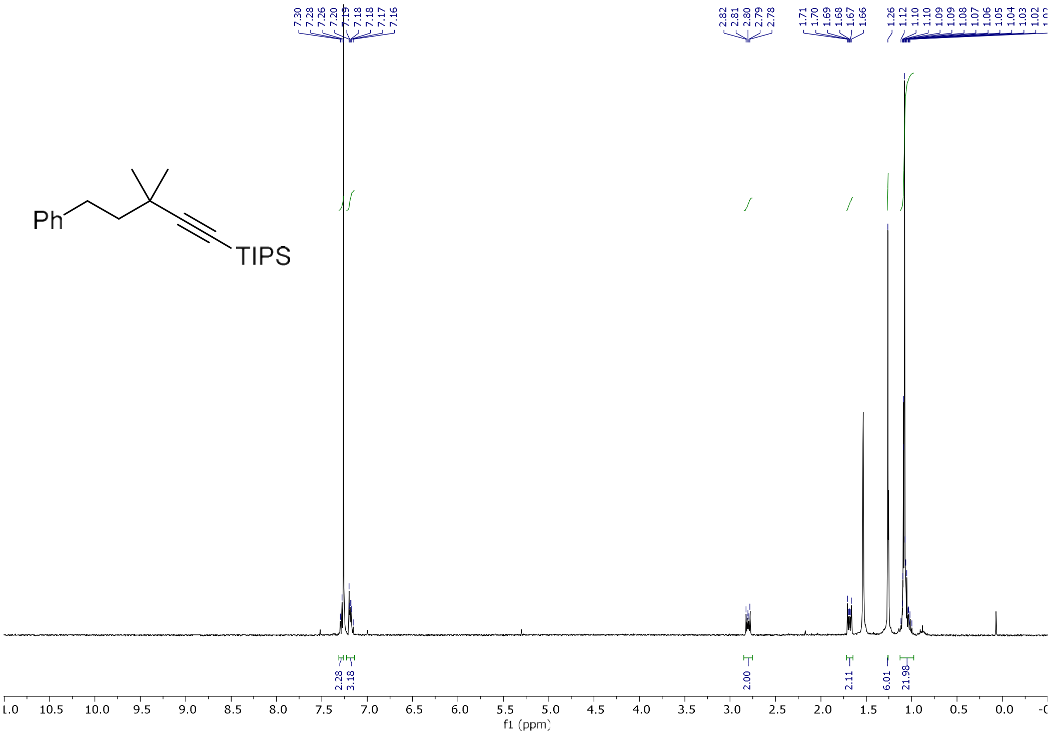

^{13}C NMR, $CDCl_3$, 101 MHz

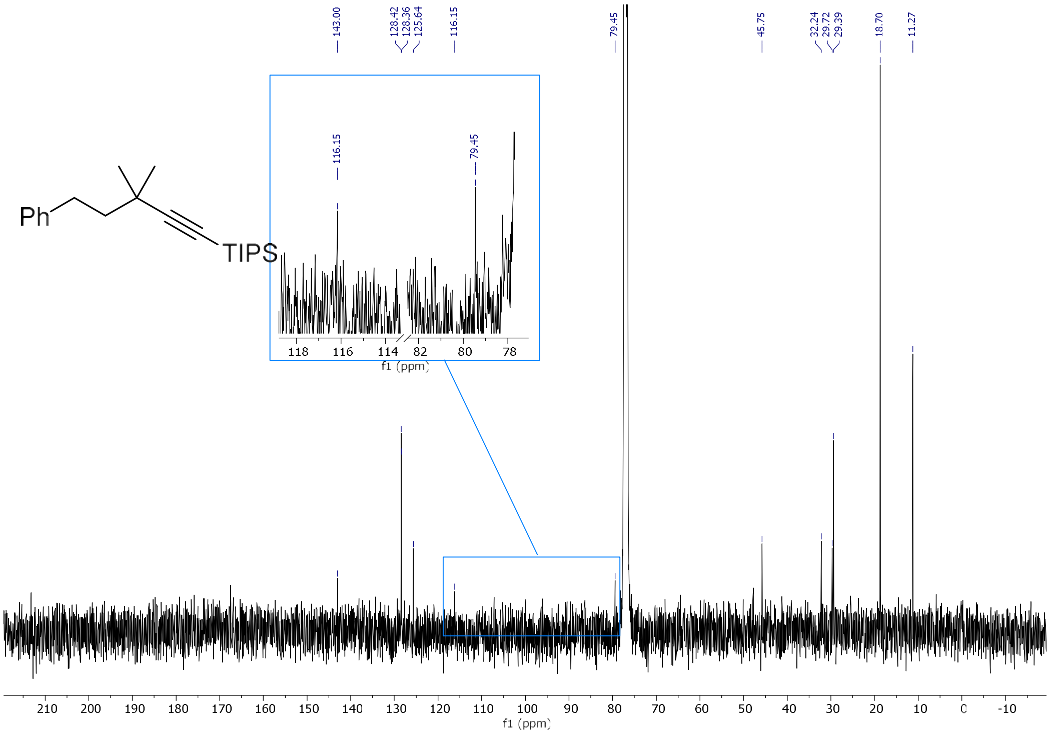

Compound **4i**

^{1}H NMR, $CDCl_3$, 400 MHz

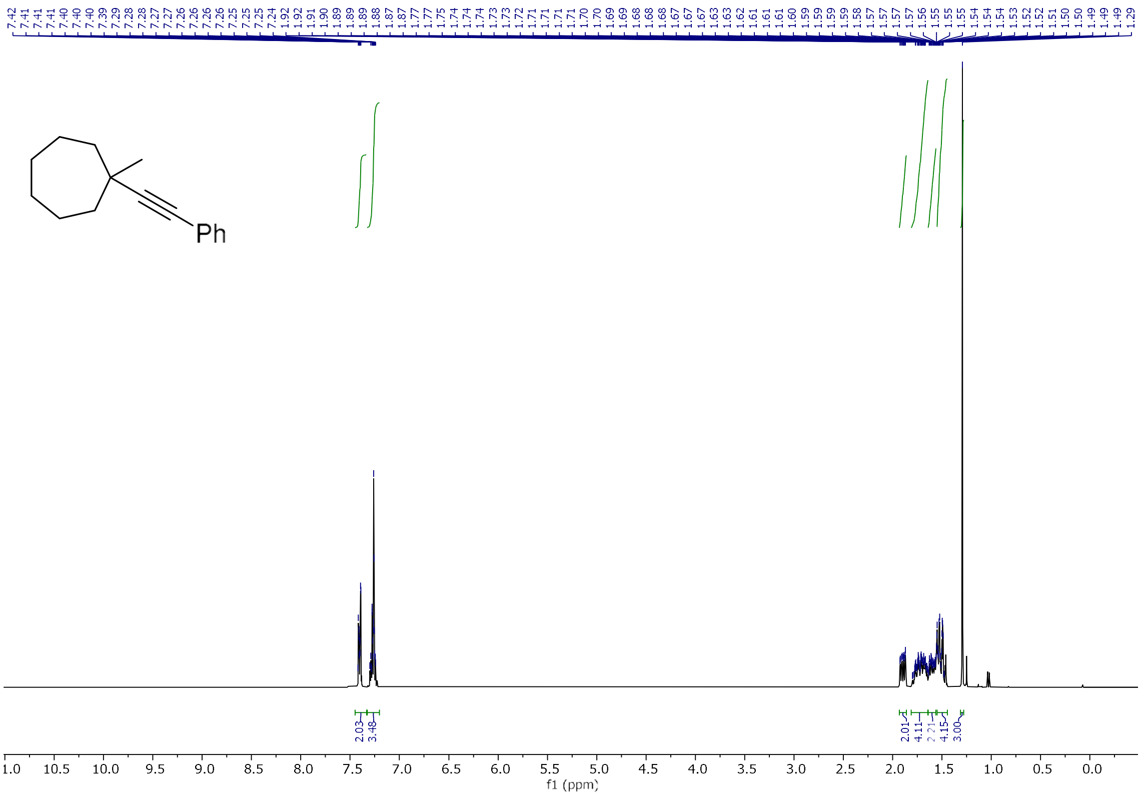

^{13}C NMR, $CDCl_3$, 400 MHz

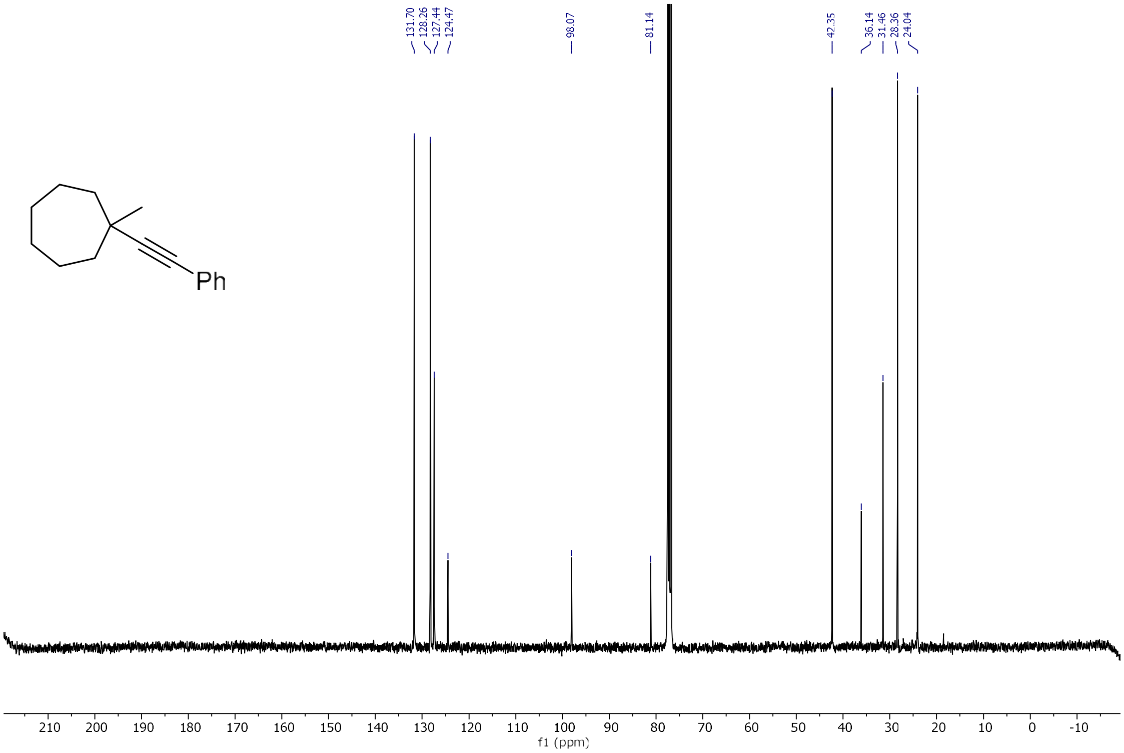

Compound **4j**

^{1}H NMR, $CDCl_3$, 400 MHz

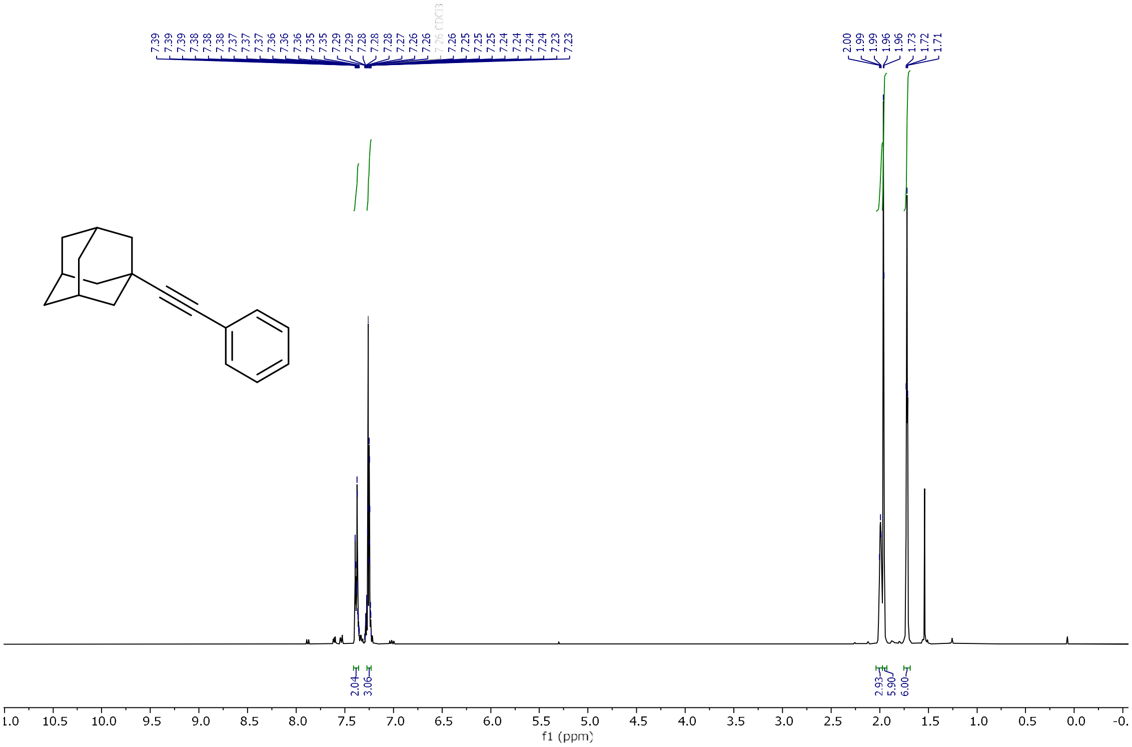

^{13}C NMR, $CDCl_3$ (with 1V% TMS), 101 MHz

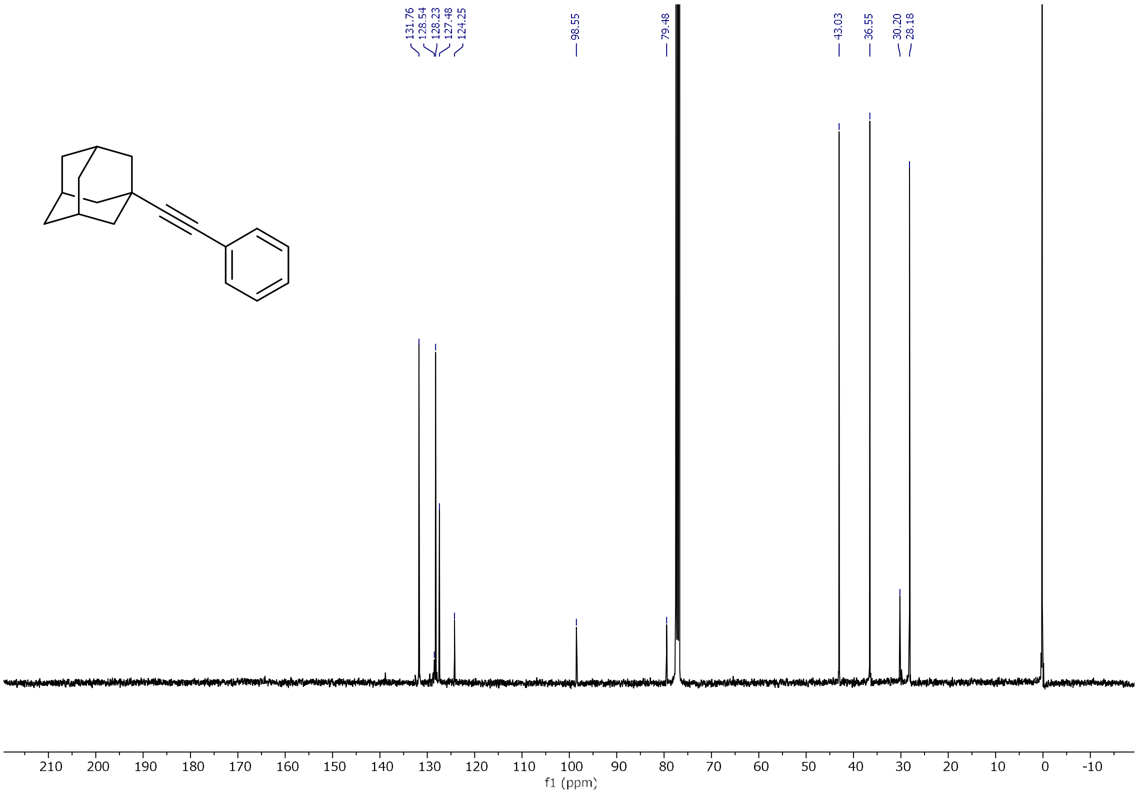

Compound **4k**

^{1}H NMR, $CDCl_3$, 400 MHz

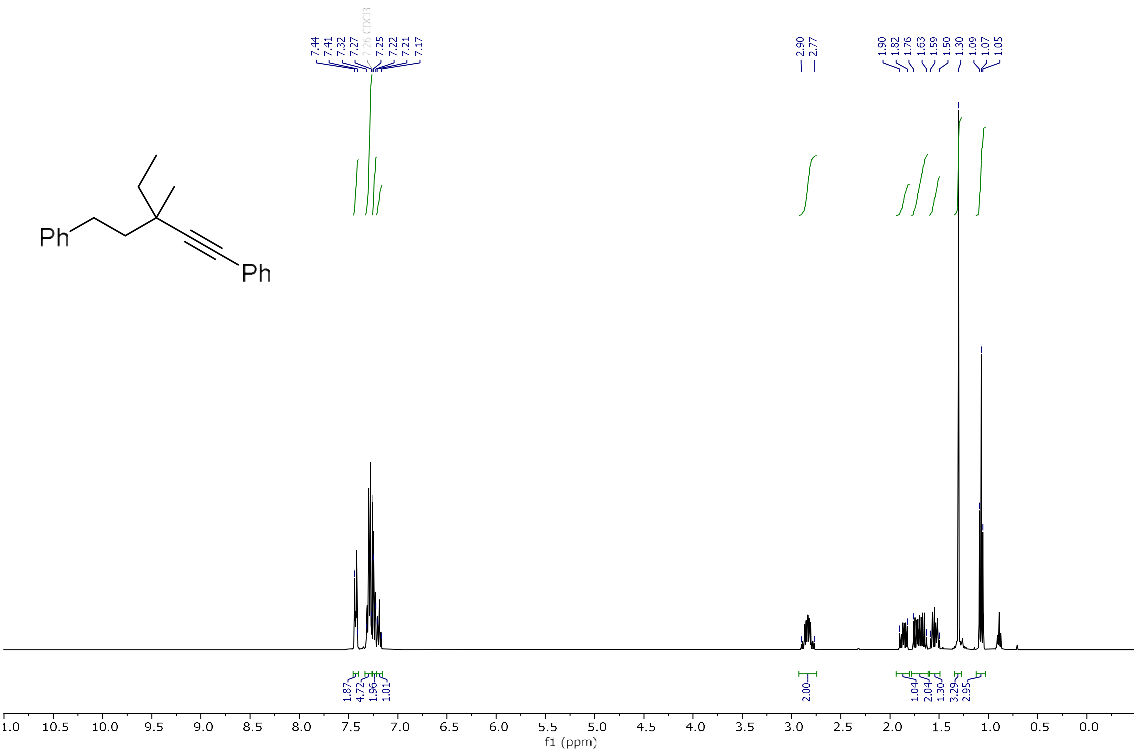

^{13}C NMR, $CDCl_3$, 101 MHz

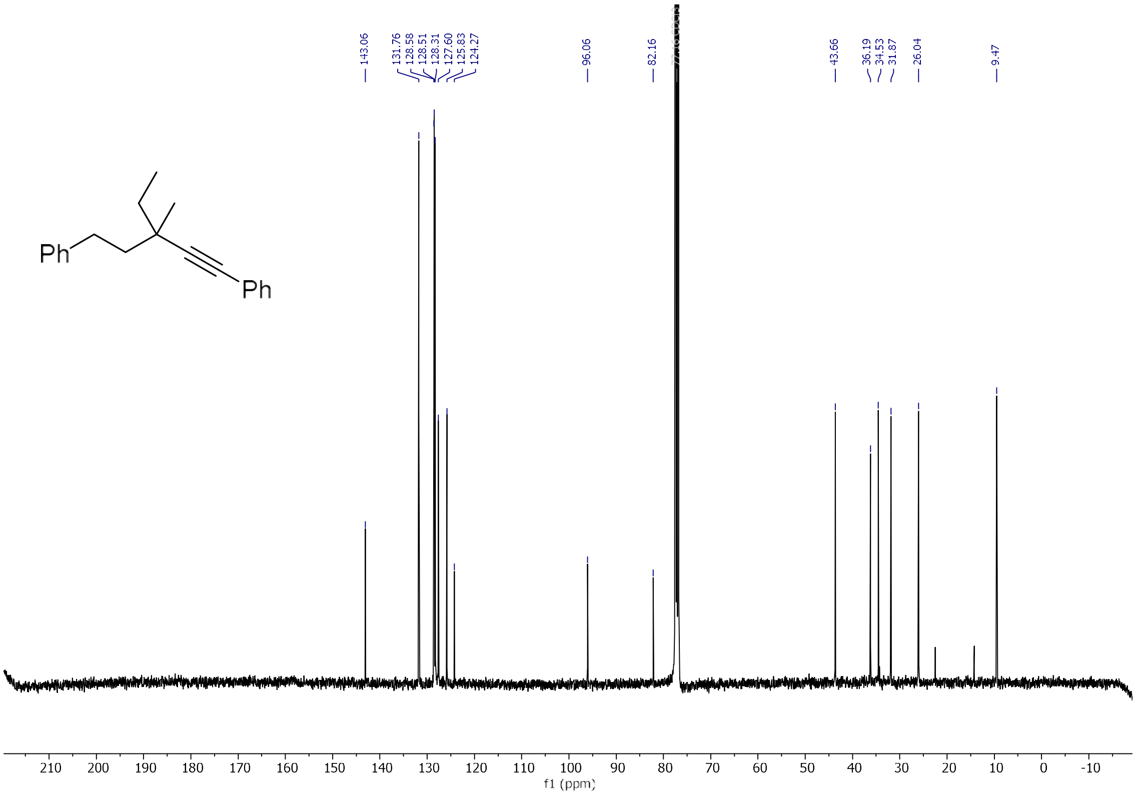

Compound **4l**

^1H NMR, $CDCl_3$, 400 MHz

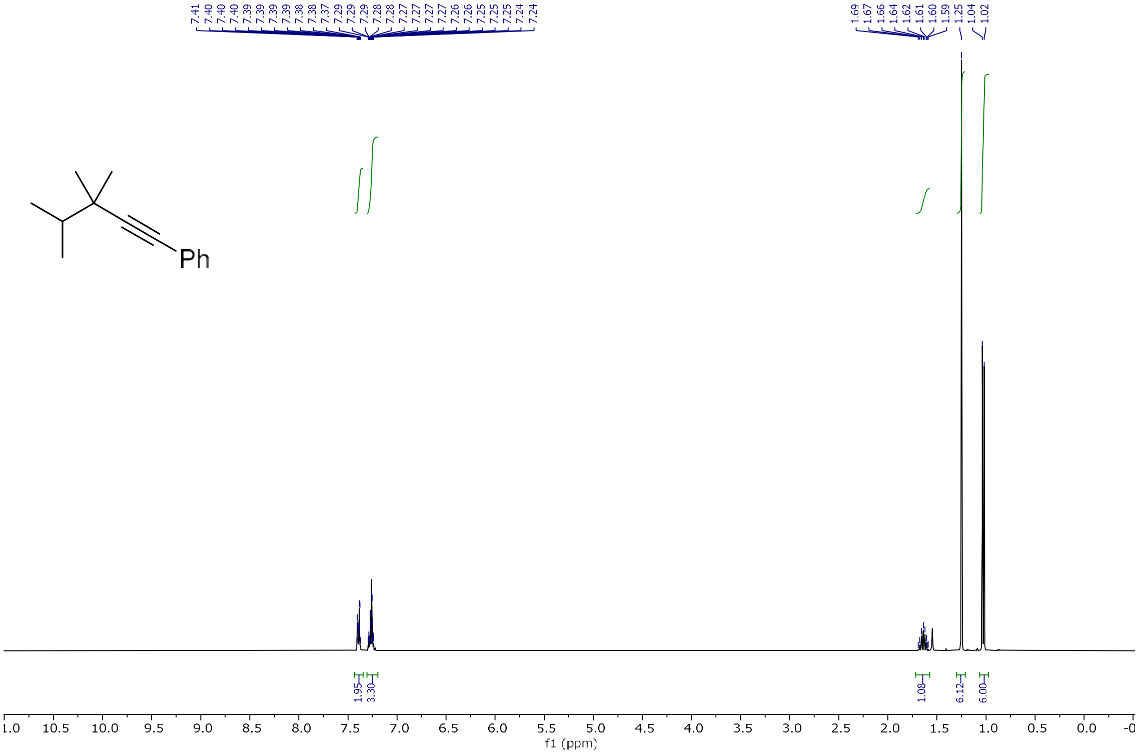

^{13}C NMR, $CDCl_3$, 101 MHz

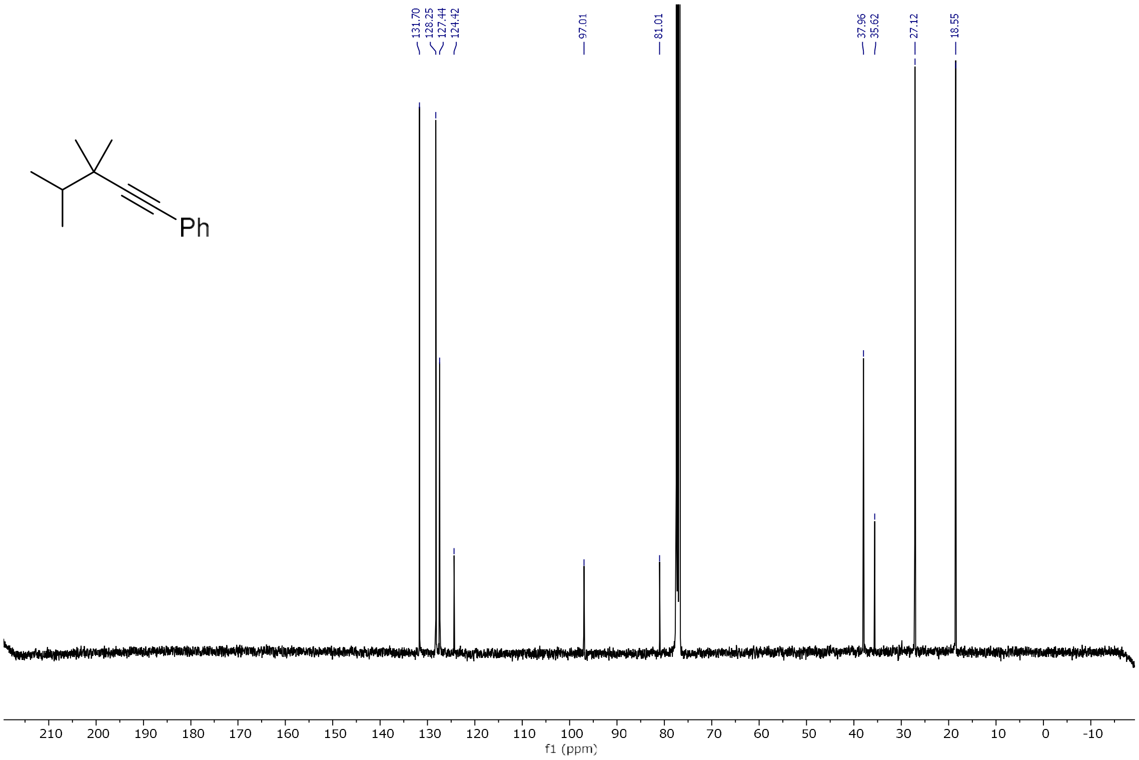

Compound **4m**

^{1}H NMR, $CDCl_3$, 400 MHz

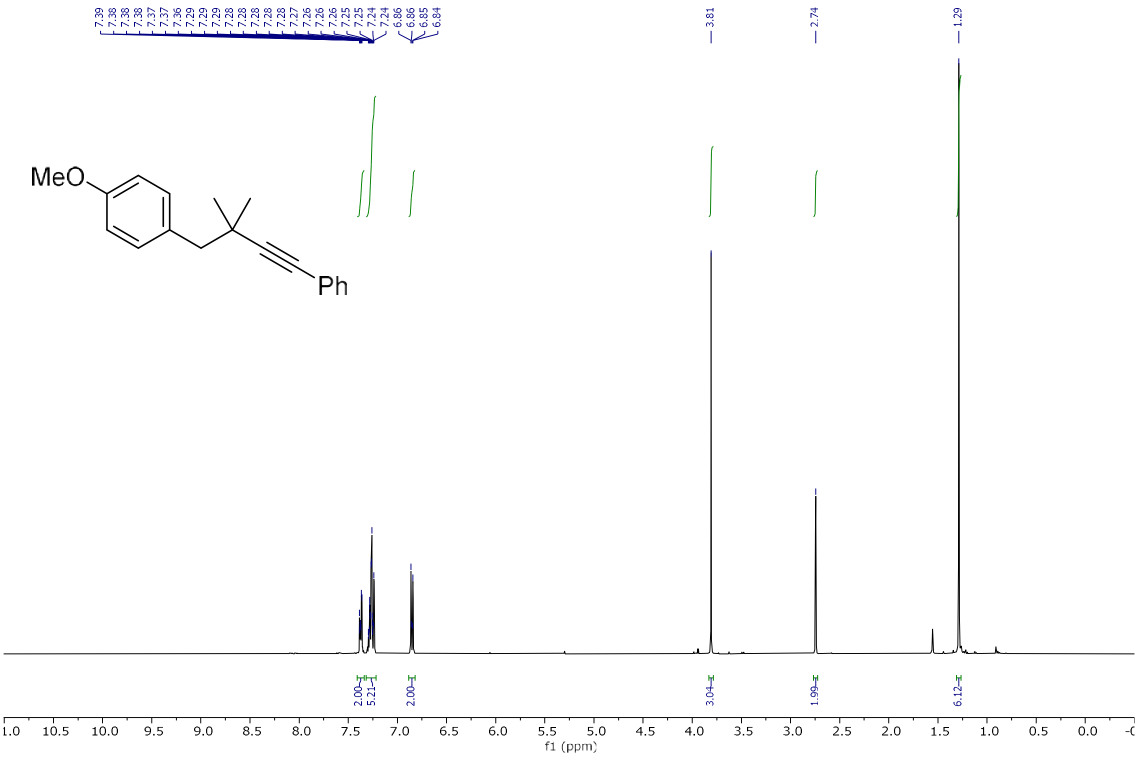

^{13}C NMR, $CDCl_3$, 101 MHz

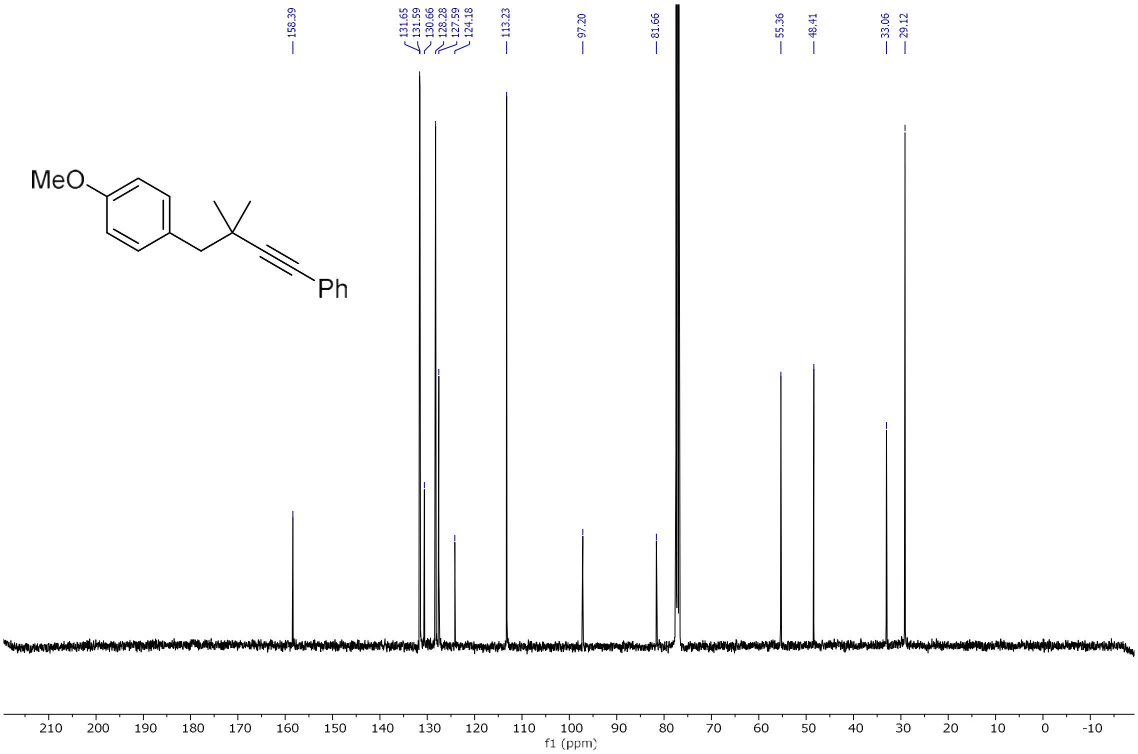

Compound **4n**

^{1}H NMR, $CDCl_3$, 400 MHz

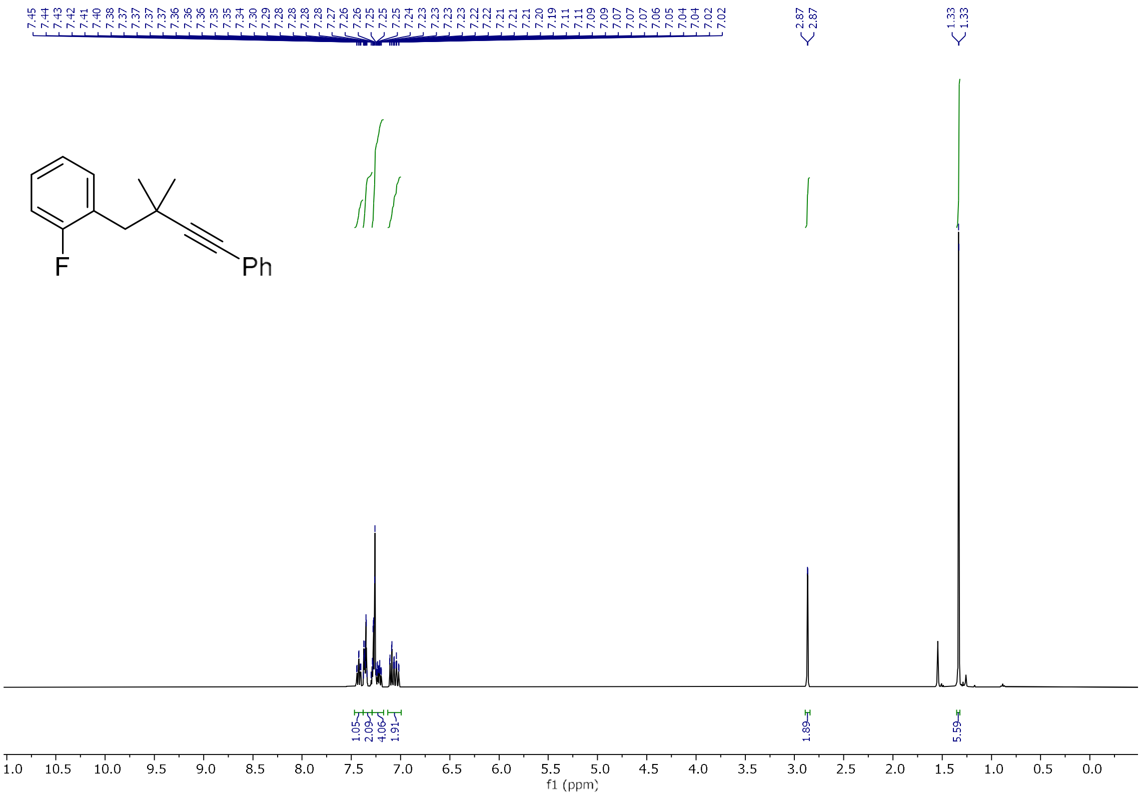

^{13}C NMR, $CDCl_3$, 101 MHz

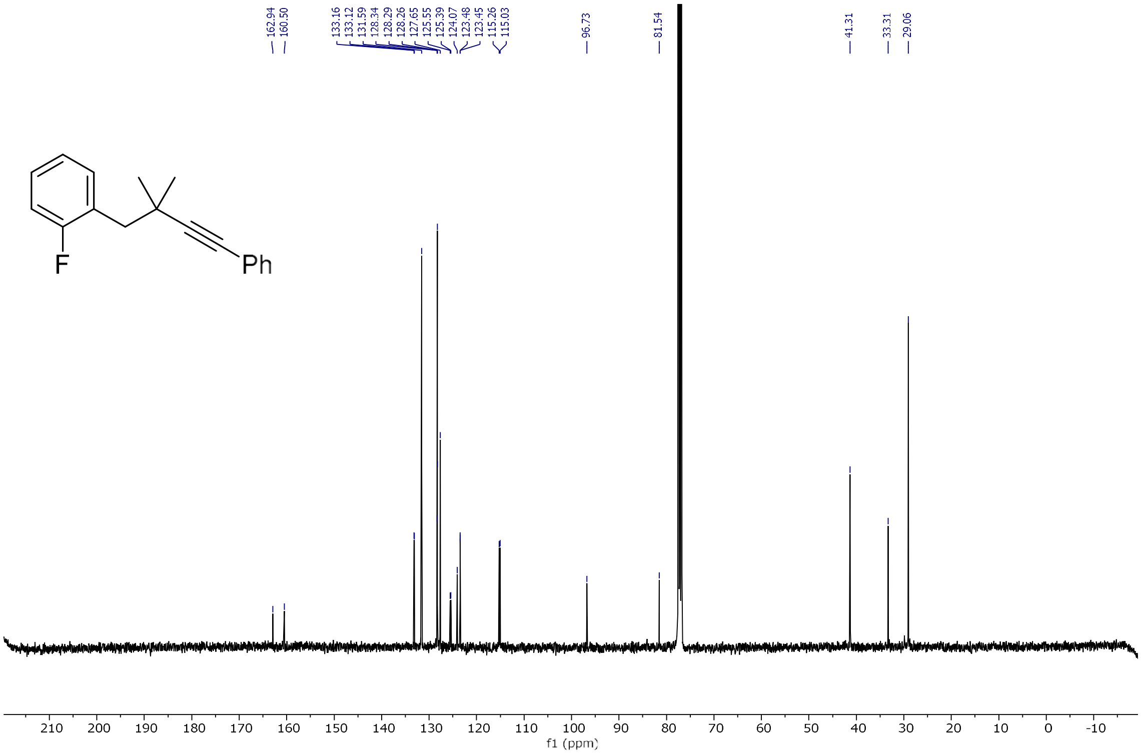

^{19}F NMR, $CDCl_3$, 376 MHz

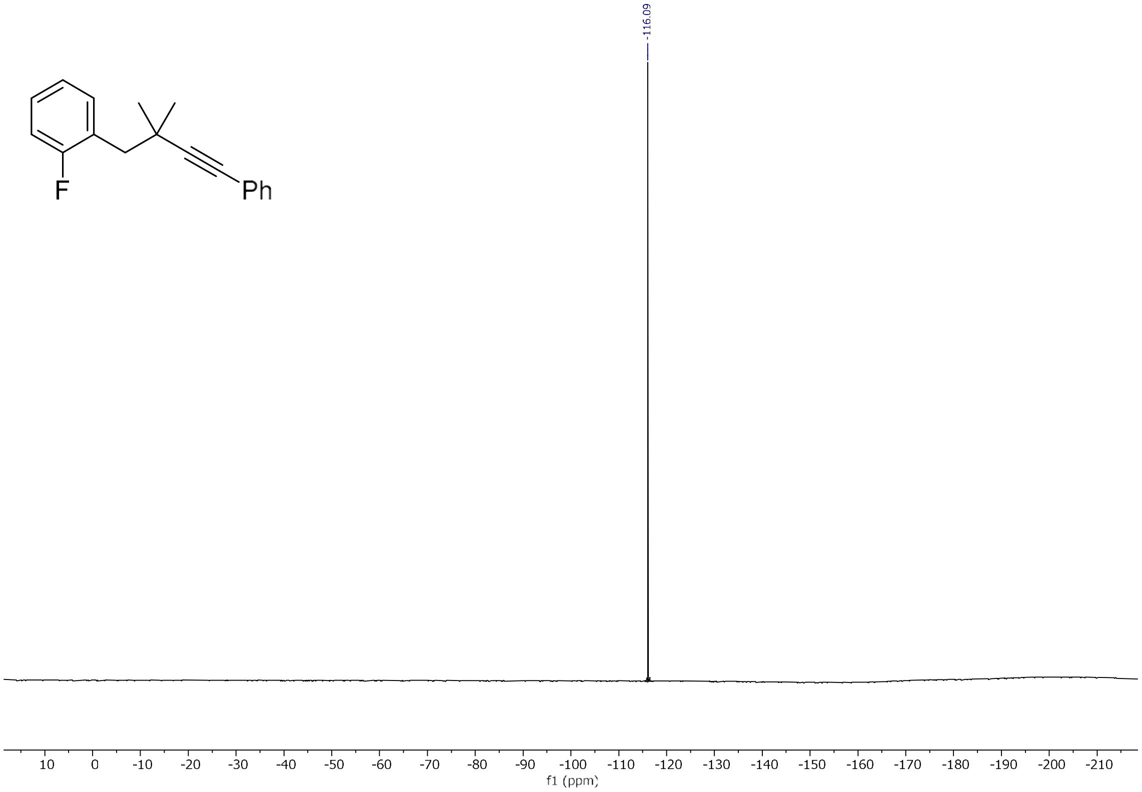

Compound **4o**

^{1}H NMR, $CDCl_3$, 400 MHz

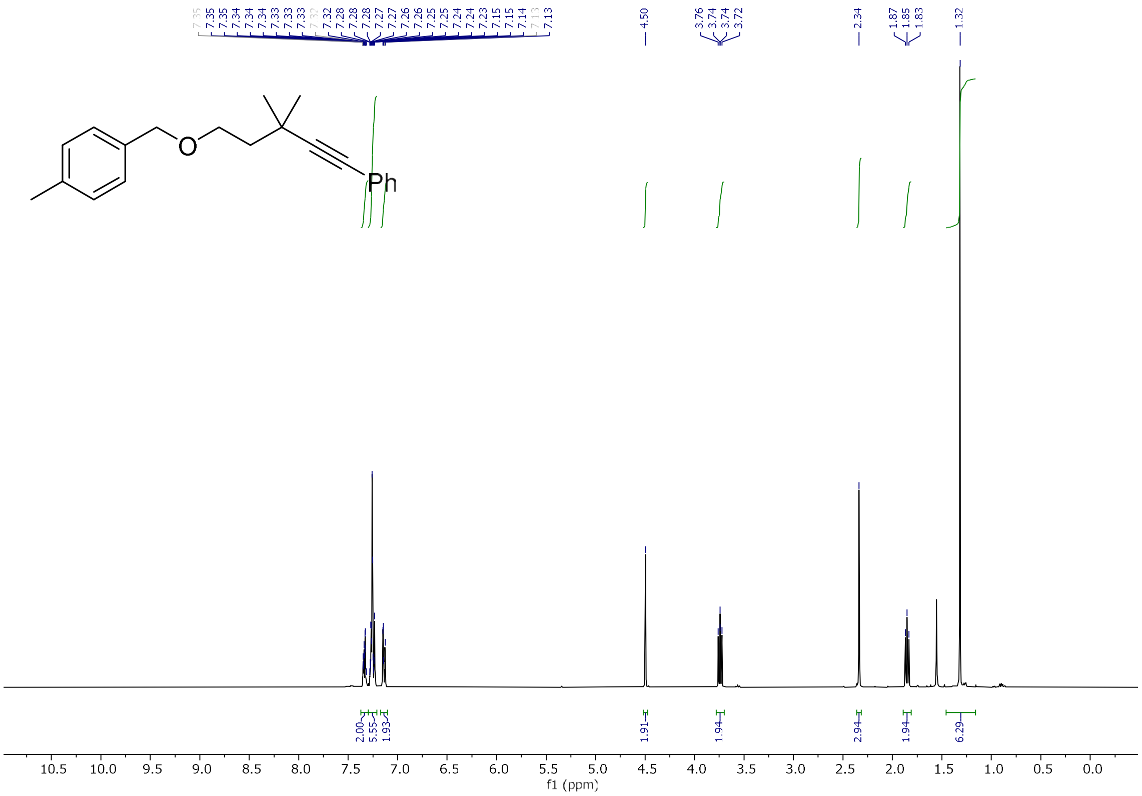

^{13}C NMR, $CDCl_3$, 101 MHz

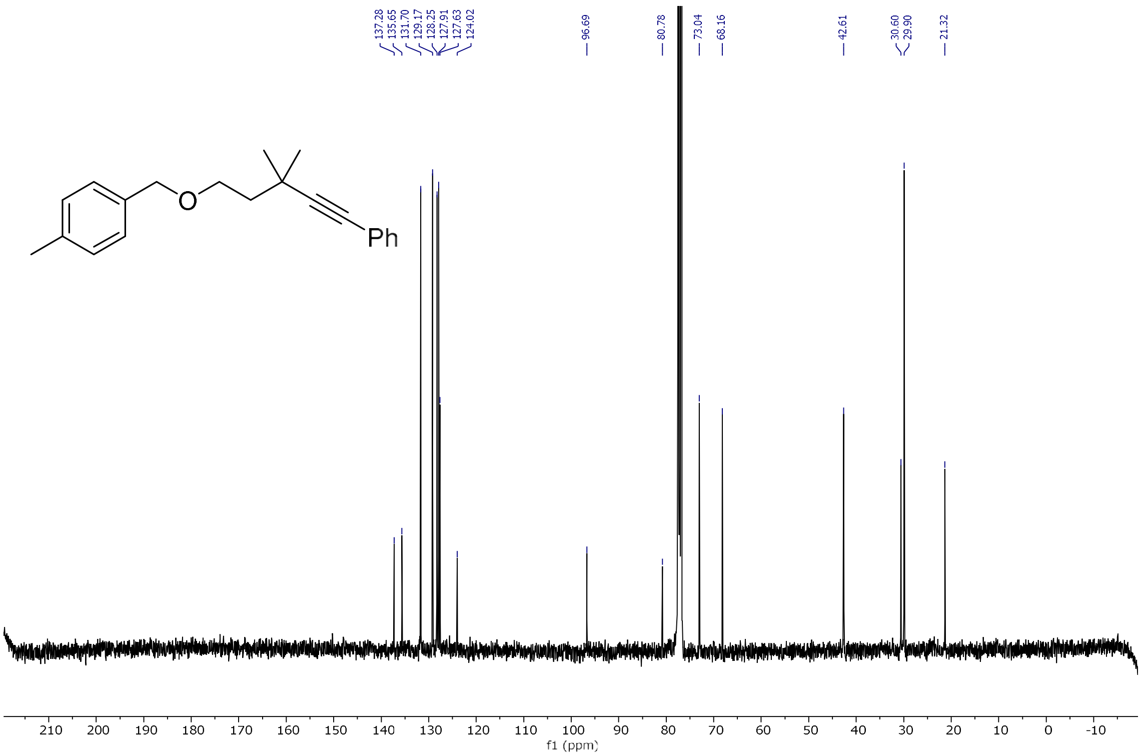

Compound **4p**

^{1}H NMR, $CDCl_3$, 400 MHz

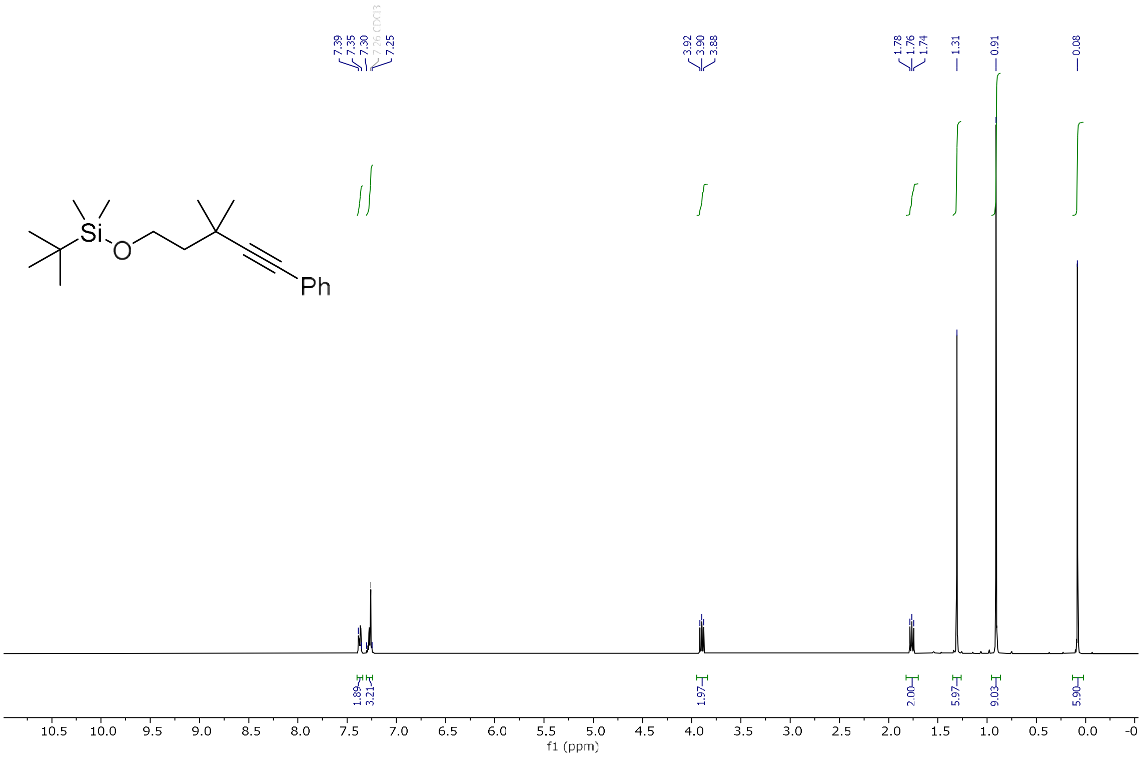

^{13}C NMR, $CDCl_3$, 101 MHz

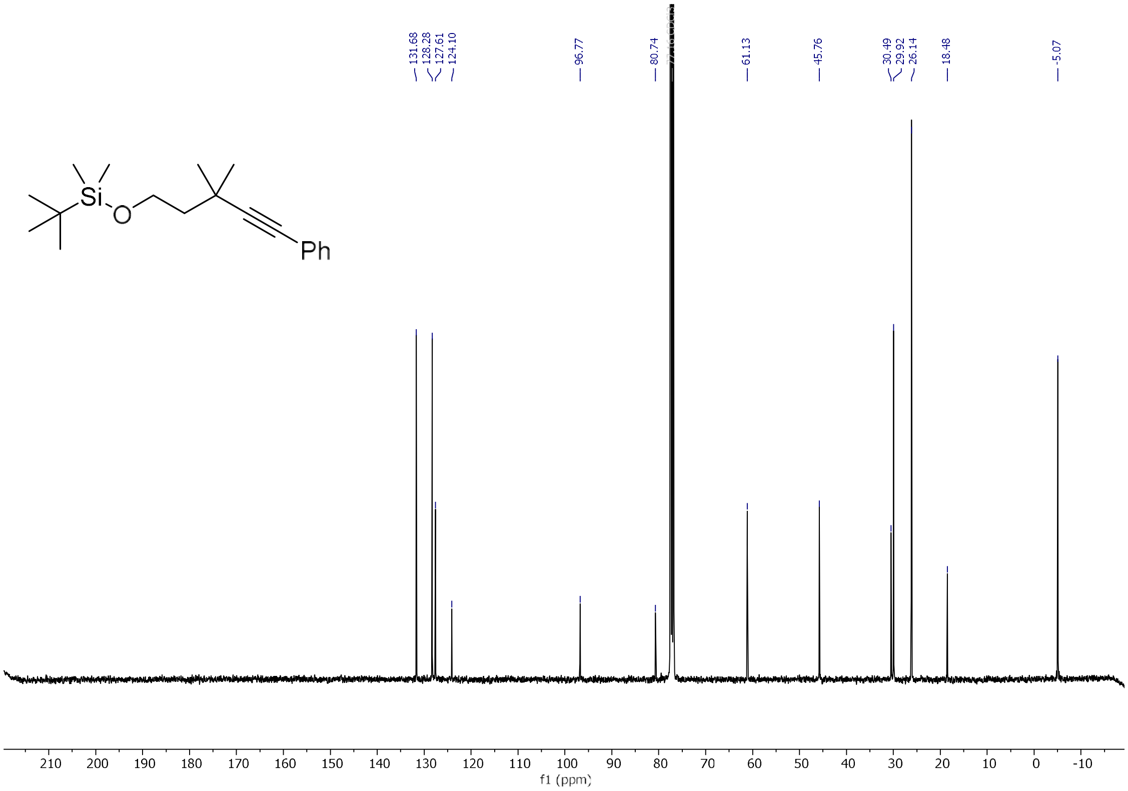

Compound **4q**

^{1}H NMR, $CDCl_3$, 400 MHz

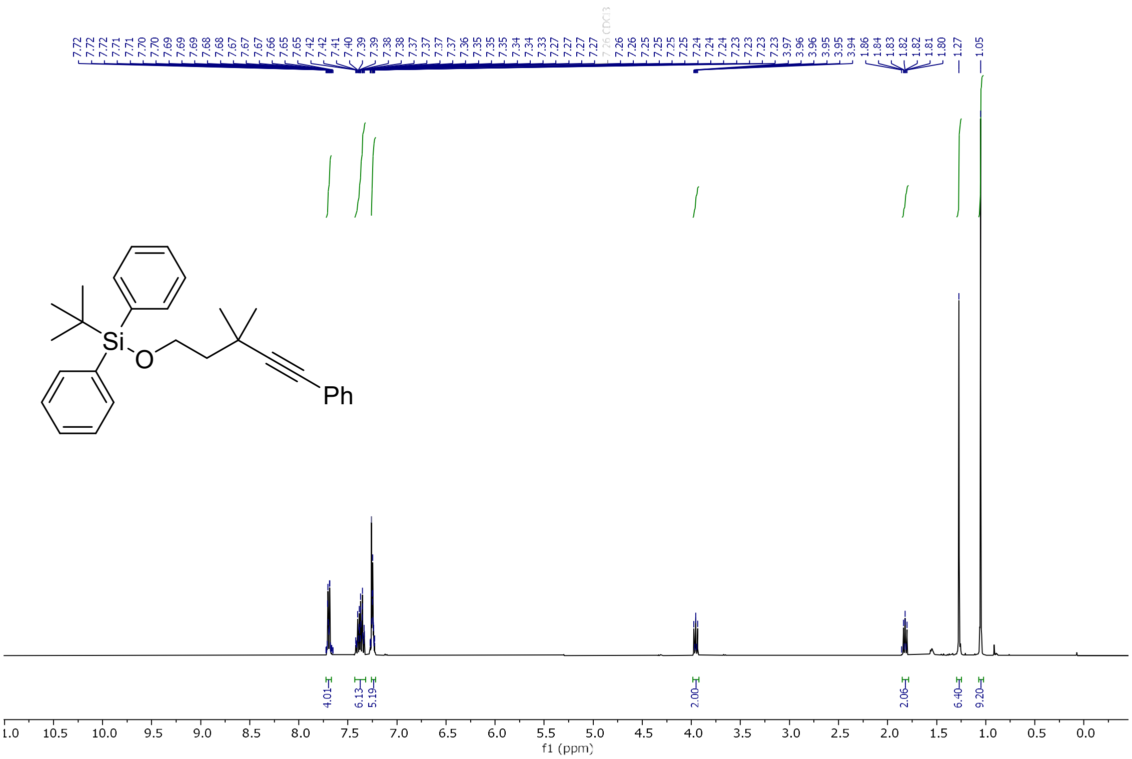

^{13}C NMR, $CDCl_3$, 101 MHz

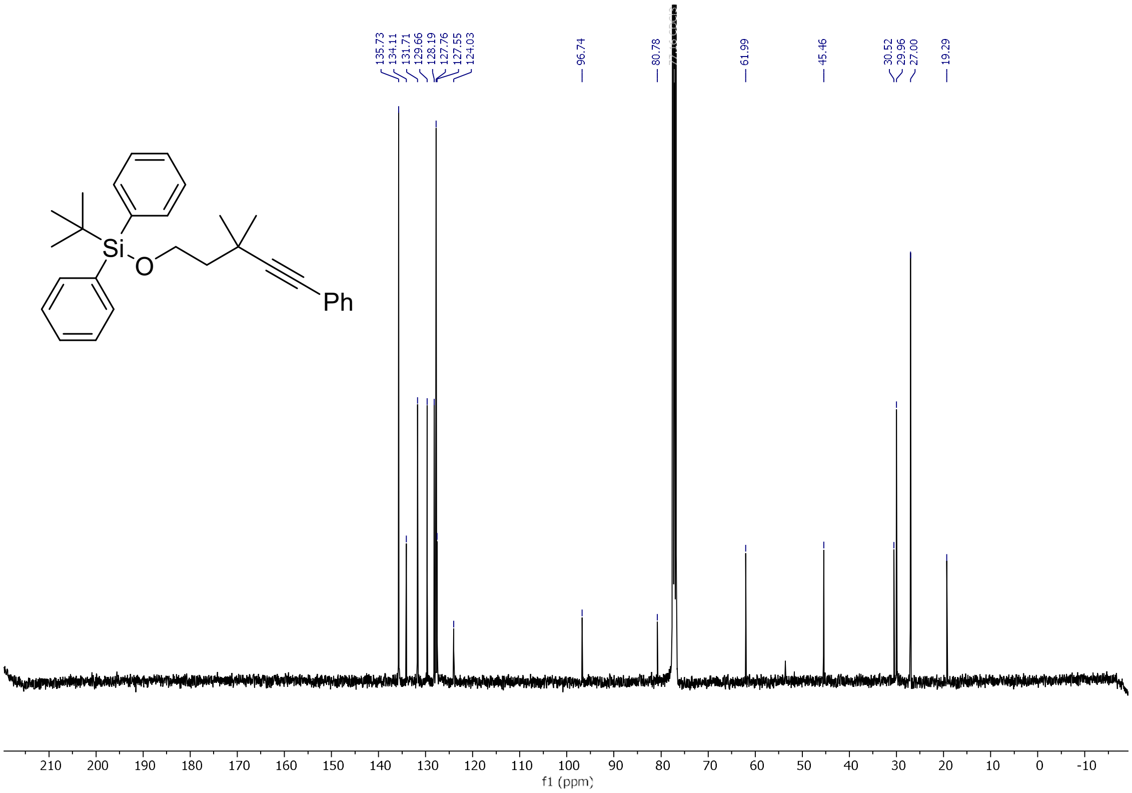

Compound **4r**

^{1}H NMR, $CDCl_3$, 400 MHz

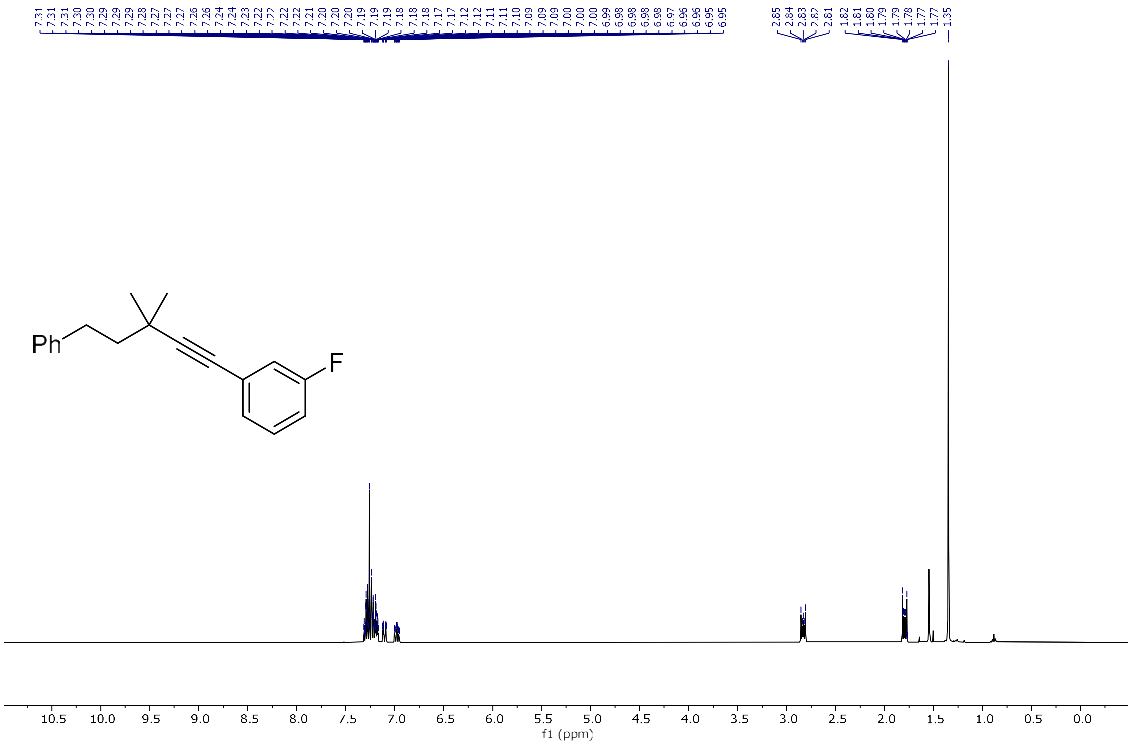

^{13}C NMR, $CDCl_3$, 101 MHz

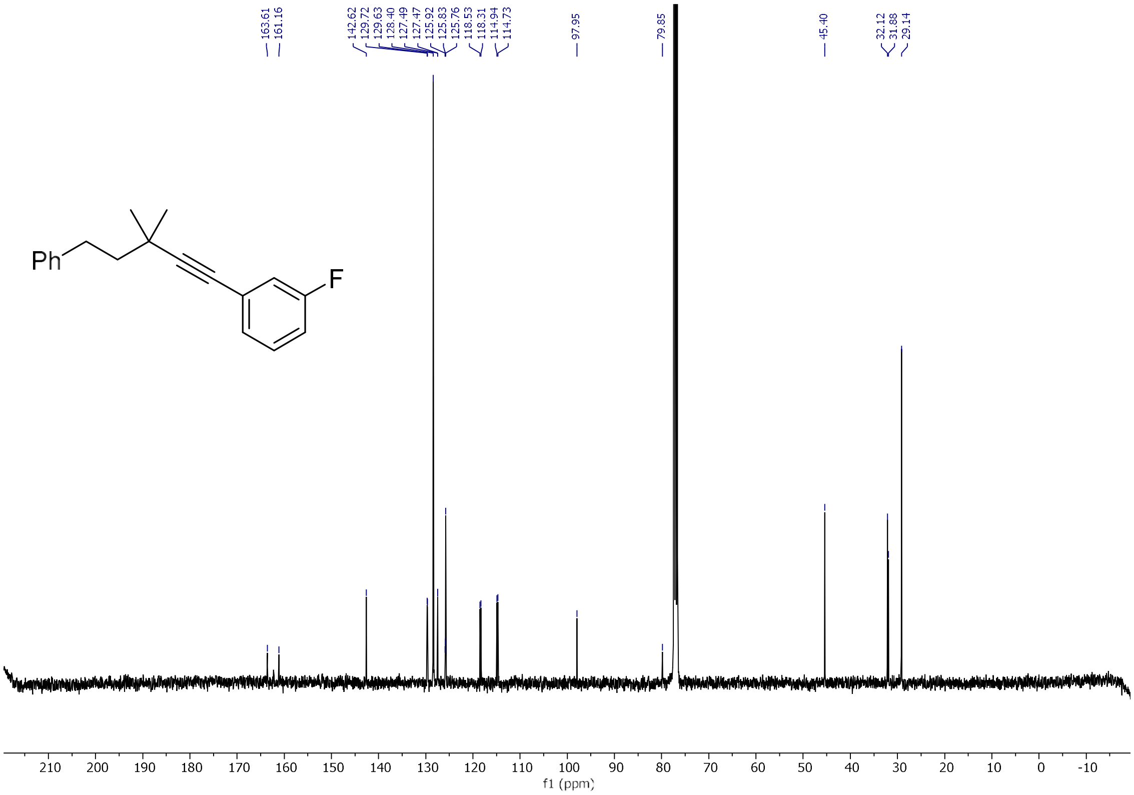

^{19}F NMR, $CDCl_3$, 376 MHz

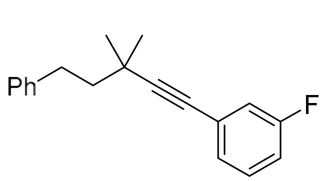

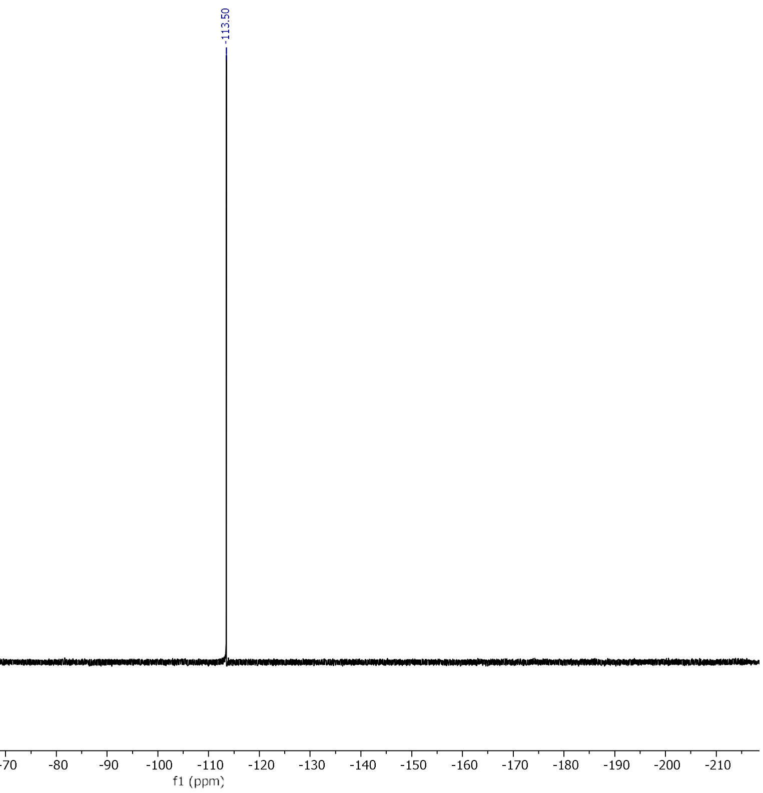

Compound **4s**

^{1}H NMR, $CDCl_3$, 400 MHz

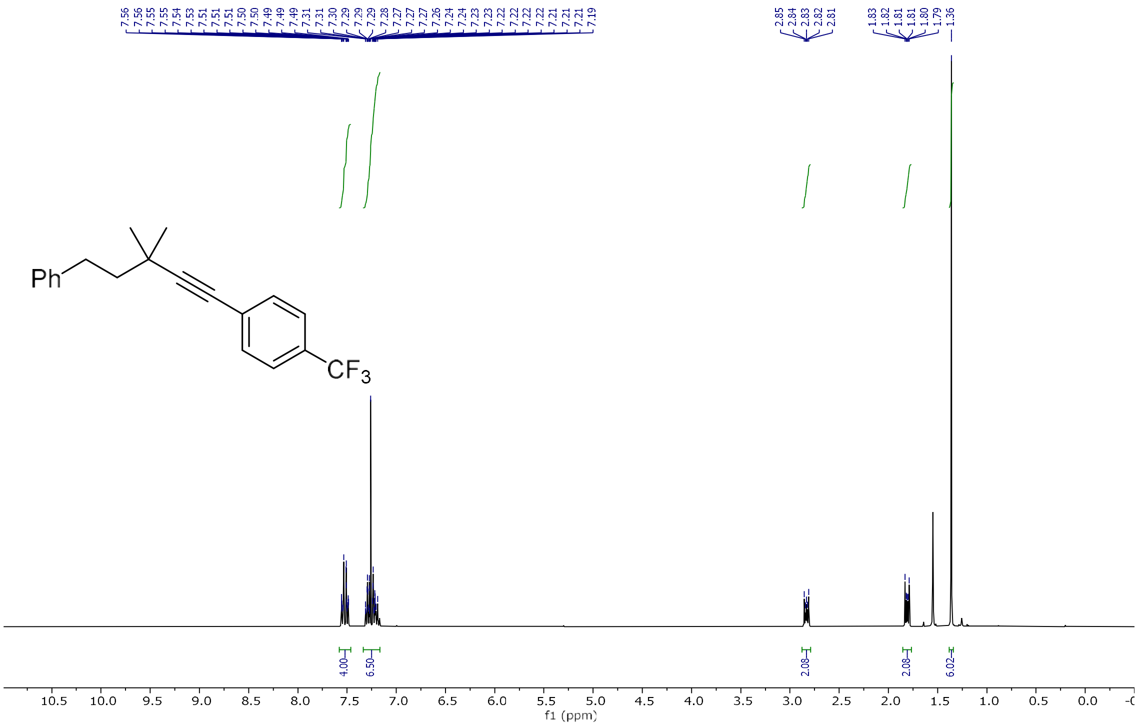

^{13}C NMR, $CDCl_3$, 101 MHz

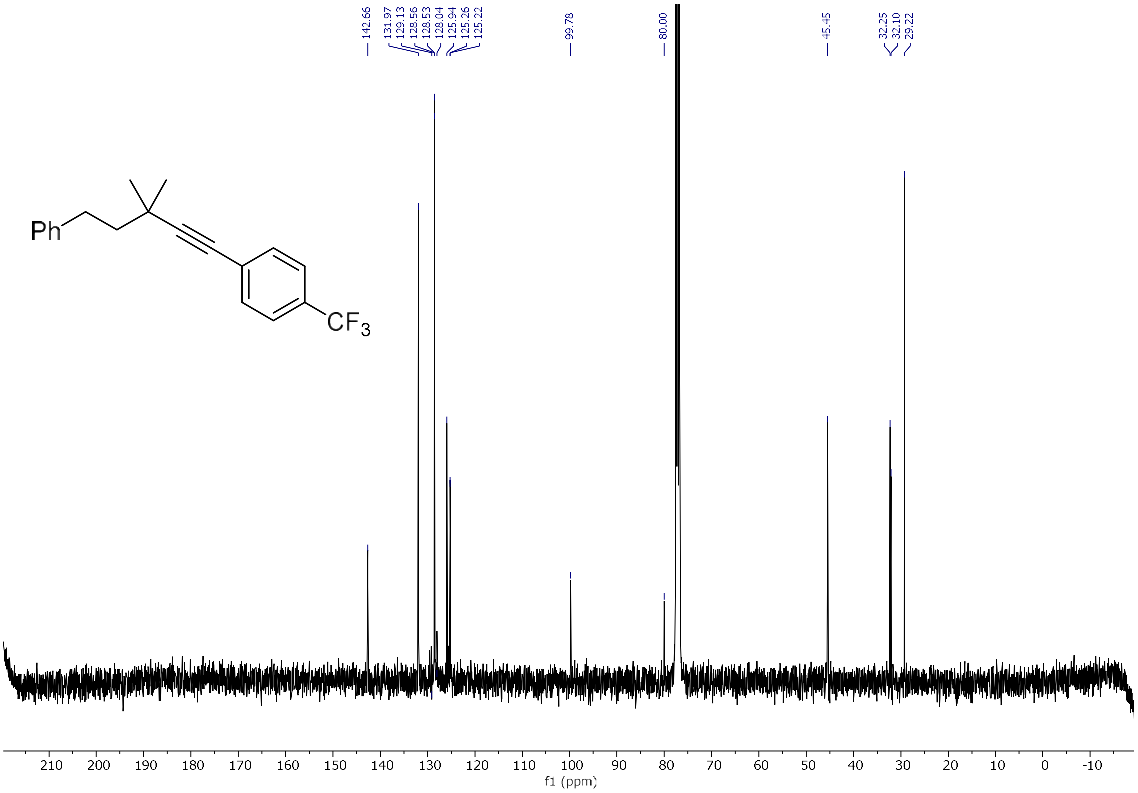

^{19}F NMR, $CDCl_3$, 376 MHz,

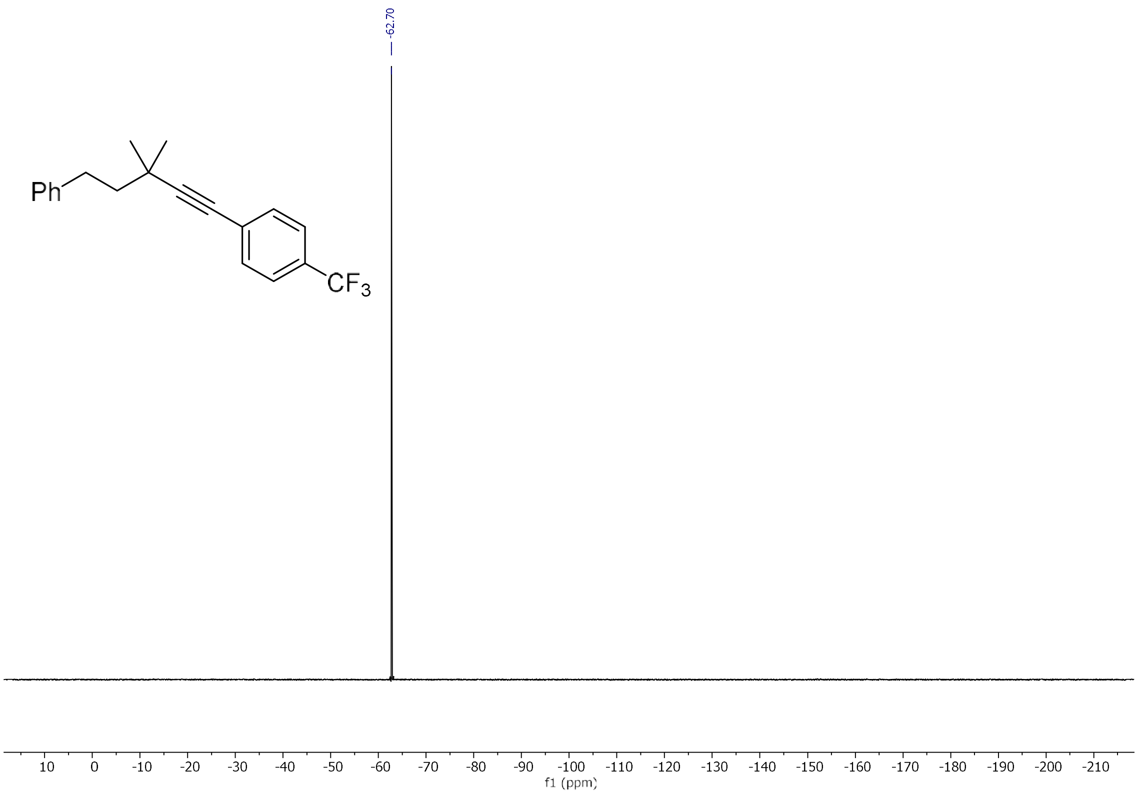

Compound **4t**

^{1}H NMR, $CDCl_3$, 400 MHz

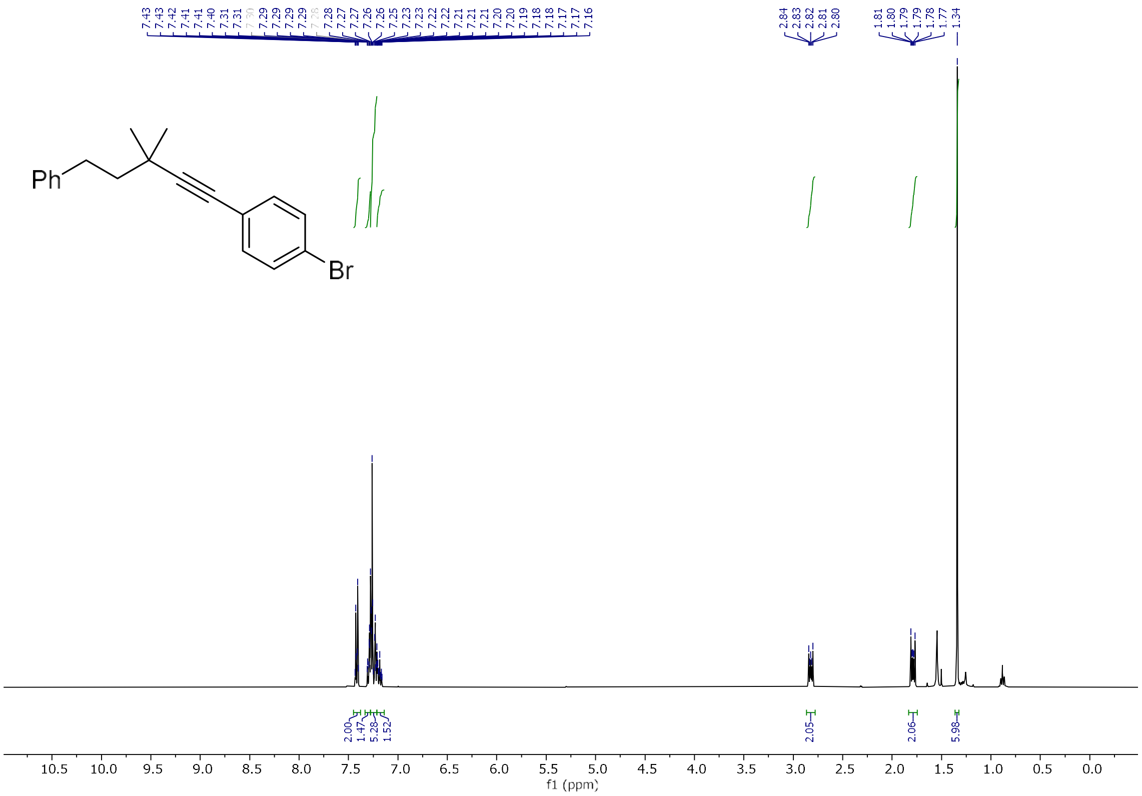

^{13}C NMR, $CDCl_3$, 101 MHz

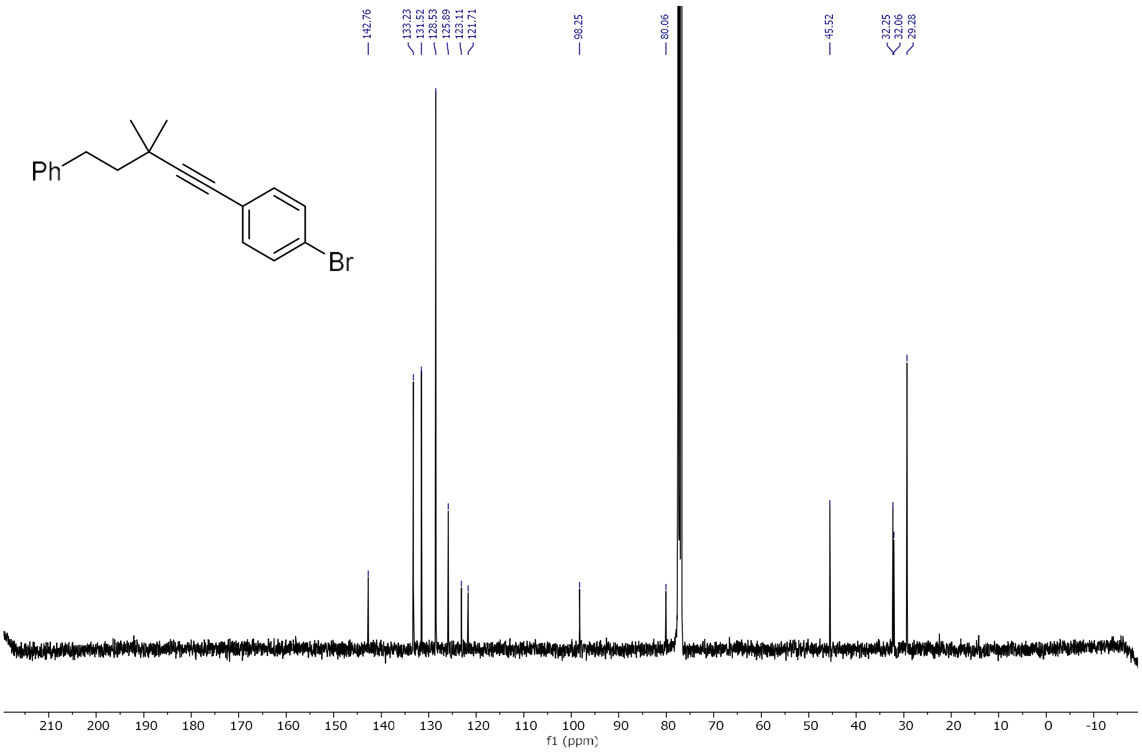

Compound **4u**

^{1}H NMR, $CDCl_3$, 400 MHz

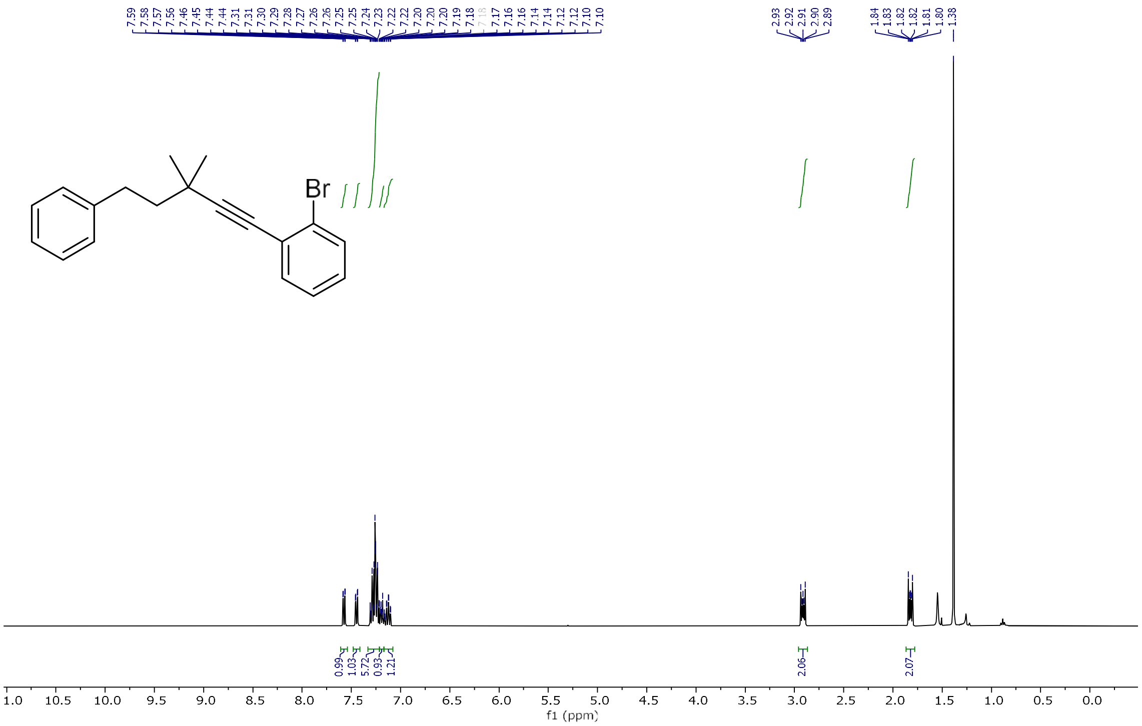

^{13}C NMR, $CDCl_3$, 101 MHz

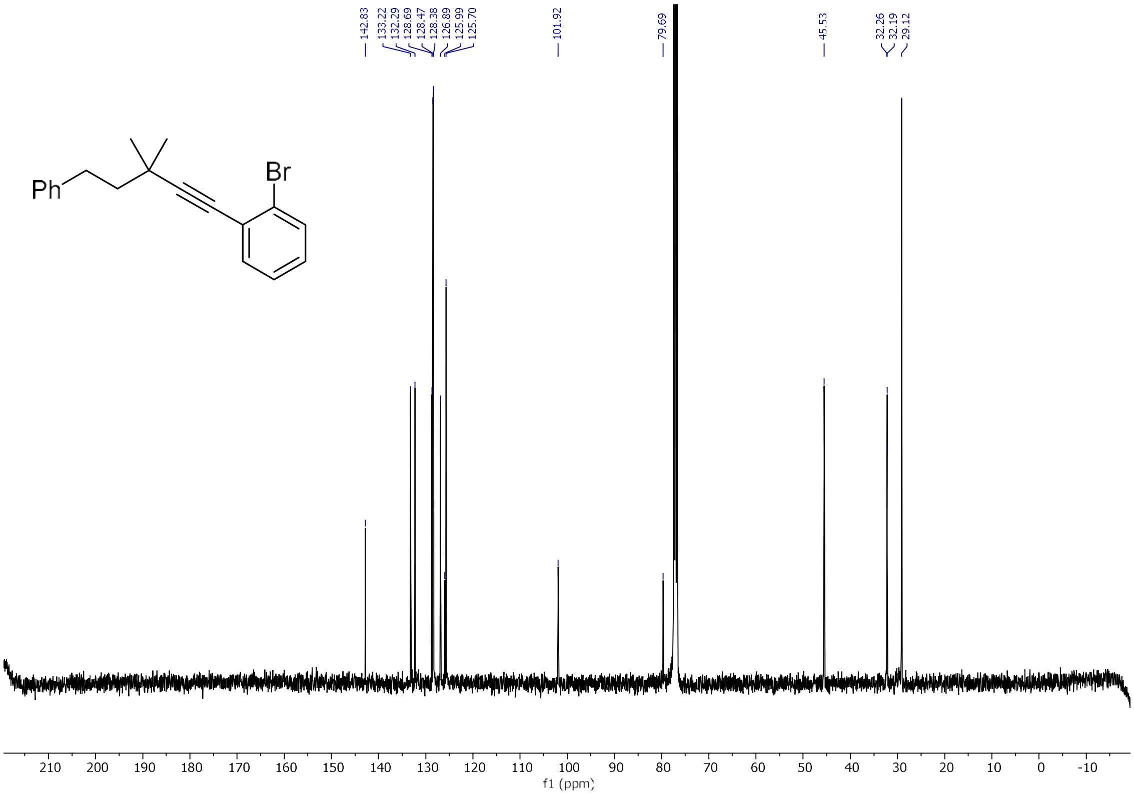

Compound **4v**

^{1}H NMR, $CDCl_3$, 400 MHz

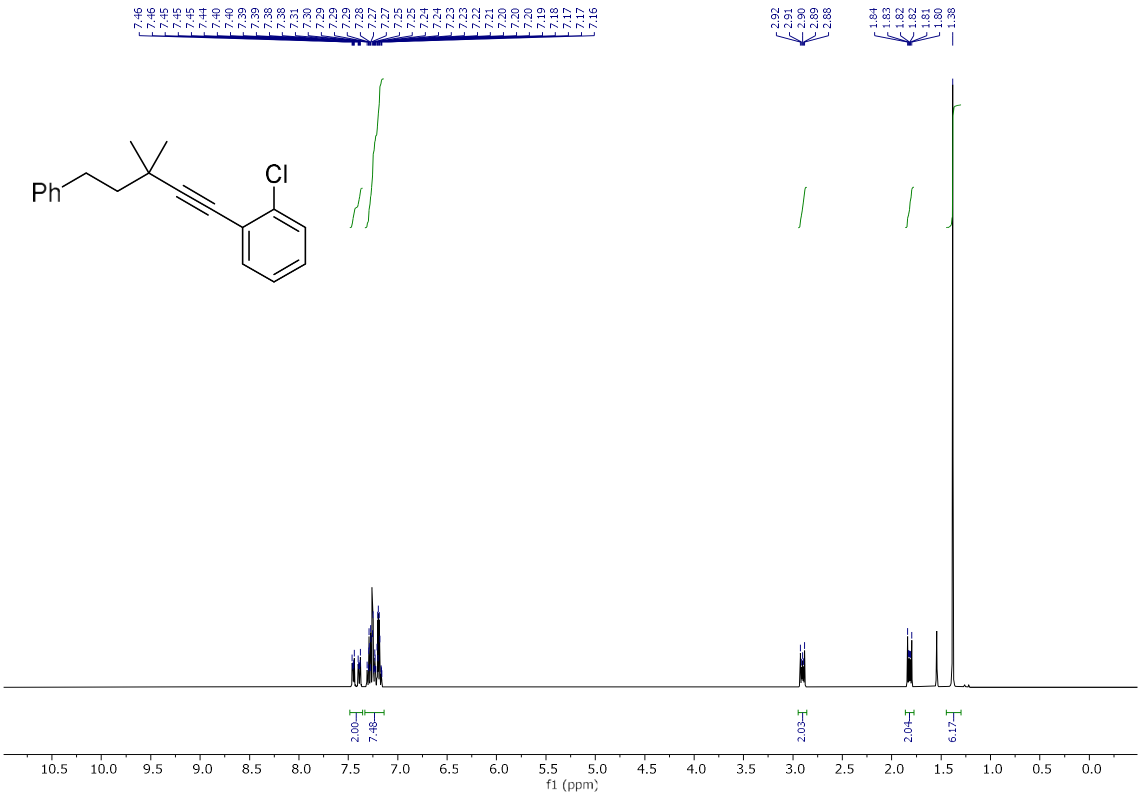

^{13}C NMR, $CDCl_3$, 400 MHz

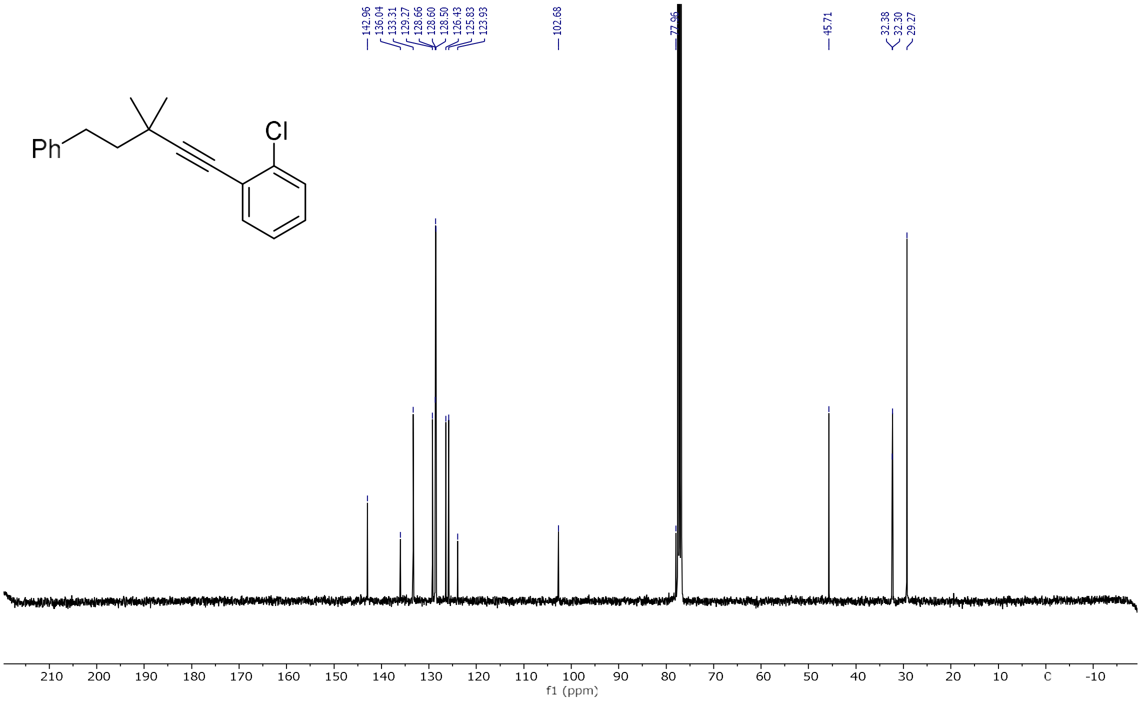

Compound **4w**

^{1}H NMR, $CDCl_3$,400 MHz

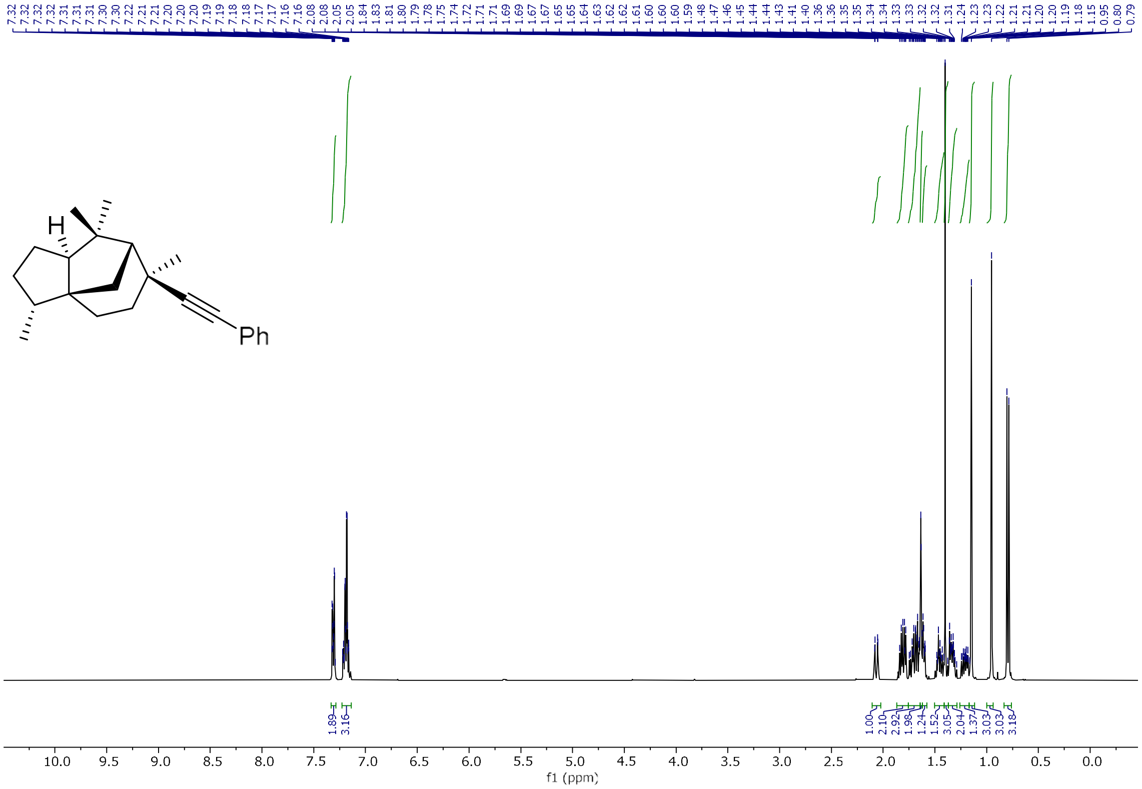

^{13}C NMR, $CDCl_3$, 101 MHz

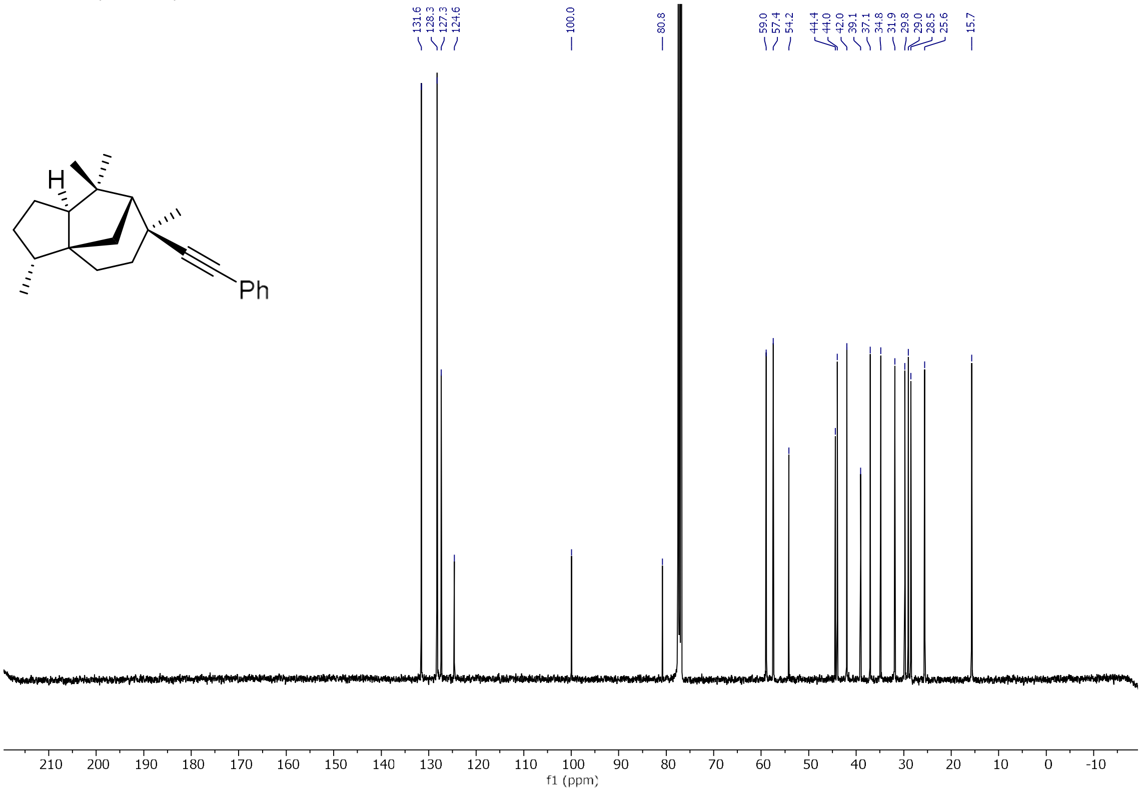

COSY

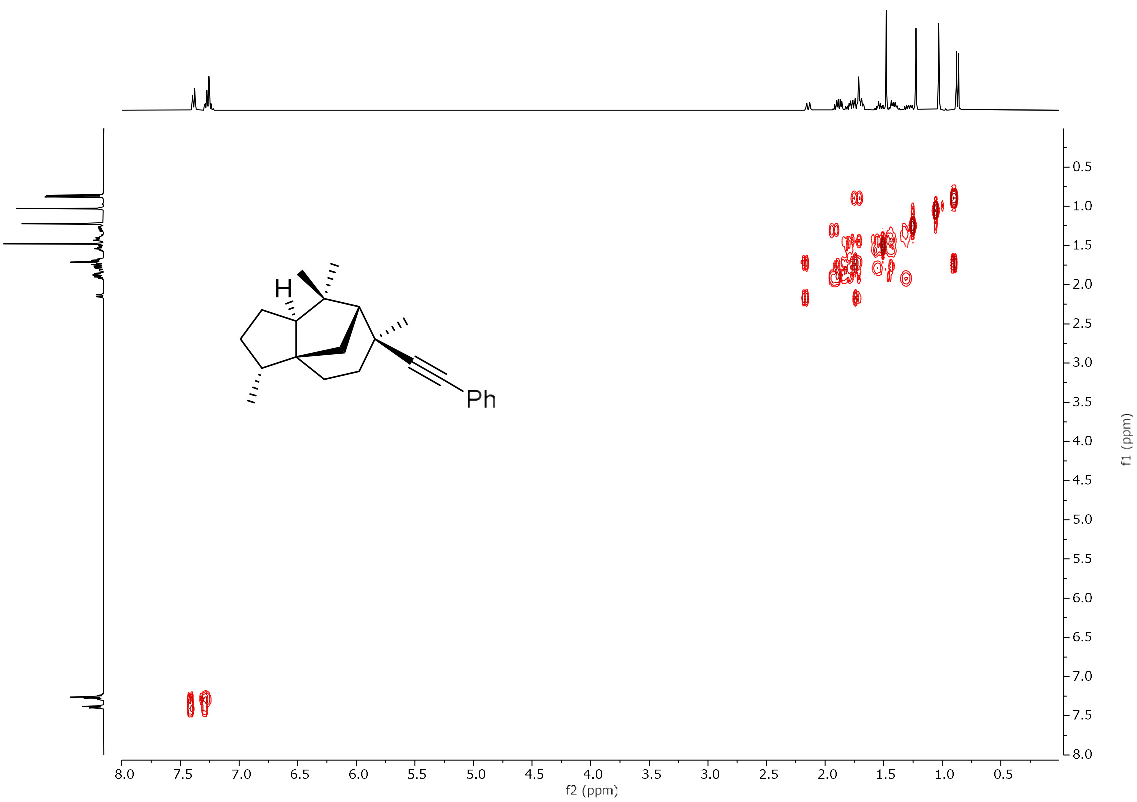

HSQC (O1P = 55 ppm, SW =80 ppm)

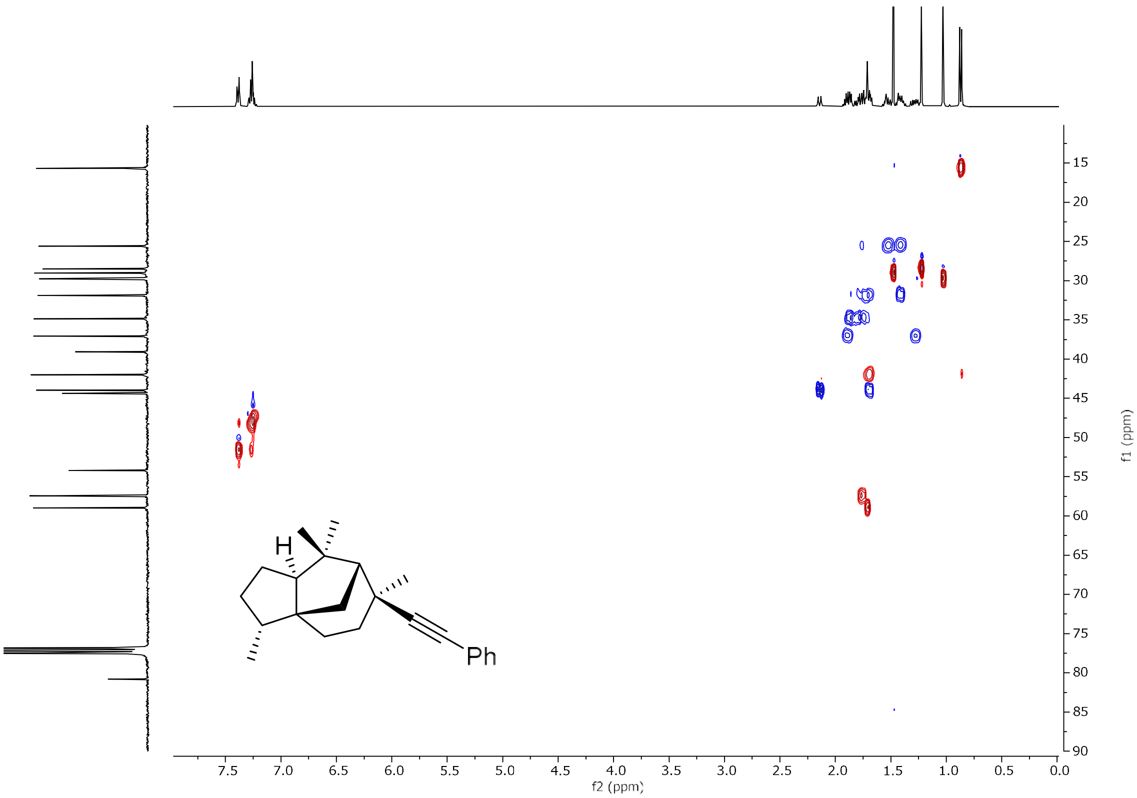

HMBC (O1P = 55 ppm; SW = 80 ppm)

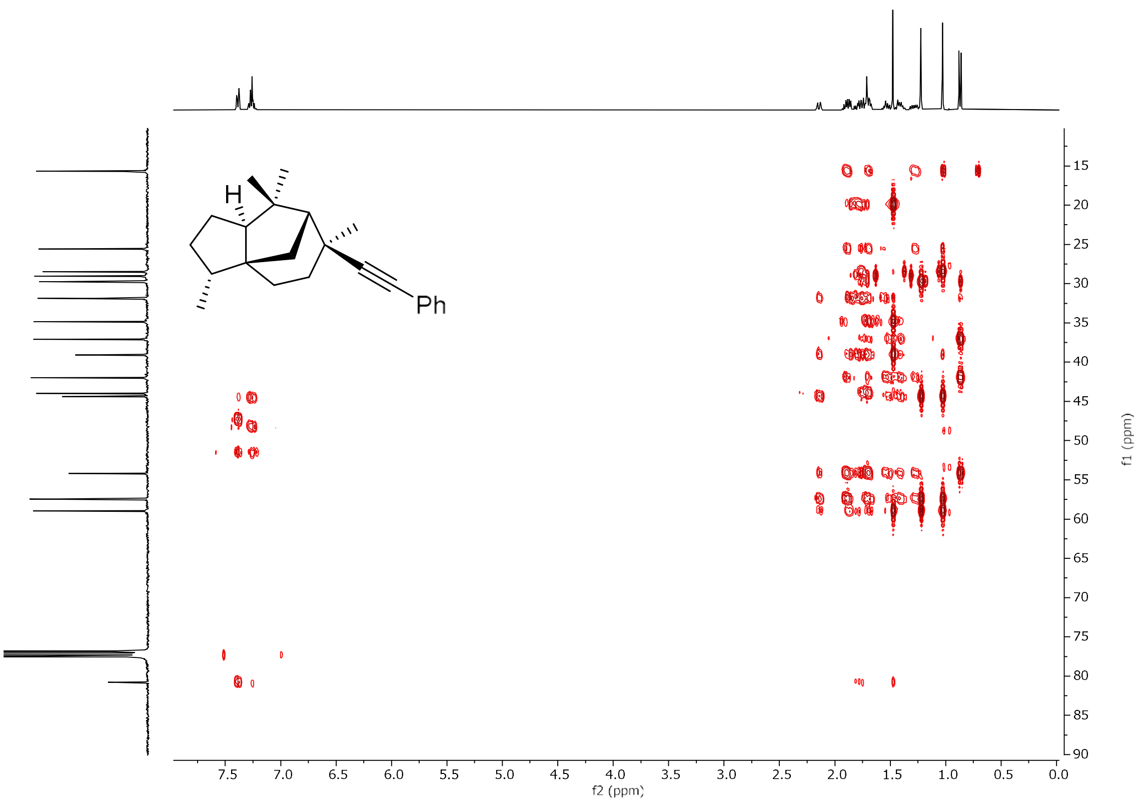

NOESY (O1P = 4 ppm, SW = 8 ppm)

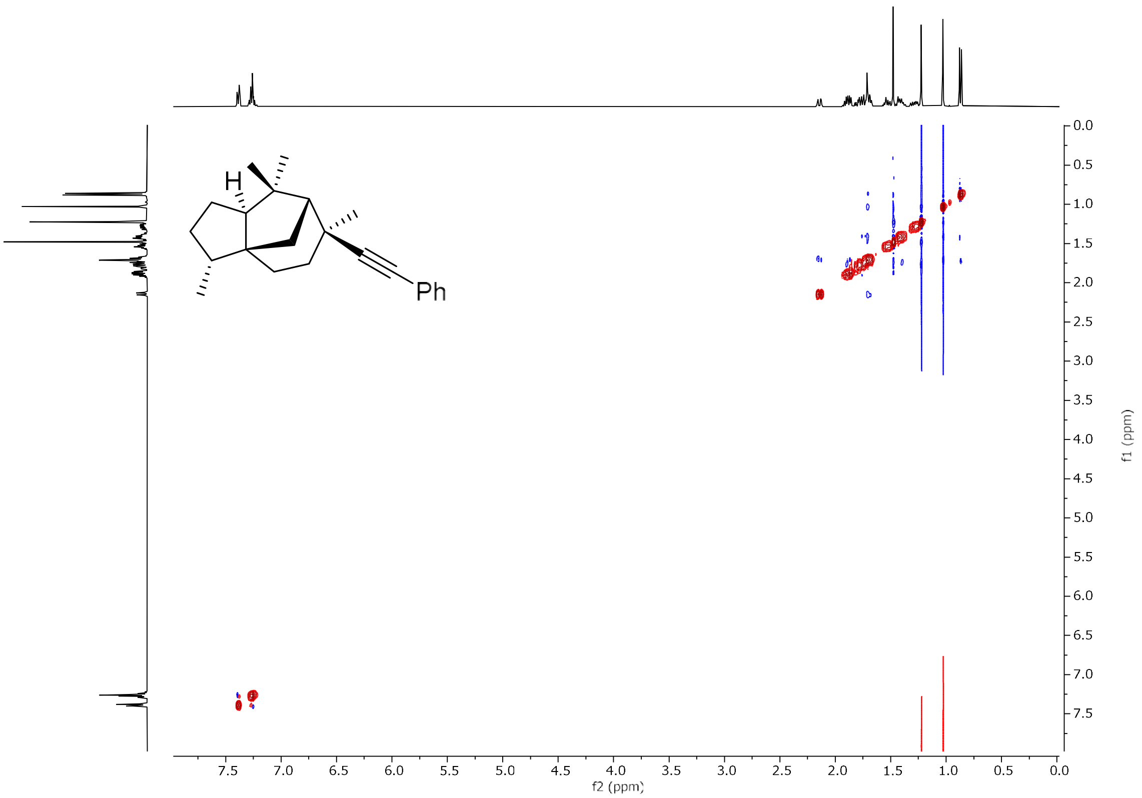

Compound **4x**

^{1}H NMR, $CDCl_3$, 400 MHz

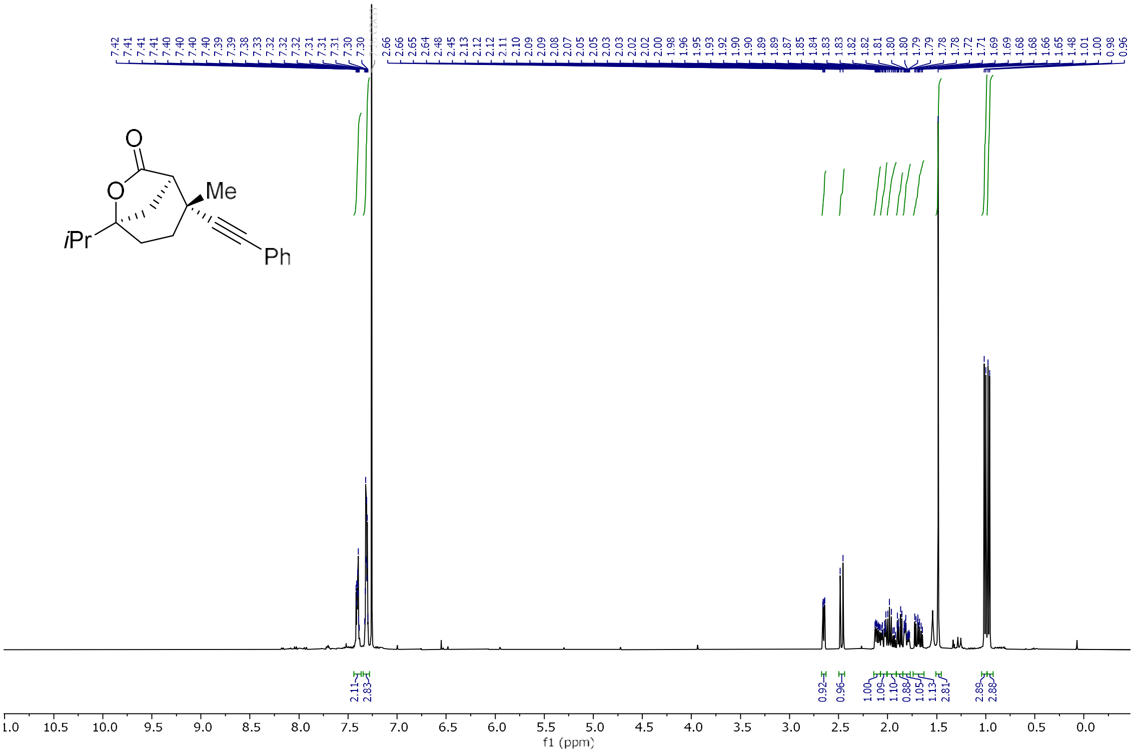

^{13}C NMR, $CDCl_3$, 101 MHz

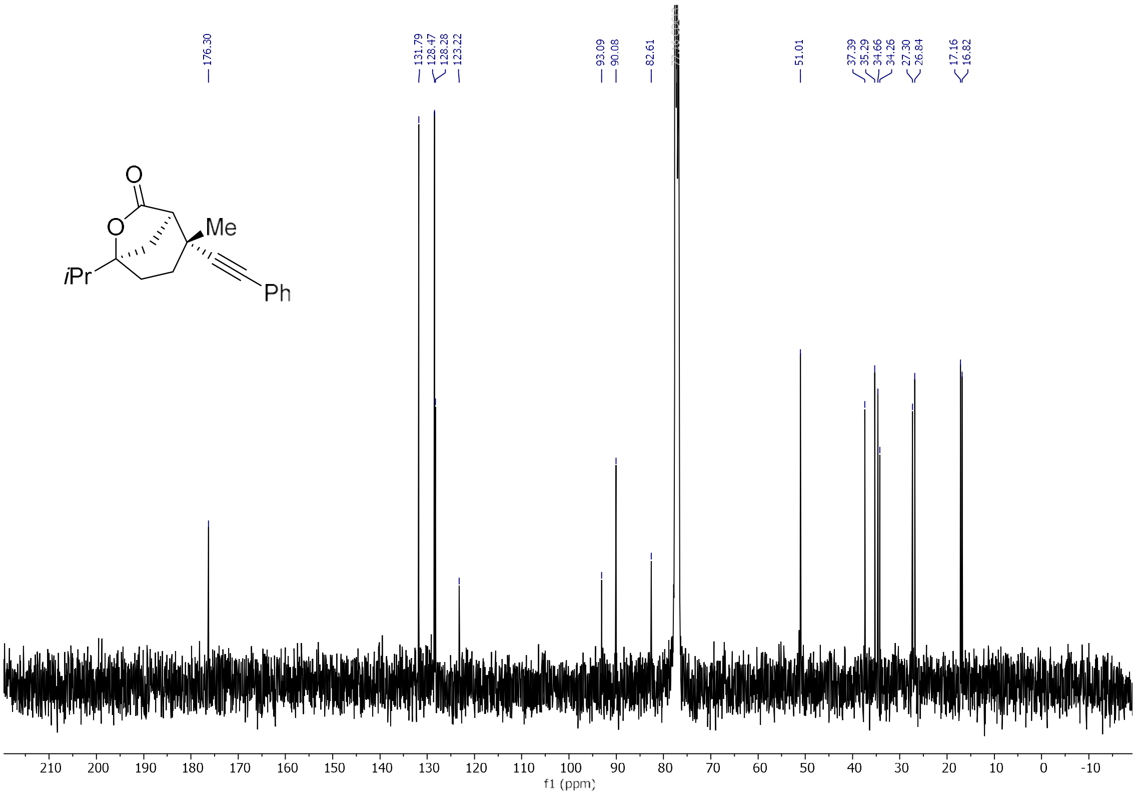

COSY (O1P = 3 ppm; SW = 6 ppm)

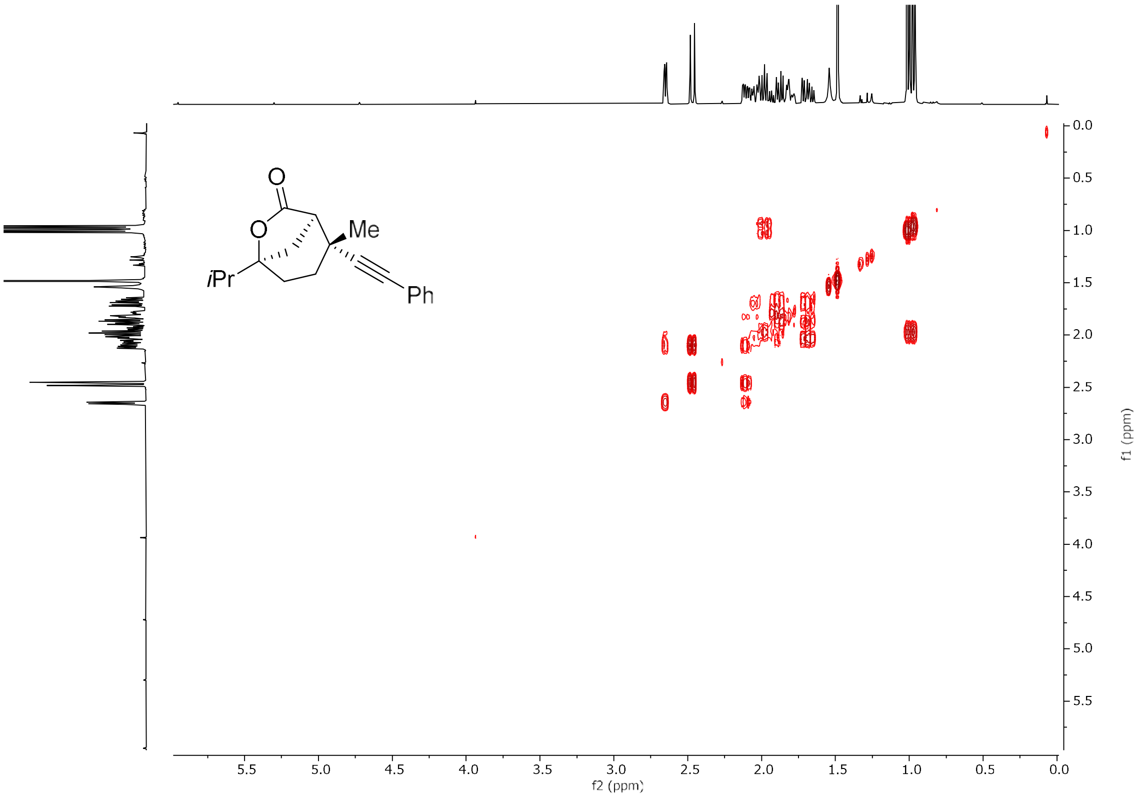

HSQC

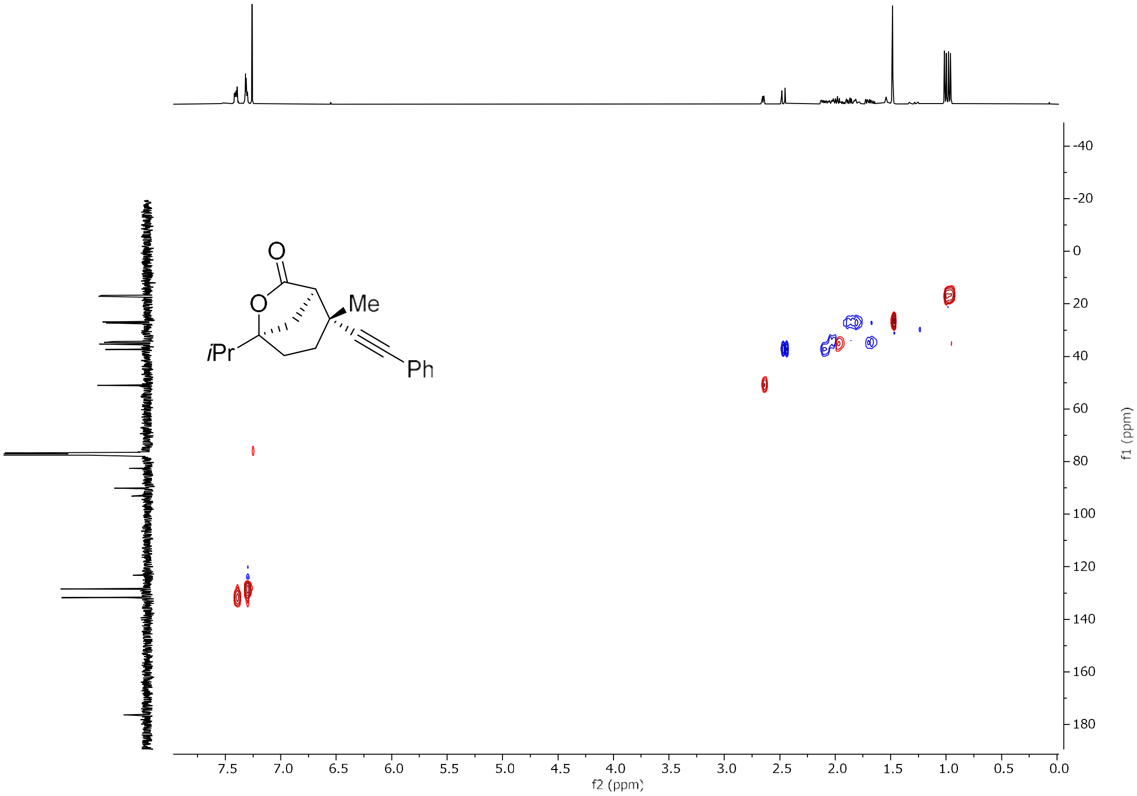

HMBC

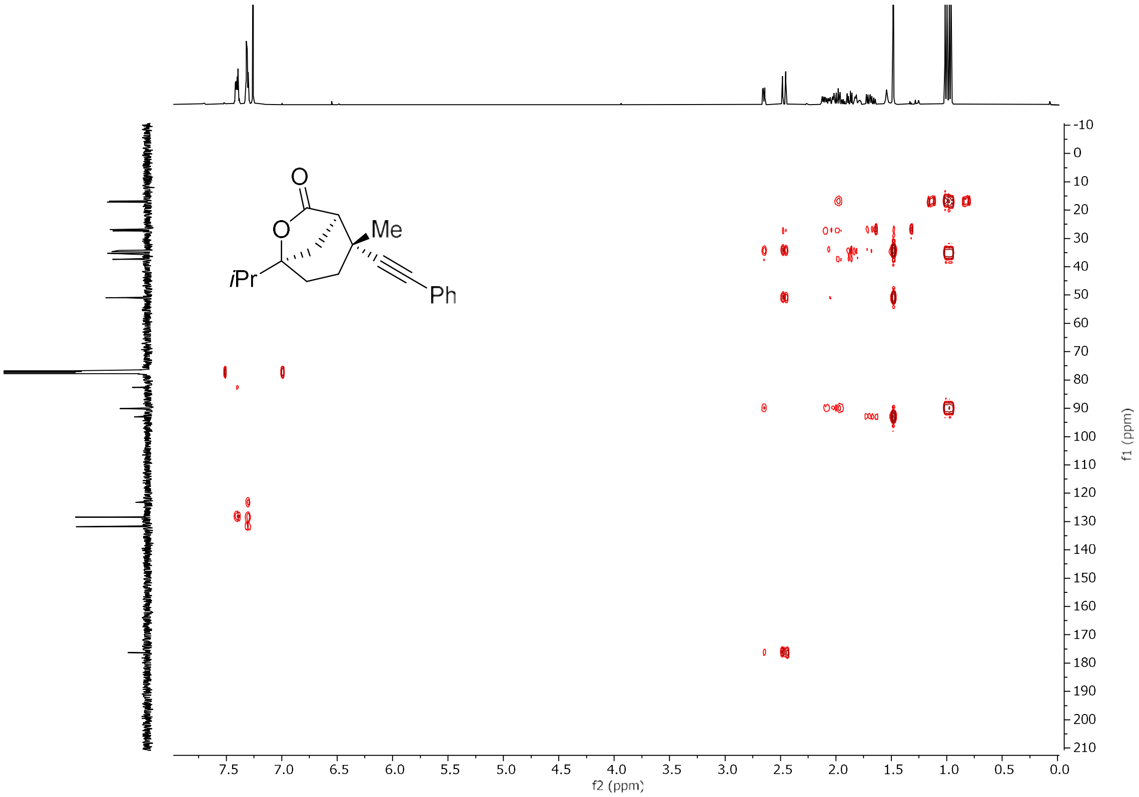

NOESY

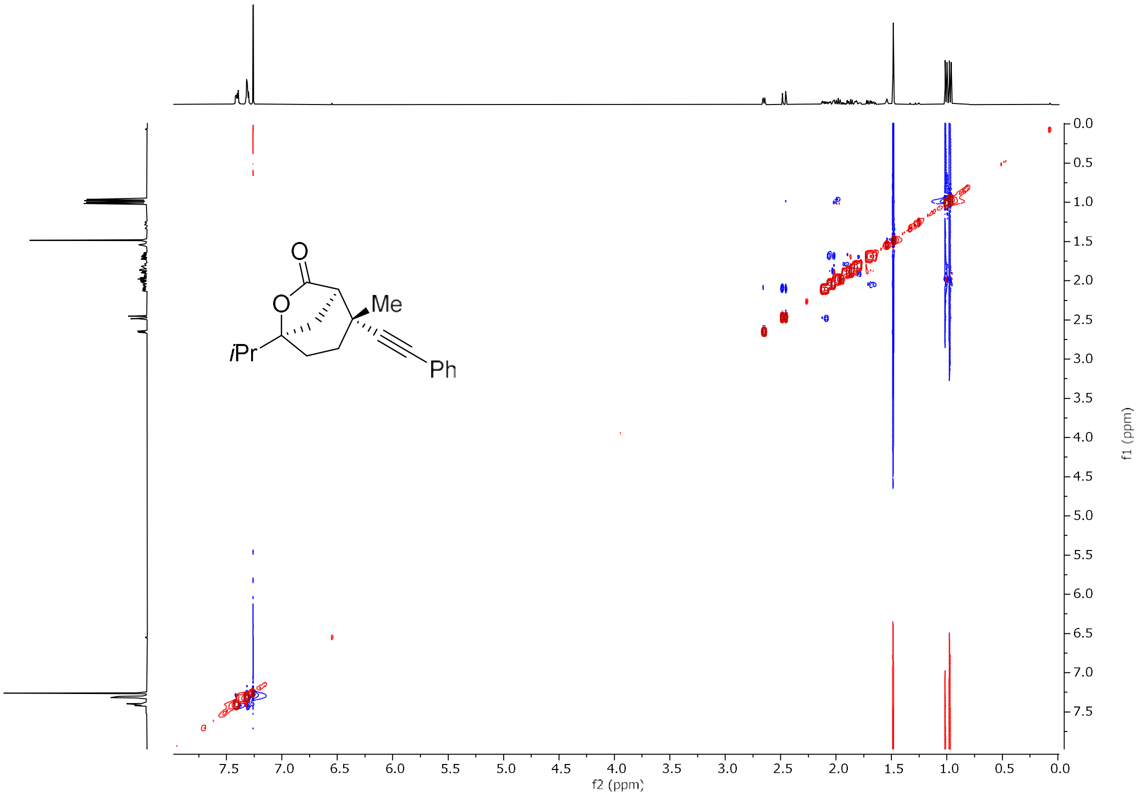

Compound **8a** (previously reported)

^{1}H NMR, $CDCl_3$, 400 MHz

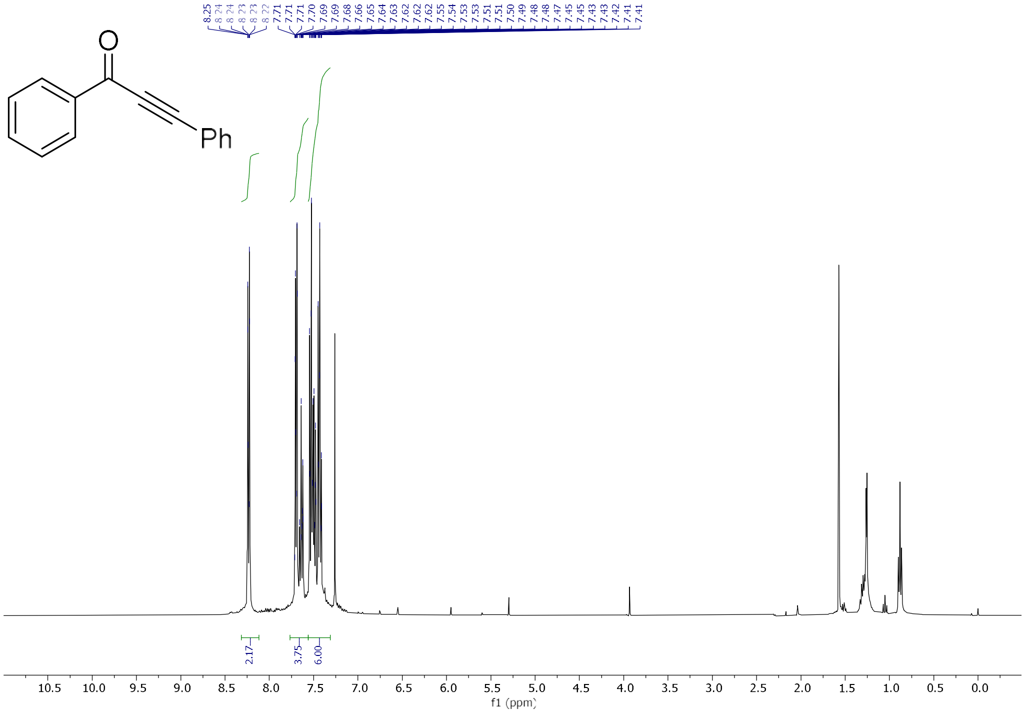

Compound **8b** (previously reported)

^{1}H NMR, $CDCl_3$, 400 MHz

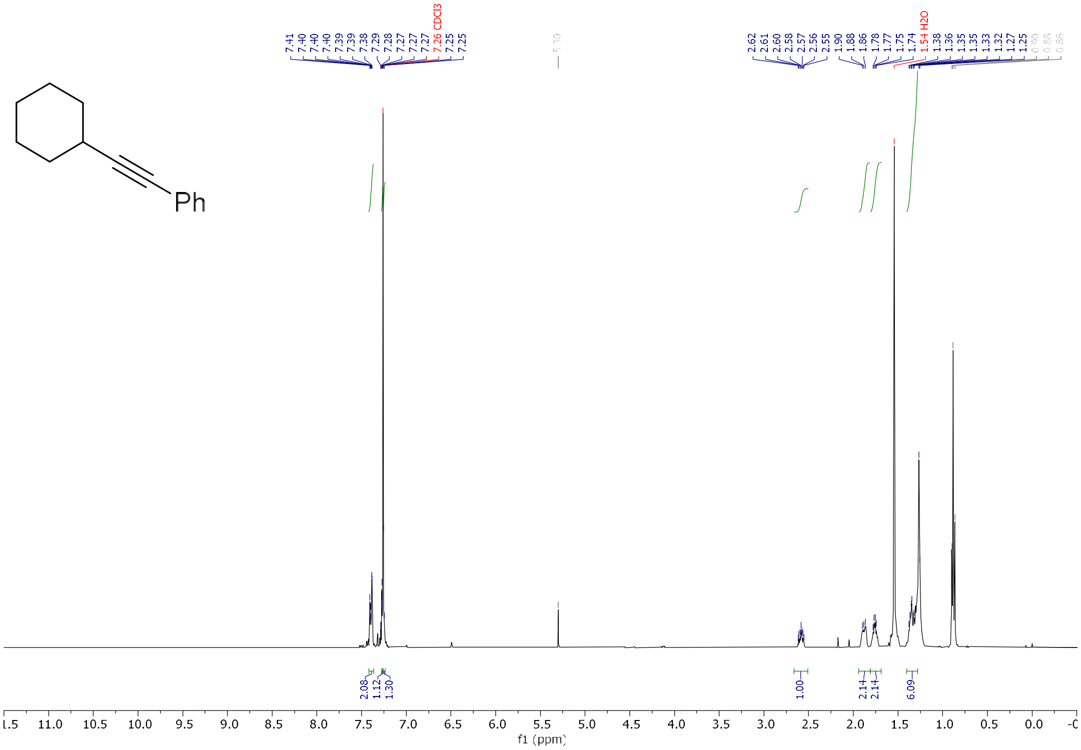

Compound **8c** (previously reported)

^{1}H NMR, $CDCl_3$, 400 MHz

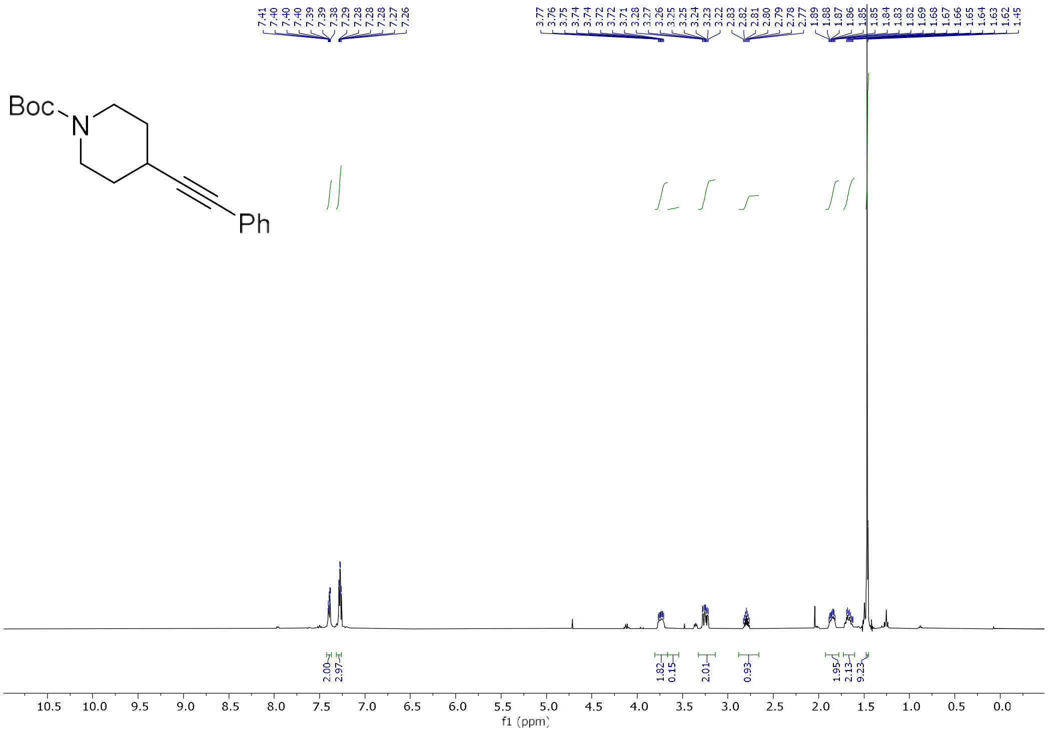

Compound **10a** (previously reported)

^{1}H NMR, CD_3CN, 400 MHz

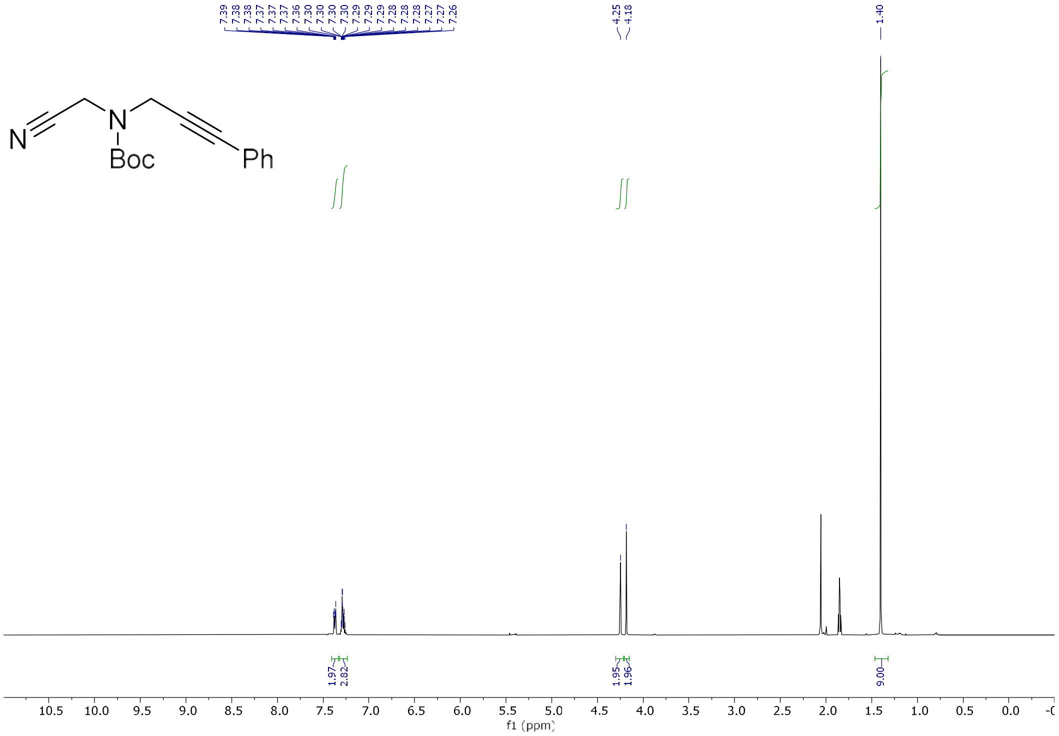

Compound **10b** (previously reported)

^{1}H NMR, $CDCl_3$, 400 MHz

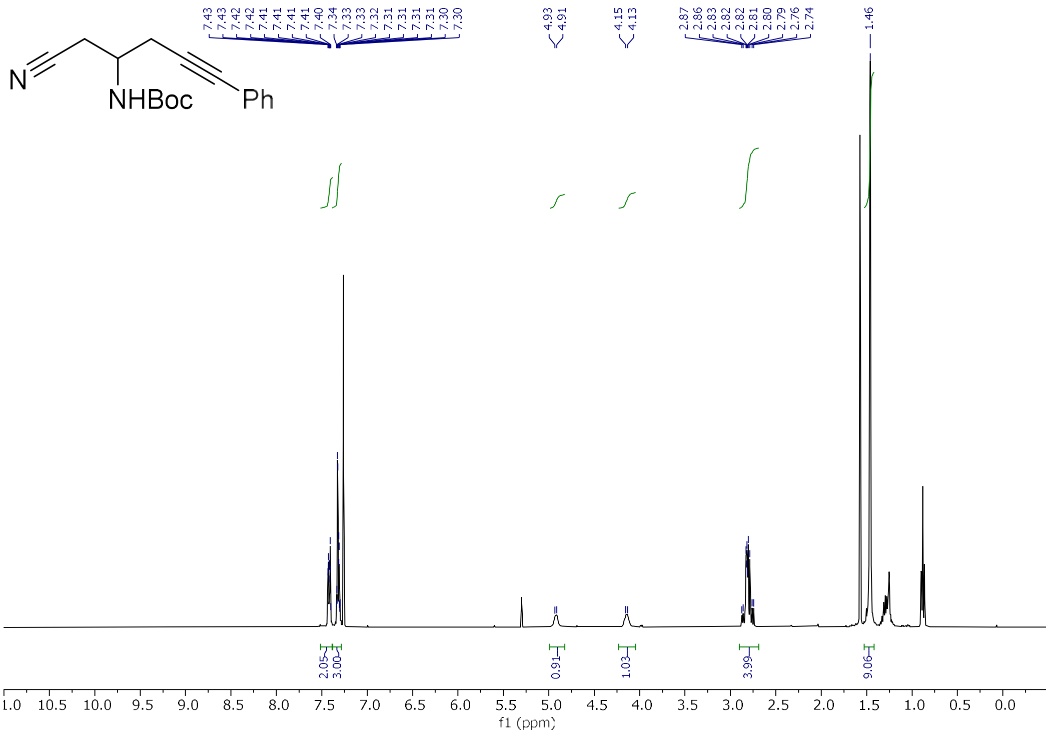

Compound **10c** (previously reported)

^{1}H NMR, $CDCl_3$, 400 MHz

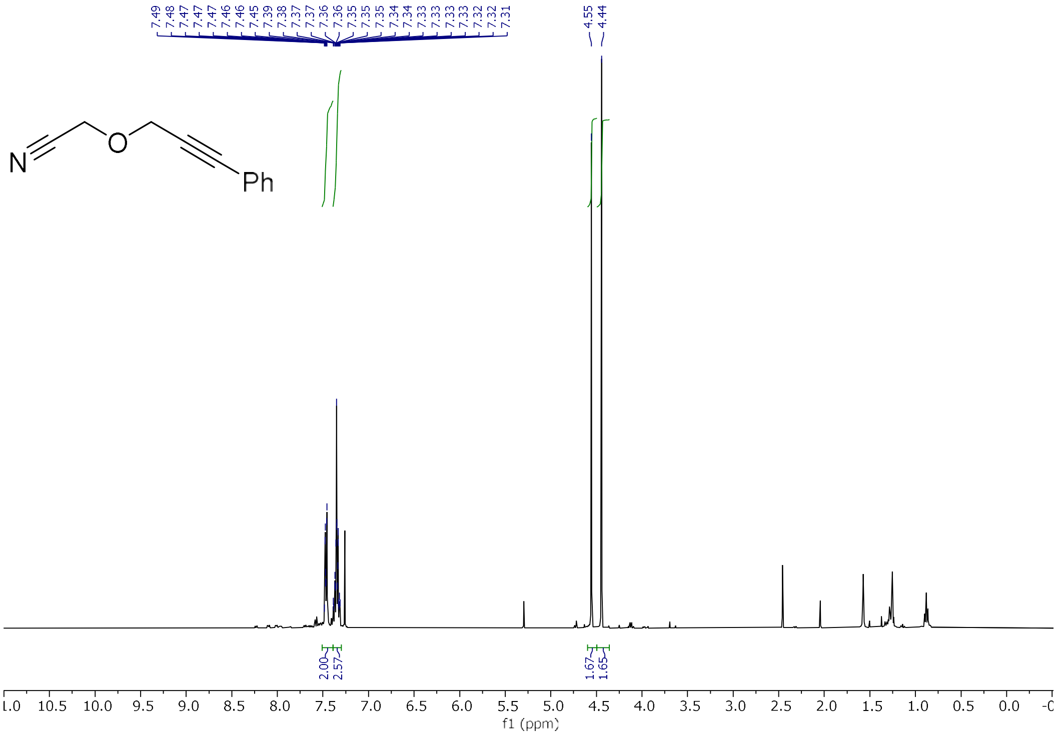

Compound **12a** (previously reported)

^{1}H NMR, $CDCl_3$, 400 MHz

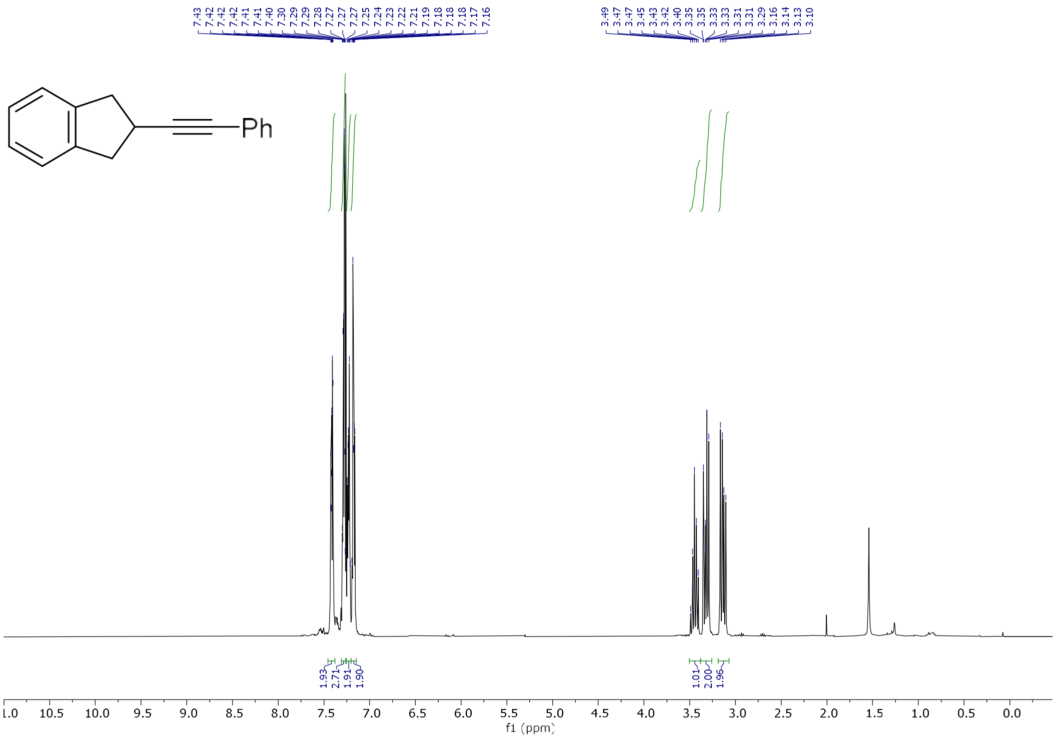

Compound **12b** (previously reported)

^{1}H NMR, $CDCl_3$, 400 MHz

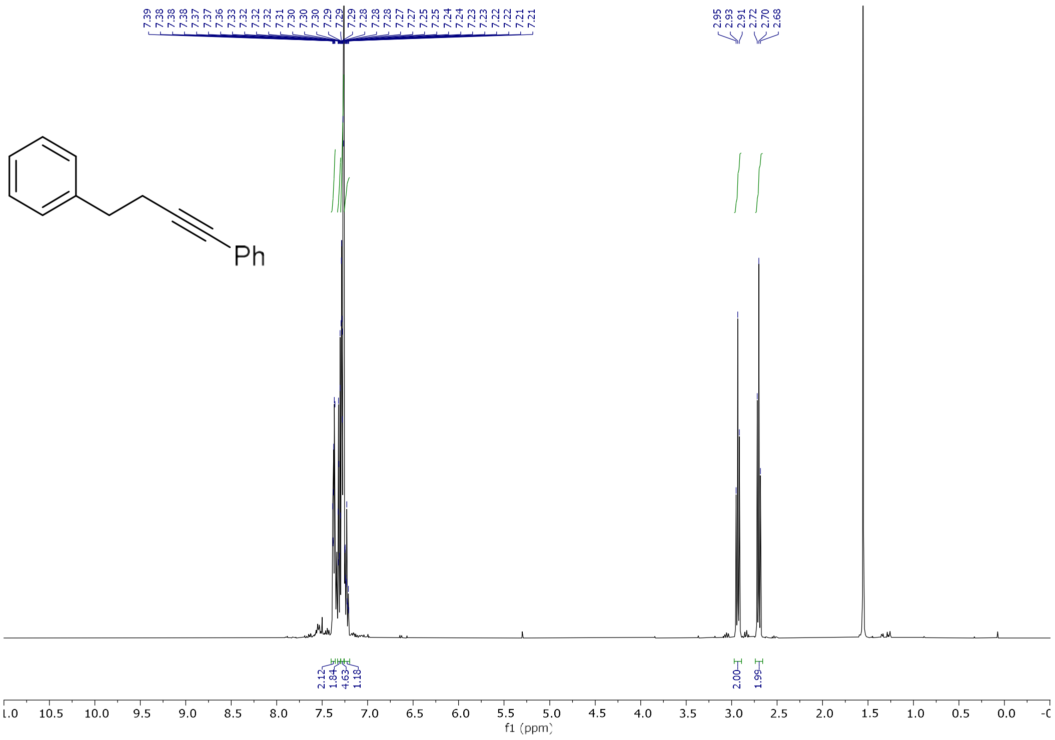

Compound **16**

^{1}H NMR, $CDCl_3$, 400 MHz

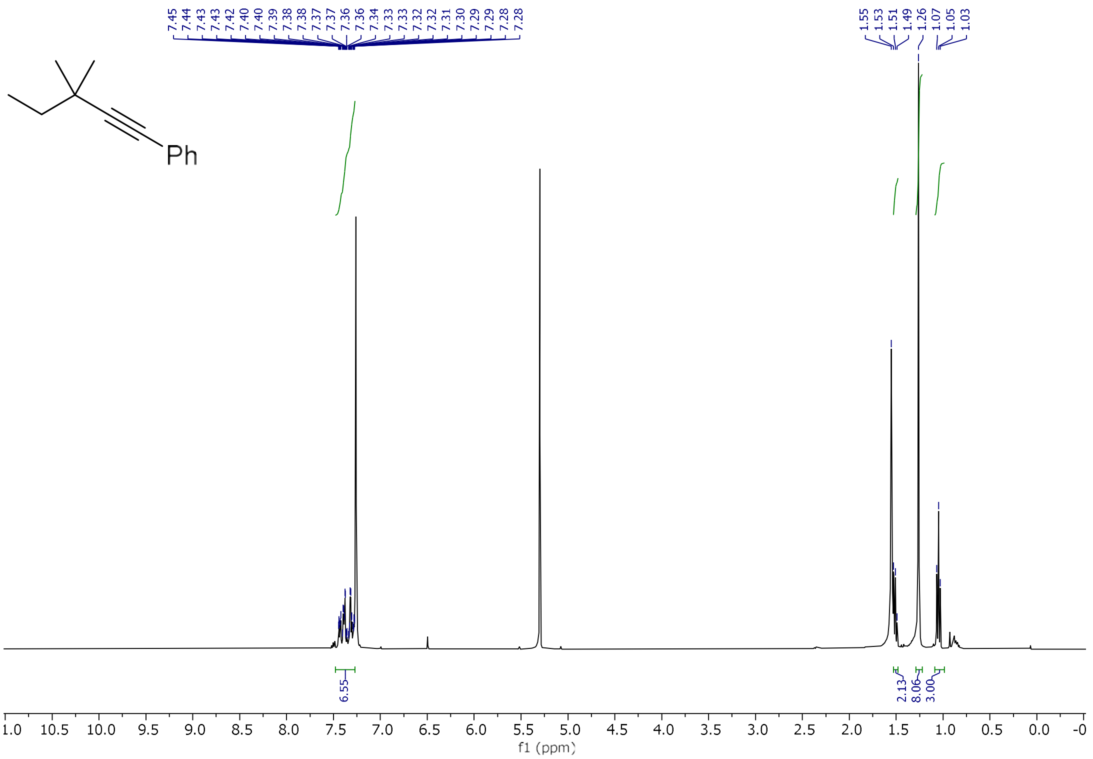

^{13}C NMR, $CDCl_3$, 101 MHz

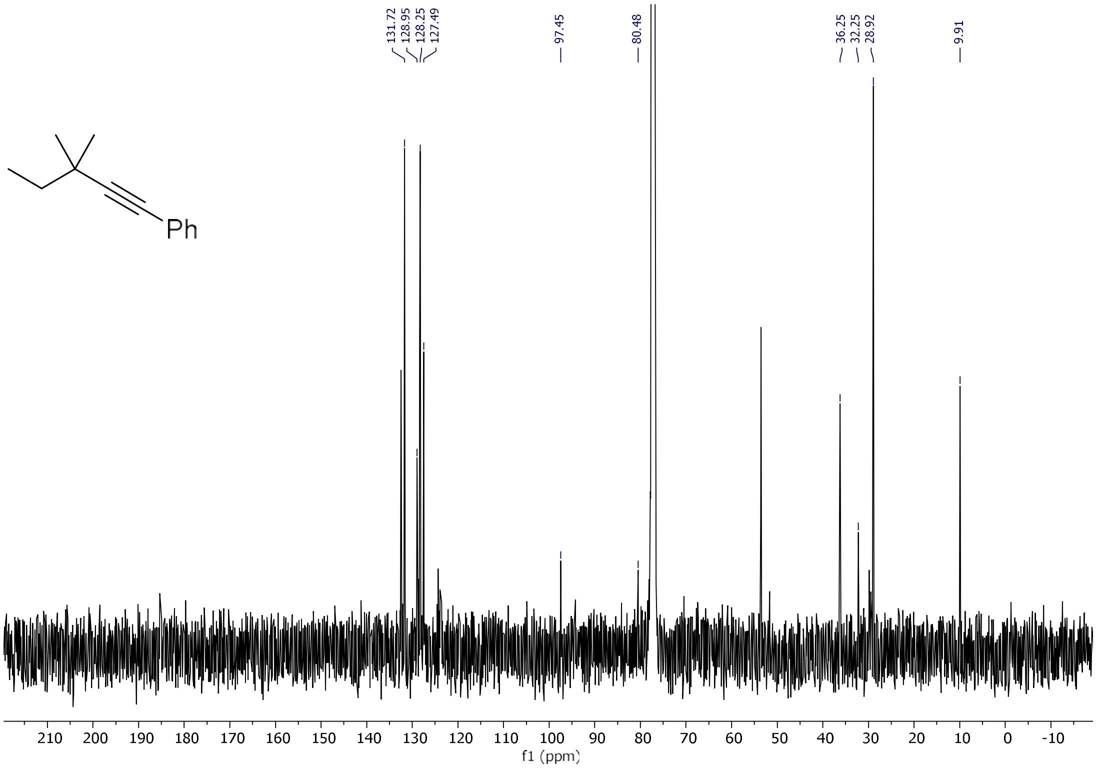

Compound **5b**

^{1}H NMR, $CDCl_3$, 400 MHz

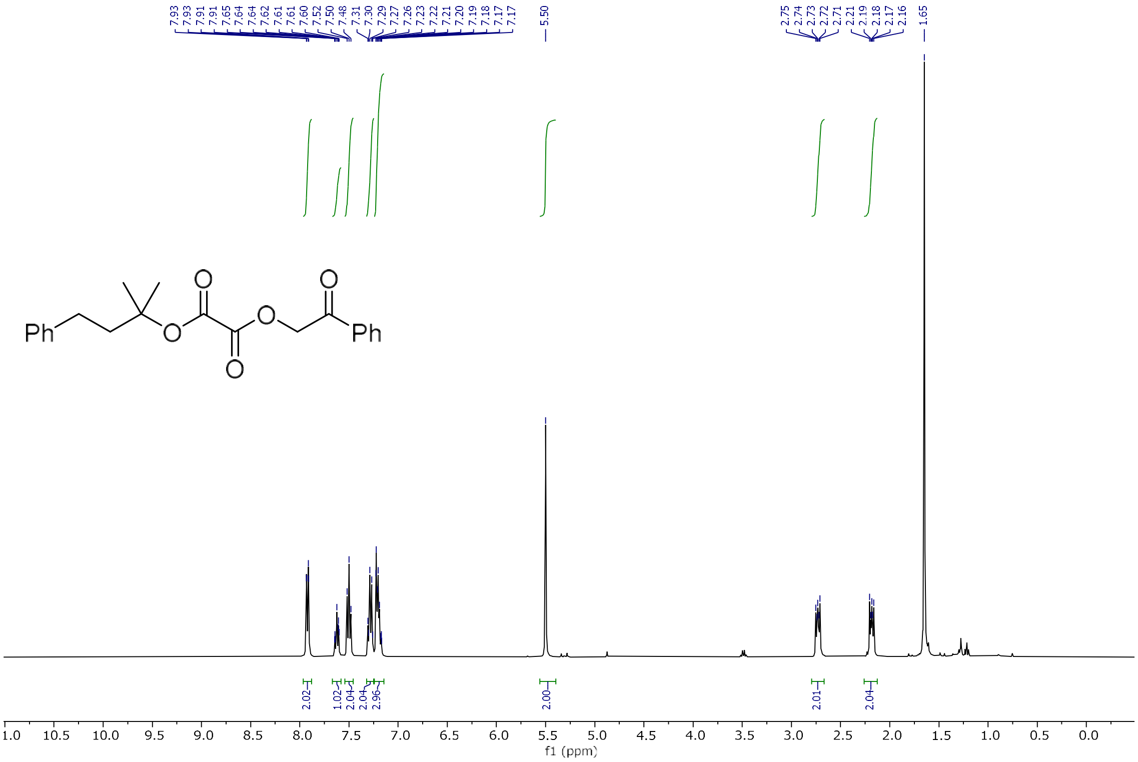

^{13}C NMR, $CDCl_3$, 101 MHz

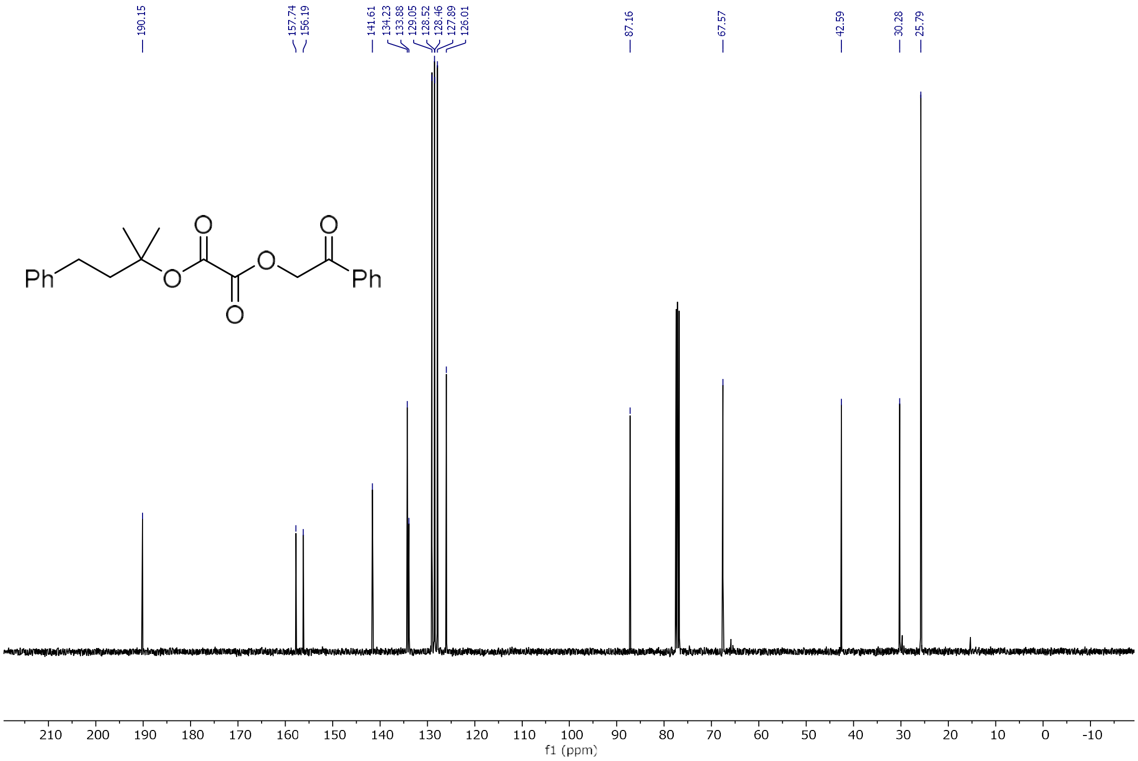

Supplement: Supplementary file 1 — Supporting Information [file ANIE-60-23827-s001.pdf]
